# *In silico* comparison of genomic regions containing genes coding for enzymes and transcription factors for the phenylpropanoid pathway in *Phaseolus vulgaris* L. and *Glycine max* L. Merr

**DOI:** 10.3389/fpls.2013.00317

**Published:** 2013-09-05

**Authors:** Yarmilla Reinprecht, Zeinab Yadegari, Gregory E. Perry, Mahbuba Siddiqua, Lori C. Wright, Phillip E. McClean, K. Peter Pauls

**Affiliations:** ^1^Department of Plant Agriculture, University of GuelphGuelph, ON, Canada; ^2^Department of Plant Sciences, North Dakota State UniversityFargo, ND, USA

**Keywords:** common bean, soybean, phenylpropanoid pathway, genome sequence, *in silico* map, comparative mapping

## Abstract

Legumes contain a variety of phytochemicals derived from the phenylpropanoid pathway that have important effects on human health as well as seed coat color, plant disease resistance and nodulation. However, the information about the genes involved in this important pathway is fragmentary in common bean (*Phaseolus vulgaris* L.). The objectives of this research were to isolate genes that function in and control the phenylpropanoid pathway in common bean, determine their genomic locations *in silico* in common bean and soybean, and analyze sequences of the *4CL* gene family in two common bean genotypes. Sequences of phenylpropanoid pathway genes available for common bean or other plant species were aligned, and the conserved regions were used to design sequence-specific primers. The PCR products were cloned and sequenced and the gene sequences along with common bean gene-based (g) markers were BLASTed against the *Glycine max* v.1.0 genome and the *P. vulgaris* v.1.0 (Andean) early release genome. In addition, gene sequences were BLASTed against the OAC Rex (Mesoamerican) genome sequence assembly. In total, fragments of 46 structural and regulatory phenylpropanoid pathway genes were characterized in this way and placed *in silico* on common bean and soybean sequence maps. The maps contain over 250 common bean g and SSR (simple sequence repeat) markers and identify the positions of more than 60 additional phenylpropanoid pathway gene sequences, plus the putative locations of seed coat color genes. The majority of cloned phenylpropanoid pathway gene sequences were mapped to one location in the common bean genome but had two positions in soybean. The comparison of the genomic maps confirmed previous studies, which show that common bean and soybean share genomic regions, including those containing phenylpropanoid pathway gene sequences, with conserved synteny. Indels identified in the comparison of Andean and Mesoamerican common bean *4CL* gene sequences might be used to develop inter-pool phenylpropanoid pathway gene-based markers. We anticipate that the information obtained by this study will simplify and accelerate selections of common bean with specific phenylpropanoid pathway alleles to increase the contents of beneficial phenylpropanoids in common bean and other legumes.

## Introduction

Common bean (*Phaseolus vulgaris* L.) and soybean (*Glycine max* L. Merr) belong to the Papilionoid subfamily of legumes. Both species are grown for their seeds and the protein, oil and starch they contain. The soybean, which is rich in protein (40 g kg^−1^) and oil (20 g kg^−1^) is the most economically valuable legume, worth over $100B/y (http://www.soystats.com/2012/Default-frames.htm). However, the common bean is the most important legume for direct human consumption (Broughton et al., 2003), due to it's high protein, complex carbohydrate and dietary fiber content. Beans also contain significant amounts of micronutrients and vitamins, including folate. Both species contain phenylpropanoid pathway-derived bioactive secondary metabolites such as flavonoids, lignans and isoflavones with potential medicinal properties (Mazur and Adlercreutz, 1998; Sirtori, 2001). Compared to soybean, the levels of isoflavones are far lower in common bean, but the levels of various compounds from the phenylpropanoid pathway that act as antioxidants are high. Thus, there is the potential to develop consumer awareness of common bean as a preferred source of these compounds in the diet, and with additional knowledge there is the possibility to increase the levels of these compounds in the common bean through conventional or marker-assisted selection (MAS).

Phenylpropanoids play vital roles in plants' interactions with their environments and have varied and important functions in processes such as: UV protection, disease resistance, nodulation (N_2_ fixation) and seed coat color determination. In contrast to the good understanding of genetics and biochemistry of seed and flower pigmentation in petunia, Arabidopsis or maize (Lepiniec et al., 2006), the roles of phenylpropanoids and the genes that code for the enzymes in the phenylpropanoid pathway are still poorly understood in the common bean.

Seed, flower and pubescence colors have utility in soybean breeding programs as hybridity markers. Commercial soybean has yellow seed, although natural variation in seed pigmentation exists. Seed coat color in soybean is determined by three independent loci (*I, T* and *R*) distinct from flower color-controlling loci (Owen, 1928; Palmer et al., 2004), some of which are associated with genes coding for enzymes of anthocyanin biosynthesis. For example, the *I* (inhibitor) locus (light, dark or saddled hilum color) was associated with six inverted chalcone synthase (CHS) repeats on chromosome Gm08 (Todd and Vodkin, 1996) and the *T* locus (tawny brown or gray pubescence color) co-localized with flavonoid 3′ hydroxylase (F3′H) on chromosome Gm06 (Toda et al., 2002; Zabala and Vodkin, 2003). However, the *R* locus (black, brown or striped seed coat color) mapped on chromosome Gm09 could not be linked with known anthocyanidin synthase (ANS), dihydroflavonol 4-reductase (DFR), or UDP-flavonoid glucosyltransferase genes. Findings by Gillman et al. (2011) suggest that loss of function mutation in an R2R3 MYB transcription factor results in brown hilum and brown seed coat.

Large genetic diversity exists between and within the Mesoamerican (Mesoamerica, Durango and Jalisco) and Andean (Nueva Granada, Chile, and Peru) gene pools of the common bean. They are distinguishable not only by geographic origin but also by seed size, growth habit, environmental adaptation, fertility barriers, resistance to diseases, phaseolin types, isozyme, and molecular marker diversities (Kwak and Gepts, 2009). In common bean, a number of genes control the colors (*P, R, J, D, G, B, V*, and *Rk*) and patterns (*T, Z, L, J, Bip*, and *Ana*) of seed coats (Prakken, 1970, 1972). Interactions among these genes are important in defining each of the major common bean market classes such as white, black, pinto or cranberry (McClean et al., 2002). Although none of the biochemical functions of the genes have been defined (Hosfield, 2001), it is believed that flavonoids are the pigments responsible for seed coat color in common bean (Beninger et al., 1998). Markers based on the genes that control seed coat color would greatly facilitate germplasm utilization in common bean breeding programs. RAPD (random amplified polymorphic DNA) and STS (sequence-tagged sites) markers associated with seed coat color genes have been identified and mapped on several linkage groups (Brady et al., 1998; Erdmann et al., 2002; McClean et al., 2002). Also, gene-based markers for several enzymes of the flavonoid/anthocyanin pathway (F3H, DFR, and ANS) have been developed (McClean et al., 2002), but their connection with color genes has not been established.

Phenylpropanoids consist of a large group of natural products containing a phenyl ring attached to a 3-C propane side chain (C6-C3). They are synthesized by the enzymes in the phenylpropanoid pathway (Holton and Maizeish, 1995; Dixon and Steele, 1999), which is unique to plants and probably is one of the most studied biochemical pathways. It starts from the aromatic amino acid phenylalanine and leads to the synthesis of a wide variety of phytochemicals including: simple phenylpropanoids, like phenylpropenes, and complex phenolics, such as lignins, lignans, coumarins, isoflavonoids, flavonoids, anthocyanidins, and proanthocyanidins (condensed tannins). The general (central) phenylpropanoid pathway involves three steps: deamination of phenylalanine into trans-cinnamic acid [catalyzed by phenylalanine ammonia-lyase (PAL)], hydroxylation of cinnamate into *p*-coumaric acid (4-coumaric acid) [catalyzed by cinnamate 4-hydroxylase (C4H)] and ATP-dependent formation of the CoA thioester 4-coumaroyl CoA (*p*-coumaroyl CoA) [catalyzed by 4-coumarate:CoA ligase (4CL)]. This compound is the substrate for several branches of the pathway leading to biosynthesis of lignin/lignans, flavonoid/anthocyanins and isoflavonoids (Winkel-Shirley, 2001; Dixon, 2005; Dixon et al., 2005; Vogt, 2010). Four structurally and functionally different isoforms of 4CL were detected in soybean (Lindermayr et al., 2002).

Most of the enzymes catalyzing individual steps of the pathway are identified and the genes coding for them are cloned for a number of plant species, including: *Arabidopsis thaliana* (Meyer et al., 1996; Aguade, 2001), *Petunia hybrida* (Holton et al., 1993), and maize (Andersen et al., 2008). The regulation of expression of the structural genes encoding the phenylpropanoid pathway enzymes is complex and is thought to occur primarily at the transcription level (Vom Endt et al., 2002; Grotewold, 2005; Hichri et al., 2011; Falcone-Ferreyra et al., 2012). In Arabidopsis, early biosynthetic genes (CHS, CHI, F3H, and FLS) are activated before the late biosynthetic genes (DFR, ANR) by the basic-Helix-Loop-Helix (bHLH) and R2R3-type MYB families of transcription factors (Pelletier et al., 1997). Similar activation patterns are observed in other dicotyledonous plants. A number of transcription factors regulating mostly the flavonoid/anthocyanin or lignin branches have been detected in many plant species, including legumes (Davies and Schwinn, 2003; Zhang et al., 2003; Yoshida et al., 2010; Zhao and Dixon, 2011).

The phenylproanoid pathway, especially the isoflavonoid branch is well characterized in soybean (Graham et al., 2008). However, the information about this important pathway in common bean is fragmentary and needs to be expanded to facilitate selection of lines with increased levels of these beneficial compounds. We initiated isolation and mapping genes coding for structural and regulatory proteins of this pathway. Only few genes (PAL1, CHS, CHI, CAD, and several PER) were characterized and their map positions identified before the initiation of this study.

Whole genome sequences are available for both soybean and common bean. The recent release of the complete draft of the soybean (cultivar Williams 82) genome sequence (v.1.0 in 2010) gives new insights into genome organization of this ancient paleopolyploid (Schmutz et al., 2010). The size of the genome is approximately 975 Mb, with over 46,000 genes captured in 20 chromosomes. As result of at least two rounds of polyploidization [~13 million years ago (MYA) and ~59 MYA] the soybean genome contains redundancy and gene duplications (Schmutz et al., 2010). The majority of low copy sequences are present in more than two copies [including numerous genes coding for enzymes of phenylpropanoid pathway (>100 PER, 54 LAC, 84 OMT, 214 UGT)] and a significant portion of the genome contains repetitive sequences. The early release of the common bean (Andean landrace G19833) genome sequence was in August 2012. The size of the common bean genome is approximately half of the size of the soybean genome (521.1 Mb), organized into 11 chromosomes with 27,197 loci containing 31,638 protein-coding transcripts (Phytozome, 13 Nov 2012). The availability of the genome sequences allows the organization of the individual genomes to be studied, as well as allowing for comparison of the two genomes at the nucleotide level. In general, for any gene/sequence in common bean, two corresponding homologous genes/sequences could potentially be found in soybean.

Moreover, Galeano et al. (2009) and McClean et al. (2010) showed that the two genomes, which were compared before the release of the common bean genome sequence (August 2012), share significant synteny. In general, regions homologous to regions in two soybean chromosomes were found for all 11 common bean chromosomes, with a minor marker rearrangement and/or sequence orientation. For example, three regions on the common bean chromosome Pv1 are syntenic to the regions of six soybean chromosomes: top (region between markers g1795 and g822) with soybean chromosomes Gm14 and Gm17, middle (region between markers g2490 and g1404) with soybean chromosomes Gm03 and Gm19, and bottom (region between markers g1224 and g2132) with soybean chromosomes Gm11 and Gm18 (McClean et al., 2010).

The objectives of the present work were to isolate genes coding for enzymes of phenylpropanoid pathway in common bean, determine *in silico* their genomic locations in common bean and soybean and analyze sequences of the *4CL* gene family in two common bean genomes. Using genomic information from the other plant species, we were able to increase the information about the structural and regulatory genes that control the synthesis of beneficial phenylpropanoid compounds in common bean. In addition, the work describes the development of *in silico* maps, and compares genomic regions containing genes coding for enzymes of the phenylpropanoid pathway in common bean and soybean so that the selection and/or molecular manipulation can be done in the future to increase their levels in these crops.

## Materials and methods

### Isolation of phenylpropanoid pathway genes

#### Cloning phenylpropanoid pathway gene fragments

***Plant material.*** Mature seeds of white (cultivar OAC Rex) or colored common beans were surface-sterilized by sodium hypochlorite (2.5% commercial bleach) with a drop of Tween 20 and grown on basal Murashige and Skoog (MS) media in Magenta tissue culture containers under low light. Seven day-old seedlings were washed, frozen in liquid nitrogen and kept at −80°C until they were used.

***Sequence selection and design of gene-based primers.*** Databases were searched for phenylpropanoid pathway gene sequences from the common bean or other plant species. The sequences were aligned in ClustalW at EBI (http://www.ebi.ac.uk/clustalw/) and PCR primers were designed to amplify conserved regions using the Primer3 program (Rozen and Skaletsky, 2000), which were synthesized by Sigma-Aldrich (Oakville, ON, Canada). The majority of the primers were: 18–24 base pairs long, selected to have a GC content of 40–60% with fewer than four contiguous identical bases and melting temperatures between 55 and 65°C. The gene-based primers were used with total common bean cDNA or genomic DNA in PCRs to amplify fragments of a number of genes of the phenylpropanoid pathway in common bean.

***Extraction and amplification of nucleic acids.*** Total RNA was extracted from the 7-day-old common bean seedlings using the Trizol™ Reagent (Invitrogen Inc., Burlington, ON, Canada). The RT-PCR was performed with the RETROscript® first strand synthesis kit (Ambion, Austin, TX, USA) according to manufacturer's instruction. PCR amplification was performed in a total volume of 20 μl containing 1x PCR buffer (supplied with enzyme), 3 mM MgCl_2_ (supplied with enzyme), 0.5 mM each of dNTPs (GE Healthcare Bio-Sciences Corp., Piscataway, NJ, USA), 1.6 U *Taq* DNA Polymerase (Invitrogen), 5 μM each forward and reverse primers, and 0.5 μl of common bean RT reaction. The reaction mixture was amplified with a Techne Touchgene Gradient Thermal Cycler (Kracheler Scientific, Albany, NY, USA). The amplification program consisted of an initial denaturation step at 94°C for 2 min, followed by 35 cycles of denaturation at 94°C for 30 s, annealing at gradient range 55–65°C for 45 s, and extension at 72°C for 1 min and finished by final extension at 72°C for 7 min.

Genomic DNA was isolated from the first trifoliate leaf from the white (or colored) common bean in 2% (w/v) CTAB buffer (Doyle and Doyle, 1990). The STS PCR was as for RT-PCR except that 25 ng of common bean genomic DNA was used as template. The reaction mixture was amplified with a PTC-100™ Programmable Thermal Controller (MJ Research Inc., Watertown, MA, USA). The amplification program consisted of an initial denaturation step at 94°C for 2 min, followed by 35 cycles of denaturation at 94°C for 30 s, annealing at 55–60°C for 45 s, and extension at 72°C for 1 min and finished by final extension at 72°C for 7 min.

The RT-PCR and STS PCR products were run on a 1% (w/v) agarose gel in 1x modified TAE buffer (Millipore Corporation, Billerica, MA, USA) for 3 h at 80 V, stained with ethidium bromide and visualized under UV light. The fragments were excised from the gel, purified with Montage DNA Gel extraction kit (Millipore) and cloned using a TOPO™ TA cloning kit (Invitrogen) according to the manufacturer's instructions. Plasmid DNA was extracted with QIAprep® Miniprep kit (Qiagen, Mississuaga, ON, Canada) and used as template for cycle sequencing using the CEQ™ 2000XL DNA Analysis System (Beckman Coulter, Fullerton, CA, USA). Sequences were compared to the existing sequences from common bean or other plant species to ensure that the target genes were cloned using BLAST default parameters with a cut off value of 1E^−10^ (http://www.ncbi.nlm.nih.gov/BLAST/). Sequences were verified and submitted to GenBank under accession numbers CW652092 to CW652106 (EST) and CV670734 to CV670763 (GSS).

#### Cloning TT2 transcription factor fragments

RNA was extracted from 1–1.5 cm whole, immature siliques of cranberry-like Witrood common bean using the Qiagen RNeasy Plant Mini Kit (Qiagen) as per manufacturer's instructions with the following two modifications. Ambion RNA Isolation Aid (Applied Biosystems, Streetsville, Canada) was included in the Qiashredder step in order to increase RNA yield and the use of Ambion DNA-free Turbo DNase (Applied Biosystems) optimized the removal of contaminating DNA. The absence of contaminating DNA (less than 10 copies per sample) was confirmed by real-time PCR analysis of the RNA samples with primers for the actin gene. Synthesis of cDNA from the RNA samples was carried out according to manufacture's instructions for Ambion's RETROscript Reverse Transcription for RT-PCR (Applied Biosystems). Successful reverse transcription reactions were confirmed for all samples by post-transcription real-time PCR analysis of the cDNA for the actin gene sequence. Standard PCR was done using cDNA as template. Annealing temp was 54.7°C with forward primer 5′-TCTTCTCACATCAACACAAAC-3′ and reverse primer 5′-CATACTAGAAGAATTGGAATG-3′. PCR fragments were excised from the agarose gel, cloned into TOPO vector (Invitrogen) and sequenced (Genomics Facility-Advanced Analysis Centre, University of Guelph, Guelph, ON, Canada).

#### Isolation of selected set of phenylproanoid pathway genes

The common bean BAC library (constructed from *Hin*dIII digested and size selected landrace G19833 genomic DNA), obtained from the Clemson University Genomics Institute (CUGI, http://www.genome.clemson.edu/) was screened with DIG-labeled probes of six phenylproanoid pathway genes (CHS-A-Z, CHS-B-Z, DFR-Z, Myb15-Z, PAL2-Z, and PAL3-Z) amplified from cloned genomic DNA. The hybridization probes were synthesized using a digoxygenin (DIG) PCR Labeling kit (Roche Diagnostics Canada, Laval, QC, Canada) according to the manufacturer's instructions. Membranes [prepared by CUGI (containing 55,296 BAC clones of common bean landrace G19833 genomic DNA)] were screened by Southern hybridization with the DIG labelled probes. The identities of the BAC clones to which the probes strongly hybridized were identified on the bases of the membrane positions and patterns of the hybridization spots seen in the images. BAC clones strongly hybridized with the probes were obtained from the CUGI and plasmid DNA was extracted using plasmid extraction kit (Qiagen). The presence of probe sequences was verified by PCR amplification using gene-specific primers and the PCRs were compared to the original genomic PCR bands. One clone per gene with the appropriate size was selected for sequencing. *Escherichia coli* cells carrying individual BAC clones were grown on LB plates supplemented with 12.5 μg/ml of chloramphenicol at 37°C. For each BAC clone, one colony was picked and cultured in 2 ml LB supplemented with 12.5 μg/ml chloramphenicol. Approximately 10–15 μg of DNA from each clone was used for 454 next generation sequencing at the National Research Centre Council (Saskatoon, SK, Canada). Sequence reads were trimmed for contamination [bacterial (*E. coli*) genomic DNA, BAC clone backbone (pBeloBAC11) and vectors] in Univec database using CLC Genomics Workbench (http://www.clcbio.com) and assembled using the CLC Genomics Workbench reference assembly algorithm and genome sequence of common bean (http://www.Phytozome.org) as a reference. The consensus sequence was analyzed for the presence of coding regions with GENSCAN (http://genes.mit.edu/GENSCAN.html) and FGENESH (http://www.softberry.com/) using *Medicago truncatula* and Arabidopsis, respectively as the model organisms. Their putative functions were determined by comparing the coding regions to known genes at the GenBank.

#### Isolation of dihydroflavonol 4-reductase (DFR) paralogs

***Growing plants and sample collection.*** Seeds from common bean cultivar OAC Rex were grown in the greenhouse at 25/22°C for 16 h photoperiod with a light intensity of 150 μol m^−2^ s^−2^. The immature seeds (1–3 mm) were collected from green pods and immediately flash frozen in liquid nitrogen and kept at −80°C until extraction of total RNA.

***Cloning DFR2 and DFR3.*** Sequence information for *DFR-Z* gene obtained from the G19855 BAC clone PV-GBa 0072I22 (CUGI) was used to isolate two DFR paralogous cDNAs. Total RNA was extracted from immature seeds using RNeasy Plant Mini Kit (Qiagen) and treated with DNase (Ambion). The cDNA was amplified with 1 μg of DNase treated RNA with qScript ™ cDNA supermix (Quanta Biosciences, Gaithersburg, MD, USA) and used as a template in all PCR reactions. Initial PCR was performed with degenerate primers (forward- 5′-ATCGGRTCRTGGCTTGTYATG-3′ and reverse- 5′-GCTYCWGTRWACATRTCYTCYAAGSTGTAYTTRAA-3′) de-signed from the highly conserved sequences of DFR proteins from several closely related plant species. The PCR program was set up with an initial denaturation step of 95°C for 2 min followed by 34 cycles of 95°C (45 s)/58°C (45 s)/72°C (1 min) and a final extension step at 72°C for 10 min. The cDNA fragments were cloned into TOPO vector (Invitrogen) and sequenced. The preliminary analysis revealed two similar sequences representing two different common bean *DFR* paralogous genes (named *DFR2* and *DFR3*). The 5′ and 3′ sequences were obtained by 5′ and 3′ Rapid Amplification of cDNA (RACE; Clontech, Mountain View, CA, USA). For the first round amplification of *DFR2* and *DFR3* upstream and downstream sequences, PCR was performed with DFR2 reverse primer, 5′-ACCAGGCAGCTCCACCAAATGCTTCACCTT-3′ and forward primer 5′-ACCTCTTGTTGTTGGTCCCTTTCTCATGCCAACAATGCC-3′, DFR3 reverse primer, 5′-TGCACCTGGAAGTTCCACCAAATGCTTCACCTTCTTCAT-3′ and DFR3 forward primer 5′-ACCTCTTGTTGTTGGTCCCTTTCTCATGCCTACAATGCCA-3′ with an initial denaturation step of 95°C for 1 min followed by 32 cycles of 95°C (30 s)/68°C (1 min) followed by a cycle of 68°C (1 min)/70°C (10 min) and a final extension step of 72°C for 10 min. The second round nested PCR was performed with DFR2 reverse primer, 5′-TCGAACGGTGTAGCCACGCTCGAGGA-3′ and forward primer 5′-CCTAATCACTGCCCTTTCGCCCATCACAGGAAATGAGG 3′, DFR3 reverse primer, 5′ TCGAACGGTGTAGCCACGCTCCATTAGCC-3′ and DFR3 forward primer 5′- CACTGCTCTTTCACTCATCACAGGAAATGAAGGGCATTACC-3′ with an initial denaturation temperature of 95°C for 2 min followed by 32 cycles of 95°C (45 s)/64°C (45 s)/72°C (45 s) followed by a final extension step at 72°C for 10 min. The amplified fragments were cloned into TOPO vector and sequenced. Finally, the coding sequences of *DFR2* and *DFR3* were amplified with DFR2 forward primer, 5′-ATGGGTTCAGTGTCTGAAACTG-3′ and reverse primer 5′-TAGTTCTGAATGATGCCATTCATG-3′; DFR3 forward primer, 5′-ATGGGTTCAACTTCCGAATCC-3′ and reverse primer, 5′-CTATTTGTGCATGGTGCCATTAAC-3′.

### Development and comparison of common bean and soybean sequence maps

#### Sequence collection used to compare common bean and soybean genomes

Sequences used to develop and compare common bean and soybean *in silico* maps are listed in Table 1.

**Table 1:** **Phenylpropanoid pathway gene and marker sequences used in BLAST analyses**.

**Sequence**	**Number**	**Source**
Phenylpropanoid pathway genes	200	GenBank
Cloned common bean phenylpropanoid pathway gene sequences—published	35	GenBank
Cloned common bean phenylpropanoid pathway gene sequences—not published	11	University of Guelph
Common bean seed coat color gene-based markers	10	McClean et al., 2002/LIS^a^
Soybean seed coat color gene-based markers	12	Yang et al., 2010/GenBank
Common bean “g” markers	300	LIS
Common bean SSRs	60	LIS/PhaseolusGenes
Soybean SSRs	10	SoyBase
Total	638	

a Legume information system (http://www.comparative-legumes.org/).

***Phenylpropanoid pathway gene sequences.*** Literature and databases were searched for phenylpropanoid pathway gene sequences from common bean, soybean or other plant species. The EC numbers of all known phenylpropanoid biosynthesis enzymes in soybean were recovered from the Kyoto Encyclopedia of Genes and Genomes (KEGG, http://www.genome.jp/kegg/) pathway database, the MetaCyc database (MetaCyc.org) of metabolic pathways and enzymes and the BioCyc (biocyc.org) collection of pathway/genome databases.

***Molecular marker sequences.*** Two types of molecular markers were used: common bean gene-specific [g (whole genome gene-based; McConnell et al., 2010)] and random RAPD, STS and simple sequence repeats (SSRs). Marker sequences were retrieved from the Legume Information System (LIS, http://www.comparative-legumes.org/) and/or PhaseolusGenes database (http://phaseolusgenes.bioinformatics.ucdavis.edu/) using the default settings.

*Common bean g markers.* The list of the g markers (matrix) shared between common bean and soybean was obtained at the LIS with the maps and comparison point where common bean PvMcCleanNDSU2007 map containing g markers was compared with soybean Gm JGI 8X Sequence Assembly at LIS (http://cmap.comparative-legumes.org/). In this work 300 g marker sequences were used. In some instances, only primer sequences were available.

*Markers associated with seed coat color gene.* Sequences of common bean markers associated with the seed color genes (Erdmann et al., 2002; McClean et al., 2002) were retrieved from the LIS. Sequences of soybean seed coat color gene-based markers were from Yang et al. (2010).

*SSR markers.* Common bean SSRs were used to fill gaps in positioning markers associated with the seed color genes on sequence maps. Only those shared by both, soybean and common bean were mapped *in silico*. Initially, the positions of the common bean SSR markers in the soybean genome were extracted from the LIS (McClean et al., 2010) and then updated with the soybean v.1.0 release. In soybean, sequences of SSRs flanking the markers associated with the seed coat color genes (Yang et al., 2010) were retrieved from the SoyBase/GenBank. Soybean SSRs flanking seed coat color genes reported by Yang et al. (2010) were also used in *in silico* mapping.

#### Development and comparison of phenylpropanoid pathway gene sequence-based maps in common bean and soybean

***Common bean and soybean phenylpropanoid pathway gene sequence-based maps.*** Available nucleotide sequences (genes and markers) were BLASTed against soybean (v.1.0) and common bean (early release v.1.0) genomes at Phytozome (soybean at http://www.phytozome.net/soybean.php and common bean at http://www.phytozome.net/commonbean_er.php) using default parameters. The significance threshold *E*-value was set at E^−30^ and 150 bp minimum sequence length alignments with query sequences. In soybean, the top two hits on two different chromosomes were selected and labeled as 1 (copy 1 for the top hit) and 2 (copy 2 for the second hit). Where the top hit was a cluster of genes (*CHS*), several sequences were selected and the first was labeled as 1-a, second as 1-b, etc. The name of phenylpropanoid pathway gene sequences consisted of enzyme/gene abbreviation and accession number. Sequence positions on chromosomes were indicated by the first nucleotide (nt) on the physical map (Phytozome).

***Common bean-soybean sequence-based comparative map.*** The current map was developed *in silico* based on the McClean et al. (2010) common bean-soybean comparative map. All sequences shared by common bean and soybean are indicated. Synteny blocks were defined by the presence of three or more sequence features shared by the common bean and soybean genomes. The orientation of a marker/gene sequence on a chromosome was indicated by an up (↑, reverse)/down (↓, forward) arrow.

### Use of common bean and soybean whole genome sequences

#### Sequence-based analysis of a gene family coding for 4-coumarate:CoA-ligase (4CL) enzyme of general phenylpropanoid pathway in common bean and soybean

Using soybean *4CL4* gene (Accession X69955) as a query, Phytozome was searched for homologous proteins from common bean and soybean. Genomic and protein sequences were retrieved for both species. Translations of genomic sequences into amino acids were checked using FGENESH with the *Medicago* gene model and/or ExPASy (http://web.expasy.org/translate/) at the Swiss Institute of Bioinformatics (SIB). Amino acids and DNA sequence alignments and constructions of phylogenetic trees were done in CLC Genomics Workbench 3 using a Neighbor Joining (NJ) algorithm. The accuracy of alignments was confirmed with ClustalW at EBI and edited manually. Twenty-one common bean and soybean 4CL sequences selected based on the similarity to four published soybean 4CLs were re-analyzed. Conserved domains of the selected 4CL proteins were analyzed at the NCBI Conserved Domain Database (CDD, http://www.ncbi.nlm.nih.gov/Structure/bwrpsb/bwrpsb.cgi) (Marchler-Bauer et al., 2011).

#### Sequence polymorphism in common bean 4CL genes

Eight common bean *4CL* genes were selected for polymorphism analysis and marker development. Reference common bean (Andean landrace G19833) *4CL* sequences were BLASTed against OAC Rex (Mesoamerican) genome assembly in CLC Genomics Workbench. OAC Rex scaffold and contig segments containing *4CL* genes were then compared with the corresponding G19833 sequences in ClustalW at EBI. The alignments were adjusted manually and searched for sequence polymorphism (SNPs and indels). OAC Rex *4CL* genes that were verified were submitted to the GenBank under accession numbers KF303286 (*4CL-1*) to KF303293 (*4CL-8).*

Sequencing of Mesoamerican common bean cultivar OAC Rex is one of the major objectives of an ongoing multi institutional (University of Guelph, University of Windsor, University of Western Ontario and Agriculture and Agri-Food Canada) *Phaseolus* genomics project (www.beangenomics.ca). Two mate pair libraries were prepared (50 bp read length) and sequenced using the Illumina HiSeq platform at the Center for Applied Genomics (Toronto, ON, Canada)]. Scaffolding and contig assembly were performed using SOAP2 *de novo* and ABYSS (Perry et al., 2013; DiNatale et al., unpublished). Currently, the OAC Rex genome is assembled into megabase contigs; pseudo-chromosome builds have been initiated and public release is expected to be in 2014.

## Results

### Isolation of phenylpropanoid pathway genes in common bean

#### Cloning phenylpropanoid pathway gene fragments

**Figure 1:**
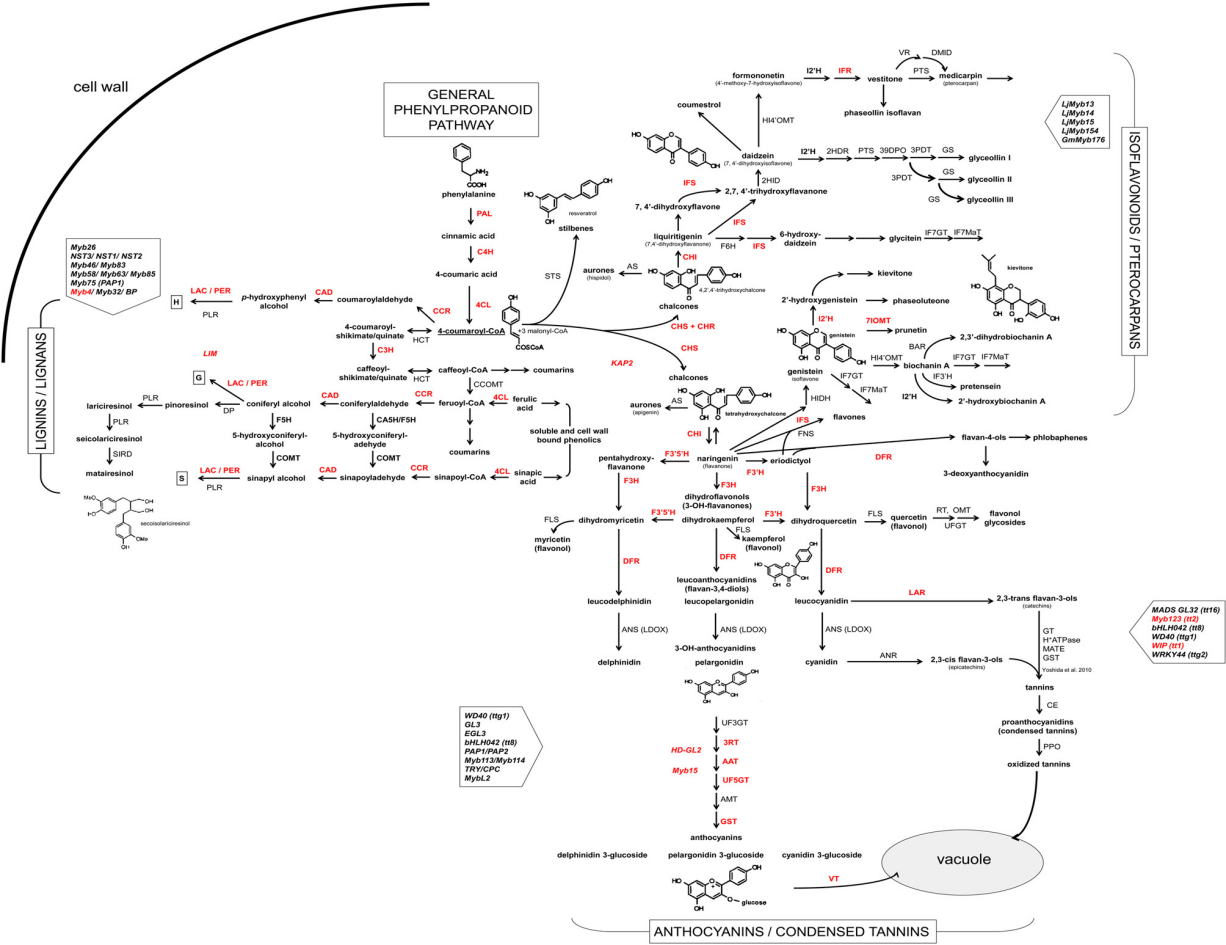
**Scheme of the phenylpropanoid pathway in common bean and soybean**. Enzymes and transcription factors of the phenylpropanoid pathway [based on biochemical and genetic studies of several plant species and (KEGG Pathway database (http://www.genome.jp/kegg/pathway.html)] are presented in uppercase letters: PAL, phenylalanine ammonia lyase (EC:4.3.1.24); C4H, cinnamate 4-hydroxylase (EC:1.14.13.11); 4CL, 4-coumarate:CoA ligase (EC:6.2.1.12); C3H, 4-coumarate 3-hydroxylase (EC:1.14.13.-); COMT, caffeate O-methyltransferase (EC:2.1.1.68); F5H, ferulic acid 5-hydroxylase (EC:1.14.-.-); CCoOMT, caffeoyl CoA O-methyltransferase (EC:2.1.1.104); CCR, cinnamoyl CoA-reductase (EC:1.2.1.44); HCT, hydroxycinnamoyl-CoA: shikimate/guinate hydroxycinnamoyltransferase (EC:2.3.1.133); CAD, cinnamyl alcohol dehydrogenase (EC:1.1.1.195); LAC, laccase (EC:1.10.3.2); PER, lignin peroxydase (EC:1.11.1.7); PLR, pinoresinol/lariciresinol reductase; DP, dirigent protein; STS, stilbene synthase; CHS, chalcone synthase (EC:2.3.1.74); CHI, chalcone isomerase (EC:5.5.1.6); FNS, flavone synthase (EC:1.14.11.22); F3H, flavanone 3-hydroxylase (EC:1.14.11.9); F3′H, flavonoid 3′-hydroxylase (EC:1.14.13.21); F3′5′H, flavonoid 3′5′-hydroxylase (EC:1.14.13.88); FLS, flavonol synthase (EC:1.14.11.23); DFR, dihydroflavanol 4-reductase (EC:1.1.1.219); ANS (LDOX), anthocyanidin synthase (EC:1.14.11.19); UF3GT, UDP glucose flavonoid 3-O-glucosyltransferase (EC:2.4.1.115); 3RT, anthocyanidin-3-glucoside rhamnosyl transferase (EC:2.4.1.-); AAT, anthocyanin 5-aromatic acyltransferase (EC:2.3.1.153); AMT, anthocyanin methyltransferase; GST, glutathione S-transferase (EC:2.5.1.18); VT, vacuolar transporter; LAR, leucoanthocyanidin reductase (EC:1.17.1.3); ANR, anthocyanidin 4-reductase (1.3.1.77); GT, glucosyltransferase; MATE, multidrug and toxic compound extrusion; CE, condensed enzymes; PPO, polyphenol oxidase (EC:1.10.3.1); CHR, chalcone reductase (EC:1.1.1.-); IFS, 2-hydroxyisoflavanone synthase (EC:1.14.13.86); HIDH, 2-hydroxyisoflavanone dehydratase (EC:4.2.1.105); 7IOMT, isoflavone 7-O-methyltransferase (EC:2.1.1.150); I2'H, isoflavone 2′-hydroxylase (EC:1.14.13.89); IFR, isoflavanone reductase (EC:1.3.1.45); F6H, flavonoid 6-hydroxylase (EC:1.14.13.-); HI4′OMT, isoflavone 4-O-methyltransferase (EC:2.1.1.46); IF7MaT, isoflavone 7-O-methyltransferase (EC:2.3.1.115); VR, vestitone reductase (EC:1.1.1.-); DMID, dihydroxy-methoxy-isoflavanol dehydratase; G4DT, pterocarpan 4-dimethylallyltransferase; PTS, pterocarpan synthase (EC:1.1.1.246); KAP2, H-box-binding TF (CHS); Myb4, R2R3-type Myb TF (PAL); LIM1, TF LIM1 domain protein (lignin); HD-GL2, TF homeodomain protein GL2 like 1/ANL2; Myb15, R2R3-type Myb TF; WIP, TF zinc finger WIP (TT1); TGA1, basic domain/leucine zipper TF. Common bean genes cloned in this study are shown in bold red.

In this study, we amplified and sequenced fragments of 46 genes coding for structural and/or regulatory proteins of phenylpropanoid pathway in common bean (designated with red lettering in Figure 1).

***Structural gene fragments.***

*General phenylpropanoid pathway.* Six cDNA fragments for structural genes coding for enzymes of the general phenylpropanoid pathway (PAL, C4H, and 4CL) were cloned and sequenced in common bean (Table 2). In all cases significant BLAST scores (≥E^−10^) were detected. The PCR fragments ranged in sizes from 300 bp (PAL2) to 550 bp (4CL1). Gene fragments for two 4-coumarate CoA ligase (*4CL1* and *4CL2*) were cloned in this study using *G. max 4CL1* (Accession AF279267) and *4CL2* (Accession AF002259), respectively (Lindermayr et al., 2002) as source sequences (Table 2). Additional copies of *4CL* gene need to be isolated in the future. In addition, complete *PAL2* (*PAL2-Z*) and *PAL3* (*PAL3-Z*) gene sequences were determined (Table 3).

**Table 2:** **Cloned phenylpropanoid pathway gene fragments in common bean**.

**Enzyme**	**Gene**	**PCR primers**			**Common bean clone**		
		**Source**	**Position (bp)**	**Sequence (5′-3′)**	**Size (bp)**	**BLASTn *E*-value**	**GenBank accession**
**GENERAL PHENYLPROPANOID PATHWAY**							
Phenylalanine ammonia lyase	*PAL1*	*P. vulgaris*	1060–1628	GGATCTGCTGAAAGTGGTTGACA	486	0.0	CV670758
		M11939		TCCGGCTTTCCATGTAGATTTG	506		CV670759
	*PAL2*	*P. vulgaris*	1612–3729	CCCACCGCCGTACCAAACAA	399	5E-24	CV670734
		P19142		GCAACTCAAAGAACTCAGAACCAA			
	*PAL3*	*P. vulgaris*	630–1373	GTGCAAACTCCAACAGGCAAA	298	1E-49	CV670735
		P19143		GCAGCAATGTAGGACAGAGGAA			
Cinnamate 4-hydroxylase	*C4H*	*P. vulgaris*	716–1074	GTTCATTCAGGCCACGAGGTT	359	0.0	CV670736
		Y09447		CCAACTCAGCTATTGCCCATT			
4-coumarate: CoA ligase	*4CL1*	*G. max*	903–1447	GCGTTGGTTAAGAGCGGAGAGA	545	0.0	CV670737
		AF279267		CCTACAACGGCAGCATCAGAAA			
	*4CL2*	*G. max*	870–1271	CTCCTTGCTTGCTCTCATTCA	402	5E-64	CV670738
		AF002259		CCTTTCATAATCTGGTCGCCTCT			
**LIGNIN/LIGNAN BRANCH**							
Cinnamoyl CoA reductase	*CCR*	*G. max*	129–575	CTGGTGGCTTCATCGCCTCT	338	E-130	CV670739
		BI426824		CAGCCTTCCCATAGCAATACCAA			
Cinnamyl alcohol dehydrogenase	*CAD1*	*M. sativa*	855–1160	GACACAATATCTGCTGCTCATT	306	1E-54	CV670740
		L46856		GGCCACATCAATCACAAACCGAT			
4-coumarate-3 hydroxylase	*C3H*	*S. indicum*	325–871	GACTTGATTTGGTCCGATTATGG	386	2E-97	CV670754
		AY065995		CGATAACGGTGTCATCACTGAG	468	E-147	CV670755
Caffeate O-methyltransferase	*COMT*	*M. sativa*	388–779	GATGGTGTATCCATTTCTGCTCTT	401	6E-98	CV670741
		M63853		CCACCAACATGCTCAACTCCT			
Ferulate 5-hydroxylase	*F5H*	*G. max*	67–534	AGCGGGTCCAACAAGAGCTG	468	E-135	CV670742
		BM527849		CGAACACGTCACCCATGTCC			
Laccase	*LAC*	*G. max*	2937–3980	CCACTCCCTGCTTACAACGACA	489	E-142	CV670743
		AF527604		CCCGAAACCCTCTGCAACAA			
Lignin peroxidase (PER)	*FBP1*	*P. vulgaris*	224–982	CGATAGTGAGCGAGCAAGAAG	513	9E-63	CW652094
		AF149277		CCAAAGTAGCCAATCCAGCAGA			
	*FBP4*	*P. vulgaris*	133–827	TGACGGTTCTGTTCTCATTTCC	496	0.0	CW652095
		AF149279		TGCCCATCTTCACCATAGCATTT	495	0.0	CW652096
**FLAVONOID/ANTHOCYANIN BRANCH**							
Chalcone synthase	*CHS*	*P. vulgaris*	194–971	GTGAGCACATGACCGACCTCA	585	0.0	CV670744
		X06411		CCAGGGTGTGCAATCCAGAA			
Chalcone isomerase	*CHI*	*P. vulgaris*	950–1826	CGTCACTCGCCACCAAGTGGAA	215	E-176	CV670745
		Z15046		GCCTTTGGCTTCAGCATCACCAT			
Chalcone reductase	*CHR*	*G. max*	52–489	TGCCTTTGAGGTTGGCTACAGA	579	6E-55	CW652099
		BU578179		ACAGCAGGAGGGATGGTTGC	465	2E-78	CW652100
Rhamnosyl transferase	*3RT*	*P. coccineus*	53–303	TGGGTGTTGAGGGTTCATCG	251	2E-79	CV670748
		CA914524		CACTCGCGCATTTATCCCTTG			
Flavanone 3-hydroxylase	*F3H*	*M. sativa*	2119–2856	GCAAGAAGATTGTGGAGGCTT	590	E-119	CW652103
		X78994		CCTGGATCTGTGTGGCGTTT			
Flavonoid 3′-hydroxylase	*F3′H*	*G. max*	242–1082	GGCTTCGTCGATGTCGTGGT	486	E-128	CV670760
		AB061212		GGTGGGCCAAGTCCTCTTCTTT	508	E-139	CV670761
Anthocyanin 5-O-glucosyltransferase	*UF5GT*	*P. coccineus*	6–340	CCAAGGCGTAATCCAAGTCCA	221	E-103	CV670749
		CA914529		GGGTTTCAGGCTCTCCATGA			
Anthocyanidin reductase	*ANR*	*M. truncatula*	369–778	GTGTTGAAAGCATGTGTAAGAGCA	576	5E-80	CW652104
		AY184243		GCCCGACAAATATCCTCGACAT	590	6E-80	CW652105
Anthocyanin 5-acyltransferase	*AAT*	*P. coccineus*	166–572	GGGTGTCTCTTCACCACTCCA	407	0.0	CV670746
		CA900148		GAAATGGCGGCGAAAGTGTT			
Glutathione S-transferase	*GST1*	*P. acutifolius*	202–802	GCTTCTCAAATACAACCCAGTTCA	487	2E-85	CW652097
				GAAACGCAATGGTAACTAACAGCA	502	0.0	CW652098
	*GST2*	AY220095			509	E-179	CV670756
					498	0.0	CV670757
Vacuolar transporter	*VT*	*P. coccineus*	3–455	TGGAAGTGGATGGCAAGCAG	451	0.0	CV670747
		CA907034		TCACATACTTGTGCTGACAGTGAA			
**ISOFLAVONOID BRANCH**							
2-hydroxyisoflavanone synthase	*IFS*	*G. max*	573–1325	GGCGAGGCTGAGGAGATCAGA	507	E-139	CV670762
		AF195818		CTGGGAGTGGTGGGTGCATT	506	E-154	CV670763
Isoflavanone reductase	*IFR*	*M. sativa*	906–1581	GGAAGACACATAGTTTGGGCAAGT	268	E-139	CV670750
		U17436		CGGTAAAGGCGTGGCAACAA			
7-O-methyltransferase	*IOMT*	*M. sativa*	234–637	GGTAACGTGCGGCGTCTCAT	456	0.0	CV670751
		AF000975		GCAGTTGTTCCAGTTCCACCA			
**REGULATORY GENES**							
KAP-2 (CHS)	*KAP2*	*P. vulgaris*	504–1007	GAAGGCACAAAGGAAGAGCAA	504	0.0	CV670752
		AF293344		CCAGTGAGAAGTGGATAAGGGAA			
Myb AtMyb4 (PAL)	*Myb4*	*P. coccineus*	92–406	AAGATGGGAAGAGCTCCTTGTT	544	4E-64	CW652106
		CA902489		ATCTCATTATCTGTTCGTCCTGGT			
Homeodomain protein GL2 like1/ANL2	*HD*	*P. coccineus*	3–326	GTCGGTGGAGACCGTGAACA	324	8E-95	CV670753
		CA902455		CGAACCACCACCCTTGCCTA			
ZINC finger WIP (TT1)	*WIP*	*P. coccineus*	2–496	GGGCCTCATTCACATGCAA	534	4E-70	CW652101
		CA902512		CGTGGGTGGTCGATGTTGTT			
LIM domain protein WLIM1 (lignin)	*LIM*	*G. max*	27–569	GGCATTTGCAGGAACAACTCAG	458	0.0	CW652092
		BU764417		GCACTCTTCTCATGGTCACCTTC	489	0.0	CW652093
Myb AtMYB15	*Myb15*	*P. coccineus*	39–452	GAGTGATGGCGTGCGGAGTA	519	E-162	CW652102
		CA902486		CCCAGCGTCTCATGCAGCTT			

**Table 3:** **G19833 BAC clones containing common bean phenylpropanoid pathway genes**.

**BAC clone**	**Pv v.1.0 assembly (*in silico* chromosome position, nt)**	**Gene**	**Size (bp)**
PV-GBa 0005G03^a^	Chr2:3,691,740..3,830,946	*CHS-B-Z*^b^	1265
PV-GBa 0083H05	Chr1:14,526,848..14,805,105	*CHS-A-Z*	1700
PV-GBa 0072I22	Chr7:48,408,886..48,606,955	*DFR-Z*	2793
PV-GBa 0043K12	Chr7:13,377,553..13,576,830	*Myb15-Z*	1781
PV-GBa 0079P22	Chr7:36,990,886..37,135,402	*PAL2-Z*	3830
PV-GBa 0061D18	Chr8:59,298,741..59,462,821	*PAL3-Z*	2573

a Clemson University Genomics Institute (CUGI, http://www.genome.clemson.edu/).

b Copy number = 8.

*Lignin/lignan branch.* PCR fragments were amplified from common bean cDNA or genomic DNA with PCR primers designed on the basis of sequence information from related legume species (such as *Medicago sativa*) or very different plant species (including *Sesamum indicum* or *Forsythia intermedia*) for all of the major enzymes in this branch, including: cinnamoyl CoA reductase (CCR), cinnamyl alcohol dehydrogenase (CAD), 4-coumarate-3 hydroxylase (C3H), caffeate O-methyltransferase (COMT), ferulate 5-hydroxylase (F5H), laccase (LAC) and two peroxidases (PER). Significant BLAST scores with the expected gene sequences were detected for most of the cloned common bean sequences (Table 2). However, the sequences of the fragments amplified with the caffeic acid CoA 3-O-methyltransferase (CCOMT) and pinoresinol/lariciresinol reductase (PLR) primers did not have significant similarity with the authentic sequences according to their BLAST scores (data not shown). These genes will need to be isolated in future studies.

*Flavonoid/anthocyanin branch.* EST sequences from *Phaseolus coccineus* and *P. acutifolius* as well as sequences from other legumes were used to design primers for amplifying PCR fragments from common bean cDNA or genomic DNA templates of a number of enzymes in this important branch, including: chalcone synthase (CHS), chalcone isomerase (CHI), chalcone reductase (CHR), flavanone 3-hydroxylase (F3H), flavonoid 3′-hydroxylase (F3′H), anthocyanidin 4-reductase (ANR), anthocyanin 5-aromatic acyltransferase (AAT), anthocyanin rhamnosyl transferase (3RT), UDP-g1ucose:flavonoid 5-O-glucosyltransferase (UF5GT), glutathione S-transferase (GST), and vacuolar transporter (VT) (Table 2). In addition, the complete gene sequences were obtained for two common bean *CHS* (CHS-A-Z and CHS-B-Z) and *DFR* (DFR-Z) (Table 3). Three *DFR* paralogous genes were cloned and sequenced (white common bean cultivar OAC Rex). The predicted common bean DFR1 (DFR1-M) protein was similar to soybean DFR3 (61%), common bean DFR2 (DFR2-M) protein was similar to soybean DFR1 (87%) and common bean DFR3 (DFR3-M) protein was similar to soybean DFR2 (86%). Although a good coverage of this segment of the phenylpropanoid pathway in common bean was obtained in the current work, gene fragments for several enzymes including flavonol synthase (FLS), flavone synthase (FNS), flavonoid 3′5′-hydroxylase (F3′5′H), anthocyanidin synthase (ANS) and leucoanthocyanidin reductase (LAR) need to be isolated in the future. Attempts to amplify fragments of these genes with cDNA or genomic DNA templates failed (data not shown).

*Isoflavonoid branch.* Prior to this study, there was no sequence information in common bean for the genes coding for the enzymes of this branch of the phenylpropanoid pathway, except a *P. coccineus* EST corresponding to isoflavone reductase-like (IFR-l). Using this information and sequence information from the other legumes, fragments for three structural genes coding for 2-hydroxyisoflavanone synthase (IFS), isoflavanone reductase (IFR), and 7-O-methyltransferase (7IOMT) were isolated (Table 2). There is still a need to obtain sequences for genes coding for the remaining enzymes [including isoflavone 2′-hydroxylase (I2′H), vestitone reductase (VR) and dihydroxy-methoxy-isoflavanol dehydratase (DMID)] of this branch of the phenylpronaoid pathway in common bean.

***Regulatory gene fragments.*** At the beginning of this study only two *P. vulgaris* gene sequences for regulatory genes of the phenylpropanoid pathway, *KAP2* (Lindsay et al., 2002) and bZIP *TGA1* (Tucker et al., 2002), were available. By utilizing *P. coccineus* ESTs and sequence information from a variety of plant species it was possible to design primers to amplify fragments of additional transcription factors from common bean cDNA and genomic DNA templates. Initially, 18 different transcription factor sequences were targeted. However, BLAST searches with the sequences of the fragments that were amplified indicated that only six genes encoding common bean transcription factors (Myb4, LIM, KAP-2, HD, WIP, and Myb15) were identified as putative (or possible) regulatory genes of this pathway (Table 2). In addition, the whole gene sequence was isolated for Myb15 (Table 3) and two gene fragments for TT2 transcription factor (similar to LjTT2 a, b, and c) were amplified and sequenced (data not shown).

### Sequence-based comparative mapping of phenylpropanoid pathway genes in common bean and soybean

#### Common bean and soybean phenylpropanoid pathway gene-based sequence maps

We developed *in silico* phenylproanoid pathway gene sequence-based maps by mapping phenylpropanoid pathway gene sequences to common bean and soybean chromosomes (Tables S1, S2). Over 600 gene and marker sequences were used in development of *in silico* maps. As expected, the majority of gene/marker sequences mapped to one chromosome in common bean but two chromosomes in soybean. This resulted in mapping approximately two times as many sequence features in soybean genome (1011) compared to common bean (507). The current *in silico* maps contain sequences of phenylpropanoid pathway genes (260 in common bean and 459 in soybean) and common bean and soybean marker sequences (seed coat color gene-based markers, g markers and SSRs).

***Common bean sequence map.*** The common bean *in silico* sequence map contains 507 features on 11 chromosomes (Tables 4, S1), ranging from 25 (chromosomes Pv4, Pv10, and Pv11) to 83 (chromosome Pv2).

**Table 4:** **Characteristics of common bean phenylpropanoid pathway gene-based sequence map**.

**Chromosome**	**Total features**	**Position (nucleotide number)**		**Coverage (bp)**	**Size (bp)**^**a**^
		**Start**	**End**		
Pv1	56	1,071,371	52,150,964	51,079,593	52,205,531
Pv2	83	238,724	48,041,224	47,802,500	49,040,938
Pv3	54	950,004	51,791,104	50,841,100	52,284,309
Pv4	25	1,428,273	45,736,396	44,308,123	45,960,019
Pv5	26	2,347,029	40,478,924	38,131,895	40,819,286
Pv6	51	9,243,136	31,761,604	22,518,468	31,977,256
Pv7	51	599,228	50,145,180	49,545,952	51,758,522
Pv8	69	155,567	59,310,664	59,155,097	59,662,532
Pv9	40	5,750,860	36,534,776	30,783,916	37,469,608
Pv10	25	2,032,367	43,002,236	40,969,867	43,275,151
Pv11	25	169,275	49,713,356	49,544,081	50,367,376
Total	507				514,820,528

a Phytozome (www.phytozome.net/commonbean.php)—Accessed: February 13, 2013.

***Soybean sequence map.*** The soybean *in silico* sequence map contains 1011 features on 20 chromosomes (Tables 5, S2), ranging from 31 (chromosomes Gm16) to 100 (chromosome Gm08).

**Table 5:** **Characteristics of soybean phenylpropanoid pathway gene-based sequence map**.

**Chromosome**	**Total features**	Position (nucleotide number)		**Coverage (bp)**	**Size (bp)^a^**
		**Start**	**End**		
Gm01	33	992,725	55,451,076	54,458,351	55,915,595
Gm02	59	19,669	51,366,328	51,346,659	51,656,713
Gm03	33	1,606,119	46,881,240	45,275,121	47,781,076
Gm04	35	189,069	48,284,344	48,095,275	49,243,852
Gm05	46	242,329	41,919,824	41,677,495	41,936,504
Gm06	52	229,125	50,276,300	50,047,175	50,722,821
Gm07	54	305,682	42,294,768	41,989,086	44,683,157
Gm08	100	329,749	46,669,032	46,339,283	46,995,532
Gm09	65	349,423	45,986,536	45,637,113	46,843,750
Gm10	47	648,359	50,652,976	50,004,617	50,969,635
Gm11	52	305,133	39,002,116	38,696,983	39,172,790
Gm12	34	136,883	39,748,568	39,611,685	40,113,140
Gm13	74	798,836	44,256,628	43,457,792	44,408,971
Gm14	48	402,041	48,273,860	47,871,819	49,711,204
Gm15	55	190,218	50,089,752	49,899,534	50,939,160
Gm16	31	493,954	37,180,576	36,686,622	37,397,385
Gm17	44	542,091	40,950,824	40,408,733	41,906,774
Gm18	71	31,200	62,243,640	62,212,440	62,308,140
Gm19	41	740,074	50,265,556	49,525,482	50,589,441
Gm20	37	1,123,688	46,134,760	45,011,072	46,773,167
Total	1,011				950,068,807

a Phytozome (www.phytozome.net/soybean.php)—Accessed: February, 2013.

#### Sequence-based comparative mapping of phenylpropanoid pathway genes in common bean and soybean

**Figure 2:**
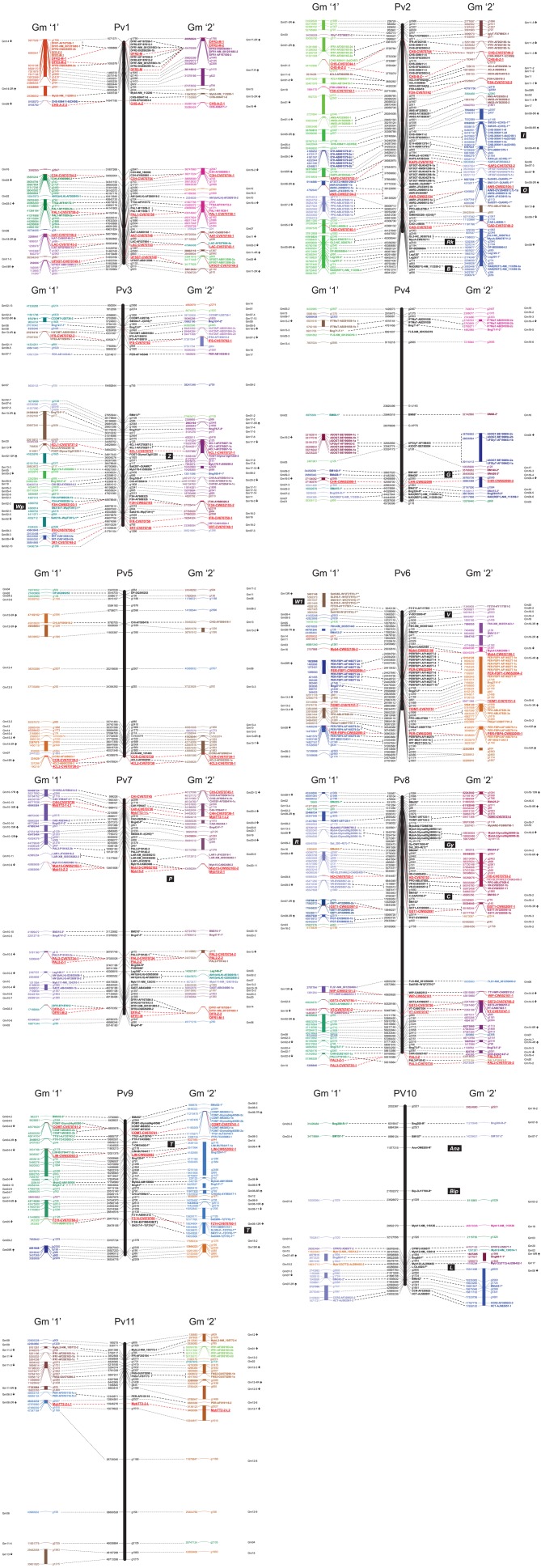
**Phenylpropanoid pathway gene sequence-based common bean-soybean comparative map**. Marker and phenylpropanoid pathway gene names (abbreviated plus accession number) are shown on right of each chromosome. Feature position [bp, start nt shown (Phytozome)] is indicated on left of each chromosome. Syntenic fragments of soybean chromosomes (each in different color) are shown on left (copy “1”) and right (copy “2”) of common bean chromosome (black). Phenylpropanoid pathway gene fragments cloned in this study are shown in red, bold, and underlined. Tentative positions of seed coat color genes are shown in black blocks. Enzyme abbreviations: PAL, phenylalanine ammonia lyase; C4H, cinnamate 4-hydroxylase; 4CL, 4-coumarate:CoA ligase; C3H, 4-coumarate 3-hydroxylase; COMT, caffeate O-methyltransferase; F5H, ferulic acid 5-hydroxylase; CCoOMT, caffeoyl CoA O-methyltransferase; CCR, cinnamoyl CoA-reductase; HCT, hydroxycinnamoyl-CoA: shikimate/guinate hydroxycinnamoyltransferase; CAD, cinnamyl alcohol dehydrogenase; LAC, laccase; PER, lignin peroxydase; PLR, pinoresinol/lariciresinol reductase; DP, dirigent protein; STS, stilbene synthase; CHS, chalcone synthase; CHI, chalcone isomerase; FNS, flavone synthase; F3H, flavanone 3-hydroxylase; F3′H, flavonoid 3′-hydroxylase; F3′5′H, flavonoid 3′5′-hydroxylase; FLS, flavonol synthase; DFR, dihydroflavanol 4-reductase; ANS (LDOX), anthocyanidin synthase; UF3GT, UDP glucose flavonoid 3-O-glucosyltransferase; 3RT, anthocyanidin-3-glucoside rhamnosyl transferase; AAT, anthocyanin 5-aromatic acyltransferase; AMT, anthocyanin methyltransferase; GST, glutathione S-transferase; VT, vacuolar transporter; LAR, leucoanthocyanidin reductase; ANR, anthocyanidin 4-reductase; PPO, polyphenol oxidase; CHR, chalcone reductase; IFS, 2-hydroxyisoflavanone synthase; HIDH, 2-hydroxyisoflavanone dehydratase; 7IOMT, isoflavone 7-O-methyltransferase; I2′H, isoflavone 2′-hydroxylase; IFR, isoflavanone reductase; F6H, flavonoid 6-hydroxylase; HI4′OMT, isoflavone 4-O-methyltransferase; IF7MaT, isoflavone 7-O-methyltransferase; VR, vestitone reductase; DMID, dihydroxy-methoxy-isoflavanol dehydratase; G4DT, pterocarpan 4-dimethylallyltransferase; PTS, pterocarpan synthase.

The comparative map (Figure 2) was developed *in silico* by aligning sections of related soybean chromosomes to common bean chromosomes, based on the sequence positions of common bean g markers and phenylpropanoid pathway genes that were shared by the two species. The correspondence between common bean and soybean chromosomes agrees to a large extent with previously reported patterns of shared synteny between the two species (McClean et al., 2010; Galeano et al., 2011). Additional sequence features/regions shared between the two genomes were also identified. In particular, we focussed on identifying the positions of many of the phenylpropanoid pathway genes in common bean and their syntenic regions on corresponding soybean chromosome blocks. The positions of dihydroflavonol 4-reductase (DFR) on common bean chromosome Pv1 between markers g1795 and g1645 and chalcone isomerse (CHI_INT3) between markers g503 and g2298 were reported previously (McClean et al., 2010) but their corresponding locations in soybean genome were not identified. In addition, we *in silico* mapped the sequences of several common bean and/or soybean markers associated with seed coat color in both species (Figure 2). Also, several common bean seed coat color and pattern genes were *in silico* positioned on the common bean-soybean sequence-based comparative map according to their positions on the common bean genetic maps (McClean et al., 2002) and syntenic locations in the soybean genome (summarized in Table 6).

**Table 6:** **Genome location of common bean seed coat color and pattern genes**.

**Common bean**						**Soybean synteny**	
**Gene**		**Phenotype**	**Genome location**				
			**Chromosome**	**Closest marker(s)**	**Nearest phenylpropanoid pathway gene(s)**		
Color	*P*	Ground factor	Pv7	g487-BM210	Myb15-CW652102/PAL2-P19142-1	Gm20/Gm13	Gm10
	*C*	Complete color	Pv8	C-OAP2_700_-F^a^	HD-CV670753; VR-EU925557/GST1-AY220095	Gm18	Gm07
	*J* (=*L*)	Mature color development	NA	NA^b^	NA	NA	NA
	*G*	Yellow brown	Pv4	BM140-g2595	A3OGT-BE190894/CHR-CW652099	Gm09	Gm16
	*B*	Gray brown	Pv2	g364-g656	NAD(REF1)-NM_113359-2/-	Gm08	Gm05
	*V*	Violet	Pv6	V-OD12_800_-R^a^	F3′5′-AY117551/F6H-XM_003551443	Gm18	Gm13 *W1*-F3′5′H
	*Rk*	No color expression	Pv2	g2540-g1194	CAD-CV670740/MycA-EW678711/DP-BE806864	Gm08 *I*—CHS, *O*—ANR	Gm05
Pattern	*T*	Totally colored	Pv9	T-OM19_400_-F^a^	COMT-CV670741; PTR-TC433083/LIM-BU764417	Gm06 *T*-F3′H	Gm04
	*Z* (=*D*)	Zonal	Pv3	g125-g1925	4CL1-AF279267-1; FOGT-Glyma13g01220/Myb176-FJ550209	Gm17 Gm02-*Wp*-F3H	Gm13/Gm05
	*Bip*	Bipunctata	Pv10	Bip-OJ17_700_-R^a^	Myb11-NM_116126/-	Gm03/Gm19	Gm07
	*Ana*	Anasazi	Pv10	Ana-OM9_200_-R^a^	–	Gm07	Gm03
	*Gy*	Greenish-yellow	Pv8	Gy-OW17_600_-R^a^	MybA-Glyma09g36990-1b/HD-CA902455	Gm18	Gm9 *R*

a Markers associated with the seed coat color/pattern genes (McClean et al., 2002).

b not available.

***Pv1.*** We confirmed three large syntenic blocks identified previously (McClean et al., 2010; Galeano et al., 2011) between common bean chromosome Pv1 and six soybean chromosomes, including with: Gm14 (17 features)/Gm17 (12 features), Gm03 (17 features)/Gm19 (18 features), and Gm18 (12 features)/Gm11 (8 features). In addition, one to four sequences were shared between Pv1 and several (nine) soybean chromosomes (Figure 2). Eleven phenylpropanoid gene sequences mapped to the common bean chromosome Pv1, including eight cloned in this study (*DFR2-M, DFR3-M, CHS-A-Z, C3H*-CV670754, *PAL1*-CV670758, *AAT*-CV670746, *LAC*-CV670743, and *UF5GT*-CV670749, shown in red in Figure 2). *DFR2* (sequence start 1,086,866 nt) and *DFR3* (sequence start 1,093,579 nt), located in the first block, are syntenic to soybean *DFR* genes on chromosomes Gm14 and Gm17, respectively. *C3H* (33,062,004 nt) and *PAL1* (44,150,744 nt) found on the second block were syntenic to soybean genes on chromosomes Gm03 and Gm19, respectively. The third syntenic block contains sequences of *AAT* (50,073,684 nt), *LAC* (50,563,496), and *UF5GT* (51,855,780). The first copy of these genes was found on syntenic soybean chromosome Gm18 (in reverse order) but the second copy of these genes was located on three separate chromosomes [syntenic Gm11 and two other chromosomes (Gm02 and Gm05, respectively)].

***Pv2.*** This study confirmed the existence of two major syntenic blocks between common bean chromosome Pv2 and soybean chromosomes Gm01 (22 features)/Gm11 (22 features) and Gm05 (27 features)/Gm08 (43 features), that were identified previously (McClean et al., 2010; Galeano et al., 2011). In addition, one to 12 sequences were shared between Pv2 and eight soybean chromosomes (Figure 2). Twenty phenylpropanoid pathway gene sequences were found on chromosome Pv2, six of them cloned in this study (*CHS*-CV670744, *CHS-B-Z, F5H*-CV670742, *KAP2*-CV670752, *ANR*-CW652104, and *CAD*-CV670740). A cluster containing four *CHS* genes was found on the common bean chromosome Pv2 (at 3,726,784 nt; 3,733,338 nt; 3,765,494 nt; 3,775,079 nt). The first gene was syntenic to soybean *CHS7* on chromosome Gm01 and *CHS8* on chromosome Gm11. The fifth common bean *CHS* gene was found on position 33,749,564 nt (sequence start) and was syntenic to a single soybean *CHS* on chromosome Gm05 (*CHS2*) but corresponds to a cluster of six *CHS* genes on chromosome Gm08. The locations of the seed coat color genes *Rk* and *B* that were mapped previously to the end of chromosome Pv2, are indicated (McClean et al., 2002). Interestingly, soybean seed coat color genes *I* [factor which inhibits pigment formation in the seed coat, associated with six *CHS* repeats (Tuteja et al., 2004)] and *O* [factor for dull brown seed coat color, may be associated with anthocyanin reductase (ANR, Yang et al., 2010)] were located on chromosome Gm08, which are syntenic to the regions of Pv2 chromosome containing common bean *CHS* and *ANR*, respectively.

***Pv3.*** Blocks of soybean chromosomes Gm02 (16 sequence features) and Gm05 (eight features)/Gm17 (21 features) and Gm02/Gm16 (six features) were syntenic to common bean chromosome Pv3 (McClean et al., 2010; Galeano et al., 2011). In addition, one to 11 sequences were shared between Pv3 and many (11) soybean chromosomes (Figure 2). Eleven phenylpropanoid pathway genes were identified on chromosome Pv3, including five cloned in this study (*IFS*-CV670762, *4CL1*-CV670737, *F3H*-CW652103, *IFR*-CV670750, and *3RT*-CV670748). Two copies of the *4CL1* gene were found 13,788 bp apart on the common bean chromosome Pv3 (*4CL1-2* at the start position 34,628,464 nt and *4CL1-1* at the start position 34,642,252 nt, respectively). Soybean seed coat color gene *Wp*, (purple flower and hypocotyl color and the production of black pigment in the seed coat) associated previously with flavanone 3-hydroxylase (F3H) on chromosome Gm02 (Zabala and Vodkin, 2005), was mapped to the expected region that was syntenic to common bean *F3H*. Previously, RAPD marker OAM10 was associated with common bean seed coat pattern gene *Z* (zonal) and was positioned between markers Bng12 and Bng165 on chromosome Pv3 (McClean et al., 2002). However, in the current study there were no significant BLAST hits with sequence of this marker and the *Z* gene was tentatively positioned in the region between the closest SSRs, Bng75 and Bng165.

***Pv4.*** Blocks of soybean chromosomes Gm13 (five features)/Gm19 (eight features) were syntenic with common bean chromosome Pv4, as detected previously (McClean et al., 2010). A new syntenic block was identified with soybean chromosomes Gm16 (12 features) and Gm09 (13 sequence features). In addition, one to four sequences were shared between Pv4 and several (seven) additional soybean chromosomes (Figure 2). Six phenylpropanoid pathway genes were identified on common bean chromosome Pv4, including chalcone reductase (*CHR*-CW652099) cloned in this study. There were no significant BLAST hits with markers OU14, OAP3, and OAP14 (McClean et al., 2002) associated with the common bean seed coat gene *G* (yellow seed coat, from *Gelbe* in German) and the gene was tentatively positioned in the region between the closest SSRs, BM68, and Bng224.

***Pv5.*** Blocks of soybean chromosomes Gm15 (13 features)/Gm13 (16 sequence features) and Gm12 (seven features)/GmGm13 were syntenic with common bean chromosome Pv5, as identified previously (McClean et al., 2010). In addition, one to two sequences were shared between Pv5 and several (seven) soybean chromosomes, including Gm08 (two features) previously identified as a syntenic block (Gm08/Gm 12, McClean et al., 2010) (Figure 2). Four phenylpropanoid pathway genes were identified on common bean chromosome Pv5, two cloned in this study (*CCR*-CV670739 at the start position 40,358,576 nt and *4CL2*-CV670738 at the position 40,478,924 nt). *CCR* and *4CL2* genes were also identified on syntenic soybean chromosomes Gm15 and Gm13, respectively positioned in the reverse orientation at the chromosome ends.

***Pv6.*** Blocks of soybean chromosomes Gm08 (15 sequence features)/Gm18 (nine features), Gm13 (10 features)/Gm15, Gm08/Gm15, Gm15/Gm09 (11 features), and Gm19 (three features) and Gm12 (five features) were syntenic with common bean chromosome Pv6, as identified previously (McClean et al., 2010). In addition, one to two sequences were shared between Pv6 and six additional soybean chromosomes (Figure 2). Nine phenylpropanoid pathway genes were found on the common bean chromosome Pv6, including four cloned in this study (*Myb4*-CW652106, *PER-FBP1*-CW652094, *7IOMT*-CV670751, and *PER-FBP4*-CW652095). Two copies of the common bean transcription factor gene *Myb4* (23,032,616 nt) were identified on soybean chromosomes Gm19 and Gm16, respectively. A cluster of 10 PERs (*FBP1*-AF149277) found at the positions 24,384,794 nt to 24,475,020 nt was syntenic to six PERs on the soybean chromosome Gm15 and six genes on Gm09, both clusters in reverse order. Another example is a single PER gene (*FBP4*-AF149279) on the common bean chromosome Pv6 (30,963,524 nt) associated with two clusters of four soybean peroxidases on the chromosomes Gm15 and three genes on the chromosome Gm08. The sequence of the marker OD12 that was associated with the common bean seed coat color *V* (violet factor, changing white or pale lilac flowers into violet and causing violet to black seed coat colors) gene (McClean et al., 2002) mapped between *F3′5′H*-AY17551 and *F6H*-XM_003551443 at the top of the chromosome Pv6. In soybean, the seed coat color gene *W1* is associated with F3′5′H (Zabala and Vodkin, 2007). Several F3′5′H gene-based markers (Yang et al., 2010) mapped to the location of F3′5′H on the soybean chromosome Gm13. This map position was syntenic to the region of common bean *F3′5′H* gene located at the top of the chromosome Pv6.

***Pv7.*** Blocks of soybean chromosome Gm10 (39 sequence features)/Gm20 (25 features) and Gm10/Gm13 (six features) were syntenic with common bean chromosome Pv7, as identified previously (McClean et al., 2010). In addition, one to six sequences were shared between Pv7 and five soybean chromosomes (Figure 2). Thirteen phenylpropanoid pathway genes were identified on the common bean chromosome Pv7, including six cloned in this study (*CHI*-CV670745, *C4H*-CV670736, *TT2-1-L, Myb15*-CW652102/*Myb15-Z, PAL2*-CV670734/*PAL2-Z*, and *DFR-Z/DFR1-M*). Cinnamate 4-hydroxylase gene (*C4H*-J09447) on the common bean chromosome Pv7 was associated with one copy on soybean chromosome Gm20 and second on the chromosome Gm10. A BLAST search with the RAPD marker sequence OU3, which is tightly linked with the common bean seed coat color *P* gene (pigment ground factor) on chromosome Pv7 (Erdmann et al., 2002), had no significant hits in our search. The *P* gene was tentatively placed between two closest SSR markers, Bng42, and BM210.

***Pv8.*** Two large syntenic blocks were found between common bean chromosome Pv8 and soybean chromosome blocks from Gm18 (34 sequence features)/[Gm02 (18 features), Gm8 (10 features), Gm09 (17 features), Gm07 (6 features)], and Gm14 (19 features)/Gm02, as identified previously (McClean et al., 2010). In addition, one to six sequences were shared between Pv8 and eight other soybean chromosomes (Figure 2). Fifteen phenylpropanoid pathway genes were identified on the common bean chromosome Pv8, including six cloned in the current study (*HD*-CV670753, *GST1*-CW652097, *WIP*-CW652101, *GST2*-CV670756, *VT*-CV670747, and *PAL3*-CV670735/*PAL3-Z*). Most of these sequences were associated with two copies on two soybean chromosomes. The only exceptions were polyphenol oxidase (*PPO*-ABL67928) with a single sequence and isoflavonoid glucosyltransferase (*IFGT*-EV280635) with two sequences on chromosome Gm18. The sequence of RAPD marker OW17, which was associated with the common bean seed coat color gene *Gy* (greenish-yellow factor; McClean et al., 2002) was mapped at the top portion of the chromosome Pv8 close to a cluster of Myb transcription factors. Soybean SSR marker Sat_293, associated with the seed coat color *R* gene (Yang et al., 2010), was also mapped to this region of the chromosome Pv8 and to the syntenic region on the soybean chromosome Gm09. A second marker (OAP2; McClean et al., 2002) sequence, linked to seed coat color gene *C* (required for complete seed coat color) mapped between SSR markers BM165 and BM153, close to the glutathione S-transferase (GST-AY220095) sequence on the chromosome Pv8.

***Pv9.*** Common bean chromosome Pv9 was syntenic to sequence blocks of soybean chromosomes Gm04 (28 shared sequence features)/Gm06 (33 features), as identified previously (McClean et al., 2010). The region at the lower arm of common bean chromosome Pv9 containing six g markers was syntenic to regions in soybean chromosomes Gm09 and Gm15. In addition, one to two sequences were shared between Pv9 and four other soybean chromosomes (Figure 2). Eight phenylpropanoid pathway genes were identified on the common bean chromosome Pv9, including three cloned in this study (*COMT*-CV670741, *LIM*-CW652092, and *F3′H*-CV707760). Common bean chalcone isomerase (CHI-AY595417) positioned at 20,929,174 nt was associated with a single copy on the soybean chromosome Gm06 but was missing from duplicated region on the chromosome Gm04. The RAPD marker sequence OM19, linked to seed coat pattern *T* gene (McClean et al., 2002), mapped on the common bean chromosome Pv9 between pterocarpan reductase (*PTR*-TC433083) and LIM transcription factor (*LIM*-BU764417). Several soybean seed coat color *T* gene-based markers [associated with F3′H (Yang et al., 2010)] were mapped to the F3′H location on chromosome Gm06 and to the syntenic region on common bean chromosome Pv9.

***Pv10.*** Common bean chromosome Pv10 was syntenic to soybean chromosome blocks of Gm07 (16 shared sequence features)/Gm08 (nine features) and Gm16 (four features), as identified previously (McClean et al., 2010). In addition, one to four sequences were shared between chromosome Pv10 and seven soybean chromosomes (Figure 2). Six phenylpropanoid pathway gene sequences mapped to the common bean chromosome Pv10, none of which were cloned in the current study. Several transcription factors were found on the common bean chromosome Pv10. Interestingly, for common bean transcription factor Myb11-NM_116126 (29,502,170 nt) only one copy was found on the soybean chromosome Gm19. Sequences of RAPD markers associated with three seed coat pattern genes (McClean et al., 2002) were mapped on the chromosome Pv10. The markers OM9, linked with the gene *Ana*, and OJ17 linked with the *Bip* gene, mapped to the top arm of chromosome Pv10, between markers BM157 and g1029. Marker OL4, linked to the seed coat pattern *L* gene, mapped to the bottom arm of chromosome Pv10 close to the Myb123 transcription factor (*Myb123*-AJ2994452).

***Pv11.*** Common bean chromosome Pv11 was syntenic to sequence blocks of soybean chromosomes Gm11 (16 features)/Gm12 (20 features), as identified previously (McClean et al., 2010), and Gm06 (seven features)/Gm12. In addition, one to four sequences were shared between chromosome Pv11 and seven other soybean chromosomes (Figure 2). Five phenylpropanoid pathway genes were found on the common bean chromosome Pv11 including MybTT2 transcription factor (*TT2-2-L*) that was cloned in this study. Isoflavonoid reductase (*IFR*-AF20184) mapped on the common bean chromosome Pv11 (3,852,808 nt) was also associated with the clusters of three genes on the soybean chromosome Gm11 and four genes on the soybean chromosome Gm01.

### Potential uses of whole genome sequences: common bean and soybean examples

The availability of common bean and soybean whole genome sequences could have significant impact on genetics and breeding of these important legume crops. The phenylpropanoid pathway is associated with many agriculturally-important traits, including disease resistance, nodulation or seed coat color. The whole genome sequences could be used to develop gene-specific markers, study gene structure or identify genes underlying important QTL.

#### 4-coumarate:CoA-ligase (4CL): an enzyme associated with many traits

To illustrate potential application of common bean and soybean whole genome sequences, we selected 4-coumarate:CoA ligase (4CL), a key enzyme of general phenylpropanoid pathway, positioned at the branching point leading to biosynthesis of lignin/lignans, flavonoids/anthocyanins, isoflavonoids or stilbenes for a detailed analysis. Two common bean *4CL* gene fragments homologous to soybean genes *4CL1* and *4CL2*, respectively, were isolated in this study (Table 2) and *in silico* mapped in both common bean and soybean (Figure 2).

***Genome organization of 4CL genes in common bean and soybean.*** Using soybean *4CL4* (X69955) cDNA sequence as a query, 128 common bean and soybean 4CL homologous protein sequences were retrieved from the Phytozome. The alignment of 98 primary transcripts (CLC Genomics Workbench, data not shown) and Neighbour Joining analysis produced a phylogenetic tree revealing complex relationships among 33 common bean and 65 soybean sequences (Figure S1A). Based on the similarity with the four published soybean *4CL*s [Accessions AF279267 (*4CL1*), AF002259 (*4CL2*), AF002258 (*4CL3*), and X69955 (*4CL4*)], 21 sequences were selected and re-analyzed. All of the proteins belonged to the AFD_class_I superfamily (cl17068, adenylate forming domain, class I) of acyl- and aryl-CoA ligases. Proteins similar to soybean 4CL1 (Glyma17g07190.2) and 4CL2 (Glyma13g44950.1) formed two separate clusters, while soybean 4CL3 (Glyma11g01230.1) and 4CL4 (Glyma01g44270.1) grouped together (Figure S1B).

**Figure 3:**
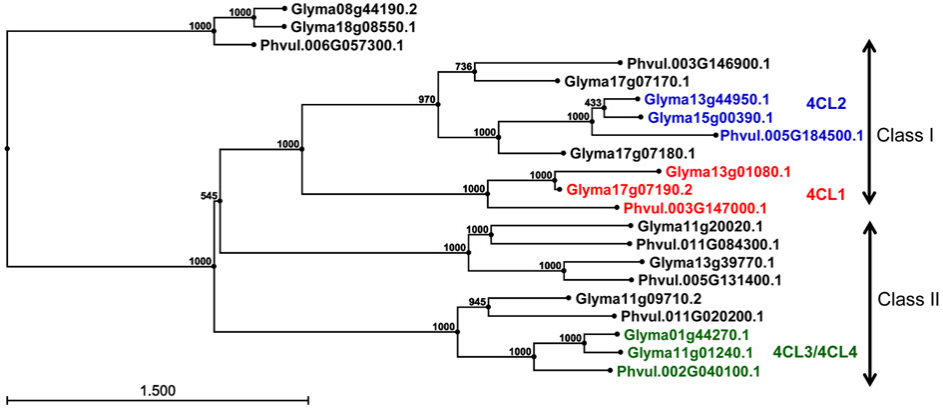
**Phylogenetic analysis of *4CL* genes in common bean and soybean**. Phylogenetic tree was constructed from *4CL* genomic sequences using Neigbor Joining (NJ) algorithm and 1000 bootstrap replications in CLC Genomics Workbench 3. Scale represents genetic distance and node numbers indicate bootstrap support values.

The genomic sequences were separated into two groups (Figure 3). The larger group consisted of 18 sequences and was divided into three clusters. The first cluster was further divided into two subclusters and contained class I 4CLs. Soybean *4CL1* (AF279267) sequence was in the second group. This sequence, mapped to chromosomes Gm17 (Glyma17g07190 at position 5,257,882 nt) and grouped with a soybean gene on chromosome Gm13 (Glyma13g01080 at position 798,830 nt) and a common bean gene on the chromosome Pv3 (Phvul003G147000 at position 34,642,252 nt). A subgroup containing soybean *4CL2* (AF002259) was further separated into two groups. The *4CL2* gene (Glyma13g44950) mapped on chromosome Gm13 (at position 44,256,607 nt) grouped with the soybean gene on chromosome Gm15 [Glyma15g00390 at position 194,042 nt; both soybean genes are annotated as 4CLs (KEGGORTH, Phytozome)] and a common bean gene on the chromosome Pv5 (Phvul.005G184500 at position 40,478,925 nt). Soybean gene Glyma17g07180 (position at Gm17:5,244,177 nt) was joined to this cluster while the second soybean sequence (Glyma17g07170 at position Gm17:5,222,676 nt) and common bean gene (Phvul.003G146900 at position Pv3:34,628,465 nt) made a separate branch. All three genes are annotated as 4CLs (KEGGORTH, Phytozome). Soybean genes *4CL3* (AF002258) on chromosome Gm11 (Glyma11g01240 at position 732,167 nt) and *4CL4* (X69955) on the chromosome Gm01 [Glyma01g44270 at position 54,978,159 nt, annotated as 4CL (KEGGORTH, Phytozome)] grouped together with a common bean gene on the chromosome Pv2 (Phvul.002G040100 at position 3,833,723 nt, annotated as 4CL (KEGGORTH, Phytozome)]. These genes belong to class II of 4CL enzymes. Two additional genes (soybean Glyma11g09710 at position Gm11:6,919,239 nt and common bean Phvul.011G020200 at position Pv:1,586,744 nt) made a separate branch of this group. The middle cluster was separated into two subclusters each consisting of one soybean and one common bean gene, all annotated as 4CLs (KEGGORTH, Phytozome). In addition, a group of two soybean (Glyma08g44190 and Glyma18g08550) and one common bean (Phvul.006G057300) *4CL* gene sequences [annotated as 4CLs (KEGGORTH, Phytozome)] formed an independent cluster (Figure 3).

**Figure 4:**
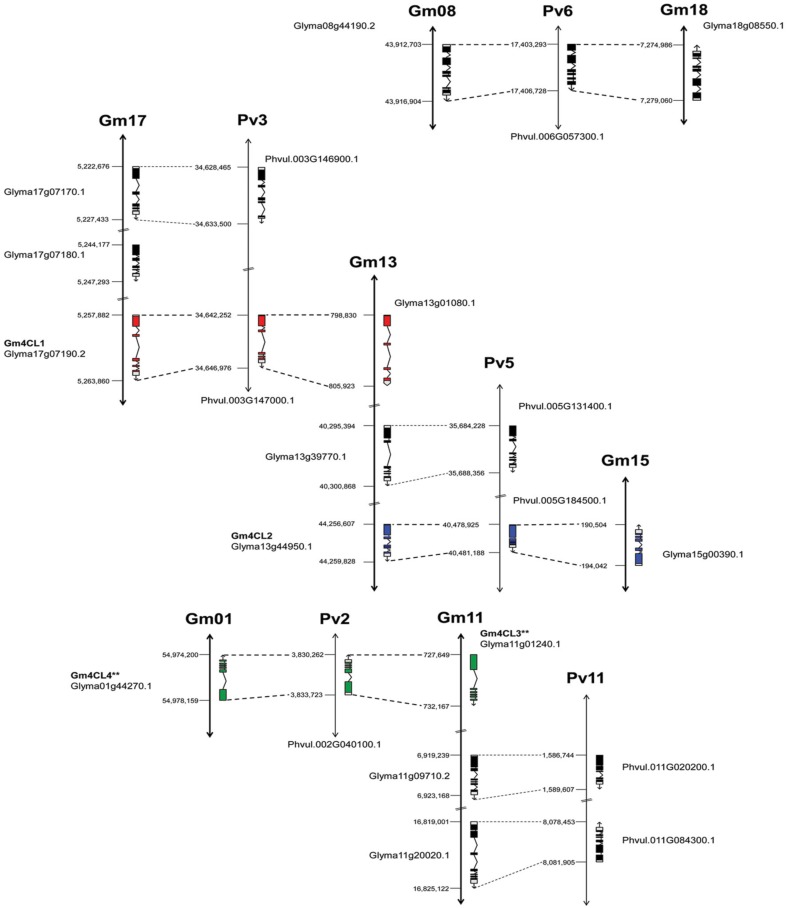
**Genome organization of *4CL* genes in soybean and common bean**. Black bars represent chromosomes. Exon/intron structure of *4CL* genes (Phytozome) is indicated. Exons are represented as filled boxes and introns are shown as lines between the exons. Sequence orientation on the chromosome is indicated with an arrow. ^**^Exon/intron structure of Glyma01g44270 and Glyma11g01240 loci was corrected based on the Phytozome/GenBank/cDNA sequence alignments.

The genome distribution of these genes is illustrated in Figure 4. Out of the 13 soybean 4CLs identified, nine are located on three chromosomes (Gm17, Gm13, and Gm11) and six out of eight common bean genes were also found on three chromosomes (Pv3, Pv5, and Pv11). For example, soybean chromosome Gm17 contains a cluster of three genes, including *4CL1* (Glyma17g07190) on the third position. A second copy of this gene is found on the top arm of chromosome Gm13 and the common bean *4CL1* gene (Phvul.003G146900) on the chromosome Pv3. The soybean *4CL2* gene (Glyma13g44950) was found on the lower arm of chromosome Gm13, and a second copy (Glyma15g00390) on chromosome Gm15 but in the reverse orientation. The common bean *4CL2* gene (Phvul.005G184500) was mapped to chromosome Pv5. The soybean *4CL4* gene (Glyma01g44270) was positioned on the chromosome Gm01 and the *4CL3* gene (Glyma11g01240) was mapped on the top arm of chromosome Gm11 but in the reverse orientation. Common bean *4CL3/4* gene (Phvul.002G040100) was mapped on the chromosome Pv2.

**Figure 5:**
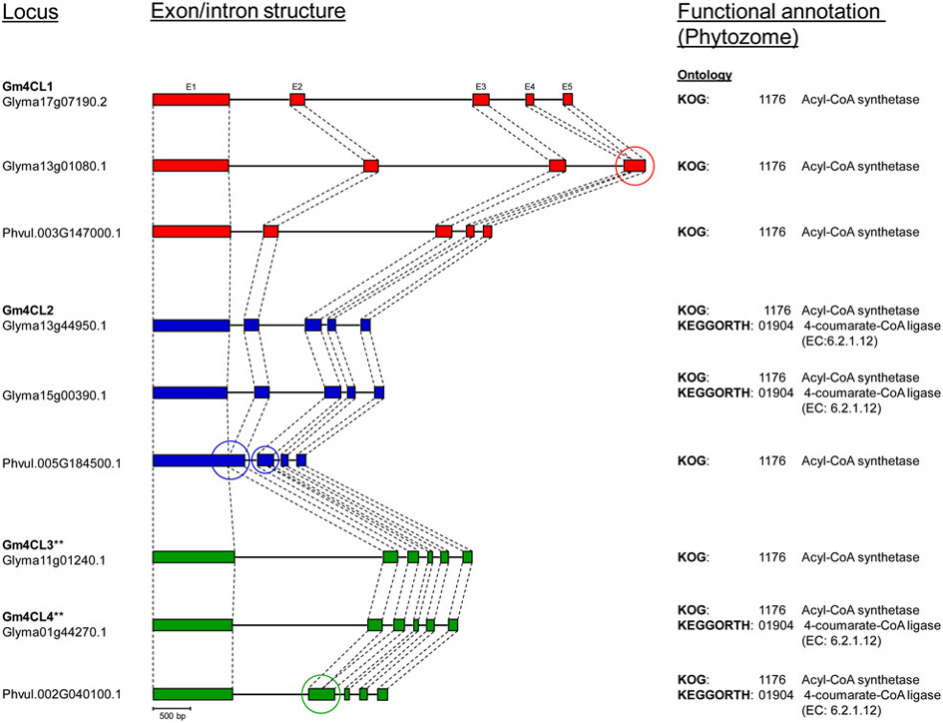
**Comparison of *4CL* genes exon/intron structure between soybean (Williams 82, Phytozome) and common bean (G19833, Phytozome)**. Exons (E) are represented as filled boxes and introns are shown as lines between the exons. ^**^Exon/intron structure (exon 1 and intron 1) of Glyma01g44270 and Glyma11g01240 loci was corrected based on the Phytozome/GenBank/cDNA sequence alignments. Circles indicate loss/gain of exon.

The annotated genes do differ in size and structure, and we examined exon/intron structure of six soybean and three common bean *4CL* genes (Table S3). They have coding regions of similar size [shortest (1617 bp) in *4CL2-2* (Glyma15g00390) and largest (1713 bp) in *4CL3* (Glyma11g01240)] arranged in four to six exons. All nine genes are characterized by the presence of a large exon 1 [978 bp in Glyma15g00390 (*4CL2-2*) to 1201 bp in common bean Phvul.005G185500 (*4CL2*)]. The sizes of introns were more variable, and the total size of introns ranged from 332 bp in common bean Phvul.005G184500 (*4CL2*) to 4758 bp in soybean Glyma13g01080 (*4CL1-2*). All three common bean 4CL sequences had smaller total intron sizes when compared to their soybean homologs within the same group. Comparison of these nine genes at the exon/intron level confirmed very close relationships between soybean *4CL3* and *4CL4* genes (Figure 5). Similarly, two soybean *4CL2* genes are closely related, as are soybean *4CL1* and common bean *4CL1*.

***Sequence polymorphisms of 4CL genes in common bean.*** Because of the central position in the phenylpropanoid pathway, development of gene-based markers for 4-coumarate:CoA ligase could have significant impacts on breeding common bean for various phenylpropanoids and different traits. Searches for potential inter-pool SNPs/indels were done by comparing the genomic sequences of eight *4CL* genes in the Mesoamerican cultivar OAC Rex (University of Guelph, GenBank accessions KF303286 to KF303293) with those in the reference, Andean landrace G19833 (Phytozome) (Table 7).

**Table 7:** **Eight 4CL loci in common bean cultivars G19833 and OAC Rex**.

**G19833—Phytozome^a^**		**OAC Rex—GenBank**		
**4CL locus**	**Size (bp)**	**Accession**	**Gene**	**Size (bp)**
Phvul.002G040100	3462	KF303286	*4CL-1*	3540
Phvul.003G146900	5036	KF303287	*4CL-2*	9800
Phvul.003G147000	4716	KF303288	*4CL-3*	4880
Phvul.005G131400	4129	KF303289	*4CL-4*	4160
Phvul.005G184500	2264	KF303290	*4CL-5*	2320
Phvul.006G057300	3436	KF303291	*4CL-6*	3696
Phvul.011G020200	2864	KF303292	*4CL-7*	2960
Phvul.011G084300	3453	KF303293	*4CL-8*	3477

a Accessed 5 June 2013.

Sequence differences were detected for all *4CL* genes, with the exception of the Phvul.011G020200 (Table 8). The alignment between the two genotypes for this gene was perfect (Figure S2). For the other *4CL* genes, differences in aligned sequences ranged from 17 bp in OAC Rex accession KF303290 (Phvul.005G184500 gene) to 4785 bp in the accession KF303287 (Phvul.003G146900 gene) and were found in both, coding and non-coding regions. For example, Phvul.006G057300 gene comparison revealed 18 single nucleotide substitutions and two deletions (eight nucleotides in intron 1 and a single nucleotide in intron 4) in OAC Rex sequence.

**Table 8:** **Polymorphism of *4CL* gene sequences in Mesoamerican common bean cultivar OAC Rex compared to the reference, Andean landrace G19833**.

**OAC Rex accession G19833 locus**	**Polymorphism**	**5**′**UTR**	**E1**	**I1**	**E2**	**I2**	**E3**	**I3**	**E4**	**I4**	**E5**	**I5**	**E6**	**3**′**UTR**
KF303286 Phvul.002G040100	SNPs^a^	2	3	21	0	0	0	0	0	0	0	−	−	2
	Insertions^b^	0	282–290	1342–1344	0	0	0	0	0	0	3291–3293	−	−	0
				1487–1512										
				2128										
				2294										
	Deletions^c^	116 (−4)	0	0	0	2680 (−2)	0	2918 (−4)	0	0	0	−	−	0
KF303287 Phvul.003G146900	SNPs	2	8	7	0	11	3	0	0	31	0	−	−	1
	Insertions	0	733–5411	6034	0	7059	0	0	0	8393–8398	0	−	−	0
										9149–9157				
	Deletions	0	0	6080 (−3)	0	7009 (−2)	0	0	0	8373 (−1)	0	−	−	9623 (−1)
				6377 (−1)		7041 (−4)				9171 (−5)				
						7281 (−2)				9428 (−10)				
						7476 (−2)								
KF303288 Phvul.003G147000	SNPs	0	2	2	0	17	2	6	0	5	1	−	−	0
	Insertions	0	0	1456	0	2219–2234	0	0	0	0	0	−	−	0
						2314–2325								
						2345–2346								
						3603–3604								
						3803–3807								
	Deletions	0	0	0	0	2098 (−9)	0	0	0	0	0	−	−	0
						2102 (−19)								
						2274 (−1)								
						2870 (−1)								
						2932 (−1)								
						3700 (−14)								
KF303289 Phvul.005G131400	SNPs	0	2	2	3	18	2	3	2	3	0	0	0	5
	Insertions	0	0	0	0	1663 1926	0	0	0	0	0	0	0	0
	Deletions	0	0	1305 (−1)	0	1858 (−1)	0	0	0	0	0	0	0	0
						1884 (−19)								
						1992 (−3)								
						2524 (−42)								
KF303290 Phvul.005G184500	SNPs	0	9	1	2	0	0	1	0	−	−	−	−	4
	Insertions	0	0	0	0	0	0	0	0	−	−	−	−	0
	Deletions	0	0	0	0	0	0	0	0	−	−	−	−	0
KF303291 Phvul.006G057300	SNPs	0^a^	3	4	2	0	0	0	0	4	0	4	1	−
	Insertions	0^b^	0	0	0	0	0	0	0	0	0	0	0	−
	Deletions	0^c^	0	864 (−8)	0	0	0	0	0	2716 (−1)	0	0	0	−
KF303292 Phvul.011G020200	SNPs	0	0	0	0	0	0	0	0	0	0	−	−	0
	Insertions	0	0	0	0	0	0	0	0	0	0	−	−	0
	Deletions	0	0	0	0	0	0	0	0	0	0	−	−	0
KF303293 Phvul.011G084300	SNPs	0	6	2	5	6	1	4	0	0	0	0	0	1
	Insertions	0	0	890	0	0	0	0	0	0	0	0	0	0
	Deletions	0	0	0	0	1841 (−70)	0	0	0	0	0	0	0	0
						1982 (−1)								
						1998 (−1)								
						2056 (−1)								

a Number of SNPs in OAC Rex sequence;

b Deletion (number of missing nucleotides in OAC Rex sequence compared to G19833) starts after indicated nucleotide;

c Nucleotide number(s) indicate position of an insertion in OAC Rex sequence.

Although differences in coding regions of several *4CL* genes led to the amino acids changes, none of them would likely affect enzyme activity (Figure S3). The only exception was OAC Rex gene Phvul.003G146900 (accession KF303287). This gene (9800 bp) was interrupted by 4768 bp a LTR transposon insertion in exon 1 at the position 733..5411 nt, which would result in an inactive enzyme.

## Discussion

### Isolation of phenylpropanoid pathway gene fragments in common bean

The impetus for the current work was the lack of systematic information for the genes of the phenylpropanoid pathway in common bean. This is a real limitation for research dealing with the synthesis of a number of important secondary metabolites, including isoflavonoids, anthocyanins and lignin. At the beginning of this study only few phenylpropanoid pathway gene sequences were available in public databases for common bean. In the general phenylpropanoid pathway, all three genes coding for phenylalanine ammonia lyase (*PAL1*, Edwards et al., 1985; *PAL2* and *PAL3*, Cramer et al., 1989) and a single gene for cinnamate 4-hydroxylase (*C4H*, Nedelkina et al., 1999) were previously isolated in common bean. A number of enzymes are involved in the lignin/lignan branch of the phenylpropanoid pathway, in most cases, encoded by small gene families. Only few sequences for lignin peroxidases were available for common bean at the beginning of this study (Blee et al., 2001). The flavonoid/anthocyanin branch, which encodes the enzymes involved in the production of anthocyanins and proanthocyanidins (condensed tannins) associated with seed coat color as well as different flavonoids involved in nodulation and plant defense, is probably the most studied segment of the phenylpropanoid pathway. Numerous enzymes, coded mostly by small gene families, catalyze the synthesis of these compounds. However, only gene sequences for chalcone synthase (*CHS*, Ryder et al., 1987) and chalcone isomerase (*CHI*, Mehdy et al., unpublished, sequence available at http://www.ncbi.nlm.nih.gov/nuccore/Z15046.1) were available for common bean in databases before the initiation of this study. The isoflavonoid branch is unique to legumes and poorly understood in common bean. No sequence information was available in common bean for the genes coding for the enzymes of this branch of the phenylpropanoid pathway, except a *P. coccineus* EST corresponding to isoflavone reductase-like protein (IFR-l, Bui et al., unpublished, sequence available at http://www.ncbi.nlm.nih.gov/nucest/ca914441) at the initiation of this study. The regulation of the phenylpropanoid pathway by transcription factors is well characterized in maize, Arabidopsis, snapdragon, or petunia and numerous genes coding for transcription factors have been isolated from these plants (Chandler et al., 1989; Martin et al., 1991; Menting et al., 1994; Kranz et al., 1998). However, at the beginning of this study only two common bean gene sequences for regulatory genes of the phenylpropanoid pathway [*KAP2* (Lindsay et al., 2002) and bZIP *TGA1* (Tucker et al., 2002)] were available.

The current work added significantly to our knowledge of a number of important genes in the phenylpropanoid pathway. We cloned, sequenced and analyzed gene fragments for 46 structural and regulatory genes coding for 25 major enzymes and seven regulatory proteins of this pathway in common bean. In addition to previously isolated genes [*PAL* (*PAL1, PAL2*, and *PAL3*), *C4H*, PER (*FBP1* and *FBP4*), *CHS, CHI*, and *KAP2*], we identified gene fragments homologous to soybean *4CL1* and *4CL2* (general), most of the genes coding for enzymes of lignin/lignan branch (CAD, CCR, C3H, COMT, F5H, and LAC), relatively good coverage of flavonoid/anthocyanin branch (CHR, F3H, F3′H, RT, UF5GT, ANR, AAT, GST, and VT) and gene fragments coding for three enzymes of the isoflavonoid branch (IFS, IFR, and IOMT).

Difficulties in cloning were encountered with a number of genes, particularly with members of the larger gene families (such as *4CL, CCOMT*, or *CHS*) and transcription factors. As an example, although initially, 18 different transcription factor sequences were targeted, only seven authentic common bean transcription factors (Myb4, LIM, KAP2, HD, WIP, Myb15, and TT2) were cloned and identified. These difficulties might be attributed to the fact that, because of the lack of detailed genomic information at the beginning of this work, some of the PCR primers that were designed spanned introns and were not effective in amplifying a product from the genomic DNA template. Also, for targets that were members of large gene families, unanticipated polymorphisms in the gene sequences could have prevented their amplification by PCR.

In addition to the gene fragments, we obtained the complete genomic sequences of six phenylpropanoid pathway genes (*PAL2, PAL3, CHS-A, CHS-B, DFR*, and *Myb15*) from G19833 BAC clones. These give valuable information about the intron/exon structure for these genes as well as information about the upstream promoter regions. They are of particular interest because some genes, including *Myb15* and *PAL2*, map close to the pigmentation locus *P* (Yadegari, unpublished), or represent the first committed step in the pathway (*PAL*), or, like *CHS* and *DFR*, are differentially expressed in black vs. white seeded beans (Yadegari, unpublished; Siddiqua et al., unpublished).

Associations between seed coat color genes and enzymes of anthocyanin biosynthetic pathway have been identified in soybean. For example, the *I* locus was associated with a *CHS* gene cluster on chromosome Gm08 (Todd and Vodkin, 1996) while the *T* locus was associated with the *F3′H* gene on chromosome Gm06 (Toda et al., 2002; Zabala and Vodkin, 2003). However, in common bean none of the nine seed coat color genes has been associated with a phenylpropanoid pathway genes. Caldas and Blair (2009) identified a number of QTL for condensed tannins on several linkage groups and associated them with putative color genes. For example, QTL Cst1a (closest marker Y04.2) on linkage group Pv7 was associated with *P* gene, Cst2a (OAM10) on Pv3 with *Z* gene, Cst3b (B1DB1D) on Pv10 with *Bip* while QTL Cst1c (L041.05) on Pv8 was associated with *C* gene. The current study identified potential associations between several seed coat color genes and phenylpropanoid pathway genes in common bean. In particular, seed coat pattern gene *Z* (zonal) mapped close to *4CL1* gene on chromosome Pv3, *V* gene (violet factor) close to F3′5′H on Pv6 and *P* gene (pigment ground factor) was placed close to the transcription factor Myb15 on chromosome Pv7. In a number of cases regions of common bean and soybean chromosomes that share synteny also contained mapped seed coat color genes. For example, Pv2 (*Rk, B*) and Gm08 (*I, O*), Pv3 (*Z*) and Gm02 (*Wp*), Pv6 (*V*) and Gm13 (*W1*), Pv08 (*Gy, C*) and Gm (*R*), and Pv09 (*T*) and Gm06 (*T*). The phenylpropanoid pathway genes that are located in these regions would be interesting to explore more thoroughly as candidates for these classical color genes. The information gained from studies in one species may help to narrow the search in the other.

### Development and application of phenylpropanoid pathway gene sequence-based common bean-soybean comparative map

The release of the complete common bean genome sequence (http://www.phytozome.net/commonbean.php) created the opportunity to expand our ongoing phenylpropanoid pathway research by *in silico* mapping of annotated phenylpropanoid pathway gene sequences. Because of close relatedness between common bean and soybean, a phenylpropanoid pathway gene sequence map was also constructed for soybean, which was sequenced in 2010 (Schmutz et al., 2010). This allowed the more extensive knowledge for soybean phenylpropanoid metabolism to be directly related to common bean.

Comparative mapping has been widely used in plants. Good examples are comparative maps of grasses (Gale and Devos, 1998; Feuillet and Keller, 2002) and Brassicas (Lagercrantz, 1998; Parkin et al., 2005). Using legume anchor markers (Hougaard et al., 2008) and common bean g markers (McConnell et al., 2010) syntenic regions in a number of legumes have been linked. Several comparative mapping studies with soybean and common bean have been conducted (Galeano et al., 2009; McClean et al., 2010; Galeano et al., 2011). The current study expanded upon research done by McClean et al. (2010) with a focus on genes coding for enzymes of phenylpropanoid pathway. The genome comparison confirmed the transferability of SSR markers among legumes (He et al., 2006; Somta et al., 2009) and the common bean g markers to soybean (McClean et al., 2010). The comparison of the complete genomes for common bean and soybean allowed numerous phenylpropanoid pathway genes to be mapped and their annotations to be supported by the independent work in the other genome. The current work confirmed previously reported extensive syntenic regions between the two species (Galeano et al., 2009; McClean et al., 2010) but with the addition of phenylpropanoid pathway genes. The high degree of shared synteny between common bean and soybean meant that the identity of certain genes was strengthened by their chromosome context. Phenylpropanoid pathway gene fragments isolated in this study as well as original (query) sequences were positioned on one chromosome in common bean but on two chromosomes in soybean. This is in agreement with the close relatedness between the two species (Galeano et al., 2009; McClean et al., 2010) and the belief that the soybean genome underwent duplication after the establishment of the *Phaseolus* and *Glycine* genomes from a common progenitor (Shoemaker et al., 2008; Schmutz et al., 2010).

Comparative studies of the common bean and soybean can lead to deeper understanding of gene families in the two species. For example, when *4CL4* (Accession X69955) is used as a query for the *4CL* gene family in common bean and soybean, 128 homologous proteins were retrieved from the Phytozome. The *4CL* gene family in soybean (Lindermayr et al., 2002), which encodes 4-coumarate:CoA ligase has a central position in phenylpropanoid pathway and different 4CL isoforms with distinct substrate conversion profiles are involved in biosynthesis of various phenylpropanoids (lignin vs. flavonoids). A cluster analysis grouped 33 common bean and 65 soybean homologous sequences into gene/protein families. Clustering protein sequences into homologous groups can help to annotate uncharacterized protein sequences. Further analysis of the clusters containing four soybean 4CL isozymes (genomic and protein sequences) confirmed the separation of the gene family into two classes (Lindermayr et al., 2002), where *4CL1* (Gm17) and *4CL2* (Gm13) formed two individual but close clusters while similar *4CL3* (Gm11) and *4CL4* (Gm01) grouped together. Segments of chromosomes Gm01 and Gm11 containing *4CL4* and *4CL3* genes are duplicated in soybean and are syntenic to the region of bean chromosome Pv2, which contain a third member of this cluster. Clustering revealed duplication of *4CL* genes and potentially new *4CL* (or 4CL-like) gene family members. The *4CL1* locus (Gm17) grouped with a homologous soybean sequence on Gm13 (duplicated region) and a common bean sequence on Pv3 (syntenic region). Similarly, *4CL2* locus (Gm13) formed a cluster with a homologous locus on soybean chromosome Gm15 (duplicated region) and a common bean locus on chromosome Pv5 (syntenic region). Homologous loci that clustered together shared similar gene structure. This study illustrates how *in silico* mapping can aid in identification of additional (duplicated) *4CL* (or 4CL-like) loci and potentially other genes in legumes. This would be the first step for further verification by functional analysis and better understanding of phenylpropanoid pathway.

From the alignment and comparison of the eight Andean (G19833 at Phytozome) and Mesoamerican (OAC Rex, University of Guelph accessions KF303286 to KF03293) *4CL* (or 4CL-like) gene sequences numerous SNPs and several indels were found, in both the coding and non-coding regions for seven genes. Because of the central position of 4CL, development of these markers might be useful in breeding common bean (and other legumes) for various traits associated with the phenylpropanoid pathway. For example, 4CL was identified as a potential target for manipulating lignin biosynthesis and mechanical properties of poplar (Voelker et al., 2010) and rice (Gui et al., 2011).

## Conclusions

The current work was motivated by the lack of systematic information regarding the genes of the phenylpropanoid pathway in the common bean. By cloning numerous genes and gene fragments that encode major enzymes of this pathway, the study added significantly to the knowledge of the phenylpropanoid pathway in the common bean. In addition, potential associations between several seed coat color genes and the phenylpropanoid pathway genes were identified in the common bean.

While conducting this study, the complete sequence of the common bean genome was released, giving us the opportunity to map *in silico* the annotated phenylpropanoid pathway gene sequences. Due to better understanding of the phenylpropanoid pathway in the soybean, and close relatedness of the common bean to the soybean, a sequence-based *in silico* map for soybean allowed us to relate these findings directly to the common bean. Moreover, phenylpropanoid pathway gene annotations were supported by the independent work in the two genomes.

The comparison of the complete genomes for the common bean and the soybean confirmed the previously reported extensive syntenic regions between the two species, but this time with the addition of phenylpropanoid pathway genes. With the high degree of synteny between the two species, the identity of certain genes was strengthened by their chromosome context. Phenylpropanoid pathway gene fragments were positioned on one chromosome in the common bean, but on two chromosomes in the soybean. This is in agreement with the close relationship between the two species and the duplication of the soybean genome. Many of the common bean-soybean syntenic regions contained mapped seed coat color genes. It would be interesting to explore the phenylpropanoid pathway genes that are located in these regions more thoroughly as candidates for these classical color genes. The information gained from studies in one species may help to narrow the search in the other.

Finally, using the *4CL* gene family as an example, this study illustrates how *in silico* mapping can lead to better understanding of gene families and aid in the identification of additional (duplicated) genes in legumes. Homologous loci that clustered together shared a similar gene structure. This would be the first step for further verification by functional analysis and for better understanding of the legume phenylpropanoid pathway. Indels identified in the comparison of Andean and Mesoamerican *4CL* common bean sequences can be used to develop 4CL gene-based intra-pool markers. Due to its central position in the pathway, the development of 4CL gene-based markers might be useful in breeding common bean (and other legumes) for many traits associated with the phenylpropanoid pathway.

We believe that the information obtained in this study has the potential to accelerate and simplify breeding common bean for increased contents of beneficial phenylpropanoids and is transferable to other legumes.

### Conflict of interest statement

The authors declare that the research was conducted in the absence of any commercial or financial relationships that could be construed as a potential conflict of interest.

## Supplementary material

Figure S1 **Phylogenetic relationships among common bean and soybean 4CL homologous proteins**. Trees were generated from 4CL protein sequence analyses in CLC Genomics Workbench 3 using Neigbor Joining (NJ) algorithm and 1000 bootstrap replications. Scale represents genetic distance and node numbers indicate bootstrap support values. **(A)** Primary transcripts (98 protein sequences); **(B)** Twenty-one 4CL proteins selected based on similarity to soybean 4CL1 (red), 4CL2 (blue) and 4CL3/4CL4 (green).

Figure S2 **Nucleotide sequence comparison of eight *4CL* loci (A-H) between Andean common bean landrace G19833 (reference) and Mesoamerican cultivar OAC Rex**. Genomic sequences were aligned in ClustalW at EBI. Polymorphic sites (SNPs/indels) are indicated (G19833-blue, OAC Rex-red).

Figure S3 **Comparison of G19833 (Andean) and OAC Rex (Mesoamerican) 4CL amino acid sequences**. G19833 and OAC Rex 4CL sequences are aligned in ClustalW at EBI. Positions of conserved features (NCBI) in aligned proteins are indicated.

Table S1 **Common bean phenylpropanoid pathway gene sequence-based *in silico* map**.

Table S2 **Soybean phenylpropanoid pathway gene sequence-based *in silico* map**.

Table S3 **Exon/intron structure of selected *4CL* genes in common bean and soybean**.
