# Supplementary material for: In silico comparison of genomic regions containing genes coding for enzymes and transcription factors for the phenylpropanoid pathway in Phaseolus vulgaris L. and Glycine max L. Merr
Source: Front Plant Sci. 2013 Sep 5;4:317. doi: 10.3389/fpls.2013.00317 (PMC3763686; doi:10.3389/fpls.2013.00317)
Supplement: Figure S1 — Phylogenetic relationships among common bean and soybean 4CL homologous proteins. Trees were generated from 4CL protein sequence analyses in CLC Genomics Workbench 3 using Neigbor Joining (NJ) algorithm and 1000 bootstrap replications. Scale represents genetic distance and node numbers indicate bootstrap support values. (A) Primary transcripts (98 protein sequences); (B) Twenty-one 4CL proteins selected based on similarity to soybean 4CL1 (red), 4CL2 (blue) and 4CL3/4CL4 (green). [file DataSheet1.ZIP › Supplementary Material/Supplemental Figure S2 - Nucleotide sequence comparison of 4CLs.doc]

```
Figure S2 | Nucleotide sequence comparison of eight 4CL loci (A-H) between Andean common bean landrace G19833 (reference) and Mesoamerican cultivar OAC Rex

A) Nucleotide sequence comparison of the 4CL locus Phvul.002G040100 between two common bean genotypes


G19833 Phvul.002G040100        --------------------------------AGAGTGGCTGAAAGAAGA 18
OAC Rex KF303286               AAATGTTAGAGTTGGGGGTAGGTAGGTAAGTAAGAGTGGCTGGAAGAAGA 50
                                                               ********** *******

G19833 Phvul.002G040100        ACGCTTGTTCTGAAAACGTGATCCAATGTGTGGCTGCTCCTTCTCCATCA 68
OAC Rex KF303286               ACGCTTGTTCTGAAAACGTGATCCAATGTGTGGCTGCTCCTTCTCCATCA 100
                               **************************************************

G19833 Phvul.002G040100        TTCATTCACTCACTCACTCATTCTCACCTAACACCCTTTCCCTTCCCTTC 118
OAC Rex KF303286               TTCATTCACTCACTCA----TTCTCACCTAACACCCTTTCCCTTCCCTTC 146
                               ****************    ******************************

G19833 Phvul.002G040100        TCTATATTACCTTCTTCACATGTTTCCCTCTCCTCACTCTCTCTCTCTCT 168
OAC Rex KF303286               TCTATATTACCTTCTTCCCATGTTTCCCTCTCCTCACTCTCTCTCTCTCT 196
                               ***************** ********************************

G19833 Phvul.002G040100        CTCTCTCTCCTTAAACTCTTCATTCCACTCTAACTAACACACCCCATTCA 218
OAC Rex KF303286               CTCTCTCTCCTTAAACTCTTCATTCCACTCTAACTAACACACCCCATTCA 246
                               **************************************************
                                 ATG                                             EXON 1
G19833 Phvul.002G040100        CCATGCTAAGTGTTGCTCCTTCTCTTGATACCAAC---------ACCAAA 259
OAC Rex KF303286               CCATGCTAAGTGTTGCTCCTTCTCTTGATACCAACACCAAAGACACCAAA 296
                               ***********************************         ******

G19833 Phvul.002G040100        ACAGACCAAACTCAAATTCCCCAAACTACCCATGTTTTCAAGTCTAAATT 309
OAC Rex KF303286               ACAGACCAAACTCAAATTCCCCAAACTACCCATGTTTTCAAGTCTAAATT 346
                               **************************************************

G19833 Phvul.002G040100        ACCAGACATACCAATCTCCAACCACCTATCCCTCCATGATTACTGCTTCC 359
OAC Rex KF303286               ACCAGACATACCAATCTCCAACCACCTATCCCTCCATGATTACTGCTTCC 396
                               **************************************************

G19833 Phvul.002G040100        AAAACCTCTCCCAATTTGCAGACCGTCCCTGCCTCATCGTCGGCTCCGCC 409
OAC Rex KF303286               AAAACCTCTCCCAATTTGCAGACTGTCCCTGCCTCATCGTCGGCTCCGCC 446
                               *********************** **************************

G19833 Phvul.002G040100        TCCAAAACCTACACCTACGCACAGACGTACCTTATTTCGCGCAAGATCGC 459
OAC Rex KF303286               TCCAAAACCTACACCTACGCACAGACGTACCTTATTTCGCGCAAGATCGC 496
                               **************************************************

G19833 Phvul.002G040100        TGCCGGATTCTCGAATCTCGGAATCCGCAAGGGCGACGTCGTCATGATCC 509
OAC Rex KF303286               TGCCGGATTCTCGAATCTCGGAATCCGCAAGGGCGACGTCGTCATGATCC 546
                               **************************************************

G19833 Phvul.002G040100        TCCTCCAGAACTCCGCCGAGTTCGTCCTCTCCTTCCTCGCCGCCTCCATG 559
OAC Rex KF303286               TCCTCCAGAACTCCGCCGAGTTCGTCCTCTCCTTCCTCGCCGCCTCCATG 596
                               **************************************************

G19833 Phvul.002G040100        GTCGGAGCTGTAGCCACCACCGCCAACCCGTTCTACACCGCCGCCGAGAT 609
OAC Rex KF303286               GTCGGAGCTGTAGCCACCACCGCCAACCCGTTCTACACCGCCGCCGAGAT 646
                               **************************************************

G19833 Phvul.002G040100        CTTCAAGCAGTTCACCGCCTCCAAGGCGAAGCTGATCATAACGCAGGCAA 659
OAC Rex KF303286               CTTCAAGCAGTTCACCGCCTCCAAGGCGAAGCTGATCATAACGCAGGCAA 696
                               **************************************************

G19833 Phvul.002G040100        TGTACGTGGAAAAGCTCCGCAGCCACGACGCCGGATGGAGCCTCGGCGAG 709
OAC Rex KF303286               TGTACGTGGAAAAGCTCCGCAGCCACGACGCCGGATGGAGCCTCGGCGAG 746
                               **************************************************
G19833 Phvul.002G040100        GACTTCAAGGTCGTGACCGTCGACGATCCGCCGGAGAATTGCCTCCACTT 759
OAC Rex KF303286               GACTTCAAGGTCGTGACCGTCGACGATCCGCCGGAGAATTGCCTCCACTT 796
                               **************************************************

G19833 Phvul.002G040100        CTCCGTGCTCTCTGAGGCGAACGAGAGCGACGCTCCCGAGGTGGAGATTC 809
OAC Rex KF303286               CTCCGTGCTCTCTGAGGCGAACGAGAGCGACGCTCCCGAGGTGGAGATTC 846
                               **************************************************

G19833 Phvul.002G040100        ATCCGGACGACGCCGTGGCGCTGCCGTTTTCCTCCGGCACCACGGGCCTG 859
OAC Rex KF303286               ATCCGGATGACGCCGTGGCGCTGCCGTTTTCCTCCGGCACCACGGGCCTG 896
                               ******* ******************************************

G19833 Phvul.002G040100        CCGAAGGGAGTGATCCTCACGCACAAGAGTTTGACCACGAGCGTGGCGCA 909
OAC Rex KF303286               CCGAAGGGAGTGATCCTCACGCACAAGAGTTTGACCACGAGCGTGGCGCA 946
                               **************************************************

G19833 Phvul.002G040100        GCAGGTTGACGGGGAGAATCCTAATCTCTACCTCACCACTGAGGACGTGC 959
OAC Rex KF303286               GCAGGTTGACGGGGAGAATCCTAATCTCTACCTCACCACCGAGGACGTGC 996
                               *************************************** **********

G19833 Phvul.002G040100        TCCTCTGCGTGCTTCCGCTGTTTCACATATTCTCGCTCAACAGCGTGCTG 1009
OAC Rex KF303286               TCCTCTGCGTGCTTCCGCTGTTTCACATATTCTCGCTCAACAGCGTGCTG 1046
                               **************************************************

G19833 Phvul.002G040100        TTATGCGCGCTTAGAGCCGGGAGCGGCGTTTTGCTTATGCAGAAGTTCGA 1059
OAC Rex KF303286               TTATGCGCGCTTAGAGCCGGGAGCGGCGTTTTGCTTATGCAGAAGTTCGA 1096
                               **************************************************

G19833 Phvul.002G040100        GATTGGGACGCTGCTGGAGCTTATACAGCGCTACCGCGTGTCGGTGGCGA 1109
OAC Rex KF303286               GATTGGGACGCTGCTGGAGCTTATACAGCGCTACCGCGTGTCGGTGGCGA 1146
                               **************************************************

G19833 Phvul.002G040100        TGGTGGTGCCGCCGCTGGTGCTGGCGCTGGCCAAGAATCCGATGGTGGCG 1159
OAC Rex KF303286               TGGTGGTGCCGCCGCTGGTGCTGGCGCTGGCCAAGAATCCGATGGTGGCG 1196
                               **************************************************

G19833 Phvul.002G040100        AACTTTGACCTCAGTTCAATACGGTTAGTTCTCTCCGGCGCCGCTCCCTT 1209
OAC Rex KF303286               AACTTTGACCTCAGTTCAATACGGTTAGTTCTCTCCGGCGCCGCTCCCTT 1246
                               **************************************************

G19833 Phvul.002G040100        GGGTAAGGAACTCGAAGAGGCTCTCCACAGCAGGGTGCCTCACGCTGTTT 1259
OAC Rex KF303286               GGGTAAGGAACTCGAAGAGGCTCTCCACAGCAGGGTGCCTCACGCTGTTT 1296
                               **************************************************
                                                                                 INTRON 1
G19833 Phvul.002G040100        TGGGACAGgtaaataattacaccaaaactaattattattattatt---at 306
OAC Rex KF303286               TGGGACAGgtaaataattacaccaaaactaattattattattattattat 1346
                               *********************************************   **

G19833 Phvul.002G040100        ctacgtaaaactctaatttaaatgataaaaataataattaaaaattatca 1356
OAC Rex KF303286               ctacgtaaaactctaatttaaatgataaatataataattaaaaattacca 1396
                               ***************************** ***************** **

G19833 Phvul.002G040100        ctatactattaaaaaataatatcaattagaaattatagcatataagaacc 1406
OAC Rex KF303286               ctatactattaaaaaataatatcaattagaaattatagcatataagaacc 1446
                               **************************************************

G19833 Phvul.002G040100        attaaaaaataatattaattaaaagttaaaatattttatg---------- 1446
OAC Rex KF303286               attaaaaaataatattaattaaaaattaaaatattttatagtatacaagt 1496
                               ************************ **************

G19833 Phvul.002G040100        ----------------atatataagtagatgggaatttagtaattttatg 1480
OAC Rex KF303286               atacataatatatataatatataagtagatgcgaatttattgattttatg 1546
                                               *************** ******* * ********
```

```
G19833 Phvul.002G040100        agattaaattaaatttaaagtttatttttattaatatagttaaaattatt 1530
OAC Rex KF303286               agattaaattaaatttaaattttatttttattaatatagttaaaattatt 1596
                               ******************* ******************************

G19833 Phvul.002G040100        taatattacgttaggggaaaatttataagaatacaaatggtcgttttcat 1580
OAC Rex KF303286               taatattacgttaaaggcaaatttataggaatacaaatggtcgttttcat 1646
                               *************  ** ********* **********************

G19833 Phvul.002G040100        tatttatttctcatcaaccttttgcatgcgataatgggcatatatgaagt 1630
OAC Rex KF303286               tatttatttctcatcaaccttttgcatgcgataatgggcatatatgaagt 1696
                               **************************************************

G19833 Phvul.002G040100        tgtatattaattaagaataccatggacacgtgtcaccttctcctccactg 1680
OAC Rex KF303286               tgtatattaattaagaataccatggacacgtgtcaccttctcctccactg 1746
                               **************************************************

G19833 Phvul.002G040100        actgttactaattaagaatataaacatattttctttatatataggtgcag 1730
OAC Rex KF303286               actgttattaattaagaatataaacatattttctttatatataggtgctg 1796
                               ******* **************************************** *

G19833 Phvul.002G040100        acttttaagaatatacgtctaaataaatcattaaaatatttcattatagc 1780
OAC Rex KF303286               acttttaagaatatacgtctaaataaatcattaaaatatttcattatagc 1846
                               **************************************************

G19833 Phvul.002G040100        tgttaaaaaaatattaaaatctttacttgagtgtgtgcatgcaagttttt 1830
OAC Rex KF303286               tgttaaaaaaatattaaaatctttacttgagtgtgtgcatgcaaaatttt 1896
                               ********************************************  ****

G19833 Phvul.002G040100        atggtagttagctcaccaaccaccccacaccaaacaccgaaaccaaaacg 1880
OAC Rex KF303286               atggtagttagctcaccaaccaccccacaccaaacaccgaaaccaaaacg 1946
                               **************************************************

G19833 Phvul.002G040100        gtgaaatagaagcacaagcatccaaacaagacttttagtgacgtgtacaa 1930
OAC Rex KF303286               gtgaaatagaagcacaagcatccaaacaagacttttagtgacgtgtacaa 1996
                               **************************************************

G19833 Phvul.002G040100        cttgacaccttcattttcattgccatttggcacacgtgacgtaagccaaa 1980
OAC Rex KF303286               cttgacaccttcattttcattgccatttggcacacgtgacgtaagccaaa 2046
                               **************************************************

G19833 Phvul.002G040100        ctaacagtggaccagtcaacaattcactgcacctccctctgcaacctaat 2030
OAC Rex KF303286               ctaacagtggaccagtcaacaattcactgcacctccccctgcaacctaat 2096
                               ************************************* ************

G19833 Phvul.002G040100        tcctttcattttaaatatttttttaaaaaat-caagtagcacacagatac 2079
OAC Rex KF303286               tcctttcattttaaatatttaaaaaaaaaattcaagtagcacacagatac 2146
                               ********************    ******* ******************

G19833 Phvul.002G040100        atgatatatgaaatctcaatataaatataagtttatatattatataatta 2129
OAC Rex KF303286               atgatatatgaaatctcaatataaatataagtttatatattatataatta 2196
                               **************************************************

G19833 Phvul.002G040100        taaattaataataaattatataaatatatttcaattttttttaggaaaat 2179
OAC Rex KF303286               taaattaataataaattatataaatatatttcaattttttttaggaaaat 2246
                               **************************************************

G19833 Phvul.002G040100        aatatttctcatgattaatttaaaataatttatggatcttatgttgt-GA 2228
OAC Rex KF303286               aatatttctcatgattaatttaaaataatttatggatcttatgttgttga 2296
                               *********************************************** **
                                                                                 EXON 2
G19833 Phvul.002G040100        ctttaaaatgcatgtatgttacagGGTTACGGGATGACAGAAGCAGGACC 2278
OAC Rex KF303286               ctttaaaatgcatgtatgttacagGGTTACGGGATGACAGAAGCAGGACC 2346
                               **************************************************
```

```
G19833 Phvul.002G040100        AGTGCTTTCCATGTGTTTGGGGTTTGCAAAGCAACCATTTCCAACAAAAT 2328
OAC Rex KF303286               AGTGCTTTCCATGTGTTTGGGGTTTGCAAAGCAACCATTTCCAACAAAAT 2396
                               **************************************************

G19833 Phvul.002G040100        CGGGATCTTGTGGAACTGTTGTGAGAAATGCAGAACTAAAGGTTGTTGAC 2378
OAC Rex KF303286               CGGGATCTTGTGGAACTGTTGTGAGAAATGCAGAACTAAAGGTTGTTGAC 2446
                               **************************************************

G19833 Phvul.002G040100        CCTGAAACTGGTCGTTCTCTCGGCTACAATCAGCCCGGTGAAATTTGCAT 2428
OAC Rex KF303286               CCTGAAACTGGTCGTTCTCTCGGCTACAATCAGCCCGGTGAAATTTGCAT 2496
                               **************************************************

G19833 Phvul.002G040100        CCGAGGGCTACAGATCATGAAAGGATATCTGAACGATGATCAAGCCACAG 2478
OAC Rex KF303286               CCGAGGGCTACAGATCATGAAAGGATATCTGAACGATGATCAAGCCACAG 2546
                               **************************************************

G19833 Phvul.002G040100        CATCCACCATCGATGCAGAGGGTTGGCTTCACACCGGTGATGTTGGCTAT 2528
OAC Rex KF303286               CATCCACCATCGATGCAGAGGGTTGGCTTCACACCGGTGATGTTGGCTAT 2596
                               **************************************************

G19833 Phvul.002G040100        GTAGACGATGACGATGAAATTTTCATCGTTGACAGGGTCAAAGAACTCAT 2578
OAC Rex KF303286               GTAGACGATGACGATGAAATTTTCATCGTTGACAGGGTCAAAGAACTCAT 2646
                               **************************************************
                                                                                 INTRON 2
G19833 Phvul.002G040100        CAAATTTAAAGGCTTCCAGgtatatatatatatatacacacacatatata 2628
OAC Rex KF303286               CAAATTTAAAGGCTTCCAGgtatatatatatata--cacacacatatata 2694
                               **********************************  **************

G19833 Phvul.002G040100        aacatctaagttaaatttgcctaaaagtttcagagaaaaaaaaattgtga 2678
OAC Rex KF303286               aacatctaagttaaatttgcctaaaagtttcagagaaaaaaaaattgtga 2744
                               **************************************************
                                                                                 EXON 3
G19833 Phvul.002G040100        caatgacaagtgattttgtttttgccagGTGCCCCCTGCAGAACTTGAAG 2728
OAC Rex KF303286               caatgacaagtgattttgtttttgccagGTGCCCCCTGCAGAACTTGAAG 2794
                               **************************************************
                                                                                 INTRON 3
G19833 Phvul.002G040100        GGCTTCTTGTAAGCCATCCCTCCATTGCAGACGCAGCAGTTGTCCCgtaa 2778
OAC Rex KF303286               GGCTTCTTGTAAGCCATCCCTCCATTGCAGACGCAGCAGTTGTCCCgtaa 2844
                               **************************************************

G19833 Phvul.002G040100        gcttcttaatcttctgaatttgaatgacttacattgtgcatatatatata 2828
OAC Rex KF303286               gcttcttaatcttctgaatttgaatgacttacattgtgcatatatatata 2894
                               **************************************************

G19833 Phvul.002G040100        tatatatatgtatgtatgtatgtatataactgaccattaatttgatcgat 2878
OAC Rex KF303286               tatatatatgtatgtatgtatgta----actgaccattaatttgatcgat 2940
                               ************************    **********************
                                                                                 EXON 4
G19833 Phvul.002G040100        ttgaacagACAAAAGGATGTTGTTGCTGGTGAACTTCCCGTTGCTTTCGT 2928
OAC Rex KF303286               ttgaacagACAAAAGGATGTTGTTGCTGGTGAACTTCCCGTTGCTTTTGT 2990
                               *********************************************** **

G19833 Phvul.002G040100        TGTGAGATCAAACGGCTTTGATCTGACAGAAGAGGCTGTAAAAGAGTTTA 2978
OAC Rex KF303286               TGTGAGATCAAACGGCTTTGATCTGACAGAAGAGGCTGTAAAAGAGTTTA 3040
                               **************************************************
                                                                                 INTRON 4
G19833 Phvul.002G040100        TAGCTAAACAGgtatgagtgtgacgataaatctaacttcatttatgtctc 3028
OAC Rex KF303286               TAGCTAAACAGgtatgagtgtgacgataaatctaacttcatttatgtctc 3090
                               **************************************************

G19833 Phvul.002G040100        atcaagtgtttgagaaatttcatgagagcttaatcttgaagagggttttg 3078
OAC Rex KF303286               atcaagtgtttgagaaatttcatgagagcttaatcttgaagagggttttg 3140
```

**************************************************

```
                                                                                 EXON 5
G19833 Phvul.002G040100        attagtgtaattgaatttgttctgaacagGTAGTGTTTTACAAAAGACTG 3128
OAC Rex KF303286               attagtgtaattgaatttgttctgaacagGTAGTGTTTTACAAAAGACTG 3190
                               **************************************************

G19833 Phvul.002G040100        CACAAAGTTTATTTTGTTCATGCCATTCCCAAGTCTCCATCAGGAAAGAT 3178
OAC Rex KF303286               CACAAAGTTTATTTTGTTCATGCCATTCCCAAGTCTCCATCAGGAAAGAT 3240
                               **************************************************

G19833 Phvul.002G040100        ATTAAGGAAAGACCTGAGAGCAAAGCTGGAAGCCGCCGCCGCCGCCGCCG 3228
OAC Rex KF303286               ATTAAGGAAAGACCTGAGAGCAAAGCTGGAAGCCGCCGCCGCCGCCGCCG 3290
                               **************************************************
                                                TAA
G19833 Phvul.002G040100        ---CCACTCCCACGCCTTAAGGCCAGCACGCCAGAACCTCCTCTTTTTTT 3275
OAC Rex KF303286               CCGCCACTCCCACGCCTTAAGGCCAGCACGCCAGAACCTCCTCTTTTTTT 3340
                                  ***********************************************

G19833 Phvul.002G040100        TCTCCAGATTTTTTATTTAAATTATTACTGGGTTAACACGTGTAACTCAC 3325
OAC Rex KF303286               TCTCCAGATTTTTTATTTAAATTATTATTGGGTTAACACGTGTAACTCAC 3390
                               *************************** **********************

G19833 Phvul.002G040100        GTGTCAATGCTTGAATCACTGTATTCTTATGTCCATTGCCTGAGCTTCTC 3375
OAC Rex KF303286               GTGTCAATGCTTGAATCACTGTATTCTTATGTCCATTGCCTGAGCTTCTC 3440
                               **************************************************

G19833 Phvul.002G040100        CGGGTATTGTAATTTTAGATCAATATTATATTATTTCTGCTATCACATTT 3425
OAC Rex KF303286               CGGGTATTGTAATTTTAGATCAATATTATATTATTTCTGTTATCACATTT 3490
                               *************************************** **********

G19833 Phvul.002G040100        TCCCATTTTCTTCTCCTACCCATCATAAATCACACCA------------- 3462
OAC Rex KF303286               TCCCATTTTCTTCTCCTACCCATCATAAATCATACCATTTTTATTTTATT 3540
                               ******************************** ****


OAC Rex sequence:
>KF303286 OAC Rex gene for 4-coumarate:CoA logase (4CL-1; 3,540 bp); similar to P. vulgaris 4CL locus Phvul.002G040100 (cultivar G19833)
```

AAATGTTAGAGTTGGGGGTAGGTAGGTAAGTAAGAGTGGCTGGAAGAAGAACGCTTGTTCTGAAAACGTGATCCAATGTGTGGCTGCTCCTTCTCCATCATTCATTCACTCACTCATTCTCACCTAACACCCTTTCCCTTCCCTTCTCTATATTACCTTCTTCCCATGTTTCCCTCTCCTCACTCTCTCTCTCTCTCTCTCTCTCCTTAAACTCTTCATTCCACTCTAACTAACACACCCCATTCACCATGCTAAGTGTTGCTCCTTCTCTTGATACCAACACCAAAGACACCAAAACAGACCAAACTCAAATTCCCCAAACTACCCATGTTTTCAAGTCTAAATTACCAGACATACCAATCTCCAACCACCTATCCCTCCATGATTACTGCTTCCAAAACCTCTCCCAATTTGCAGACTGTCCCTGCCTCATCGTCGGCTCCGCCTCCAAAACCTACACCTACGCACAGACGTACCTTATTTCGCGCAAGATCGCTGCCGGATTCTCGAATCTCGGAATCCGCAAGGGCGACGTCGTCATGATCCTCCTCCAGAACTCCGCCGAGTTCGTCCTCTCCTTCCTCGCCGCCTCCATGGTCGGAGCTGTAGCCACCACCGCCAACCCGTTCTACACCGCCGCCGAGATCTTCAAGCAGTTCACCGCCTCCAAGGCGAAGCTGATCATAACGCAGGCAATGTACGTGGAAAAGCTCCGCAGCCACGACGCCGGATGGAGCCTCGGCGAGGACTTCAAGGTCGTGACCGTCGACGATCCGCCGGAGAATTGCCTCCACTTCTCCGTGCTCTCTGAGGCGAACGAGAGCGACGCTCCCGAGGTGGAGATTCATCCGGATGACGCCGTGGCGCTGCCGTTTTCCTCCGGCACCACGGGCCTGCCGAAGGGAGTGATCCTCACGCACAAGAGTTTGACCACGAGCGTGGCGCAGCAGGTTGACGGGGAGAATCCTAATCTCTACCTCACCACCGAGGACGTGCTCCTCTGCGTGCTTCCGCTGTTTCACATATTCTCGCTCAACAGCGTGCTGTTATGCGCGCTTAGAGCCGGGAGCGGCGTTTTGCTTATGCAGAAGTTCGAGATTGGGACGCTGCTGGAGCTTATACAGCGCTACCGCGTGTCGGTGGCGATGGTGGTGCCGCCGCTGGTGCTGGCGCTGGCCAAGAATCCGATGGTGGCGAACTTTGACCTCAGTTCAATACGGTTAGTTCTCTCCGGCGCCGCTCCCTTGGGTAAGGAACTCGAAGAGGCTCTCCACAGCAGGGTGCCTCACGCTGTTTTGGGACAGGTAAATAATTACACCAAAACTAATTATTATTATTATTATTATCTACGTAAAACTCTAATTTAAATGATAAATATAATAATTAAAAATTACCACTATACTATTAAAAAATAATATCAATTAGAAATTATAGCATATAAGAACCATTAAAAAATAATATTAATTAAAAATTAAAATATTTTATAGTATACAAGTATACATAATATATATAATATATAAGTAGATGCGAATTTATTGATTTTATGAGATTAAATTAAATTTAAATTTTATTTTTATTAATATAGTTAAAATTATTTAATATTACGTTAAAGGCAAATTTATAGGAATACAAATGGTCGTTTTCATTATTTATTTCTCATCAACCTTTTGCATGCGATAATGGGCATATATGAAGTTGTATATTAATTAAGAATACCATGGACACGTGTCACCTTCTCCTCCACTGACTGTTATTAATTAAGAATATAAACATATTTTCTTTATATATAGGTGCTGACTTTTAAGAATATACGTCTAAATAAATCATTAAAATATTTCATTATAGCTGTTAAAAAAATATTAAAATCTTTACTTGAGTGTGTGCATGCAAAATTTTATGGTAGTTAGCTCACCAACCACCCCACACCAAACACCGAAACCAAAACGGTGAAATAGAAGCACAAGCATCCAAACAAGACTTTTAGTGACGTGTACAACTTGACACCTTCATTTTCATTGCCATTTGGCACACGTGACGTAAGCCAAACTAACAGTGGACCAGTCAACAATTCACTGCACCTCCCCCTGCAACCTAATTCCTTTCATTTTAAATATTTAAAAAAAAAATTCAAGTAGCACACAGATACATGATATATGAAATCTCAATATAAATATAAGTTTATATATTATATAATTATAAATTAATAATAAATTATATAAATATATTTCAATTTTTTTTAGGAAAATAATATTTCTCATGATTAATTTAAAATAATTTATGGATCTTATGTTGTTGACTTTAAAATGCATGTATGTTACAGGGTTACGGGATGACAGAAGCAGGACCAGTGCTTTCCATGTGTTTGGGGTTTGCAAAGCAACCATTTCCAACAAAATCGGGATCTTGTGGAACTGTTGTGAGAAATGCAGAACTAAAGGTTGTTGACCCTGAAACTGGTCGTTCTCTCGGCTACAATCAGCCCGGTGAAATTTGCATCCGAGGGCTACAGATCATGAAAGGATATCTGAACGATGATCAAGCCACAGCATCCACCATCGATGCAGAGGGTTGGCTTCACACCGGTGATGTTGGCTATGTAGACGATGACGATGAAATTTTCATCGTTGACAGGGTCAAAGAACTCATCAAATTTAAAGGCTTCCAGGTATATATATATATACACACACATATATAAACATCTAAGTTAAATTTGCCTAAAAGTTTCAGAGAAAAAAAAATTGTGACAATGACAAGTGATTTTGTTTTTGCCAGGTGCCCCCTGCAGAACTTGAAGGGCTTCTTGTAAGCCATCCCTCCATTGCAGACGCAGCAGTTGTCCCGTAAGCTTCTTAATCTTCTGAATTTGAATGACTTACATTGTGCATATATATATATATATATATGTATGTATGTATGTAACTGACCATTAATTTGATCGATTTGAACAGACAAAAGGATGTTGTTGCTGGTGAACTTCCCGTTGCTTTTGTTGTGAGATCAAACGGCTTTGATCTGACAGAAGAGGCTGTAAAAGAGTTTATAGCTAAACAGGTATGAGTGTGACGATAAATCTAACTTCATTTATGTCTCATCAAGTGTTTGAGAAATTTCATGAGAGCTTAATCTTGAAGAGGGTTTTGATTAGTGTAATTGAATTTGTTCTGAACAGGTAGTGTTTTACAAAAGACTGCACAAAGTTTATTTTGTTCATGCCATTCCCAAGTCTCCATCAGGAAAGATATTAAGGAAAGACCTGAGAGCAAAGCTGGAAGCCGCCGCCGCCGCCGCCGCCGCCACTCCCACGCCTTAAGGCCAGCACGCCAGAACCTCCTCTTTTTTTTCTCCAGATTTTTTATTTAAATTATTATTGGGTTAACACGTGTAACTCACGTGTCAATGCTTGAATCACTGTATTCTTATGTCCATTGCCTGAGCTTCTCCGGGTATTGTAATTTTAGATCAATATTATATTATTTCTGTTATCACATTTTCCCATTTTCTTCTCCTACCCATCATAAATCATACCATTTTTATTTTATT

```
B) Nucleotide sequence comparison of the 4CL locus Phvul.003G146900 between two common bean genotypes

G19833 Phvul.003G146900      ---GGCAATGAACTCTATTTCTAGCTGTTCCACCGTAGAACTACGGATTC 47
OAC Rex KF303287             GATGGCAATGAACTCTATTTCTAGCTGCTCCACCGTAGAACTACGGATTC 50
                                ************************ **********************

G19833 Phvul.003G146900      TACACTAACATGTCCAACTGATCCAAACCAAATCTGCAAAAGAAAAGCAG 97
OAC Rex KF303287             TACACTAACATGTCCAACTGATCCAAACCAAATCTGCAAAAGAAAAGCAG 100
                             **************************************************

G19833 Phvul.003G146900      AGAAAGCATCGTGACGCAAGAAACTACAACCCACATAACTCATCAACAAC 147
OAC Rex KF303287             AGAAAGCATCGTGACGCAAGAAACTAGAACCCACATAACTCATCAACAAC 150
                             ************************** ***********************

G19833 Phvul.003G146900      TTCCTCATCACATTCTTTTCCACTCTACTTTCTCATCTTTCACTCCCATT 197
OAC Rex KF303287             TTCCTCATCACATTCTTTTCCACTCTACTTTCTCATCTTTCACTCCCATT 200
                             **************************************************
                                                                           ATG EXON 1
G19833 Phvul.003G146900      CACACTCTCCACCACCCAAACCAACAACGTAGCAACCAAAACCAAAATGG 247
OAC Rex KF303287             CACACTCTCCACCACCCAAACCAACAACGTAGCAACCAAAACCAAAATGG 250
                             **************************************************

G19833 Phvul.003G146900      AACAACAACAATCTACACAACTTCACAATGATGACTTCATTTTCCGTTCC 297
OAC Rex KF303287             AACAACAACAATCTACACAACTTCACAATGATGACTTCATTTTCCGTTCC 300
                             **************************************************

G19833 Phvul.003G146900      AAACTCCCCGACATTTACATCCCCACGCACCTCCCTCTCCACACTTACAT 347
OAC Rex KF303287             AAACTCCCCGACATTTACATCCCCACGCACCTCCCTCTCCACACTTACAT 350
                             **************************************************

G19833 Phvul.003G146900      CTTCCAAAATATCTCCCAATTCAGCCACCGACCCTGCCTCATCAACGCCG 397
OAC Rex KF303287             CTTCCAAAACATCTCCCAATTCAGCCACCGACCCTGCCTCATCAACGCCG 400
                             ********* ****************************************

G19833 Phvul.003G146900      CCACCGGAGACTGCTTCACCTACGCCGAGGTCCAACTCACCTCTCGCAGA 447
OAC Rex KF303287             CCACCGGAGACTGCTTCACCTACGCCGAGGTCCAACTCACCTCTCGCAGA 450
                             **************************************************

G19833 Phvul.003G146900      GTCGCCGCCGGCCTCAACAAACTCGGCATCGAGAAGGGCGACGTCATCCT 497
OAC Rex KF303287             GTCGCCGCCGGCCTCAACAAACTCGACATCCAGAAGGGCGACGTCATCCT 500
                             ************************* **** *******************

G19833 Phvul.003G146900      CCTTCTCCTCCAGAACTGCCCCCAGTTCGTCTTCGCATTCCTCGGCTCCT 547
OAC Rex KF303287             CCTTCTCCTCCAGAACTGCCCCCAGTTCGTCTTCGCATTCCTCGGCTCCT 550
                             **************************************************

G19833 Phvul.003G146900      CCTTCTGTGGCGCCACTGTGACCACCGCTAACCCCTTCTACACTCCGGCG 597
OAC Rex KF303287             CCTTCTGTGGCGCCACTGTGACCACCGCTAACCCCTTCTACACTCCGGCG 600
                             **************************************************

G19833 Phvul.003G146900      GAGGTGGCCAAGCAAGCCGCGGCGTCCAACGCCAAACTCATCATAACGCA 647
OAC Rex KF303287             GAGGTGGCCAAGCAAGCCGCGGCGTCCAACGCCAAACTCATCATAACGCA 650
                             **************************************************

G19833 Phvul.003G146900      GGCGTCCTACGTGGACAAAGTAAAGGATTACGCGAGGGAAAAGGACGTCA 697
OAC Rex KF303287             GGCGTCCTACGTGGACAAAGTAAAGGATTACGCGAGGGAAAAGGACGTCA 700
                             **************************************************
                                                        TSR  TG
G19833 Phvul.003G146900      GAGTGATGTGCGTTGACGCGGCGCCGGAAGGTT----------------- 730
OAC Rex KF303287             GAGTGATGTGCGTTGACACGGCGCCGGAAGGTTGTAAAAAATAGACACTC 750 5’LTR
                             ***************** ***************                   (733-1,030)

G19833 Phvul.003G146900      --------------------------------------------------
OAC Rex KF303287             ACTCACACTTAAATTCTCTCTAAAAGAAACACTCTTCTTTGCAATTCTCT 800

G19833 Phvul.003G146900      --------------------------------------------------
OAC Rex KF303287             CTATTGTATTTCTTTCTGTGGTGTATCTTACAATTGAGAGAGCATCCTAT 850

G19833 Phvul.003G146900      --------------------------------------------------
OAC Rex KF303287             TTATAGGCTTTAGGAAGCTCTTCTAGAAAAGTCTTCATTATGGCCTTAAA 900

G19833 Phvul.003G146900      --------------------------------------------------
OAC Rex Contig23329904CL     ACCCCACTAACAACACAATTAAAATCCCACTCACAACTTTTGACCTTTTA 950

G19833 Phvul.003G146900      --------------------------------------------------
OAC Rex KF303287             CATATCCAAACTCCCATTCTACTCTTTTCTAGTAACTTCTAACTTTAAAA 1000
                                                         CA
G19833 Phvul.003G146900      --------------------------------------------------
OAC Rex KF303287             CCCACAAATTACATTAAAGTTTATTCAACAAAACTCCCCCATAAACTTAA 1050
                                TTTCTACCATCCATC - PPT (polypurine tract)
G19833 Phvul.003G146900      --------------------------------------------------
OAC Rex KF303287             ATGTTTCTACCATCCATCATGCCAATCATCTTCTTGAATTTGTCGAAAAA 1100
                                                                              IN (integrase)
G19833 Phvul.003G146900      --------------------------------------------------(1,047-5,219)
OAC Rex KF303287             GACTTTCGGTAGCGGTTTTGTGAAGATATCAGCAACTTGATCTTGACTTG 1150

G19833 Phvul.003G146900      --------------------------------------------------
OAC Rex KF303287             CCACATGCACCAATTCCACAATTCCTTTCTTCACATGATCACGAATAAAG 1200

G19833 Phvul.003G146900      --------------------------------------------------
OAC Rex KF303287             TGAAAACGAACGTCAATATGTTTGCTTCTTTCATGGTTCACTGGGTTCTT 1250

G19833 Phvul.003G146900      --------------------------------------------------
OAC Rex KF303287             TGCCAACTCAATTGCTGATTTGTTGTCAACTTGAATCACAGTTGCATCTA 1300

G19833 Phvul.003G146900      --------------------------------------------------
OAC Rex KF303287             GTTGCTTTAGCTCCATCTTACTCAACAAGTTTCTTAGCCATATCGCATGA 1350

G19833 Phvul.003G146900      --------------------------------------------------
OAC Rex KF303287             CATACACACCAAGATGCTGCCACATATTCAGCTTCACACGTCGATAGCGT 1400

G19833 Phvul.003G146900      --------------------------------------------------
OAC Rex KF303287             CACGATCGGTTGCTTCTTTGAAAGCCAAGAAAATGCTGTGTTGCCCATAA 1450

G19833 Phvul.003G146900      --------------------------------------------------
OAC Rex KF303287             AGAATACATATCCCGATGTACTTTTCCGATCATCTATGTCTCCGCACCAA 1500

G19833 Phvul.003G146900      --------------------------------------------------
OAC Rex KF303287             TCGCTGTCAGAGTAACCCACCAACTTGTAATCTTCTGCTTTTGAGTAGAA 1550

G19833 Phvul.003G146900      --------------------------------------------------
OAC Rex KF303287             CAATCCGAGTGATACAGTACCTTGGATGTACCGTAGGACTCGCTTCAACG 1600

G19833 Phvul.003G146900      --------------------------------------------------
OAC Rex KF303287             CCTTCCAATGTGAGTAAACCGGTTCCTCCATGAATCGACTTATGATGCCG 1650

G19833 Phvul.003G146900      --------------------------------------------------
OAC Rex KF303287             ACACTTAATGAAAGATCCGGCCTTGTGCATGTGAGATAACGAAGGCTTCC 1700

G19833 Phvul.003G146900      --------------------------------------------------
OAC Rex KF303287             TACCAAACTCCGGTATCTACTTGCATCGACACGTTCTCCTCCATCGAACT 1750

G19833 Phvul.003G146900      --------------------------------------------------
OAC Rex KF303287             TTGAGAGTTTTGCACCTGGTTCCATTGGTATTGATACTGGGTTGCAATTT 1800

G19833 Phvul.003G146900      --------------------------------------------------
OAC Rex KF303287             TCCATCTTGTACTTCTTCAAAATCTCCTTTGCATATTTCTCCTGTGATAC 1850

G19833 Phvul.003G146900      --------------------------------------------------
OAC Rex KF303287             AAAAATACCTGTCTCTTTTTGTCTAACTTCCAAGCCAAGGAAAAACTTCA 1900

G19833 Phvul.003G146900      --------------------------------------------------
OAC Rex KF303287             TCAATCCCAAATCTGTCATCTCAAATTCCCGTCTCATTGTGCCCTTAAAC 1950

G19833 Phvul.003G146900      --------------------------------------------------
OAC Rex KF303287             TCTTCTATCATATCATTATTGTTACCCATAAAAATGAGATCATCAACATA 2000

G19833 Phvul.003G146900      --------------------------------------------------
OAC Rex KF303287             AAGAGCAACAAATATCATGTTACCTCCATTGTTCTTTGCGTAAAGAGCAT 2050

G19833 Phvul.003G146900      --------------------------------------------------
OAC Rex KF303287             GCTCGTAAGGACATTGCTTGAACCCGTTCTCCTTGAAGTATGTATCGATA 2100

G19833 Phvul.003G146900      --------------------------------------------------
OAC Rex KF303287             CGAGTATTCCATGCCCGCGGTGCTTGCTTCAACCCGTAAAGTGCCTTCTT 2150

G19833 Phvul.003G146900      --------------------------------------------------
OAC Rex KF303287             CAATTTTAGCACCTTCTTCTCCTCTCCGATTTTCATGTACCCAGGTGGTT 2200

G19833 Phvul.003G146900      --------------------------------------------------
OAC Rex KF303287             GTTCGATGTAAACTTCTTCTTCAAGCACACCATTCAAGAATGCCGACTTG 2250

G19833 Phvul.003G146900      --------------------------------------------------
OAC Rex KF303287             ACATCCATTTGAAATATCGGCCATTTAAATTGAGCCGCTTGGGAAATGAG 2300

G19833 Phvul.003G146900      --------------------------------------------------
OAC Rex KF303287             CAATCGAATTGTCTCCATTCTTACAACGGGAGCAAACACCTCATCGTAGT 2350

G19833 Phvul.003G146900      --------------------------------------------------
OAC Rex KF303287             CAATTCCCTCCTTTTGTTTGTATCCCTTCGCAACGAGTCGCGCCTTGTAC 2400

G19833 Phvul.003G146900      --------------------------------------------------
OAC Rex KF303287             CTCTCTATCTCGCCTTGAGCATTCATCTTTTTCTTGAATATCCACTTCAC 2450

G19833 Phvul.003G146900      --------------------------------------------------
OAC Rex KF303287             ACCAATGGGCTGACTTCCCTCTGGCAATTCTGTTAGCTCCCATGTGTTGT 2500

G19833 Phvul.003G146900      --------------------------------------------------
OAC Rex KF303287             TGCGGTCAATCGCTTTAATCTCCTCATCCATGGCAGTTTGCCACTTCTTG 2550

G19833 Phvul.003G146900      --------------------------------------------------
OAC Rex KF303287             TCTCGCACCGCCTCTTCAAAGCTGATATTTTCAGCATCTGCCAAAAGACA 2600

G19833 Phvul.003G146900      --------------------------------------------------
OAC Rex KF303287             TACTAGGTGTACCTCATTTGTCGAATCATACAAATCATGCAAACTTCGAA 2650

G19833 Phvul.003G146900      --------------------------------------------------
OAC Rex KF303287             TTTTAGGTTGTCGTGGTTCATCTTCATCATCGGTTGTTTCGGAATTTATA 2700

G19833 Phvul.003G146900      --------------------------------------------------
OAC Rex KF303287             CTTGTCGGTGATGATTCTCCAACTTCAATCATAACCTCTGAAGAATTGTT 2750

G19833 Phvul.003G146900      --------------------------------------------------
OAC Rex KF303287             CCAATCCCACTCGCTTGCTTCATTTATTTGCACATCACGACTCACCGTCA 2800

G19833 Phvul.003G146900      --------------------------------------------------
OAC Rex KF303287             CCTTCTTGCTTATTGGATCAAGTAGCTTGTACCCTTTTGTCTTCTCATCA 2850

G19833 Phvul.003G146900      --------------------------------------------------
OAC Rex KF303287             TACCCAATAAAAACATACCTTTTGCTTTTGTCTTCGAGCTTCGTTCTTCG 2900

G19833 Phvul.003G146900      --------------------------------------------------
OAC Rex KF303287             TTGATCTGGTACGTGTGCATAAGCCACACTACCAAACACTTTGAGATGAG 2950

G19833 Phvul.003G146900      --------------------------------------------------
OAC Rex KF303287             AAACTGTTGGCTTTTGTCCGCTCCATGCCTCTTGTGGTGTTTGATCATCT 3000

G19833 Phvul.003G146900      --------------------------------------------------
OAC Rex KF303287             AACTTCACATGTGGACATCGATTTTGCACATAGATGGCGCATTGCACAGC 3050

G19833 Phvul.003G146900      --------------------------------------------------
OAC Rex KF303287             TTCCGCCCAAAATTCCTTTGGCATCTTTTTGCTCTTAAGCATTGATCGAA 3100

G19833 Phvul.003G146900      --------------------------------------------------
OAC Rex KF303287             CCATGTCGAGAATGGTCCGATTCTTCCTCTCGGCCACACCATTTTGTTGA 3150

G19833 Phvul.003G146900      --------------------------------------------------
OAC Rex KF303287             GGAGTGTATGGTGCAGTTAGAAATCGCCTTATGCCTTGCTCCTCGCAATA 3200

G19833 Phvul.003G146900      --------------------------------------------------
OAC Rex KF303287             CTCCATGAAAGTCGTTGAAGTATACTCGCCACCTCTATCAGATCGTACAG 3250

G19833 Phvul.003G146900      --------------------------------------------------
OAC Rex KF303287             CTTTGATCTGTTTATCAGTTGTCCTCTCCACCATCACCTTGAACTTTTTG 3300

G19833 Phvul.003G146900      --------------------------------------------------
OAC Rex KF303287             AACACCTCAAATGCCTCGGATTTTTCTTTCAGAAAGTAAACCCAAGTTTT 3350

G19833 Phvul.003G146900      --------------------------------------------------
OAC Rex KF303287             CCGTGAGAAATCATCAATGAAGGTAATGAAATACCTTTTACCGCTGAAGG 3400

G19833 Phvul.003G146900      --------------------------------------------------
OAC Rex KF303287             ACTCTGGAGTAATTGGTCCACATATGTCGGTATGAATCAATTCGAGAGGT 3450

G19833 Phvul.003G146900      --------------------------------------------------
OAC Rex KF303287             TGCTTAGCCCAATATTGAGCCTTTTTTGGAAACGAGGTTCTCACATGCTT 3500

G19833 Phvul.003G146900      --------------------------------------------------
OAC Rex KF303287             GCTGAGCACACATTCTTCACAAAATTTTCCCTCGTAGTCCATGTTGGGTA 3550

G19833 Phvul.003G146900      --------------------------------------------------
OAC Rex KF303287             GCCCGTGCACCATATTCTTCTTCGCTAACTCTTTTAGTCCACCATGATGT 3600

G19833 Phvul.003G146900      --------------------------------------------------
OAC Rex KF303287             AGGTGACCAAAACGGAGATGCCACAACGATGCCTTATCTTCTATGTTGAC 3650

G19833 Phvul.003G146900      --------------------------------------------------
OAC Rex KF303287             TTGCAAACATTTTTCTCGTATGCTTCTCAAATTCAGCTTGTACATCCGAT 3700

G19833 Phvul.003G146900      --------------------------------------------------
OAC Rex KF303287             TTCTTGCCATCTCAATTCGGGCAACCAAACACCCTTGCTTGTTCTTCAAG 3750

G19833 Phvul.003G146900      --------------------------------------------------
OAC Rex KF303287             TGTAGGAATCGATCTTTCAAAAATATCGAGTAACCTTTCTCCGTGAGTTG 3800

G19833 Phvul.003G146900      --------------------------------------------------
OAC Rex KF303287             CCCCATACTCAAAATGTTGGTCTTGAGGTCTGGTACGTAATAAACATCTT 3850

G19833 Phvul.003G146900      --------------------------------------------------
OAC Rex KF303287             GGAGTGACCCAATTAAGCCATCCTTTTGTAAGTAGCAAACCGTACCTCGC 3900

G19833 Phvul.003G146900      --------------------------------------------------
OAC Rex KF303287             CCTTTGACCTCCACCTTTGATGCATCTCCAAATGAAACATGACCATCTTC 3950

G19833 Phvul.003G146900      --------------------------------------------------
OAC Rex KF303287             AATCTTTTGCATATCTTTGAATAGGTACTCGTGACCGCACATATGGTTGC 4000

G19833 Phvul.003G146900      --------------------------------------------------
OAC Rex KF303287             TTGCTCCAGAGTCGAGGTACCACAGAGTGTCATTGTTAATGTTGACTTCA 4050

G19833 Phvul.003G146900      --------------------------------------------------
OAC Rex KF303287             TCTTGAGCCATCAAGAGAACACCTTCATTCGTTTCGACTTCCAAGGCTAG 4100

G19833 Phvul.003G146900      --------------------------------------------------
OAC Rex KF303287             GTTGGTTGTTTCTTCTATCTTTATATCAGCACGACAATCTTTTGCAAAAT 4150

G19833 Phvul.003G146900      --------------------------------------------------
OAC Rex KF303287             GCCCCACTTTACCACAATTGTAACATTTGTCAGAGTTACAATCCTTCGCA 4200

G19833 Phvul.003G146900      --------------------------------------------------
OAC Rex KF303287             TAGTGACCATATTTGTGACATTTGTAGCACTCGATGTTGGAATAATTCGA 4250

G19833 Phvul.003G146900      --------------------------------------------------
OAC Rex KF303287             TCGGCCGCCTCTTCCTCTACCACGTCCTCTTCCACGCCAATTTGGTTGGC 4300

G19833 Phvul.003G146900      --------------------------------------------------
OAC Rex KF303287             TCGACTGTTCCTTCTCCTTATAGTACCCTTCATGGTTGCTGCCTTTTCCA 4350

G19833 Phvul.003G146900      --------------------------------------------------
OAC Rex KF303287             CCACGACCGTTTCCACGACTTCCACGACCACGCCCTCTATATTGAGAATT 4400

G19833 Phvul.003G146900      --------------------------------------------------
OAC Rex KF303287             TTGATGATAGAGTACCTTTTCGTCTTTGATTGACGCCTTGGTTTGAAGTG 4450

G19833 Phvul.003G146900      --------------------------------------------------
OAC Rex KF303287             CTTGCTCGAGTGTTTCCTCCTTCTTCTTCTTCTTTCGTTGCTCGTGTGCC 4500

G19833 Phvul.003G146900      --------------------------------------------------
OAC Rex KF303287             TCGAGAGAACCGGCGAGCTCGTCGACTGTGAGCGTCGCAAGGTCCTTTGA 4550

G19833 Phvul.003G146900      --------------------------------------------------
OAC Rex KF303287             CTCTTCTATCGCGCACACAATACTCTCAAAATTGTCTGTTAATGTTCTCA 4600

G19833 Phvul.003G146900      --------------------------------------------------
OAC Rex KF303287             AAATCTTCTCCACAACTCGTGCATCGGTTAGAGTTTCACCGTTTCGGTTG 4650

G19833 Phvul.003G146900      --------------------------------------------------
OAC Rex KF303287             AGTTGATTCACCACAGCCTGTACACGCGTGATGTAGTCAGATACACTTTC 4700

G19833 Phvul.003G146900      --------------------------------------------------
OAC Rex KF303287             TGACTCCATCATCTTCATGTTCTCCAACTCGCCACGCAGAGTTTGTAGAC 4750

G19833 Phvul.003G146900      --------------------------------------------------
OAC Rex KF303287             GCACTTGCTTAACTCGGTCAGCTCCTTTGAACACCTTCTCCAATATGTCC 4800

G19833 Phvul.003G146900      --------------------------------------------------
OAC Rex KF303287             CATGCTTCCTTTGAAGTGGACGCACCAGCAATCTTCTCAAAAATTGCCTC 4850

G19833 Phvul.003G146900      --------------------------------------------------
OAC Rex KF303287             ATCAACAGCTCTATACAACATGTACAAAGCTGCCTTATCTTTCGATCGCA 4900

G19833 Phvul.003G146900      --------------------------------------------------
OAC Rex KF303287             TCTCTTTCAATGCCTTGGTTTGAGCTGCCGTATAACCCGTGGTATTGGTC 4950

G19833 Phvul.003G146900      --------------------------------------------------
OAC Rex KF303287             GGTTCTTCAAAACCTTCTTCGACCACCTCCCATGAATCCTGAGAACCGAG 5000

G19833 Phvul.003G146900      --------------------------------------------------
OAC Rex KF303287             AAGAGCTTTCATTTGAATGCTCCAGTTGTCATATCTGGTCGCCTTCGTTA 5050

G19833 Phvul.003G146900      --------------------------------------------------
OAC Rex KF303287             ACCGTGGTAGCGGTATTTGACCCGTCACATTCGCCATTGGCTCTTGATAC 5100
                                  TG
G19833 Phvul.003G146900      --------------------------------------------------
OAC Rex KF303287             CAAATTGTAAAAAATAGACACTCACTCACACTTAAATTCTCTCTAAAAGA 5150 3’LTR
                                                                               (5,106-5,405)
G19833 Phvul.003G146900      --------------------------------------------------
OAC Rex KF303287             AACACTCTTCTTTGCAATTCTCTCTATTGTATTTCTTTCTGTGGTGTATC 5200

G19833 Phvul.003G146900      --------------------------------------------------
OAC Rex KF303287             TTACAATTGAGAGAGCATCCTATTTATAGGCTTTAGGAAGCTCTTCTAGA 5250

G19833 Phvul.003G146900      --------------------------------------------------
OAC Rex KF303287             AAAGTCTTCATTATGGCCTTAAAACCCCACTAACAACACAATTAAAATCC 5300

G19833 Phvul.003G146900      --------------------------------------------------
OAC Rex KF303287             CACTCACAACTTTTGACCTTTTACATATCCAAACTCCCATTCTACTCTTT 5350

G19833 Phvul.003G146900      --------------------------------------------------
OAC Rex KF303287             TCTAGTAACTTCTAACTTTAAAAAACCCACAAATTACATTAAAGTTTATT 5400

                                CA TSR (target site repeat: AAGGT)
G19833 Phvul.003G146900      -----------ACCTGGCCTTCTCGGATTTAACGGAGGCGAATGAGAGAG 769
OAC Rex KF303287             CAACAAAGGTTACCTGGCCTTCTCGGATTTGACGGAGGCGAATGAGAGAG 5450
                                        ******************* *******************

G19833 Phvul.003G146900      ACATTCCCGGCGTTAAAATATCCCCGGACGACGTAGTTGCGCTGCCGTAT 819
OAC Rex KF303287             ACATTCCCGGCGTTAAAATATCCCCAGACGACGTAGTTGCGCTGCCGTAT 5500
                             ************************* ************************

G19833 Phvul.003G146900      TCTTCAGGAACCACAGGGCTTCCGAAGGGAGTGATGCTAACGCACAAGGG 869
OAC Rex KF303287             TCTTCAGGAACCACAGGGCTTCCGAAGGGAGTGATGCTAACGCACAAGGG 5550
                             **************************************************
                              ATG
G19833 Phvul.003G146900      GATGGTTACGAGCGTGGCGCAACAAGTCGACGGAGAGAACCCTAATCTGT 919
OAC Rex KF303287             GATGGTTACGAGCGTGGCGCAACAAGTCGACGGAGAGAACCCTAATCTGT 5600
                             **************************************************

G19833 Phvul.003G146900      ATCTACGCAGCGAGGACGTGGTGTTGTGCGTGCTTCCACTTTTCCATATT 969
OAC Rex KF303287             ATCTACGCAGCGAGGACGTGGTGTTGTGCGTGCTTCCACTTTTCCATATT 5650
                             **************************************************

G19833 Phvul.003G146900      TATTCGCTCAACACTGTTTTGCTCTGTTCGCTTCGAGTTGGCGCTGCGGT 1019
OAC Rex KF303287             TATTCGCTCAACACTGTTTTGCTCTGTTCGCTTCGAGTTGGCGCTGCCGT 5700
                             *********************************************** **

G19833 Phvul.003G146900      TTTGGTAATGCCCAAATTTGAGATAGCTTCGTTGCTGGAATTGGTCGAGA 1069
OAC Rex KF303287             TTTGGTAATGCCCAAATTTGAGATAGCTTCGTTGCTGGAATTGGTCGAGA 5750
                             **************************************************

G19833 Phvul.003G146900      AATACAAGGTGAGCGTGGCGCCGTTTGTGCCACCCATCATTTTGGCCATT 1119
OAC Rex KF303287             AACACAAGGTGAGCGTGGCGCCGTTTGTGCCACCCATCATTTTGGCCATT 5800
                             ** ***********************************************

G19833 Phvul.003G146900      GCAAAGACTCCAGATTTGGAGCGATACGACCTGTCCTCGATTCGAATGAT 1169
OAC Rex KF303287             GCAAAGACTCCAGATTTGGAGCGATACGACCTGTCCTCGATTCGAATGAT 5850
                             **************************************************

G19833 Phvul.003G146900      TTTGTCTGGCGCCGCTCCTGTGGGAAAGGAACTTGAGGACTCTGTGAGGG 1219
OAC Rex KF303287             TTTGTCTGGCGCCGCTCCTGTGGGAAAGGAACTTGAGGACTCTGTGAGGG 5900
                             **************************************************
                                                                               INTRON 1
G19833 Phvul.003G146900      CCAAACTTCCCCACGCCACACTTGGACAGgtaattatttctgttaattta 1269
OAC Rex KF303287             CCAAACTTCCCCACGCCACACTTGGACAGgtaattatttctgttaattta 5950
                             **************************************************

G19833 Phvul.003G146900      gtattcaaggttttctgatttccagtttcatgtttacctgaagtggtcaa 1319
OAC Rex KF303287             gtattcaaggttttctgatttccagtttcatgtttacctgatgtggacaa 6000
                             ***************************************** **** ***

G19833 Phvul.003G146900      attagtttacttttttagcacccttccgaatac-caaatataaatagtta 1368
OAC Rex KF303287             attagtttacttttttagcacccatccgaatactcaaatataaatagtta 6050
                             *********************** ********* ****************
```

```
G19833 Phvul.003G146900      ttttatttatttgttatgtcatacaaaagtagaatgataaaatagaaagt 1418
OAC Rex KF303287             ttttatttatttattatgtcatacaaaagt---atgataaaatagaaagt 6097
                             ************ *****************   *****************

G19833 Phvul.003G146900      tttgaaaaggtgattttgaaatctgatagcagttccttgtagaagcaact 1468
OAC Rex KF303287             tttgaaaaggtgattttgaaatctgatagcagttccttatagaagcaact 6147
                             ************************************** ***********

G19833 Phvul.003G146900      aactaaatatcagaaagaggaaatgaatagttgcttaattgtaagatata 1518
OAC Rex KF303287             aactaaaaatcagaaagaggaaatgaatagttgtttaattgtaagatata 6197
                             ******* ************************* ****************

G19833 Phvul.003G146900      cgacattaaatttagtgaaaatgatatttaagatggagttgtataatttt 1568
OAC Rex KF303287             cgacattaaatttagtgaaaatgatatttaagatggagttgtataatttt 6247
                             **************************************************

G19833 Phvul.003G146900      attgtgattagtttagaaataaataaataatatgaaaaaatattaaaata 1618
OAC Rex KF303287             attgtgattagtttagaaataaataaataatatgaaaaaatattaaaata 6297
                             **************************************************

G19833 Phvul.003G146900      atagtataaatgtaagtgtatgattatagtggggtatgtttggtaaagtg 1668
OAC Rex KF303287             atagtataaatgtaagtgtatgattatagtggggtatgtttggtaaagtg 6347
                             **************************************************

G19833 Phvul.003G146900      ttttcatcattgggaggaagaatcatagaaagtaacctcccaatgagaaa 1718
OAC Rex KF303287             ttttcatcattgggaggaagaatcatagaa-gtaacctcccaatgagaaa 6396
                             ****************************** *******************
                                                                               EXON 2
G19833 Phvul.003G146900      gaaatggagaggatgaaagaaggcgaacgtgtacatgcagGGGTATGGGA 1768
OAC Rex KF303287             gaaatggagaggatgaaagaaggcgaacgtgtacatgcagGGGTATGGGA 6446
                             **************************************************

G19833 Phvul.003G146900      TGACAGAGGCAGGGCCAGTGCTTTCAATGAGCTTAGCGTTTGCGAAGGAA 1818
OAC Rex KF303287             TGACAGAGGCAGGGCCAGTGCTTTCAATGAGCTTAGCGTTTGCGAAGGAA 6496
                             **************************************************

G19833 Phvul.003G146900      GCAGTGCATGTGAAATCAGGTTCGTGCGGGACCGTAGTAAGAAACGCAGA 1868
OAC Rex KF303287             GCAGTGCATGTGAAATCAGGTTCGTGCGGGACCGTAGTAAGAAACGCAGA 6546
                             **************************************************

G19833 Phvul.003G146900      GATGAAGATCATTGACCCTCACACTGCTGCTTCTCTCCACAGAAATCAAG 1918
OAC Rex KF303287             GATGAAGATCATTGACCCTCACACTGCTGCTTCTCTCCACAGAAATCAAG 6596
                             **************************************************
                                                                               INTRON 2
G19833 Phvul.003G146900      CCGGTGAGATTTGCATCAGAGGCAACCAGATCATGAAAGgtataatttac 1968
OAC Rex KF303287             CCGGTGAGATTTGCATCAGAGGCAACCAGATCATGAAAGgtataatttac 6646
                             **************************************************

G19833 Phvul.003G146900      gttcttttacttattgctgtttttcttattcaaatttattcacataaaca 2018
OAC Rex KF303287             gttcttttacttattgctgcttttcttattcaaatttattcacataaaca 6696
                             ******************* ******************************

G19833 Phvul.003G146900      catttaaaaaaaaatacacagatacataaattgtaattccaatatacaaa 2068
OAC Rex KF303287             catttaaaaaaaaatacacagatacataaattgtaattccaatatacaaa 6746
                             **************************************************

G19833 Phvul.003G146900      aaataaagaacttgtatttttcaattgctcgtccttcatttttattttaa 2118
OAC Rex KF303287             aaataaagaacttgtatttttcaattgctcgtccttcatttttattttaa 6796
                             **************************************************

G19833 Phvul.003G146900      aggctcacgtcaagttgaatactttacttcaatcttttattatcttttac 2168
OAC Rex KF303287             aggctcacgtcaagttgaatactttacttcaatcttttattatcttttac 6846
                             **************************************************
```

```
G19833 Phvul.003G146900      ttttatagaaattgtatttagtatttatgatttttttaaaacttactatt 2218
OAC Rex KF303287             ttttatagaaattgtatttagtatttatgatttttttaaaacttactatt 6896
                             **************************************************

G19833 Phvul.003G146900      tattttttttataaaaattgatttgacatattccacttactttttgaaat 2268
OAC Rex KF303287             tattttttttataaaaattgatttgacatattccacttaatttttgaaat 6946
                             *************************************** **********

G19833 Phvul.003G146900      tatttatgtttctttaatttttatataaacatatttttaaataatttgat 2318
OAC Rex KF303287             tatttatgtttctttaatttttatataaacatatttttaaataatttgat 6996
                             **************************************************

G19833 Phvul.003G146900      taatagttttttttaaaaaaattttgtatgctttataaatttttataaac 2368
OAC Rex KF303287             taatagttttttt--aaaaaaatttgtacgctttataaatttttttt--- 7041
                             *************  ****** ****** *************** *

G19833 Phvul.003G146900      attttttttattttgagat-ttttcttaataatttgattaagaatttttt 2417
OAC Rex KF303287             -ttattttttttttgagatgttttcttaataatttgattaagaatttttt 7090
                              ** ***** ********* ******************************

G19833 Phvul.003G146900      tttttttacttttctttatttttttagtatttatatatacatattttttt 2467
OAC Rex KF303287             tttttttacttttctttatttttttagtatttatatatacatattttttt 7140
                             **************************************************

G19833 Phvul.003G146900      attttgagattattttggataacttgactatgaaaattatacgtggttta 2517
OAC Rex KF303287             attttgagattattttggataatttgactatgaaaattatacgtggttta 7190
                             ********************** ***************************

G19833 Phvul.003G146900      tatttattaaattttttgtgtgaatatttgaaaaaaaatgaaaatatcac 2567
OAC Rex KF303287             tatttattaaattttttgtgtgaatatttgaaaaaaaatgaaaatatcac 7240
                             **************************************************

G19833 Phvul.003G146900      aatatttaaattttaatttatttaccaaattataaaaaaaaaaatcaaat 2617
OAC Rex KF303287             aatatttaaattttaatttatttaccaaattataaaaaaaa--atcaaat 7288
                             *****************************************  *******

G19833 Phvul.003G146900      aaaaaatatgcaaaaatgaaaaaaacaattttaaaaagtaaaaaagaaga 2667
OAC Rex KF303287             aaaaaatatgcaaaaatgaaaaaaacaattttaaaaagtaaaaaagaaga 7338
                             **************************************************

G19833 Phvul.003G146900      agaaatcatatgtattaaaaaaaatcatgtatttaaaacatgacattttg 2717
OAC Rex KF303287             agaaatcatatgtattaaaaaaaatcatgtatttaaaacatgacattttg 7388
                             **************************************************

G19833 Phvul.003G146900      taaaaagataaataaatacagagaagtcgagctttaaaagtatcagacct 2767
OAC Rex KF303287             taaaaagataaataaatacagagaagtcgagctttaaaagtatcagacct 7438
                             **************************************************

G19833 Phvul.003G146900      tgaacttgtaattttaaaacatgacttttttttatatataattaaaagaa 2817
OAC Rex KF303287             tgaacttgtaattttaaaacatgatttttttttatata--attaaaagaa 7486
                             ************************ *************  **********

G19833 Phvul.003G146900      tccatttataagataagtttgaatctgtactgttttttttttatataaat 2867
OAC Rex KF303287             tccatttataagataagtttgaatctgtactgttttttttttatataaat 7536
                             **************************************************

G19833 Phvul.003G146900      aaacatttgataatgtatgagtatatactaatgatttgattgattttaga 2917
OAC Rex KF303287             aaacatttgataatgtatgagtatatactaatgatttgattgattttaga 7586
                             **************************************************

G19833 Phvul.003G146900      aagtatattttgtatgaagttatagataaagtaataattaattttaggta 2967
OAC Rex KF303287             aagtatattttgtatgaagttatagataaagtaataattaattttaggta 7636
                             **************************************************
```

```
G19833 Phvul.003G146900      gttttatatcatttgatactgaaactatgtaagaaatatgaattagagga 3017
OAC Rex KF303287             gttttatatcatttgatactgaatctatgtaagaaatatgaattagagga 7686
                             *********************** **************************
                                                                               EXON 3
G19833 Phvul.003G146900      gtataggaaaagtgcataagcattttttggttagGTTACCTAAATGATGA 3067
OAC Rex KF303287             gtataggaaaagtgcataagcattttttggttagGTTACCTAAATGATGA 7736
                             **************************************************

G19833 Phvul.003G146900      AGAGGCGACAAAAATAACTATAGACAACGATGGATGGTTGCATACGGGTG 3117
OAC Rex KF303287             AGAGGCGACAAAAATAACTATAGACAAAGATGGATGGTTGCATACGGGTG 7786
                             *************************** **********************

G19833 Phvul.003G146900      ATATTGGATACATGGACGATGATGACCAGATTTTCATCGTTGATCGGTTG 3167
OAC Rex KF303287             ATATTGGATACATGGACGATGATGACCAGATTTTCATAGTTGATCGGTTA 7836
                             ************************************* ***********

G19833 Phvul.003G146900      AAGGAGTTGATCAAATACAAAGGATTTCAAGTTGCTCCTGCTGAGCTGGA 3217
OAC Rex KF303287             AAGGAGTTGATCAAATACAAAGGATTTCAAGTTGCTCCTGCTGAGCTGGA 7886
                             **************************************************
                                                                               INTRON 3
G19833 Phvul.003G146900      AGCCATGCTAATTGTCCACCCGAACATTTCTGAGGCTGTTGTTGTGTCgt 3267
OAC Rex KF303287             AGCCATGCTAATTGTCCACCCGAACATTTCTGAGGCTGTTGTTGTGTCgt 7936
                             **************************************************

G19833 Phvul.003G146900      gagttctatatttatcatataatgtcaatttttatcctatcctatttctt 3317
OAC Rex KF303287             gagttctatatttatcatataatgtcaatttttatcctatcctatttctt 7986
                             **************************************************
                                                                               EXON 4
G19833 Phvul.003G146900      tgcttttctctttcaacacaaagtttaatgatcacgttgatttcagCATG 3367
OAC Rex KF303287             tgcttttctctttcaacacaaagtttaatgatcacgttgatttcagCATG 8036
                             **************************************************

G19833 Phvul.003G146900      AAGGATGAAGTTGCAGGAGAGGTTCCAGTTGCATTTGTTGTACTGTCAAA 3417
OAC Rex KF303287             AAGGATGAAGTTGCAGGAGAGGTTCCAGTTGCATTTGTTGTACTGTCAAA 8086
                             **************************************************
                                                                              INTRON 4
G19833 Phvul.003G146900      TGGTTCTAAGATCTCTGAGGAAGAAATCAAACAATACATCTCAAAGCAGg 3467
OAC Rex KF303287             TGGTTCTAAGATCTCTGAGGAAGAAATCAAACAATACATCTCAAAGCAGg 8136
                             **************************************************

G19833 Phvul.003G146900      tattaataattagcatcggacaccccaactaatataattcttattttttt 3517
OAC Rex KF303287             tattaataattagcatcggacaccccaactaatataattcttattttttt 8186
                             **************************************************

G19833 Phvul.003G146900      ttctttatttgtactataaactaaaaaataaaaatgagttaaatatgatt 3567
OAC Rex KF303287             ttctttatttttactataaactaaaaaataaaaatgagttaaatatgatt 8236
                             ********** ***************************************

G19833 Phvul.003G146900      tttttatgtaatttcaaacgaaattaattttcattatttgtagttcaaac 3617
OAC Rex KF303287             tttttatgtaatttcaaacgaaattaattttcattatttgtagttcaaac 8286
                             **************************************************

G19833 Phvul.003G146900      tttaaatatctgagtatagtaaaaaaaactataaaaaataagtctatcaa 3667
OAC Rex KF303287             tttaaatatctgagtatagtaaaaaaaactataaaaaataagtctatcaa 8336
                             **************************************************

G19833 Phvul.003G146900      tttcttttacataataatattattatattggaatttttgatcataggatt 3717
OAC Rex KF303287             tttcttttacataataatattattatattggaatttt-gatcataggggt 8385
                             ************************************* *********  *

G19833 Phvul.003G146900      ttttttt------ctttttaacaaaatggggtatgtttaaaaaaaattac 3761
OAC Rex KF303287             ttttttttttttgctttttaacaaaatgggttatgtttaaaaaaaattac 8435
                             *******      ***************** *******************
```

```
G19833 Phvul.003G146900      aaatttaggttagtcgtgtcaattcggcacgacttcatatgcacaaatcg 3811
OAC Rex KF303287             aaatttaggttagtcgtgtcaattcggcacgacttcatatgcacaaatcg 8485
                             **************************************************

G19833 Phvul.003G146900      tctcaattagacacaactttgtcatgtcataatgaagtcgtgtcaacttg 3861
OAC Rex KF303287             tcccaattagacacgactttgccatgtcataatgaagtcgtgtcaacttg 8535
                             ** *********** ****** ****************************

G19833 Phvul.003G146900      acacgacttttgtcgtgtcatatattttttttaattttattgtttatata 3911
OAC Rex KF303287             acacgacttttgccgtgtcatatattttttttaattttattgtttatata 8585
                             ************ *************************************

G19833 Phvul.003G146900      ttaggtagtaagttatgtaatgttaaaaattaatttgatacataattttt 3961
OAC Rex KF303287             ttgggtagtaagttatgtaatgttaaaaattaatttgatacataattttt 8635
                             ** ***********************************************

G19833 Phvul.003G146900      taagaatgttagttttaaggaacaaaaaattggataatcatgtatggatc 4011
OAC Rex KF303287             taagaatgttagttttaaggaacaaaaaattggataatcatgtatggatc 8685
                             **************************************************

G19833 Phvul.003G146900      atgtttggataatcaacttattcaaactttgagtcattaactccttagga 4061
OAC Rex KF303287             atgtttggataatcagcttattcaaactttgagtcattaactccttagga 8735
                             *************** **********************************

G19833 Phvul.003G146900      cattgccctcgaacatggtttctcactagccaaaaagtatattttgatat 4111
OAC Rex KF303287             cattgccctcggacatggtttcccactagccaaaaagtatattttgatat 8785
                             *********** ********** ***************************

G19833 Phvul.003G146900      ttaatgtctcatgtatgcaaattttttgatattttgatatttaatgacgt 4161
OAC Rex KF303287             ttaatgtctcatgtatgcaatttttttgatattttgatatttaatggcgt 8835
                             ******************** ************************* ***

G19833 Phvul.003G146900      cggacatgatccatacatgattatccaatcttttgttccttaaaactaac 4211
OAC Rex KF303287             cggacatgatccatacatgattatccaattttttgttccttaaaactaac 8885
                             ***************************** ********************

G19833 Phvul.003G146900      acttttagaaaattatgtatcaaattaatttttaacattacataacttac 4261
OAC Rex KF303287             acttttagaaaattatgtataaaattaatttttaacattacataacttac 8935
                             ******************** *****************************

G19833 Phvul.003G146900      tacccaatatataaacaataaaatttaaaaaaaattaaaaaatatgacgt 4311
OAC Rex KF303287             tacccaatatataaacaataaaatttaaaaaaaaataaaaaatatgacgt 8985
                             ********************************** ***************

G19833 Phvul.003G146900      ggcagaagtcgtgccaagttgacacgatttcaatatgacagggcagaaat 4361
OAC Rex KF303287             ggcagaagtcgtgccaagttgacacgatttcaatatgacagggcagaaat 9035
                             **************************************************

G19833 Phvul.003G146900      catgccaagttggcacgactttagtatgacatggcagagtcgtgttaaat 4411
OAC Rex KF303287             catgccaagttggcacgactttagtatgacatggcagaatcgtattaaat 9085
                             ************************************** **** ******

G19833 Phvul.003G146900      tagaacgatttgtgcatatgaagttgtgccgaattggtacgacttgccta 4461
OAC Rex KF303287             tggaacgatttgtgcatatgaagtcgtgccaaattggtacgacttgccta 9135
                             * ********************** ***** *******************

G19833 Phvul.003G146900      aatttgtaatttt---------actccatttttttaaaaagcaaaaaaaa 4502
OAC Rex KF303287             aatttgtaatttttttaaacagactccatttttttt-----taaaaaaaa 9180
                             *************         *************       ********

G19833 Phvul.003G146900      aaaacctcatcatgataaaaaccctattatatgggtgttaacatgtttct 4552
OAC Rex KF303287             ataacctcatcataataaaaaccctattatattcgtgttaacatgtctct 9230
                             * *********** ******************  ************ ***
```

```
G19833 Phvul.003G146900      tcctttattataacacaatttatgtaaaagaacactcgactcaatttaat 4602
OAC Rex KF303287             tcctttattataacacaatttacgtaaaagaacactcgactcaatttaat 9280
                             ********************** ***************************

G19833 Phvul.003G146900      agtatttttactacatatatccagtaagtaaaatgtttgaattagaaaaa 4652
OAC Rex KF303287             agtatttttactacatatatccagcaagtaaaatgtttgaattagaaaaa 9330
                             ************************ *************************

G19833 Phvul.003G146900      aaaaatcactttcgtagtatgatataaggataaaaagatatttaacccat 4702
OAC Rex KF303287             aaaaatcactttcgtagtatgatataaggataaaaagatatttaacccat 9380
                             **************************************************

G19833 Phvul.003G146900      ataaatattataatattatatagtgttaaatgatgcaaaagcttctaaaa 4752
OAC Rex KF303287             ataaatattataatattatatagtgttaaatgatgtaaaagcttctaa-- 9428
                             *********************************** ************
                                                                               EXON 5
G19833 Phvul.003G146900      gtttctaaatgcaaaagcaaatttgtatattttggtggcagGTTGTGTTT 4802
OAC Rex KF303287             --------atgcaaaagcaaatttgtatattttggtggcagGTTGTGTTT 9470
                                     ******************************************

G19833 Phvul.003G146900      TACAAAAGAATCAGTAGGGTTTTCTTTGTGGGTTCAATTCCAAAGGCAGC 4852
OAC Rex KF303287             TACAAAAGAATCAGTAGGGTTTTCTTTGTGGGTTCAATTCCAAAGGCAGC 9520
                             **************************************************

G19833 Phvul.003G146900      CTCTGGAAAGATCTTGCGAAGAGAGCTGAGGGCAAGGCTTGCAAACGGTC 4902
OAC Rex KF303287             CTCTGGAAAGATCTTGCGAAGAGAGCTGAGGGCAAGGCTTGCAAACGGTC 9570
                             **************************************************
                                        TGA
G19833 Phvul.003G146900      TTCCTTCGTCGTGAATATATGGGGTACAACCTAATTCAGTCATTTTTGCT 4952
OAC Rex KF303287             TTCCTTCGTCGTGAATATATGGGGTACAACCTAATTCAGTCATTTTTGCT 9620
                             **************************************************

G19833 Phvul.003G146900      TAAAGGTGCCCATATTTTACAAGCTCAAAATGTTAGTAATAAACTTAAAT 5002
OAC Rex KF303287             TAA-GGTGCCCATATTTTACAAGCTCAAAATGTTAGTAATAAACTTAAAT 9669
                             *** **********************************************

G19833 Phvul.003G146900      AAGTTGTTTATTTTATTTTATAAATTTTATAAAA---------------- 5036
OAC Rex KF303287             AAGTTGTTTATTTTATTTTATAAATTTAATAAAATATACACTACAATAAA 9719
                             *************************** ******

G19833 Phvul.003G146900      --------------------------------------------------
OAC Rex KF303287             ATTATTAAATAGAAATTAATTTTAAAGAAAGAAATAATTAATTATTATAA 9769

G19833 Phvul.003G146900      -------------------------------
OAC Rex KF303287             TAATTAAATTAAAAATTAAAATAATTTTTTA 9800


OAC Rex sequence:
>KF303287 OAC Rex gene for 4-coumarate:CoA ligase (4CL-2; 9,800 bp) interrupted by LTR-RT in exon 1 at the position 733..5,411; similar to P. vulgaris 4CL locus Phvul.003G146900 (cultivar G19833)
```

GATGGCAATGAACTCTATTTCTAGCTGCTCCACCGTAGAACTACGGATTCTACACTAACATGTCCAACTGATCCAAACCAAATCTGCAAAAGAAAAGCAGAGAAAGCATCGTGACGCAAGAAACTAGAACCCACATAACTCATCAACAACTTCCTCATCACATTCTTTTCCACTCTACTTTCTCATCTTTCACTCCCATTCACACTCTCCACCACCCAAACCAACAACGTAGCAACCAAAACCAAAATGGAACAACAACAATCTACACAACTTCACAATGATGACTTCATTTTCCGTTCCAAACTCCCCGACATTTACATCCCCACGCACCTCCCTCTCCACACTTACATCTTCCAAAACATCTCCCAATTCAGCCACCGACCCTGCCTCATCAACGCCGCCACCGGAGACTGCTTCACCTACGCCGAGGTCCAACTCACCTCTCGCAGAGTCGCCGCCGGCCTCAACAAACTCGACATCCAGAAGGGCGACGTCATCCTCCTTCTCCTCCAGAACTGCCCCCAGTTCGTCTTCGCATTCCTCGGCTCCTCCTTCTGTGGCGCCACTGTGACCACCGCTAACCCCTTCTACACTCCGGCGGAGGTGGCCAAGCAAGCCGCGGCGTCCAACGCCAAACTCATCATAACGCAGGCGTCCTACGTGGACAAAGTAAAGGATTACGCGAGGGAAAAGGACGTCAGAGTGATGTGCGTTGACACGGCGCCGGAAGGTTGTAAAAAATAGACACTCACTCACACTTAAATTCTCTCTAAAAGAAACACTCTTCTTTGCAATTCTCTCTATTGTATTTCTTTCTGTGGTGTATCTTACAATTGAGAGAGCATCCTATTTATAGGCTTTAGGAAGCTCTTCTAGAAAAGTCTTCATTATGGCCTTAAAACCCCACTAACAACACAATTAAAATCCCACTCACAACTTTTGACCTTTTACATATCCAAACTCCCATTCTACTCTTTTCTAGTAACTTCTAACTTTAAAACCCACAAATTACATTAAAGTTTATTCAACAAAACTCCCCCATAAACTTAAATGTTTCTACCATCCATCATGCCAATCATCTTCTTGAATTTGTCGAAAAAGACTTTCGGTAGCGGTTTTGTGAAGATATCAGCAACTTGATCTTGACTTGCCACATGCACCAATTCCACAATTCCTTTCTTCACATGATCACGAATAAAGTGAAAACGAACGTCAATATGTTTGCTTCTTTCATGGTTCACTGGGTTCTTTGCCAACTCAATTGCTGATTTGTTGTCAACTTGAATCACAGTTGCATCTAGTTGCTTTAGCTCCATCTTACTCAACAAGTTTCTTAGCCATATCGCATGACATACACACCAAGATGCTGCCACATATTCAGCTTCACACGTCGATAGCGTCACGATCGGTTGCTTCTTTGAAAGCCAAGAAAATGCTGTGTTGCCCATAAAGAATACATATCCCGATGTACTTTTCCGATCATCTATGTCTCCGCACCAATCGCTGTCAGAGTAACCCACCAACTTGTAATCTTCTGCTTTTGAGTAGAACAATCCGAGTGATACAGTACCTTGGATGTACCGTAGGACTCGCTTCAACGCCTTCCAATGTGAGTAAACCGGTTCCTCCATGAATCGACTTATGATGCCGACACTTAATGAAAGATCCGGCCTTGTGCATGTGAGATAACGAAGGCTTCCTACCAAACTCCGGTATCTACTTGCATCGACACGTTCTCCTCCATCGAACTTTGAGAGTTTTGCACCTGGTTCCATTGGTATTGATACTGGGTTGCAATTTTCCATCTTGTACTTCTTCAAAATCTCCTTTGCATATTTCTCCTGTGATACAAAAATACCTGTCTCTTTTTGTCTAACTTCCAAGCCAAGGAAAAACTTCATCAATCCCAAATCTGTCATCTCAAATTCCCGTCTCATTGTGCCCTTAAACTCTTCTATCATATCATTATTGTTACCCATAAAAATGAGATCATCAACATAAAGAGCAACAAATATCATGTTACCTCCATTGTTCTTTGCGTAAAGAGCATGCTCGTAAGGACATTGCTTGAACCCGTTCTCCTTGAAGTATGTATCGATACGAGTATTCCATGCCCGCGGTGCTTGCTTCAACCCGTAAAGTGCCTTCTTCAATTTTAGCACCTTCTTCTCCTCTCCGATTTTCATGTACCCAGGTGGTTGTTCGATGTAAACTTCTTCTTCAAGCACACCATTCAAGAATGCCGACTTGACATCCATTTGAAATATCGGCCATTTAAATTGAGCCGCTTGGGAAATGAGCAATCGAATTGTCTCCATTCTTACAACGGGAGCAAACACCTCATCGTAGTCAATTCCCTCCTTTTGTTTGTATCCCTTCGCAACGAGTCGCGCCTTGTACCTCTCTATCTCGCCTTGAGCATTCATCTTTTTCTTGAATATCCACTTCACACCAATGGGCTGACTTCCCTCTGGCAATTCTGTTAGCTCCCATGTGTTGTTGCGGTCAATCGCTTTAATCTCCTCATCCATGGCAGTTTGCCACTTCTTGTCTCGCACCGCCTCTTCAAAGCTGATATTTTCAGCATCTGCCAAAAGACATACTAGGTGTACCTCATTTGTCGAATCATACAAATCATGCAAACTTCGAATTTTAGGTTGTCGTGGTTCATCTTCATCATCGGTTGTTTCGGAATTTATACTTGTCGGTGATGATTCTCCAACTTCAATCATAACCTCTGAAGAATTGTTCCAATCCCACTCGCTTGCTTCATTTATTTGCACATCACGACTCACCGTCACCTTCTTGCTTATTGGATCAAGTAGCTTGTACCCTTTTGTCTTCTCATCATACCCAATAAAAACATACCTTTTGCTTTTGTCTTCGAGCTTCGTTCTTCGTTGATCTGGTACGTGTGCATAAGCCACACTACCAAACACTTTGAGATGAGAAACTGTTGGCTTTTGTCCGCTCCATGCCTCTTGTGGTGTTTGATCATCTAACTTCACATGTGGACATCGATTTTGCACATAGATGGCGCATTGCACAGCTTCCGCCCAAAATTCCTTTGGCATCTTTTTGCTCTTAAGCATTGATCGAACCATGTCGAGAATGGTCCGATTCTTCCTCTCGGCCACACCATTTTGTTGAGGAGTGTATGGTGCAGTTAGAAATCGCCTTATGCCTTGCTCCTCGCAATACTCCATGAAAGTCGTTGAAGTATACTCGCCACCTCTATCAGATCGTACAGCTTTGATCTGTTTATCAGTTGTCCTCTCCACCATCACCTTGAACTTTTTGAACACCTCAAATGCCTCGGATTTTTCTTTCAGAAAGTAAACCCAAGTTTTCCGTGAGAAATCATCAATGAAGGTAATGAAATACCTTTTACCGCTGAAGGACTCTGGAGTAATTGGTCCACATATGTCGGTATGAATCAATTCGAGAGGTTGCTTAGCCCAATATTGAGCCTTTTTTGGAAACGAGGTTCTCACATGCTTGCTGAGCACACATTCTTCACAAAATTTTCCCTCGTAGTCCATGTTGGGTAGCCCGTGCACCATATTCTTCTTCGCTAACTCTTTTAGTCCACCATGATGTAGGTGACCAAAACGGAGATGCCACAACGATGCCTTATCTTCTATGTTGACTTGCAAACATTTTTCTCGTATGCTTCTCAAATTCAGCTTGTACATCCGATTTCTTGCCATCTCAATTCGGGCAACCAAACACCCTTGCTTGTTCTTCAAGTGTAGGAATCGATCTTTCAAAAATATCGAGTAACCTTTCTCCGTGAGTTGCCCCATACTCAAAATGTTGGTCTTGAGGTCTGGTACGTAATAAACATCTTGGAGTGACCCAATTAAGCCATCCTTTTGTAAGTAGCAAACCGTACCTCGCCCTTTGACCTCCACCTTTGATGCATCTCCAAATGAAACATGACCATCTTCAATCTTTTGCATATCTTTGAATAGGTACTCGTGACCGCACATATGGTTGCTTGCTCCAGAGTCGAGGTACCACAGAGTGTCATTGTTAATGTTGACTTCATCTTGAGCCATCAAGAGAACACCTTCATTCGTTTCGACTTCCAAGGCTAGGTTGGTTGTTTCTTCTATCTTTATATCAGCACGACAATCTTTTGCAAAATGCCCCACTTTACCACAATTGTAACATTTGTCAGAGTTACAATCCTTCGCATAGTGACCATATTTGTGACATTTGTAGCACTCGATGTTGGAATAATTCGATCGGCCGCCTCTTCCTCTACCACGTCCTCTTCCACGCCAATTTGGTTGGCTCGACTGTTCCTTCTCCTTATAGTACCCTTCATGGTTGCTGCCTTTTCCACCACGACCGTTTCCACGACTTCCACGACCACGCCCTCTATATTGAGAATTTTGATGATAGAGTACCTTTTCGTCTTTGATTGACGCCTTGGTTTGAAGTGCTTGCTCGAGTGTTTCCTCCTTCTTCTTCTTCTTTCGTTGCTCGTGTGCCTCGAGAGAACCGGCGAGCTCGTCGACTGTGAGCGTCGCAAGGTCCTTTGACTCTTCTATCGCGCACACAATACTCTCAAAATTGTCTGTTAATGTTCTCAAAATCTTCTCCACAACTCGTGCATCGGTTAGAGTTTCACCGTTTCGGTTGAGTTGATTCACCACAGCCTGTACACGCGTGATGTAGTCAGATACACTTTCTGACTCCATCATCTTCATGTTCTCCAACTCGCCACGCAGAGTTTGTAGACGCACTTGCTTAACTCGGTCAGCTCCTTTGAACACCTTCTCCAATATGTCCCATGCTTCCTTTGAAGTGGACGCACCAGCAATCTTCTCAAAAATTGCCTCATCAACAGCTCTATACAACATGTACAAAGCTGCCTTATCTTTCGATCGCATCTCTTTCAATGCCTTGGTTTGAGCTGCCGTATAACCCGTGGTATTGGTCGGTTCTTCAAAACCTTCTTCGACCACCTCCCATGAATCCTGAGAACCGAGAAGAGCTTTCATTTGAATGCTCCAGTTGTCATATCTGGTCGCCTTCGTTAACCGTGGTAGCGGTATTTGACCCGTCACATTCGCCATTGGCTCTTGATACCAAATTGTAAAAAATAGACACTCACTCACACTTAAATTCTCTCTAAAAGAAACACTCTTCTTTGCAATTCTCTCTATTGTATTTCTTTCTGTGGTGTATCTTACAATTGAGAGAGCATCCTATTTATAGGCTTTAGGAAGCTCTTCTAGAAAAGTCTTCATTATGGCCTTAAAACCCCACTAACAACACAATTAAAATCCCACTCACAACTTTTGACCTTTTACATATCCAAACTCCCATTCTACTCTTTTCTAGTAACTTCTAACTTTAAAAAACCCACAAATTACATTAAAGTTTATTCAACAAAGGTTACCTGGCCTTCTCGGATTTGACGGAGGCGAATGAGAGAGACATTCCCGGCGTTAAAATATCCCCAGACGACGTAGTTGCGCTGCCGTATTCTTCAGGAACCACAGGGCTTCCGAAGGGAGTGATGCTAACGCACAAGGGGATGGTTACGAGCGTGGCGCAACAAGTCGACGGAGAGAACCCTAATCTGTATCTACGCAGCGAGGACGTGGTGTTGTGCGTGCTTCCACTTTTCCATATTTATTCGCTCAACACTGTTTTGCTCTGTTCGCTTCGAGTTGGCGCTGCCGTTTTGGTAATGCCCAAATTTGAGATAGCTTCGTTGCTGGAATTGGTCGAGAAACACAAGGTGAGCGTGGCGCCGTTTGTGCCACCCATCATTTTGGCCATTGCAAAGACTCCAGATTTGGAGCGATACGACCTGTCCTCGATTCGAATGATTTTGTCTGGCGCCGCTCCTGTGGGAAAGGAACTTGAGGACTCTGTGAGGGCCAAACTTCCCCACGCCACACTTGGACAGGTAATTATTTCTGTTAATTTAGTATTCAAGGTTTTCTGATTTCCAGTTTCATGTTTACCTGATGTGGACAAATTAGTTTACTTTTTTAGCACCCATCCGAATACTCAAATATAAATAGTTATTTTATTTATTTATTATGTCATACAAAAGTATGATAAAATAGAAAGTTTTGAAAAGGTGATTTTGAAATCTGATAGCAGTTCCTTATAGAAGCAACTAACTAAAAATCAGAAAGAGGAAATGAATAGTTGTTTAATTGTAAGATATACGACATTAAATTTAGTGAAAATGATATTTAAGATGGAGTTGTATAATTTTATTGTGATTAGTTTAGAAATAAATAAATAATATGAAAAAATATTAAAATAATAGTATAAATGTAAGTGTATGATTATAGTGGGGTATGTTTGGTAAAGTGTTTTCATCATTGGGAGGAAGAATCATAGAAGTAACCTCCCAATGAGAAAGAAATGGAGAGGATGAAAGAAGGCGAACGTGTACATGCAGGGGTATGGGATGACAGAGGCAGGGCCAGTGCTTTCAATGAGCTTAGCGTTTGCGAAGGAAGCAGTGCATGTGAAATCAGGTTCGTGCGGGACCGTAGTAAGAAACGCAGAGATGAAGATCATTGACCCTCACACTGCTGCTTCTCTCCACAGAAATCAAGCCGGTGAGATTTGCATCAGAGGCAACCAGATCATGAAAGGTATAATTTACGTTCTTTTACTTATTGCTGCTTTTCTTATTCAAATTTATTCACATAAACACATTTAAAAAAAAATACACAGATACATAAATTGTAATTCCAATATACAAAAAATAAAGAACTTGTATTTTTCAATTGCTCGTCCTTCATTTTTATTTTAAAGGCTCACGTCAAGTTGAATACTTTACTTCAATCTTTTATTATCTTTTACTTTTATAGAAATTGTATTTAGTATTTATGATTTTTTTAAAACTTACTATTTATTTTTTTTATAAAAATTGATTTGACATATTCCACTTAATTTTTGAAATTATTTATGTTTCTTTAATTTTTATATAAACATATTTTTAAATAATTTGATTAATAGTTTTTTTAAAAAAATTTGTACGCTTTATAAATTTTTTTTTTATTTTTTTTTTGAGATGTTTTCTTAATAATTTGATTAAGAATTTTTTTTTTTTTACTTTTCTTTATTTTTTTAGTATTTATATATACATATTTTTTTATTTTGAGATTATTTTGGATAATTTGACTATGAAAATTATACGTGGTTTATATTTATTAAATTTTTTGTGTGAATATTTGAAAAAAAATGAAAATATCACAATATTTAAATTTTAATTTATTTACCAAATTATAAAAAAAAATCAAATAAAAAATATGCAAAAATGAAAAAAACAATTTTAAAAAGTAAAAAAGAAGAAGAAATCATATGTATTAAAAAAAATCATGTATTTAAAACATGACATTTTGTAAAAAGATAAATAAATACAGAGAAGTCGAGCTTTAAAAGTATCAGACCTTGAACTTGTAATTTTAAAACATGATTTTTTTTTATATAATTAAAAGAATCCATTTATAAGATAAGTTTGAATCTGTACTGTTTTTTTTTTATATAAATAAACATTTGATAATGTATGAGTATATACTAATGATTTGATTGATTTTAGAAAGTATATTTTGTATGAAGTTATAGATAAAGTAATAATTAATTTTAGGTAGTTTTATATCATTTGATACTGAATCTATGTAAGAAATATGAATTAGAGGAGTATAGGAAAAGTGCATAAGCATTTTTTGGTTAGGTTACCTAAATGATGAAGAGGCGACAAAAATAACTATAGACAAAGATGGATGGTTGCATACGGGTGATATTGGATACATGGACGATGATGACCAGATTTTCATAGTTGATCGGTTAAAGGAGTTGATCAAATACAAAGGATTTCAAGTTGCTCCTGCTGAGCTGGAAGCCATGCTAATTGTCCACCCGAACATTTCTGAGGCTGTTGTTGTGTCGTGAGTTCTATATTTATCATATAATGTCAATTTTTATCCTATCCTATTTCTTTGCTTTTCTCTTTCAACACAAAGTTTAATGATCACGTTGATTTCAGCATGAAGGATGAAGTTGCAGGAGAGGTTCCAGTTGCATTTGTTGTACTGTCAAATGGTTCTAAGATCTCTGAGGAAGAAATCAAACAATACATCTCAAAGCAGGTATTAATAATTAGCATCGGACACCCCAACTAATATAATTCTTATTTTTTTTTCTTTATTTTTACTATAAACTAAAAAATAAAAATGAGTTAAATATGATTTTTTTATGTAATTTCAAACGAAATTAATTTTCATTATTTGTAGTTCAAACTTTAAATATCTGAGTATAGTAAAAAAAACTATAAAAAATAAGTCTATCAATTTCTTTTACATAATAATATTATTATATTGGAATTTTGATCATAGGGGTTTTTTTTTTTTTGCTTTTTAACAAAATGGGTTATGTTTAAAAAAAATTACAAATTTAGGTTAGTCGTGTCAATTCGGCACGACTTCATATGCACAAATCGTCCCAATTAGACACGACTTTGCCATGTCATAATGAAGTCGTGTCAACTTGACACGACTTTTGCCGTGTCATATATTTTTTTTAATTTTATTGTTTATATATTGGGTAGTAAGTTATGTAATGTTAAAAATTAATTTGATACATAATTTTTTAAGAATGTTAGTTTTAAGGAACAAAAAATTGGATAATCATGTATGGATCATGTTTGGATAATCAGCTTATTCAAACTTTGAGTCATTAACTCCTTAGGACATTGCCCTCGGACATGGTTTCCCACTAGCCAAAAAGTATATTTTGATATTTAATGTCTCATGTATGCAATTTTTTTGATATTTTGATATTTAATGGCGTCGGACATGATCCATACATGATTATCCAATTTTTTGTTCCTTAAAACTAACACTTTTAGAAAATTATGTATAAAATTAATTTTTAACATTACATAACTTACTACCCAATATATAAACAATAAAATTTAAAAAAAAATAAAAAATATGACGTGGCAGAAGTCGTGCCAAGTTGACACGATTTCAATATGACAGGGCAGAAATCATGCCAAGTTGGCACGACTTTAGTATGACATGGCAGAATCGTATTAAATTGGAACGATTTGTGCATATGAAGTCGTGCCAAATTGGTACGACTTGCCTAAATTTGTAATTTTTTTAAACAGACTCCATTTTTTTTTAAAAAAAAATAACCTCATCATAATAAAAACCCTATTATATTCGTGTTAACATGTCTCTTCCTTTATTATAACACAATTTACGTAAAAGAACACTCGACTCAATTTAATAGTATTTTTACTACATATATCCAGCAAGTAAAATGTTTGAATTAGAAAAAAAAAATCACTTTCGTAGTATGATATAAGGATAAAAAGATATTTAACCCATATAAATATTATAATATTATATAGTGTTAAATGATGTAAAAGCTTCTAAATGCAAAAGCAAATTTGTATATTTTGGTGGCAGGTTGTGTTTTACAAAAGAATCAGTAGGGTTTTCTTTGTGGGTTCAATTCCAAAGGCAGCCTCTGGAAAGATCTTGCGAAGAGAGCTGAGGGCAAGGCTTGCAAACGGTCTTCCTTCGTCGTGAATATATGGGGTACAACCTAATTCAGTCATTTTTGCTTAAGGTGCCCATATTTTACAAGCTCAAAATGTTAGTAATAAACTTAAATAAGTTGTTTATTTTATTTTATAAATTTAATAAAATATACACTACAATAAAATTATTAAATAGAAATTAATTTTAAAGAAAGAAATAATTAATTATTATAATAATTAAATTAAAAATTAAAATAATTTTTTA

```
C) Nucleotide sequence comparison of the 4CL locus Phvul.003G147000 between two common bean genotypes

G19833 Phvul.003G147000       --------------------------------------------------
OAC Rex KF303288              AAACAAGGAAAATAAACAATTCCAAGTACATACGTGTGAAGCTGCAGTCA 50

G19833 Phvul.003G147000       ------------------------------------------------TA 2
OAC Rex KF303288              CTGTCTCAACAACTCTTGATGGAACCACGAGCTTCTGAGGCACATATATA 100
                                                                              **
                                                                              ATG  EXON 1
G19833 Phvul.003G147000       TATGCATACATGCTGGCCTCAGTTTGAAGCAAGCTCGCAAATTTTCCTAT 52
OAC Rex KF303288              TATGCATACATGCTGGCCTCAGTTTGAAGCAAGCTCGCAAATTTTCCTAT 150
                              **************************************************

G19833 Phvul.003G147000       GCCACCAACAACACCACCACCACCACCAACAATGGCACCTTCTCCACAAG 102
OAC Rex KF303288              GCCACCAACAACACCACCACCACCACCAACAATGGCACCTTCTCCACAAG 200
                              **************************************************

G19833 Phvul.003G147000       AAATCATCTTCAGATCCCCACTTCCCGATATTCCCATTCCCACGCACCTT 152
OAC Rex KF303288              AAATCATCTTCAGATCCCCACTTCCCGATATTCCCATTCCCACGCACCTT 250
                              **************************************************

G19833 Phvul.003G147000       CCACTACACTCTTACTGCTTCCAAAATCTCTCCCAATTCCAACACCGAAC 202
OAC Rex KF303288              CCACTACACTCTTACTGCTTCCAAAATCTCTCCCAATTCCAACACCGAAC 300
                              **************************************************

G19833 Phvul.003G147000       ATGCCTCATCGACGGCGAGACCGGCGAAACCCTCACCTACGCCGAAGTCG 252
OAC Rex KF303288              ATGCCTCATCGACGGCGAGACCGGCGAAACCCTCACCTACGCCGAAGTCG 350
                              **************************************************

G19833 Phvul.003G147000       ACCTTGCCGCCCACCGCATCGCCGCCGGCCTCCACAACATCGGCATCCGC 302
OAC Rex KF303288              ACCTTGCCGCCCGCCGCATCGCCGCCGGCCTCCACAACATCGGCATCCGC 400
                              ************ *************************************

G19833 Phvul.003G147000       CAGGGGGACGTCATCATGCTCGTCCTCCGCAACTGCCCCCAATTCGCCCT 352
OAC Rex KF303288              CAGGGGGACGTCATCATGCTCGTCCTCCGCAACTGCCCCCAATTCGCCCT 450
                              **************************************************

G19833 Phvul.003G147000       CGCCTTCCTCGGCGCCTCCCACCGCGGCGCCGTCATCACCACTGCCAACC 402
OAC Rex KF303288              CGCCTTCCTCGGCGCCTCCCACCGCGGCGCCGTCATCACCACTGCCAACC 500
                              **************************************************

G19833 Phvul.003G147000       CCTTCTACACGCCGCTGGAGCTTGCCAAGCAAGCAGCCGCCACCAAAACC 452
OAC Rex KF303288              CCTTCTACACGCCGCTGGAGCTTGCCAAGCAAGCAGCCGCCACCAAAACC 550
                              **************************************************

G19833 Phvul.003G147000       AGGCTGGTGATAACGCAATCCGCGTACGTCGATAAAATCAAGAGCTTCGC 502
OAC Rex KF303288              AGGCTGGTGATAACGCAATCCGCGTACGTCGATAAAATCAAGAGCTTCGC 600
                              **************************************************

G19833 Phvul.003G147000       GGACAACAATGACGTGATGGTCATGTGCATCGACTCTGAGGACGACGGCG 552
OAC Rex KF303288              GGACAACAATGACGTGATGGTCATGTGCATCGACTCTGAGGACGACGGCG 650
                              **************************************************

G19833 Phvul.003G147000       TTTTACATTTCTCCACGCTAACGAACGCCGACGAAACGGAAGCCCCCGCC 602
OAC Rex KF303288              TTTTACATTTCTCCACGCTAACGAACGCCGACGAAACGGAAGCCCCCGCC 700
                              **************************************************

G19833 Phvul.003G147000       GTTAAGATTAGCCCAGACGACGTCGTGGCGCTTCCGTTTTCTTCTGGCAC 652
OAC Rex KF303288              GTTAAGATTAGCCCAGACGACGTCGTGGCGCTTCCGTTTTCTTCTGGCAC 750
                              **************************************************

G19833 Phvul.003G147000       TTCGGGGCTCCCCAAGGGCGTTATGTTATCGCATAAAAACTTGGTCACCA 702
OAC Rex KF303288              TTCGGGGCTCCCCAAGGGCGTTATGTTATCGCATAAAAACTTGGTCACCA 800
                              **************************************************
```

```
G19833 Phvul.003G147000       CCATAGCGCAGTTAGTTGACGGCGAAAACGCGCACCAGTACACTCACAGC 752
OAC Rex KF303288              CCATAGCGCAGTTAGTTGACGGCGAAAACGCGCACCAGTACACTCACAGC 850
                              **************************************************

G19833 Phvul.003G147000       GAGGATGTGCTCCTCTGCGTGCTTCCTATGTTTCATATCTATGCGCTCAA 802
OAC Rex KF303288              GAGGATGTGCTCCTCTGCGTGCTTCCTATGTTTCATATCTATGCGCTCAA 900
                              **************************************************

G19833 Phvul.003G147000       TTCAATTTTACTCAGCGGGATTCGGTCCGGTGCGGCCGTTCTCATTGTGC 852
OAC Rex KF303288              TTCAATTTTACTCAGCGGGATTCGGTCCGGTGCGGCCGTTCTCATTGTGC 950
                              **************************************************

G19833 Phvul.003G147000       AGAAGTTTGAGATGACGAGTCTGTTGGAGCTGATCCAGAAGTACAGGGTC 902
OAC Rex KF303288              AGAAGTTTGAGATGACGAGTCTGTTGGAGCTGATCCAGAAGTACAGGGTC 1000
                              **************************************************

G19833 Phvul.003G147000       ACCGTTGCGTCCTTTGTGCCACCCATCGTTTTGGCGTTGGTTAAGAGCGG 952
OAC Rex KF303288              ACCGTTGCGTCCTTTGTGCCACCCATCGTTTTGGCGTTGGTTAAGAGCGG 1050
                              **************************************************

G19833 Phvul.003G147000       AGAGGCCCATCGATACGACCTCTCCTCTGTTCGCGCCGTCGTCACTGGCG 1002
OAC Rex KF303288              AGAGGCCCATCGATACGACCTCTCCTCTGTTCGCGCCGTCGTCACTGGCG 1100
                              **************************************************

G19833 Phvul.003G147000       CCGCACCCTTGGGAAGGGAACTACAAGAAGCTGTCGAATCTAGGCTACCA 1052
OAC Rex KF303288              CCGCACCCTTGGGAAAGGAACTACAAGAAGCTGTCGAATCTAGGCTACCA 1150
                              *************** **********************************
                                                                                INTRON 1
G19833 Phvul.003G147000       CACGCCACTTTTGGACAGgtttcattttcattacagaaaaaataataatc 1102
OAC Rex KF303288              CACGCCACTTTTGGACAGgtttcattttcattacagaaaaaataataatc 1200
                              **************************************************

G19833 Phvul.003G147000       attacccaaattcaaattaaaacgttttcctttataaaatcgataacaag 1152
OAC Rex KF303288              atcacccaaattcaaattaaaacgttttcctttataaaatcgataacaag 1250
                              ** ***********************************************

G19833 Phvul.003G147000       tgagattttagtgacgtaagctactgaaatttctattcattaaattccag 1202
OAC Rex KF303288              tgagattttagtgacgtaagctactgaaatttctattcattaaattccag 1300
                              **************************************************

G19833 Phvul.003G147000       atttctttgtcgcattaatactcatttttgtggatcacaggtacgagcgt 1252
OAC Rex KF303288              atttctttgtcgcattaatactcatttttgtggatcacaggtacgagcgt 1350
                              **************************************************

G19833 Phvul.003G147000       gggaatctcataaataaatcttacaatcacccttgcacgcggaaaaatat 1302
OAC Rex KF303288              gggaatctcataaataaatcttataatcacccttgcacgcggaaaaatat 1400
                              *********************** **************************

G19833 Phvul.003G147000       tctttttaaaaaccctcattattcatcgtacaagcttatcaaaaattatt 1352
OAC Rex KF303288              tctttttaaaaaccctcattattcatcgtacaagcttatcaaaaattatt 1450
                              **************************************************

G19833 Phvul.003G147000       ttttt-atttactttactctatttgtaaacaatatttattttaaaggata 1401
OAC Rex KF303288              ttttttatttactttactctatttgtaaacaatatttattttaaaggata 1500
                              ***** ********************************************

G19833 Phvul.003G147000       gttttgaagcatggccttcttttaaaatactgttttttttcaaaatctta 1451
OAC Rex KF303288              gttttgaagcatggccttcttttaaaatactgttttttttcaaaatctta 1550
                              **************************************************
                                                                                EXON 2
G19833 Phvul.003G147000       aaaaatagtcaaacaaaatttgttttggacttgcagGGATATGGGATGAC 1501
OAC Rex KF303288              aaaaatagtcaaacaaaatttgttttggacttgcagGGATATGGGATGAC 1600
                              **************************************************
```

```
G19833 Phvul.003G147000       AGAAGCAGGACCACTTGCGATTAGCATGGCATTTGCAAAAGAACCCTCAA 1551
OAC Rex KF303288              AGAAGCAGGACCACTTGCGATTAGCATGGCATTTGCAAAAGAACCCTCAA 1650
                              **************************************************

G19833 Phvul.003G147000       ATATAAAACCTGGTGCATGCGGCACCGTTGTGAGAAACGCTGAGATGAAA 1601
OAC Rex KF303288              ATATAAAACCTGGTGCATGCGGCACCGTTGTGAGAAACGCTGAGATGAAA 1700
                              **************************************************

G19833 Phvul.003G147000       ATCGTGGACACAGAAACGGGTGTTTCCCTTCCGAAAAACGAAACCGGTGA 1651
OAC Rex KF303288              ATCGTGGACACAGAAACGGGTGTTTCCCTTCCGAAAAACGAAACCGGTGA 1750
                              **************************************************
                                                                                INTRON 2
G19833 Phvul.003G147000       AATTTGCATAAGAGGCACAAAGGTTATGAAAGgtattatagcatatactt 1701
OAC Rex KF303288              AATTTGCATAAGAGGCACAAAGGTTATGAAAGgtattatagcatatactt 1800
                              **************************************************

G19833 Phvul.003G147000       gtattttacttataaacagagtgatgattcatgccactatcatataaaca 1751
OAC Rex KF303288              gtattttacttataaacagagtgatgattcatgccactatcatataaaca 1850
                              **************************************************

G19833 Phvul.003G147000       gagtgtgaataaaaaaaattataaaacagagtgaaacattagtcataacg 1801
OAC Rex KF303288              gagtgtgaataaaaaaaattataaaacagagtgaaacattagtcataacg 1900
                              **************************************************

G19833 Phvul.003G147000       taaactaagaaacaccaacttcgttgacgctcttcttcgacatcgattag 1851
OAC Rex KF303288              taaactaagaaacaccaacttcgttgacgctcttcttcgacatcgattag 1950
                              **************************************************

G19833 Phvul.003G147000       gttcaaacaaaaactaatactatttttctcattcgatgaacaactttcct 1901
OAC Rex KF303288              gttcaaacaaaaactaatactatttttctcattcgatgaacaactttcct 2000
                              **************************************************

G19833 Phvul.003G147000       tgagaaatattcccaacaatagcaggttggctgagacttaatccttggta 1951
OAC Rex KF303288              tgagaaatattcccaacaatagcaggttggctgagacttaatccttggta 2050
                              **************************************************

G19833 Phvul.003G147000       attaaccatcttcatcaattgtgcctttcatattcggttgtctctcccca 2001
OAC Rex KF303288              attaaccatcttcatcaattgtgcctttcatattcggttgtcttctcc-- 2098
                              *******************************************   **

G19833 Phvul.003G147000       ttagatgtttcctaagtatattacatatgcatttccttttttgaaaatta 2051
OAC Rex KF303288              -------tttc-------------------atttccttttttgaaaatta 2122
                                     ****                   ********************

G19833 Phvul.003G147000       aaatgacatatttagtatttgtatttatagttcctaaaattaagtgaatt 2101
OAC Rex KF303288              aaatgacatatttagtatttgtatttatagttcctaaaattaagtgaatt 2172
                              **************************************************

G19833 Phvul.003G147000       tttatattttaaaaattaaaatgttttgctatatttatatatatat---- 2147
OAC Rex KF303288              tttatattttaaaaattaaaatgttttgctatatttatatatatatatat 2222
                              **********************************************

G19833 Phvul.003G147000       ------------tttttttaaaaggagtgtaagtggggtggttgtgagtt 2185
OAC Rex KF303288              atatttttattttttttttaaaaggagtgtaagtggggtggttgtgagtt 2272
                                          **************************************

G19833 Phvul.003G147000       gggtggaagaaaagattggttggtatgaacagaatatacaa--------- 2226
OAC Rex KF303288              gggtggaagaaaagatgggttggtatgaacagaatatacaacagaatata 2322
                              **************** ************************

G19833 Phvul.003G147000       ---ctatgatacttatatatat--ggcctccacaaattcaaagtgggaac 2271
OAC Rex KF303288              cagctatgatacttatatatatatggcctccacaaattcaaagtgggaac 2372
                                 *******************  **************************
```

```
G19833 Phvul.003G147000       tccagaagaattcaacggctcacgtaaaagtagctttatgaggcaccaaa 2321
OAC Rex KF303288              tccagaagaattcaacggctcacgtaaaagtagctttatgaggcaccaaa 2422
                              **************************************************

G19833 Phvul.003G147000       ccatgacaatgattttagagaacttaattaataatttattatctggaatt 2371
OAC Rex KF303288              ccatgacaatgattttagagaacttaattaaaaatttattatctggaatt 2472
                              ******************************* ******************

G19833 Phvul.003G147000       ggtaacgctgacagtgacatcataatgctgcttcttccactccttaaagc 2421
OAC Rex KF303288              ggtaacgctgacagtgacatcataatgctgcttcttccactccttaaagc 2522
                              **************************************************

G19833 Phvul.003G147000       agactccgatcacataccttcacgtgcccaataactcttgcatgctaatt 2471
OAC Rex KF303288              agactccgatcacataccttcacgtgcccattaactcttgcatgctaatt 2572
                              ****************************** *******************

G19833 Phvul.003G147000       ttttcaacacttttattttttgttcaaacattttttctcctgttgtttat 2521
OAC Rex KF303288              ttttcaacacttttattttttgttcaaacattttttctcctgttgtttat 2622
                              **************************************************

G19833 Phvul.003G147000       tcttttatagctttcacatattttaggtagaactaaaattatttcctgtt 2571
OAC Rex KF303288              tcttttatagcttccacatattttaggtagaactaaaattatttcctgtt 2672
                              ************* ************************************

G19833 Phvul.003G147000       tttaatttggttttaagctagtaaattttgtgcgtttaagaagttcccat 2621
OAC Rex KF303288              tttaatttggttttaagctagtaaattttgtgcgtttaagaagttcccat 2722
                              **************************************************

G19833 Phvul.003G147000       aatttaattattttttctatttgaagaagctttaataactcgtattattt 2671
OAC Rex KF303288              aatttaattattttttctatttgaagaagctttaataactcgtattattt 2772
                              **************************************************

G19833 Phvul.003G147000       tttgtcaaaaatatgttttaaaatatttaaataaatcatctttataaaga 2721
OAC Rex KF303288              tt-gtcaaaaatatgttttaaaatatttaaataaatcatctttataaaga 2821
                              ** ***********************************************

G19833 Phvul.003G147000       atacccgtaaagaataaataaataaaatatcatgtcaactcaatttaacc 2771
OAC Rex KF303288              atacccgtaaaaaataaataaataaaatatcatgtcaactcaatttaac- 2870
                              *********** *************************************

G19833 Phvul.003G147000       ttttatgtatggttaaaactacataagtatttattaaaaattatttagat 2821
OAC Rex KF303288              ttttatgtatggttaaaactacataagtatttattaaaaattatttagat 2920
                              **************************************************

G19833 Phvul.003G147000       attgtatggttttaatttcctatacgtgctcagaaaagctcttttaaagt 2871
OAC Rex KF303288              attgtatggttt-aatttcctatacgtgctcagaaaagctcttttaaagt 2969
                              ************ *************************************

G19833 Phvul.003G147000       atatacgatatatttttgcttaattaagtaagtttttcttgttgctaaac 2921
OAC Rex KF303288              atatacgatatatttttgcttaattaagtaagtttttcttgttgctaaac 3019
                              **************************************************

G19833 Phvul.003G147000       tcttggttaaataagttatttattttaatattgtagtcaatattatataa 2971
OAC Rex KF303288              tcttggttaaataagttatttattttaatattgtagtcaatattatataa 3069
                              **************************************************

G19833 Phvul.003G147000       acttaagaattctaaagagaatatttctcttagtttttattctagattat 3021
OAC Rex KF303288              acttaagaattctaaagagaatatttctcttagtttttattctagattat 3119
                              **************************************************

G19833 Phvul.003G147000       taaattactttgtcccataaaaatacttaaacagtgttatttatatttaa 3071
OAC Rex KF303288              taaattactttgtgccataaaaatacttaaacagtgttatttatatttaa 3169
                              ************* ************************************
```

```
G19833 Phvul.003G147000       aaaagcatgtaaaaaactctatcaatatttttgaaggtattgatataaaa 3121
OAC Rex KF303288              aaaagcatgtaaaaaactctatcaatatttttgaaggtattgatataaaa 3219
                              **************************************************

G19833 Phvul.003G147000       gaatttcagcaaacaagttgttgatatataggtgagaaaaattatatgct 3171
OAC Rex KF303288              gaatttcagcacacaagttgttgatatataggtgagaaaaattatatgct 3269
                              *********** **************************************

G19833 Phvul.003G147000       taccttgaaatggaaagtgatggcagcgataagactgggagtccacatcc 3221
OAC Rex KF303288              taccttgaaatggaaagtgatggcagcgataagactggaagtccacatcc 3319
                              ************************************** ***********

G19833 Phvul.003G147000       tcacctaccccatttacctgattttcccagagagacgaaattacaaggtc 3271
OAC Rex KF303288              tcacctaccccatttacttgattttcccagagagacgaaattacaaggtc 3369
                              ***************** ********************************

G19833 Phvul.003G147000       aaaatcacagttacttttcaacaacaacaaaaactagtcacaattattca 3321
OAC Rex KF303288              aaaatcacagttacttttcaacaacaacaaaaactagtcacaattattca 3419
                              **************************************************

G19833 Phvul.003G147000       atcatgcaagacgtgtaatccacttattattctccacaccttgcaaaata 3371
OAC Rex KF303288              atcatgcaagacgtgtaatccacttattattctccacaccttgcaaaata 3469
                              **************************************************

G19833 Phvul.003G147000       gagagttacgtcaactttcgcaaaaaaattgaaatactatgaagtaggtg 3421
OAC Rex KF303288              gagagttacgtcaactttcgcagaaaaattgaaatactatgaagtaggtg 3519
                              ********************** ***************************

G19833 Phvul.003G147000       atcgataagcacttgtctattattggactgagtttaaaactctgtataat 3471
OAC Rex KF303288              atcgataagcacttgtctattattggactgagtttaaaactctgtataat 3569
                              **************************************************

G19833 Phvul.003G147000       tgataaaattaaaaattaaaaattaaaaaaaaa--ctctataaacctaat 3519
OAC Rex KF303288              tgataaaattaaaaattaaaaattaaaaaaaaaaactctataaacctaat 3619
                              *********************************  ***************

G19833 Phvul.003G147000       taaacttaactaggctaagccttgagaatgtaatattttacaaaaataac 3569
OAC Rex KF303288              taaatttaactaggctaagccttgagaatgtaatattttacaaaaataac 3669
                              **** *********************************************

G19833 Phvul.003G147000       attaattttttggaaaataatcctttcacatgtacattgaaagatatcga 3619
OAC Rex KF303288              attaattttttggaaaataatcctttcacat--------------atcga 3705
                              *******************************              *****

OAC Rex KF303288              tttataactgtaattttctacttcattaaaataaaataaaaaattatttg 3755
G19833 Phvul.003G147000       tttataactataattttctacttcatttcaataaaataaaaaattatttg 3669
                              ********* *****************  *********************

G19833 Phvul.003G147000       tcttacaattataatttaatacagattctaaaaacatattttttctt--- 3716
OAC Rex KF303288              tcttacaattataatttaatacagattctaaaaacatattttttcttaac 3805
                              ***********************************************
                                                                                EXON 3
G19833 Phvul.003G147000       --gttaatttgtggttggtcagGATATCTAAACGACCCAGAAGCTACAGA 3764
OAC Rex KF303288              atgttaatttgtggttggtcagGATATCTAAACGACCCAGAGGCTACAGA 3855
                                *************************************** ********

G19833 Phvul.003G147000       GAGAACTATAGACAAAGATGGATGGCTCCACACAGGAGATATTGGTTACA 3814
OAC Rex KF303288              GAGAACCATAGACAAAGATGGATGGCTCCACACAGGAGATATTGGTTACA 3905
                              ****** *******************************************

G19833 Phvul.003G147000       TCGACGATGATGACGAACTCTTCATTGTTGATAGGTTAAAGGAACTGATC 3864
OAC Rex KF303288              TCGACGATGATGACGAACTCTTCATTGTTGATAGGTTAAAGGAACTGATC 3955
                              **************************************************
```

```
G19833 Phvul.003G147000       AAATACAAAGGGTTCCAAGTAGCTCCTGCTGAGCTCGAAGCATTGTTAAT 3914
OAC Rex KF303288              AAATACAAAGGGTTCCAAGTAGCTCCTGCTGAGCTCGAAGCATTGTTAAT 4005
                              **************************************************
                                                                                INTRON 3
G19833 Phvul.003G147000       TGCCCATCCAAACATCTCTGAAGCTGCCGTTGTACCgtaagttcaacttt 3964
OAC Rex KF303288              TGCCCATCCAAACATCTCTGAAGCTGCCGTTGTACCgtaagttcaacttt 4055
                              **************************************************

G19833 Phvul.003G147000       caaaccctaaaaaacctcctaggatgtacatgtctgagttttatgttttt 4014
OAC Rex KF303288              caaaccctaaaaaacctcctaggatgtagatgtctgagtcttatgttttt 4105
                              **************************** ********** **********

G19833 Phvul.003G147000       ttgggtaaacttttatgtctgctttattgtctcgttatagttattttcag 4064
OAC Rex KF303288              ttaggtaaacttatatgtctgctttgttgtctcgttatagttattttcag 4155
                              ** ********* ************ ************************

G19833 Phvul.003G147000       aaaatatatacagttataattaatggtagtatgtaatgatatcaatgggt 4114
OAC Rex KF303288              aaaatatatacagttataattaatggtaatatgtaatgatatcaatgggt 4205
                              **************************** *********************
                                                                                EXON 4
G19833 Phvul.003G147000       gttggtttcagCATGAAAGATGAAGCTGCCGGAGAAATTCCTGTTGCATT 4164
OAC Rex KF303288              gttggtttcagCATGAAAGATGAAGCTGCCGGAGAAATTCCTGTTGCATT 4255
                              **************************************************

G19833 Phvul.003G147000       TGTTGTAAGATCAAAAGGTTCTGAGATCACTGAAGATGAAATAAAGCAAT 4214
OAC Rex KF303288              TGTTGTAAGATCAAAAGGTTCTGAGATCACTGAAGATGAAATAAAGCAAT 4305
                              **************************************************
                                                                                INTRON 4
G19833 Phvul.003G147000       ACATTTCACAACAGgttctgttatcgaacatgtgaattaagtatatgtga 4264
OAC Rex KF303288              ACATTTCACAACAGgttcagttatcgaacatttgaatgaagtatatgtga 4355
                              ****************** ************ ***** ************

G19833 Phvul.003G147000       cttatatttttgtggttgcaattgaatttattgggatgtgtttgtggcag 4314
OAC Rex KF303288              gttatatttttgtggttgtaattgaatttattgggatgtgtttgtggcag 4405
                               ***************** *******************************
                                                                                EXON 5
G19833 Phvul.003G147000       GTGGTATTTTACAAGAGAATCGGTAGGGTTTTCTTCACAGACTCTATTCC 4364
OAC Rex KF303288              GTGGTATTTTACAAGAGAATCGGTAGGGTTTTCTTCACAGACTCTATTCC 4455
                              **************************************************

G19833 Phvul.003G147000       TAAAGCAGCCTCAGGCAAAATTCTGAGGAAGGTACTAACTGCGAGACTTA 4414
OAC Rex KF303288              TAAAGCAGCCTCAGGCAAAATTCTGAGGAAGGTACTAACTGCAAGACTTA 4505
                              ****************************************** *******
                                                     TAG
G19833 Phvul.003G147000       ACGAAGGTCTGGTGGTGGCCAATTAGGTCCTTGTTTGGGACACACCAAAA 4464
OAC Rex KF303288              ACGAAGGTCTGGTGGTGGCCAATTAGGTCCTTGTTTGGGACACACCAAAA 4555
                              **************************************************

G19833 Phvul.003G147000       CCGTCCCTCTTTTACTTACCGCACCACAACTCTTTCTTTTGTGGTTTTTG 4514
OAC Rex KF303288              CCGTCCCTCTTTTACTTACCGCACCACAACTCTTTCTTTTGTGGTTTTTG 4605
                              **************************************************

G19833 Phvul.003G147000       TTCCAATTTTTACATCTCGTTTTCAACATGTGTTCAATACAAAAATTGTT 4564
OAC Rex KF303288              TTCCAATTTTTACATCTCGTTTTCAACATGTGTTCAATACAAAAATTGTT 4655
                              **************************************************

G19833 Phvul.003G147000       GTGTTGTATAAGTTTATTCATGTGTAACGTATGATGTTCTATGCATGTTG 4614
OAC Rex KF303288              GTGTTGTATAAGTTTATTCATGTGTAACGTATGATGTTCTATGCATGTTG 4705
                              **************************************************

G19833 Phvul.003G147000       TATATATACTGGAGACCAGACTCTGAATTACAACATGATCAAATTAAAAT 4664
OAC Rex KF303288              TATATATACTGGAGACCAGACTCTGAATTACAACATGATCAAATTAAAAT 4755
                              **************************************************
```

```
G19833 Phvul.003G147000       GCAATTTGCAAACAAAATTTTGCATAATTTAATTTGAACTTGGAATAGTG 4714
OAC Rex KF303288              GCAATTTGCAAACAAAATTTTGCATAATTTAATTTGAACTTGGAATAGTG 4805
                              **************************************************

G19833 Phvul.003G147000       GAATGTATGAAG-------------------------------------- 4726
OAC Rex KF303288              GAATGTATGAAGACCTAATATATATAGATGAGTTCATAAAGTTATCAAAA 4855
                              ************

G19833 Phvul.003G147000       -------------------------
OAC Rex KF303288              TAATAATAAAAGAAAATACAAAAAC 4880


OAC Rex sequence:
>KF303288 OAC Rex gene for 4-coumarate:CoA ligase (4CL-3; 4,880 bp); similar to P. vulgaris acyl-CoA synthetase locus Phvul.003G147000 (cultivar G19833)
```

AAACAAGGAAAATAAACAATTCCAAGTACATACGTGTGAAGCTGCAGTCACTGTCTCAACAACTCTTGATGGAACCACGAGCTTCTGAGGCACATATATATATGCATACATGCTGGCCTCAGTTTGAAGCAAGCTCGCAAATTTTCCTATGCCACCAACAACACCACCACCACCACCAACAATGGCACCTTCTCCACAAGAAATCATCTTCAGATCCCCACTTCCCGATATTCCCATTCCCACGCACCTTCCACTACACTCTTACTGCTTCCAAAATCTCTCCCAATTCCAACACCGAACATGCCTCATCGACGGCGAGACCGGCGAAACCCTCACCTACGCCGAAGTCGACCTTGCCGCCCGCCGCATCGCCGCCGGCCTCCACAACATCGGCATCCGCCAGGGGGACGTCATCATGCTCGTCCTCCGCAACTGCCCCCAATTCGCCCTCGCCTTCCTCGGCGCCTCCCACCGCGGCGCCGTCATCACCACTGCCAACCCCTTCTACACGCCGCTGGAGCTTGCCAAGCAAGCAGCCGCCACCAAAACCAGGCTGGTGATAACGCAATCCGCGTACGTCGATAAAATCAAGAGCTTCGCGGACAACAATGACGTGATGGTCATGTGCATCGACTCTGAGGACGACGGCGTTTTACATTTCTCCACGCTAACGAACGCCGACGAAACGGAAGCCCCCGCCGTTAAGATTAGCCCAGACGACGTCGTGGCGCTTCCGTTTTCTTCTGGCACTTCGGGGCTCCCCAAGGGCGTTATGTTATCGCATAAAAACTTGGTCACCACCATAGCGCAGTTAGTTGACGGCGAAAACGCGCACCAGTACACTCACAGCGAGGATGTGCTCCTCTGCGTGCTTCCTATGTTTCATATCTATGCGCTCAATTCAATTTTACTCAGCGGGATTCGGTCCGGTGCGGCCGTTCTCATTGTGCAGAAGTTTGAGATGACGAGTCTGTTGGAGCTGATCCAGAAGTACAGGGTCACCGTTGCGTCCTTTGTGCCACCCATCGTTTTGGCGTTGGTTAAGAGCGGAGAGGCCCATCGATACGACCTCTCCTCTGTTCGCGCCGTCGTCACTGGCGCCGCACCCTTGGGAAAGGAACTACAAGAAGCTGTCGAATCTAGGCTACCACACGCCACTTTTGGACAGGTTTCATTTTCATTACAGAAAAAATAATAATCATCACCCAAATTCAAATTAAAACGTTTTCCTTTATAAAATCGATAACAAGTGAGATTTTAGTGACGTAAGCTACTGAAATTTCTATTCATTAAATTCCAGATTTCTTTGTCGCATTAATACTCATTTTTGTGGATCACAGGTACGAGCGTGGGAATCTCATAAATAAATCTTATAATCACCCTTGCACGCGGAAAAATATTCTTTTTAAAAACCCTCATTATTCATCGTACAAGCTTATCAAAAATTATTTTTTTTATTTACTTTACTCTATTTGTAAACAATATTTATTTTAAAGGATAGTTTTGAAGCATGGCCTTCTTTTAAAATACTGTTTTTTTTCAAAATCTTAAAAAATAGTCAAACAAAATTTGTTTTGGACTTGCAGGGATATGGGATGACAGAAGCAGGACCACTTGCGATTAGCATGGCATTTGCAAAAGAACCCTCAAATATAAAACCTGGTGCATGCGGCACCGTTGTGAGAAACGCTGAGATGAAAATCGTGGACACAGAAACGGGTGTTTCCCTTCCGAAAAACGAAACCGGTGAAATTTGCATAAGAGGCACAAAGGTTATGAAAGGTATTATAGCATATACTTGTATTTTACTTATAAACAGAGTGATGATTCATGCCACTATCATATAAACAGAGTGTGAATAAAAAAAATTATAAAACAGAGTGAAACATTAGTCATAACGTAAACTAAGAAACACCAACTTCGTTGACGCTCTTCTTCGACATCGATTAGGTTCAAACAAAAACTAATACTATTTTTCTCATTCGATGAACAACTTTCCTTGAGAAATATTCCCAACAATAGCAGGTTGGCTGAGACTTAATCCTTGGTAATTAACCATCTTCATCAATTGTGCCTTTCATATTCGGTTGTCTTCTCCTTTCATTTCCTTTTTTGAAAATTAAAATGACATATTTAGTATTTGTATTTATAGTTCCTAAAATTAAGTGAATTTTTATATTTTAAAAATTAAAATGTTTTGCTATATTTATATATATATATATATATTTTTATTTTTTTTTTAAAAGGAGTGTAAGTGGGGTGGTTGTGAGTTGGGTGGAAGAAAAGATGGGTTGGTATGAACAGAATATACAACAGAATATACAGCTATGATACTTATATATATATGGCCTCCACAAATTCAAAGTGGGAACTCCAGAAGAATTCAACGGCTCACGTAAAAGTAGCTTTATGAGGCACCAAACCATGACAATGATTTTAGAGAACTTAATTAAAAATTTATTATCTGGAATTGGTAACGCTGACAGTGACATCATAATGCTGCTTCTTCCACTCCTTAAAGCAGACTCCGATCACATACCTTCACGTGCCCATTAACTCTTGCATGCTAATTTTTTCAACACTTTTATTTTTTGTTCAAACATTTTTTCTCCTGTTGTTTATTCTTTTATAGCTTCCACATATTTTAGGTAGAACTAAAATTATTTCCTGTTTTTAATTTGGTTTTAAGCTAGTAAATTTTGTGCGTTTAAGAAGTTCCCATAATTTAATTATTTTTTCTATTTGAAGAAGCTTTAATAACTCGTATTATTTTTGTCAAAAATATGTTTTAAAATATTTAAATAAATCATCTTTATAAAGAATACCCGTAAAAAATAAATAAATAAAATATCATGTCAACTCAATTTAACTTTTATGTATGGTTAAAACTACATAAGTATTTATTAAAAATTATTTAGATATTGTATGGTTTAATTTCCTATACGTGCTCAGAAAAGCTCTTTTAAAGTATATACGATATATTTTTGCTTAATTAAGTAAGTTTTTCTTGTTGCTAAACTCTTGGTTAAATAAGTTATTTATTTTAATATTGTAGTCAATATTATATAAACTTAAGAATTCTAAAGAGAATATTTCTCTTAGTTTTTATTCTAGATTATTAAATTACTTTGTGCCATAAAAATACTTAAACAGTGTTATTTATATTTAAAAAAGCATGTAAAAAACTCTATCAATATTTTTGAAGGTATTGATATAAAAGAATTTCAGCACACAAGTTGTTGATATATAGGTGAGAAAAATTATATGCTTACCTTGAAATGGAAAGTGATGGCAGCGATAAGACTGGAAGTCCACATCCTCACCTACCCCATTTACTTGATTTTCCCAGAGAGACGAAATTACAAGGTCAAAATCACAGTTACTTTTCAACAACAACAAAAACTAGTCACAATTATTCAATCATGCAAGACGTGTAATCCACTTATTATTCTCCACACCTTGCAAAATAGAGAGTTACGTCAACTTTCGCAGAAAAATTGAAATACTATGAAGTAGGTGATCGATAAGCACTTGTCTATTATTGGACTGAGTTTAAAACTCTGTATAATTGATAAAATTAAAAATTAAAAATTAAAAAAAAAAACTCTATAAACCTAATTAAATTTAACTAGGCTAAGCCTTGAGAATGTAATATTTTACAAAAATAACATTAATTTTTTGGAAAATAATCCTTTCACATATCGATTTATAACTGTAATTTTCTACTTCATTAAAATAAAATAAAAAATTATTTGTCTTACAATTATAATTTAATACAGATTCTAAAAACATATTTTTTCTTAACATGTTAATTTGTGGTTGGTCAGGATATCTAAACGACCCAGAGGCTACAGAGAGAACCATAGACAAAGATGGATGGCTCCACACAGGAGATATTGGTTACATCGACGATGATGACGAACTCTTCATTGTTGATAGGTTAAAGGAACTGATCAAATACAAAGGGTTCCAAGTAGCTCCTGCTGAGCTCGAAGCATTGTTAATTGCCCATCCAAACATCTCTGAAGCTGCCGTTGTACCGTAAGTTCAACTTTCAAACCCTAAAAAACCTCCTAGGATGTAGATGTCTGAGTCTTATGTTTTTTTAGGTAAACTTATATGTCTGCTTTGTTGTCTCGTTATAGTTATTTTCAGAAAATATATACAGTTATAATTAATGGTAATATGTAATGATATCAATGGGTGTTGGTTTCAGCATGAAAGATGAAGCTGCCGGAGAAATTCCTGTTGCATTTGTTGTAAGATCAAAAGGTTCTGAGATCACTGAAGATGAAATAAAGCAATACATTTCACAACAGGTTCAGTTATCGAACATTTGAATGAAGTATATGTGAGTTATATTTTTGTGGTTGTAATTGAATTTATTGGGATGTGTTTGTGGCAGGTGGTATTTTACAAGAGAATCGGTAGGGTTTTCTTCACAGACTCTATTCCTAAAGCAGCCTCAGGCAAAATTCTGAGGAAGGTACTAACTGCAAGACTTAACGAAGGTCTGGTGGTGGCCAATTAGGTCCTTGTTTGGGACACACCAAAACCGTCCCTCTTTTACTTACCGCACCACAACTCTTTCTTTTGTGGTTTTTGTTCCAATTTTTACATCTCGTTTTCAACATGTGTTCAATACAAAAATTGTTGTGTTGTATAAGTTTATTCATGTGTAACGTATGATGTTCTATGCATGTTGTATATATACTGGAGACCAGACTCTGAATTACAACATGATCAAATTAAAATGCAATTTGCAAACAAAATTTTGCATAATTTAATTTGAACTTGGAATAGTGGAATGTATGAAGACCTAATATATATAGATGAGTTCATAAAGTTATCAAAATAATAATAAAAGAAAATACAAAAAC

```
D) Nucleotide sequence comparison of the 4CL locus Phvul.005G131400 between two common bean genotypes


G19833 Phvul.005G131400        --------------------------------------------------
OAC Rex KF303289               TAATTTTTGTATAAAATATTTTGGAGAGAATATTCTTGGTCCATGAAAAC 50


G19833 Phvul.005G131400        -----------------------TATGATAATGTTATAGAATCAGAGGTT 27
OAC Rex KF303289               TTTAAGAATAAAGAAGAGTTGATTATGATAATGTTATAGAATCAGAGGTT 100
                                                      ***************************
                                                        ATG                      EXON 1
G19833 Phvul.005G131400        ATTGTGGATTCGAAATCCAATTAGAATGCAGAGATCCGGGTATGGCTCCG 77
OAC Rex KF303289               ATTGTGGATTCGAAATCCAATTAGAATGCAGAGATCCGGGTATGGCTCCG 150
                               **************************************************

G19833 Phvul.005G131400        ACGGAATATTCCGGTCACTGAGACCTTCAATCGCTCTCCCCAAACAACCC 127
OAC Rex KF303289               ACGGAATATTCCGGTCACTGAGACCTTCAATCGTTCTCCCCAAACAACCC 200
                               ********************************* ****************

G19833 Phvul.005G131400        AACCTCACACTCGTTTCCCATCTATTCAACAGAGTCGCACCATTTCCCTC 177
OAC Rex KF303289               AACCTCACACTCGTTTCCCATCTATTCAACAGAGTCGCACCATTTCCCTC 250
                               **************************************************

G19833 Phvul.005G131400        CAAACACGCCCTTATCGATGCCGATTCTTCCCAAACCCTTTCATTCGCGC 227
OAC Rex KF303289               CAAACACGCCCTTATCGATGCCGATTCTTCCCAAACCCTTTCATTCGCGC 300
                               **************************************************

G19833 Phvul.005G131400        AGTTGAAGTCGCTAACCGTTAAAGTCTCACACGGCCTCCTCCGTCTCGGC 277
OAC Rex KF303289               AGTTGAAGTCGCTAACCGTTAAAGTCTCACACGGCCTCCTCCGTCTCGGC 350
                               **************************************************

G19833 Phvul.005G131400        GTCGCCAAAAACGACGTCGTCCTCTTCCTTGCCCCCAACGATATCCGCTA 327
OAC Rex KF303289               GTCGCCAAAAACGACGTCGTCCTCTTCCTTGCCCCCAACGATATCCGCTA 400
                               **************************************************

G19833 Phvul.005G131400        CATCGTCTGCTTCCTCGCTGTCACCTCCCTCGGCGCCATCGTCACTACCG 377
OAC Rex KF303289               CATCGTCTGCTTCCTCGCTGTCACCTCCCTCGGCGCCATCGTCACTACCG 450
                               **************************************************

G19833 Phvul.005G131400        TCAATCCCGCCTACACCGCCGCCGAGGTCGCCAAACAGGCCAAAGACTCC 427
OAC Rex KF303289               TCAATCCCGCCTACACCGCCGCCGAGGTCGCCAAACAGGCCAAAGACTCC 500
                               **************************************************

G19833 Phvul.005G131400        AATCCCAAATTTGTCGTCACTGTCCCCGAACTCTGGGACAAGCTCCAACA 477
OAC Rex KF303289               AATCCCAAATTTGTCGTCACTGTCCCCGAACTCTGGGACAAGCTCCAACA 550
                               **************************************************

G19833 Phvul.005G131400        CCTCAACCTCCCCGCTGTGTTCCTTCATTCCCGCGCGCACTCCAACGCGA 527
OAC Rex KF303289               CCTCAACCTCCCCGCTGTGTTCCTTCATTCCCGCGCGCACTCCAACGCGA 600
                               **************************************************

G19833 Phvul.005G131400        CTAGCTTCGACGCGCTCGTCCATCTCGCAGGGTCGCCTACCGAGTTTCCG 577
OAC Rex KF303289               CTAGCTTCGACGCGCTCGTCCATCTCGCAGGGTCGCCTACCGAGTTTCCG 650
                               **************************************************

G19833 Phvul.005G131400        AAAGTTGACGTCAAGCAGAGTGACACGGCGGTGCTGCTGTACTCCTCCGG 627
OAC Rex KF303289               AAAGTTGACGTCAAGCAGAGTGACACGGCGGTGCTGCTGTACTCCTCCGG 700
                               **************************************************

G19833 Phvul.005G131400        GACCACTGGCCTGAGCAAGGGCGTGGTTCTAACGCACGGGAACTTCGTGG 677
OAC Rex KF303289               GACCACTGGCCTGAGCAAGGGCGTGGTTCTAACGCACGGGAACTTCGTGG 750
                               **************************************************
```

```
G19833 Phvul.005G131400        CGGCATCGTTGATGATAGGATTGGACGATGATTTGGCCGGGGAGATGCAT 727
OAC Rex KF303289               CGGCATCGTTGATGATAGGATTGGACGATGATTTGGCCGGGGAGATGCAT 800
                               **************************************************

G19833 Phvul.005G131400        AGCGTGTTCCTCTGCGTTCTGCCGATGTTTCATGTGTTTGGTTTGATGGT 777
OAC Rex KF303289               AGCGTGTTCCTCTGCGTTCTGCCGATGTTTCATGTGTTTGGTTTGATGGT 850
                               **************************************************

G19833 Phvul.005G131400        GATTTCGTACTCGCAGCTTCAGAGGGGCAGTGCTGTGGTGTCTCTGAAGA 827
OAC Rex KF303289               GATTTCGTACTCGCAGCTTCAGAGGGGCAGTGCTGTGGTGTCTCTGAAGA 900
                               **************************************************

G19833 Phvul.005G131400        AGTTTGAGTTTGAACTGGTTTTGAAGACGATTGAGAAGTTTAGTGTGACG 877
OAC Rex KF303289               AGTTTGAGTTTGAACTGGTTTTGAAGACGATTGAGAAGTTTAGTGTGACG 950
                               **************************************************

G19833 Phvul.005G131400        CATTTGTGGGTTGTGCCTCCGATTATTCTTGCTTTGGCCAAACATGGTTT 927
OAC Rex KF303289               CATTTGTGGGTTGTGCCTCCGATTATTCTTGCTTTGGCCAAACATGGTTT 1000
                               **************************************************

G19833 Phvul.005G131400        GGTTGATAAGTATGATCTTTCTTCGCTGAAGCATATTGGTTCTGGCGCTG 977
OAC Rex KF303289               GGTTGATAAGTATGATCTTTCTTCGCTGAAGCATATTGGTTCTGGCGCTG 1050
                               **************************************************

G19833 Phvul.005G131400        CTCCTCTTGGGAAAGAGTTGATGGAGGAGTGTGCTAAACGTTTCCCTCGT 1027
OAC Rex KF303289               CTCCTCTTGGGAAAGAGTTGATGGAGGAGTGTGCTAAACGTTTCCCTCGT 1100
                               **************************************************
                                                                                 INTRON 1
G19833 Phvul.005G131400        GCTACTGTTGCTCAGgtagaagacaacgtttgtggtcagtgaggtttcga 1077
OAC Rex KF303289               GCCACTGTTGCTCAGgtagaagacaacgtttgtggtcagtgaggtttcga 1150
                               ** ***********************************************

G19833 Phvul.005G131400        tttggattgtacttattaaccaagagaatggttggttataaataactttt 1127
OAC Rex KF303289               tttggattgtacttattaaccaagagaatggttggttataaataactttt 1200
                               **************************************************

G19833 Phvul.005G131400        gcacttttttagagaagttaagcgatgaaatttctataaaaactaaatac 1177
OAC Rex KF303289               gcacttttttagagaagttaagcgatgaaatttctataaaaactaaatac 1250
                               **************************************************

G19833 Phvul.005G131400        ataagtgggttttagtttatgaataatgacagaagtttactttagttttt 1227
OAC Rex KF303289               ataagtgggttttagtttatgaataatgacagaagtttactttaattttt 1300
                               ******************************************** *****

G19833 Phvul.005G131400        ttttttatgagggcttgggaagagaagaaatttatttaaagtggggttaa 1277
OAC Rex KF303289               ttttt-atgagggcttgggaagagaagaaatttatttaaagtggggttaa 1349
                               ***** ********************************************

G19833 Phvul.005G131400        aatgatttgaagttgaagtatgacttttgtgttggttgttattgttgttg 1327
OAC Rex KF303289               aatgatttgacgttgaagtatgacttttgtgttggttgttattgttgttg 1399
                               ********** ***************************************
                                                                                 EXON 2
G19833 Phvul.005G131400        tggtttcagGGATATGGTATGACTGAAACTTGTGGGATTGTTTCTGTGGA 1377
OAC Rex KF303289               tggtttcagGGATATGGTATGACTGAAACTTGTGGGATTGTTTCTGTGGA 1449
                               **************************************************

G19833 Phvul.005G131400        GAATACAAGGTTGGGGATTCGGCATAGTGGCTCTGCGGGAATGCTTGTTG 1427
OAC Rex KF303289               GAATACAAGGATGGGGATTCGGCATAGTGGCTCTGCAGGAATGCTTGTTG 1499
                               ********** ************************* *************

G19833 Phvul.005G131400        CGGGAGTGGAAGCTCAAATAGTTAGCGTGGATACACTGAAGGCTCTCCCT 1477
OAC Rex KF303289               CGGGAGTGGAAGCTCAAATAGTTAGCGTGGATACACTGAAGGCTCTCCCT 1549
                               **************************************************
```

```
                                                                                 INTRON 2
G19833 Phvul.005G131400        CCAGGACAATTGGGGGAGATATGGGTGCGGGGTCCTAATATGATGCAAGg 1527
OAC Rex KF303289               CCTGGACAATTGGGGGAGATATGGGTGCGGGGTCCTAATATGATGCAAGg 1599
                               ** ***********************************************

G19833 Phvul.005G131400        tacactccctgaaatgtggggtttattattgtattgtatcgttgccatgg 1577
OAC Rex KF303289               tacactccctgaaatgtgaggtttattattctattgtatcgttgccatgg 1649
                               ****************** *********** *******************

G19833 Phvul.005G131400        cctatagtagaaa-tgatttgaaaaaaagatgtattttctgctgtcttat 1626
OAC Rex KF303289               cctatagtagaaaataatttgaaaaaaaaatgtattttctgctgtcttat 1699
                               ************* * ************ *********************

G19833 Phvul.005G131400        gtggaggaagaatcttaggtagacaggttaagtagatttggtttaaaact 1676
OAC Rex KF303289               gtggaggaagagtcttaggtagacaggttaagtagatttggtttaaaact 1749
                               *********** **************************************

G19833 Phvul.005G131400        gtgcttgaatctgcctgttccgttttaaattgattctgctagacttgttt 1726
OAC Rex KF303289               gtgcttgaatctgcctgttccgttttaaattgattctgctagacttgttt 1799
                               **************************************************

G19833 Phvul.005G131400        tggtcgctgtccgtgtacattatacatttctctggctctttcttgtcgag 1776
OAC Rex KF303289               tggtcgctgtccgtgtacattatacatttctctggctctttcttgtcgag 1849
                               **************************************************

G19833 Phvul.005G131400        gaaacactaatttgttttaagcttggtggtgcctaatgttggctggtggg 1826
OAC Rex KF303289               gaaacacta-tttgttttaagcttggtggtgcctaa-------------- 1884
                               ********* **************************

G19833 Phvul.005G131400        tgtaaattcggaagcaagacagattagatcatgaccttaaacatgt-agt 1875
OAC Rex KF303289               -----attcggaagcaagacagattagatcatgaccttagacatgttagt 1929
                                    ********************************** ****** ***

G19833 Phvul.005G131400        cctgtatgataaaagaaaaaaagacatggtctggattctacattgtatgc 1925
OAC Rex KF303289               cctgtatgataaaagaaaaaaagacatggtctggattctacattgtatgc 1979
                               **************************************************

G19833 Phvul.005G131400        tcaagttgtttgccaaaaaaagactatcatcatgttacagtgttcttgtt 1975
OAC Rex KF303289               tcaagttgtttgc---gaaaagactatcatcatgttacagtgttcttgtt 2026
                               *************    *********************************

G19833 Phvul.005G131400        aggctgcttgatgtatagcagaaagggatttgcggttattggaagatgca 2025
OAC Rex KF303289               aggctgcttgatgtatagcagaaagggatttgcggttattagaagatgca 2076
                               **************************************** *********

G19833 Phvul.005G131400        tcatacactggccaatgtcctactctaatttttctaatcaatatttcacg 2075
OAC Rex KF303289               tcatacactggccaatgtcctactctaatttttctaatcaatatttcacg 2126
                               **************************************************

G19833 Phvul.005G131400        aatatattttattactttctctgcctcggcaactgttcctccgagagata 2125
OAC Rex KF303289               aatatattttattactttctctgcctcagcaactgttcctctgagagata 2176
                               *************************** ************* ********

G19833 Phvul.005G131400        ttgattgagttgtagctccattctccaaaaatctacatgctttcgtgcat 2175
OAC Rex KF303289               ttgattgagttgtagctccattctccaaaaatctacatgctttcgtgcat 2226
                               **************************************************

G19833 Phvul.005G131400        gaaaaatatttttttgtgaagttgaatttcatccaatcactaatttgtat 2225
OAC Rex KF303289               gaaaaatatttttttgtgaagttgaatttcatccaatcactaatttgtat 2276
                               **************************************************

G19833 Phvul.005G131400        aattagttctaaaatgtggtggtgagcatcattttggtacgggagttgag 2275
OAC Rex KF303289               aattagttctaaaatgtggtggtgagcatcattttggtacgggagttgag 2326
                               **************************************************
```

```
G19833 Phvul.005G131400        gtttttatctgactttctaactttagactctccttttcacccagcgttta 2325
OAC Rex KF303289               gtttttatctgactttctaactttagactcttcttttcacctagcgttta 2376
                               ******************************* ********* ********

G19833 Phvul.005G131400        tcaatatggatggtcaaaatggtttgtgctagataatacatcattcatta 2375
OAC Rex KF303289               tcaatatggatggtcaaaatgctttgagctagataatacatcattcatta 2426
                               ********************* **** ***********************

G19833 Phvul.005G131400        tgttgataacgacatatttgtgttcatttccaagtttgaattgtttcatg 2425
OAC Rex KF303289               tgttgataacgacatatttgtgttcaattccaagtttgaattgtttcatg 2476
                               ************************** ***********************

G19833 Phvul.005G131400        gactgttgccaacggaaactgttccttgttgagtgaaaagttaggaattt 2475
OAC Rex KF303289               gactgttgccaacggaaactgttccttgttgagtgaaaagttaggaat-- 2524
                               ************************************************

G19833 Phvul.005G131400        ttattggcaaaatcaattgagcactagagtacatgaaaataattatgctt 2525
OAC Rex KF303289               ----------------------------------------aattatgctt 2534
                                                                       **********

G19833 Phvul.005G131400        agtgctttggctgcttccttcacttttattttgcttccatattcttgtcc 2575
OAC Rex KF303289               agtgctttggctgcttccttcacttttattttgcttccatattcttgtcc 2584
                               **************************************************

G19833 Phvul.005G131400        taaaaatatgtttagggtccttagtttacttcgtatgcagtttggcatta 2625
OAC Rex KF303289               taaaaatatgtttagggtccttagtttacttcgtatgcagtttggcttta 2634
                               ********************************************** ***

G19833 Phvul.005G131400        aattgtattccatggtatcttccttgaagcaatttaataaagctattgca 2675
OAC Rex KF303289               aattgtattccatgggaacttcctagaagcaatttaataaagctattgca 2684
                               *************** * ****** *************************
                                                                                 EXON 3
G19833 Phvul.005G131400        taagttttcagGATATCACAACAATCCCCAAGCCACAAGATTGACTATAG 2725
OAC Rex KF303289               taagttttcagGATATCACAACAATCCCCAAGCCACAAGATTGACTATAG 2734
                               **************************************************

G19833 Phvul.005G131400        ATAAAAAAGGGTGGGTACATACAGGAGATCTTGGATATTTTGACGAGGAT 2775
OAC Rex KF303289               ATAAAAAAGGGTGGGTGCATACAGGAGATCTTGGATATTTTGACGAGGAT 2784
                               **************** *********************************

G19833 Phvul.005G131400        GGGCAACTTTTTGTTGTTGACCGTATCAAAGAACTGATCAAGTATAAAGG 2825
OAC Rex KF303289               GGTCAACTTTTTGTTGTTGACCGTATCAAAGAACTGATCAAGTATAAAGG 2834
                               ** ***********************************************
                                                                                 INTRON 3
G19833 Phvul.005G131400        TTTTCAGgtatgtgatccaaatctcatgtcaatggtaaatttacaatttg 2875
OAC Rex KF303289               TTTTCAGgtatgtgatccaaatctcatgtcaatggtaaatttacaatttg 2884
                               **************************************************

G19833 Phvul.005G131400        tctagcatgttataaattcatgcgtggtcacatgtcccaaatctcaatca 2925
OAC Rex KF303289               tctagcatgtcataaatgcatgcgtggtcacatgtcccaaatctcaatca 2934
                               ********** ****** ********************************

G19833 Phvul.005G131400        aattccccatttacacgagtagtagaaacataacgaggtacttaggcaat 2975
OAC Rex KF303289               aattccccatttacacgagtagtagaaacataacgaggtacttaggcaat 2984
                               **************************************************

G19833 Phvul.005G131400        ttggtgcattacatatatgttaaaccccaaattgtatcagtaagtcaaat 3025
OAC Rex KF303289               ttggtgcattacatatatgttaaaccccaaattgtatcagtaagtcaaat 3034
                               **************************************************

G19833 Phvul.005G131400        tttgtagcttcggtgcatcatgtgtttgctgttctgcgagggtaattatt 3075
OAC Rex KF303289               tttgtagcttcggtgcatcatgtgtttgctgttctgcgtgggtaattatt 3084
                               ************************************** ***********
```

```
G19833 Phvul.005G131400        tgtgtttacaatcattctgggttattcttggcgatgaaaaggatttttca 3125
OAC Rex KF303289               tgtgtttacaatcattctgggttattcttggcgatgaaaaggatttttca 3134
                               **************************************************
                                                                                 EXON 4
G19833 Phvul.005G131400        tccatctgaaactatttcccgtttgccacatcgttctaactagGTTGCAC 3175
OAC Rex KF303289               tccatctgaaactatttcccgtttgccacatcgttctaactagGTTGCAC 3184
                               **************************************************

G19833 Phvul.005G131400        CAGCAGAACTTGAAGGGCTTCTTGTTTCTCACCCTGAAATACTTGATGCA 3225
OAC Rex KF303289               CAGCAGAACTTGAAGGGCTTCTTGTTTCTCACCCAGAAATACTTGATGCT 3234
                               ********************************** **************
                                                                                 INTRON 4
G19833 Phvul.005G131400        GTTGTCATCCCgtaagtttcccatgcatgatatttatttaattattatgg 3275
OAC Rex KF303289               GTTGTCATCCCgtaagtttcccatgcatgatttttatttaattattatgg 3284
                               ******************************* ******************

G19833 Phvul.005G131400        gtacagaagaactataactcatgttattagtaattggaattcataattga 3325
OAC Rex KF303289               ctacagaagaactataactcatgttatttgtaattggaattcataattga 3334
                                *************************** *********************
                                                                                 EXON 5
G19833 Phvul.005G131400        ctcatttccattctgtagATATCCCGATGCAGAAGCTGGTGAGGTTCCAG 3375
OAC Rex KF303289               ctcatttccattctgtagATATCCCGATGCAGAAGCTGGTGAGGTTCCAG 3384
                               **************************************************

G19833 Phvul.005G131400        TTGCCTATGTCGTTCGCTCACCCAATAGTTCATTGACTGAGGAAGATGTT 3425
OAC Rex KF303289               TTGCCTATGTCGTTCGCTCACCCAATAGTTCATTGACTGAGGAAGATGTT 3434
                               **************************************************
                                                                                 INTRON 5
G19833 Phvul.005G131400        CAAAAGTTTATTGCTAAGCAGgtaagaaaaataaacaagcctgatctttg 3475
OAC Rex KF303289               CAAAAGTTTATTGCTAAGCAGgtaagaaaaataaacaagcctgatctttg 3484
                               **************************************************

G19833 Phvul.005G131400        acttgcattatatgttctttactaaatggggttctaaaataaaattgaga 3525
OAC Rex KF303289               acttgcattatatgttctttactaaatggggttctaaaataaaattgaga 3534
                               **************************************************

G19833 Phvul.005G131400        ttaacaaaatgcatgtttttaaagtgaattgccttgccctctttggttct 3575
OAC Rex KF303289               ttaacaaaatgcatgtttttaaagtgaattgccttgccctctttggttct 3584
                               **************************************************

G19833 Phvul.005G131400        gtatttgtatatggatgcttttatggccaattgaaggctacacaaacaag 3625
OAC Rex KF303289               gtatttgtatatggatgcttttatggccaattgaaggctacacaaacaag 3634
                               **************************************************

G19833 Phvul.005G131400        aggggcttattaacatctcataaatcacatcccatctgttcccgacagta 3675
OAC Rex KF303289               aggggcttattaacatctcataaatcacatcccatctgttcccgacagta 3684
                               **************************************************

G19833 Phvul.005G131400        atactggatttgaatattgtttgagtgatcatcagtcatcactgctatgc 3725
OAC Rex KF303289               atactggatttgaatattgtttgagtgatcatcagtcatcactgctatgc 3734
                               **************************************************

G19833 Phvul.005G131400        cttaatgcagcattagttctgtatatacccgtttttgttgacacttattt 3775
OAC Rex KF303289               cttaatgcagcattagttctgtatatacccgtttttgttgacacttattt 3784
                               **************************************************
                                                                                 EXON 6
G19833 Phvul.005G131400        gtatttgtttccagGTAGCACCTTTCAAAAGATTGCGAAGGGTGACATTC 3825
OAC Rex KF303289               gtatttgtttccagGTAGCACCTTTCAAAAGATTGCGAAGGGTGACATTC 3834
                               **************************************************

G19833 Phvul.005G131400        ATCAATGCTGTTCCTAAGACAGCATCGGGAAAAATTCTCCGAAGAGAGCT 3875
OAC Rex KF303289               ATCAATGCTGTTCCTAAGACAGCATCGGGAAAAATTCTCCGAAGAGAGCT 3884
                               **************************************************
```

```
                                                        TGA
G19833 Phvul.005G131400        TATTGAGAAAGTAAGATCCAAAATATGATGCCATGATATGATTTACTTTT 3925
OAC Rex KF303289               TATTGAGAAAGTAAGATCCAAAATATGATGCCATGATATGATTTACACCT 3934
                               **********************************************   *

G19833 Phvul.005G131400        TTTCAAAACCTTTTCGGGGTTCAAATTCTGAAGTATAATGCGAGATAGAG 3975
OAC Rex KF303289               TTTCAAAACCTTTTCGGGGTTCAAATTCTGAAGTATAATGCGAGATAGAG 3984
                               **************************************************

G19833 Phvul.005G131400        CGAATCCCGAAAGGTATGATGGTTGTGGTAGTGGAAAATGGAATAAACTG 4025
OAC Rex KF303289               CGAATCCGGAAAGGTATGATGGTTGTGGTAGTGGAAAATGGAATAAACTG 4034
                               ******* ******************************************

G19833 Phvul.005G131400        TACATAATAGTTCTTATAATCTTAAACTAATAAAGTTATATTGCATATCT 4075
OAC Rex KF303289               TACATAATAGTTCTTATAATCTTAAACTAATAAAATTATATTGCATATCT 4084
                               ********************************** ***************

G19833 Phvul.005G131400        ATCAATTTAGTTCATAAACTGCTATATTTTCCAATTTTAAAGGATCAATT 4125
OAC Rex KF303289               ATCAATTTAGTTCATAAACTGCTATATTTTCCAATTTTAAAGGATCAATT 4134
                               **************************************************

G19833 Phvul.005G131400        TGAT---------------------- 4129
OAC Rex KF303289               TGATGGATTTTTTCAATATGAACAAG 4160
                               ****


OAC Rex sequence:
>KF303289 OAC Rex gene for 4-coumarate:CoA ligase (4CL-4; 4,160 bp); similar to P. vulgaris 4CL locus Phvul.005G131400 (cultivar G19833)
```

TAATTTTTGTATAAAATATTTTGGAGAGAATATTCTTGGTCCATGAAAACTTTAAGAATAAAGAAGAGTTGATTATGATAATGTTATAGAATCAGAGGTTATTGTGGATTCGAAATCCAATTAGAATGCAGAGATCCGGGTATGGCTCCGACGGAATATTCCGGTCACTGAGACCTTCAATCGTTCTCCCCAAACAACCCAACCTCACACTCGTTTCCCATCTATTCAACAGAGTCGCACCATTTCCCTCCAAACACGCCCTTATCGATGCCGATTCTTCCCAAACCCTTTCATTCGCGCAGTTGAAGTCGCTAACCGTTAAAGTCTCACACGGCCTCCTCCGTCTCGGCGTCGCCAAAAACGACGTCGTCCTCTTCCTTGCCCCCAACGATATCCGCTACATCGTCTGCTTCCTCGCTGTCACCTCCCTCGGCGCCATCGTCACTACCGTCAATCCCGCCTACACCGCCGCCGAGGTCGCCAAACAGGCCAAAGACTCCAATCCCAAATTTGTCGTCACTGTCCCCGAACTCTGGGACAAGCTCCAACACCTCAACCTCCCCGCTGTGTTCCTTCATTCCCGCGCGCACTCCAACGCGACTAGCTTCGACGCGCTCGTCCATCTCGCAGGGTCGCCTACCGAGTTTCCGAAAGTTGACGTCAAGCAGAGTGACACGGCGGTGCTGCTGTACTCCTCCGGGACCACTGGCCTGAGCAAGGGCGTGGTTCTAACGCACGGGAACTTCGTGGCGGCATCGTTGATGATAGGATTGGACGATGATTTGGCCGGGGAGATGCATAGCGTGTTCCTCTGCGTTCTGCCGATGTTTCATGTGTTTGGTTTGATGGTGATTTCGTACTCGCAGCTTCAGAGGGGCAGTGCTGTGGTGTCTCTGAAGAAGTTTGAGTTTGAACTGGTTTTGAAGACGATTGAGAAGTTTAGTGTGACGCATTTGTGGGTTGTGCCTCCGATTATTCTTGCTTTGGCCAAACATGGTTTGGTTGATAAGTATGATCTTTCTTCGCTGAAGCATATTGGTTCTGGCGCTGCTCCTCTTGGGAAAGAGTTGATGGAGGAGTGTGCTAAACGTTTCCCTCGTGCCACTGTTGCTCAGGTAGAAGACAACGTTTGTGGTCAGTGAGGTTTCGATTTGGATTGTACTTATTAACCAAGAGAATGGTTGGTTATAAATAACTTTTGCACTTTTTTAGAGAAGTTAAGCGATGAAATTTCTATAAAAACTAAATACATAAGTGGGTTTTAGTTTATGAATAATGACAGAAGTTTACTTTAATTTTTTTTTTATGAGGGCTTGGGAAGAGAAGAAATTTATTTAAAGTGGGGTTAAAATGATTTGACGTTGAAGTATGACTTTTGTGTTGGTTGTTATTGTTGTTGTGGTTTCAGGGATATGGTATGACTGAAACTTGTGGGATTGTTTCTGTGGAGAATACAAGGATGGGGATTCGGCATAGTGGCTCTGCAGGAATGCTTGTTGCGGGAGTGGAAGCTCAAATAGTTAGCGTGGATACACTGAAGGCTCTCCCTCCTGGACAATTGGGGGAGATATGGGTGCGGGGTCCTAATATGATGCAAGGTACACTCCCTGAAATGTGAGGTTTATTATTCTATTGTATCGTTGCCATGGCCTATAGTAGAAAATAATTTGAAAAAAAAATGTATTTTCTGCTGTCTTATGTGGAGGAAGAGTCTTAGGTAGACAGGTTAAGTAGATTTGGTTTAAAACTGTGCTTGAATCTGCCTGTTCCGTTTTAAATTGATTCTGCTAGACTTGTTTTGGTCGCTGTCCGTGTACATTATACATTTCTCTGGCTCTTTCTTGTCGAGGAAACACTATTTGTTTTAAGCTTGGTGGTGCCTAAATTCGGAAGCAAGACAGATTAGATCATGACCTTAGACATGTTAGTCCTGTATGATAAAAGAAAAAAAGACATGGTCTGGATTCTACATTGTATGCTCAAGTTGTTTGCGAAAAGACTATCATCATGTTACAGTGTTCTTGTTAGGCTGCTTGATGTATAGCAGAAAGGGATTTGCGGTTATTAGAAGATGCATCATACACTGGCCAATGTCCTACTCTAATTTTTCTAATCAATATTTCACGAATATATTTTATTACTTTCTCTGCCTCAGCAACTGTTCCTCTGAGAGATATTGATTGAGTTGTAGCTCCATTCTCCAAAAATCTACATGCTTTCGTGCATGAAAAATATTTTTTTGTGAAGTTGAATTTCATCCAATCACTAATTTGTATAATTAGTTCTAAAATGTGGTGGTGAGCATCATTTTGGTACGGGAGTTGAGGTTTTTATCTGACTTTCTAACTTTAGACTCTTCTTTTCACCTAGCGTTTATCAATATGGATGGTCAAAATGCTTTGAGCTAGATAATACATCATTCATTATGTTGATAACGACATATTTGTGTTCAATTCCAAGTTTGAATTGTTTCATGGACTGTTGCCAACGGAAACTGTTCCTTGTTGAGTGAAAAGTTAGGAATAATTATGCTTAGTGCTTTGGCTGCTTCCTTCACTTTTATTTTGCTTCCATATTCTTGTCCTAAAAATATGTTTAGGGTCCTTAGTTTACTTCGTATGCAGTTTGGCTTTAAATTGTATTCCATGGGAACTTCCTAGAAGCAATTTAATAAAGCTATTGCATAAGTTTTCAGGATATCACAACAATCCCCAAGCCACAAGATTGACTATAGATAAAAAAGGGTGGGTGCATACAGGAGATCTTGGATATTTTGACGAGGATGGTCAACTTTTTGTTGTTGACCGTATCAAAGAACTGATCAAGTATAAAGGTTTTCAGGTATGTGATCCAAATCTCATGTCAATGGTAAATTTACAATTTGTCTAGCATGTCATAAATGCATGCGTGGTCACATGTCCCAAATCTCAATCAAATTCCCCATTTACACGAGTAGTAGAAACATAACGAGGTACTTAGGCAATTTGGTGCATTACATATATGTTAAACCCCAAATTGTATCAGTAAGTCAAATTTTGTAGCTTCGGTGCATCATGTGTTTGCTGTTCTGCGTGGGTAATTATTTGTGTTTACAATCATTCTGGGTTATTCTTGGCGATGAAAAGGATTTTTCATCCATCTGAAACTATTTCCCGTTTGCCACATCGTTCTAACTAGGTTGCACCAGCAGAACTTGAAGGGCTTCTTGTTTCTCACCCAGAAATACTTGATGCTGTTGTCATCCCGTAAGTTTCCCATGCATGATTTTTATTTAATTATTATGGCTACAGAAGAACTATAACTCATGTTATTTGTAATTGGAATTCATAATTGACTCATTTCCATTCTGTAGATATCCCGATGCAGAAGCTGGTGAGGTTCCAGTTGCCTATGTCGTTCGCTCACCCAATAGTTCATTGACTGAGGAAGATGTTCAAAAGTTTATTGCTAAGCAGGTAAGAAAAATAAACAAGCCTGATCTTTGACTTGCATTATATGTTCTTTACTAAATGGGGTTCTAAAATAAAATTGAGATTAACAAAATGCATGTTTTTAAAGTGAATTGCCTTGCCCTCTTTGGTTCTGTATTTGTATATGGATGCTTTTATGGCCAATTGAAGGCTACACAAACAAGAGGGGCTTATTAACATCTCATAAATCACATCCCATCTGTTCCCGACAGTAATACTGGATTTGAATATTGTTTGAGTGATCATCAGTCATCACTGCTATGCCTTAATGCAGCATTAGTTCTGTATATACCCGTTTTTGTTGACACTTATTTGTATTTGTTTCCAGGTAGCACCTTTCAAAAGATTGCGAAGGGTGACATTCATCAATGCTGTTCCTAAGACAGCATCGGGAAAAATTCTCCGAAGAGAGCTTATTGAGAAAGTAAGATCCAAAATATGATGCCATGATATGATTTACACCTTTTCAAAACCTTTTCGGGGTTCAAATTCTGAAGTATAATGCGAGATAGAGCGAATCCGGAAAGGTATGATGGTTGTGGTAGTGGAAAATGGAATAAACTGTACATAATAGTTCTTATAATCTTAAACTAATAAAATTATATTGCATATCTATCAATTTAGTTCATAAACTGCTATATTTTCCAATTTTAAAGGATCAATTTGATGGATTTTTTCAATATGAACAAG

```
E) Nucleotide sequence comparison of the 4CL locus Phvul.005G184500 between two common bean genotypes

G19833 Phvul.005G184500       --------------------------------CTCTGATTTCCTCTTCTC 18
OAC Rex KF303290              TTATTATTACTATTACATTACAAATACACAACCTCTGATTTCCTCTTCTC 50
                                                              ******************
                                                               ATG              EXON 1
G19833 Phvul.005G184500       TTCGAATACACAAACGAAAACACAGTTTCAGAAATGGCGGTTGAAACCCA 68
OAC Rex KF303290              TTCGAATACACAAACGAAAACACAGTTTCAGAAATGGCGGTTGAAACCCA 100
                              **************************************************

G19833 Phvul.005G184500       ACAGGGAAAGGACTTCATTTTCAGGTCCAAGCTTCCGGATATATACATCC 118
OAC Rex KF303290              ACAGGGAAAGGACTTCATTTTCAGGTCCAAGCTTCCGGATATATACATCC 150
                              **************************************************

G19833 Phvul.005G184500       CCAAACACCTTCCCCTCCACGCTTACTGCTTCCAAAATCTCCCTGACCAC 168
OAC Rex KF303290              CCAAACACCTTCCCCTCCACGCTTACTGCTTCCAAAATCTCCCTGACCAC 200
                              **************************************************

G19833 Phvul.005G184500       GCCTCACGCCCCTGCTTGATCAATGCCCCCACCGGAGAAGTCTACACCTA 218
OAC Rex KF303290              GCCTCACGCCCCTGCTTGATCAATGCCCCCACCGGAGAAGTCTTTACCTA 250
                              *******************************************  *****

G19833 Phvul.005G184500       CTCCGACGTCGACGCCACCGCCCGCAAAGTGGCGGCCGGCCTCCACAAAT 268
OAC Rex KF303290              CTCCGACGTCGACGCCACCGCCCGCAAAGTGGCGGCCGGCCTCCACAAAT 300
                              **************************************************

G19833 Phvul.005G184500       TGGGCGTGCGACAGGGCGAGGTCATCATGATCCTTCTCCCCAATTGCCCC 318
OAC Rex KF303290              TGGGCGTGCGACAGGGCGAGGTCATCATGATCCTTCTCCCCAATTGCCCC 350
                              **************************************************

G19833 Phvul.005G184500       GAATTCGTGTTCGCTTTTCTCGGGGCGTCTCATCGCGGCGCCATGGCCAC 368
OAC Rex KF303290              GAATTCGTGTTCGCTTTTCTCGGGGCGTCTCATCGCGGCGCCATGGCCAC 400
                              **************************************************

G19833 Phvul.005G184500       TGCGGCCAACCCCTTCTTCACTGCGGCGGAGATTGCGAAGCAGGCCAAGG 418
OAC Rex KF303290              TGCGGCCAACCCCTTCTTCACTGCGGCGGAGATTGCGAAGCAGGCCAAGG 450
                              **************************************************

G19833 Phvul.005G184500       CGTCCAACGCCAAGGTGCTCATCACGCAGGCCTCTTACTACGAAAAGGTG 468
OAC Rex KF303290              CGTCCAACGCCAAGGTGCTCATCACGCAGGCCTCTTACTACGAAAAGGTG 500
                              **************************************************

G19833 Phvul.005G184500       AAGGAGCTGGAGGAGGTGAAGCTGGTGTTCGTGGACTCGTGTCCGGCGGC 518
OAC Rex KF303290              AAGGAGCTGGAGGAGGTGAAGCTGGTGTTCGTGGACTCGTGTCCGGCGGC 550
                              **************************************************

G19833 Phvul.005G184500       GGAGGCAGATGGCGGTGGTCACATCCATTTCTCGGAGCTGTGTGAGGGAG 568
OAC Rex KF303290              GGAGGCATATGGCGGTGGTCACATCCATTTCTCGGAGCTGTGTGAGGGAG 600
                              ******* ******************************************

G19833 Phvul.005G184500       GCGGCGAGATGGATGAGGTGGATATAAGAGCGGATGACGTGGTGGCGTTG 618
OAC Rex KF303290              GCGGCGAGATGGATGAGGTGGATATAAGAGCGGATGACGTGGTGGCGTTG 650
                              **************************************************

G19833 Phvul.005G184500       CCGTATTCGTCGGGGACAACGGGGCTTCCGAAGGGTGTGATGCTGACGCA 668
OAC Rex KF303290              CCGTATTCGTCGGGGACAACGGGGCTTCCGAAGGGTGTTATGCTGACGCA 700
                              ************************************** ***********

G19833 Phvul.005G184500       CAAAGGGCTGGTGACGAGCATTGCTCAGCAGGTCGACGGCGAGAATCCGA 718
OAC Rex KF303290              CAAAGGGCTGGTGACGAGCATTGCTCAGCAGGTCGACGGCGAGAATCCGA 750
                              **************************************************

G19833 Phvul.005G184500       ACCTCTACTACCACTCCGACGACACCATCCTCTGCGTGCTTCCCCTGTTC 768
OAC Rex KF303290              ACCTCTACTACCACTCCGACGACACCATCCTCTGCGTGCTTCCCCTGTTC 800
                              **************************************************
G19833 Phvul.005G184500       CACATATACTCCCTCAACTCTGTTCTGCTCTGTGGCTTGCGTTCCAAGGC 818
OAC Rex KF303290              CACATATACTCCCTCAACTCTGTTCTGCTCTGTGGCTTGCGTTCCAAGGC 850
                              **************************************************

G19833 Phvul.005G184500       TGCGATTCTCCTCATGCCCAAGTTCGAAATCAAAGCCCTCCTCGCCCTCA 868
OAC Rex KF303290              TGCGATTCTCCTCATGCCCAAGTTCGAAATCAAAGCCCTCCTCGCCCTCA 900
                              **************************************************

G19833 Phvul.005G184500       TTCACAAACACAGAGTCACCGTTGCAGCCGTCGTTCCCCCCATCGTTCTC 918
OAC Rex KF303290              TTCACAAACACAGAGTCACCGTTGCAGCCGTCGTTCCCCCCATCGTTCTC 950
                              **************************************************

G19833 Phvul.005G184500       GCCATTGCCAGATCACCCGATCTCGCCAGCTACGACCTCTCTTCCATCAG 968
OAC Rex KF303290              GCCATTGCCAGATCACCCGATCTCGCCAGCTACGACCTCTCTTCCATCAG 1000
                              **************************************************

G19833 Phvul.005G184500       GCTCTTCAAGTCCGGTGGAGCTCCTCTCGGCAAAGACCTCGAAGACAGTG 1018
OAC Rex KF303290              GCTCTTCAAGTCCGGTGGAGCTCCGCTCGGCAAAGACCTGGAAGACAGTG 1050
                              ************************ ************** **********

G19833 Phvul.005G184500       TGAGGTCCAAATTCCCCACCGCCAGACTCGGCCAGGGTTACGGCATGACG 1068
OAC Rex KF303290              TGAGGTCCAAATTCCCCACCGCCAGACTCGGCCAGGGTTACGGCATGACG 1100
                              **************************************************

G19833 Phvul.005G184500       GAGGCGGGCCCCGTGCTCACAATGTCCTTAGCTTTTGCCAAAGAAGCCAT 1118
OAC Rex KF303290              GAGGCGGGCCCCGTGCTCACAATGTCCTTAGCTTTTGCTAAAGAAGCCAT 1150
                              ************************************** ***********

G19833 Phvul.005G184500       GCACGTGAAAGCAGGGGCTTGTGGAACCGTTGTGAGAAACGCGGAGATGA 1168
OAC Rex KF303290              GCACGTGAAAGCAGGGGCTTGTGGAACCGTTGTGAGAAACGCGGAGATGA 1200
                              **************************************************

G19833 Phvul.005G184500       AGATTGTGGATCCCGAAACCGCCCAATCCCTACCTCGAAACCACTCCGGT 1218
OAC Rex KF303290              AGATCGTGGATCCCGAAACCGCCCAATCGCTACCTCGAAACCACTCCGGT 1250
                              **** *********************** *********************
                                                                                INTRON 1
G19833 Phvul.005G184500       GAAATCTGCATAAGAGGCGACCAGATCATGAAAGgtacctatctacctac 1268
OAC Rex KF303290              GAAATCTGCATAAGAGGCGACCAGATCATGAAAGgtacctatctacctac 1300
                              **************************************************

G19833 Phvul.005G184500       cctcattatttttagtttgaaaaggtggtcgaacattgaaatcatgtaca 1318
OAC Rex KF303290              cctcattatttttagtttgaaaaggtggtcgaacattgaaatcatgtaca 1350
                              **************************************************

G19833 Phvul.005G184500       gcattgattaaggttgttgtgttgttggcgtgtgttggaagaaaaaggtg 1368
OAC Rex KF303290              gcattgattaaggttgttgtgttgttggcgtgtgttggaagaaaaaagtg 1400
                              ********************************************** ***
                                                                                EXON 2
G19833 Phvul.005G184500       aatgaatgaatgaacaatgtatgaatgaatgaatcagGTTATCTGAACGA 1418
OAC Rex KF303290              aatgaatgaatgaacaatgtatgaatgaatgaatcagGTTATCTGAACGA 1450
                              **************************************************

G19833 Phvul.005G184500       CAAAGAGGCAACAGAGAGGACGATAGACAAAGAGGGTTGGTTGCACACAG 1468
OAC Rex KF303290              CAAAGAGGCAACAGAGAGGACGATAGACAAAGAGGGTTGGTTGCACACAG 1500
                              **************************************************

G19833 Phvul.005G184500       GTGACATCGGTTACATCGACGAAGACGATGAGTTATTCATAGTTGATCGG 1518
OAC Rex KF303290              GTGACATCGGTTACATCGACGAAGACGATGAGTTATTCATAGTTGATAGG 1550
                              *********************************************** **

G19833 Phvul.005G184500       TTAAAGGAACTGATCAAGTACAAAGGCTTTCAGGTGGCTCCGGCTGAACT 1568
OAC Rex KF303290              TTAAAGGAACTCATCAAGTACAAAGGCTTTCAGGTGGCTCCGGCTGAACT 1600
                              *********** **************************************
```

```
G19833 Phvul.005G184500       TGAAGCCCTTCTTCTCACTCATCCCAACATCTCTGACGCCGCCGTCGTTC 1618
OAC Rex KF303290              TGAAGCCCTTCTTCTCACTCATCCCAACATCTCTGACGCCGCCGTCGTTC 1650
                              **************************************************
                                                                                INTRON 2
G19833 Phvul.005G184500       Cgtaactctctcatctcatcatctcatctcaatctctttcactgaccatt 1668
OAC Rex KF303290              Cgtaactctctcatctcatcatctcatctcaatctctttcactgaccatt 1700
                              **************************************************
                                                                                EXON 3
G19833 Phvul.005G184500       ctatatattatcctctgatgaaccataaatttcagAATGAAGGATGAAGC 1718
OAC Rex KF303290              ctatatattatcctctgatgaaccataaatttcagAATGAAGGATGAAGC 1750
                              **************************************************

G19833 Phvul.005G184500       CGCGGGAGAAGTACCTGTTGCATTTGTGGTGAGATCAAATGCTTGCATCG 1768
OAC Rex KF303290              CGCGGGAGAAGTACCTGTTGCATTTGTGGTGAGATCAAATGCTTGCATCG 1800
                              **************************************************
                                                                                INTRON 3
G19833 Phvul.005G184500       ACACAACCCAGGATGAAATCAAACACTTTATCTCCAAACAAgtacctctt 1818
OAC Rex KF303290              ACACAACCCAGGATGAAATCAAACACTTTATCTCCAAACAAgtacctctt 1850
                              **************************************************

G19833 Phvul.005G184500       ttcctcatcaatgcacttttattacactgcaataactatactgtttacac 1868
OAC Rex KF303290              ttcctcatcaatgcacttttattacactgcaataactatactgtttacac 1900
                              **************************************************
                                                                                EXON 4
G19833 Phvul.005G184500       acgtagtttatctgaaatgtgcagGTGGTGTTTTACAAAAGAATAAGCAC 1918
OAC Rex KF303290              acgtagttcatctgaaatgtgcagGTGGTGTTTTACAAAAGAATAAGCAC 1950
                              ******** *****************************************

G19833 Phvul.005G184500       AGTGTTCTTCGTTGATGCAATTCCCAAGTCACCATCAGGCAAAATCTTGC 1968
OAC Rex KF303290              AGTGTTCTTCGTTGATGCAATTCCCAAGTCACCATCAGGCAAAATCTTGC 2000
                              **************************************************
                                                                             TGA
G19833 Phvul.005G184500       GTAAGGATCTACGAGCAAAGCTAGCAGCACCTGCAGCTGTATCAAAATGA 2018
OAC Rex KF303290              GTAAGGATCTACGAGCAAAGCTAGCAGCACCTGCAGCTGTATCAAAATGA 2050
                              **************************************************

G19833 Phvul.005G184500       TGTGACATCAATGAATCCGTTCTCGTTATTATACAATACAATGTCCCCAA 2068
OAC Rex KF303290              TGTGAAATCAATGAATCCGTTCTCGTTATTATACAATACAATGTCCCCAA 2100
                              ***** ********************************************

G19833 Phvul.005G184500       CAACCTTCAACAACACACAAGCAACACCGATTATTGAGAGCCTTCACCCT 2118
OAC Rex KF303290              CGACCTTCAACAACACACAAGCAACACCGATTATTGAGAGCCTTCACCCT 2150
                              * ************************************************

G19833 Phvul.005G184500       CTTGCTTTCGTATTTTCTTCTGTACACCATTTTCACAATCTAATACCAAA 2168
OAC Rex KF303290              CTTGCTTTCGTATTTTCTTCTGTACACCATTTTCACAATCTAATACCAAA 2200
                              **************************************************

G19833 Phvul.005G184500       ATATTTTTTAAGCAACATCATTTCAGTTTTAATTTTGAAATTACGAAATT 2218
OAC Rex KF303290              ATATTTTTTAAGCAACATCATTTCAGTTTTAATTTTGAAATTATGAAATT 2250
                              ******************************************* ******

G19833 Phvul.005G184500       TAAATAAACTCGTGTTCCTATATTTTATTTATAAATAAGCAATCGT---- 2264
OAC Rex KF303290              TAAATAAACTCTTGTTCCTATATTTTATTTATAAATAAGCAATCGTTATC 2300
                              *********** **********************************

G19833 Phvul.005G184500       --------------------
OAC Rex KF303290              AGTAATTATTTTATCAACAT 2320


OAC Rex sequence:
>KF303290 OAC Rex gene for 4-coumarate:CoA ligase (4CL-5; 2,320 bp); similar to P. vulgaris acyl-CoA synthetase locus Phvul.005G184500 (cultivar G19833)
```

TTATTATTACTATTACATTACAAATACACAACCTCTGATTTCCTCTTCTCTTCGAATACACAAACGAAAACACAGTTTCAGAAATGGCGGTTGAAACCCAACAGGGAAAGGACTTCATTTTCAGGTCCAAGCTTCCGGATATATACATCCCCAAACACCTTCCCCTCCACGCTTACTGCTTCCAAAATCTCCCTGACCACGCCTCACGCCCCTGCTTGATCAATGCCCCCACCGGAGAAGTCTTTACCTACTCCGACGTCGACGCCACCGCCCGCAAAGTGGCGGCCGGCCTCCACAAATTGGGCGTGCGACAGGGCGAGGTCATCATGATCCTTCTCCCCAATTGCCCCGAATTCGTGTTCGCTTTTCTCGGGGCGTCTCATCGCGGCGCCATGGCCACTGCGGCCAACCCCTTCTTCACTGCGGCGGAGATTGCGAAGCAGGCCAAGGCGTCCAACGCCAAGGTGCTCATCACGCAGGCCTCTTACTACGAAAAGGTGAAGGAGCTGGAGGAGGTGAAGCTGGTGTTCGTGGACTCGTGTCCGGCGGCGGAGGCATATGGCGGTGGTCACATCCATTTCTCGGAGCTGTGTGAGGGAGGCGGCGAGATGGATGAGGTGGATATAAGAGCGGATGACGTGGTGGCGTTGCCGTATTCGTCGGGGACAACGGGGCTTCCGAAGGGTGTTATGCTGACGCACAAAGGGCTGGTGACGAGCATTGCTCAGCAGGTCGACGGCGAGAATCCGAACCTCTACTACCACTCCGACGACACCATCCTCTGCGTGCTTCCCCTGTTCCACATATACTCCCTCAACTCTGTTCTGCTCTGTGGCTTGCGTTCCAAGGCTGCGATTCTCCTCATGCCCAAGTTCGAAATCAAAGCCCTCCTCGCCCTCATTCACAAACACAGAGTCACCGTTGCAGCCGTCGTTCCCCCCATCGTTCTCGCCATTGCCAGATCACCCGATCTCGCCAGCTACGACCTCTCTTCCATCAGGCTCTTCAAGTCCGGTGGAGCTCCGCTCGGCAAAGACCTGGAAGACAGTGTGAGGTCCAAATTCCCCACCGCCAGACTCGGCCAGGGTTACGGCATGACGGAGGCGGGCCCCGTGCTCACAATGTCCTTAGCTTTTGCTAAAGAAGCCATGCACGTGAAAGCAGGGGCTTGTGGAACCGTTGTGAGAAACGCGGAGATGAAGATCGTGGATCCCGAAACCGCCCAATCGCTACCTCGAAACCACTCCGGTGAAATCTGCATAAGAGGCGACCAGATCATGAAAGGTACCTATCTACCTACCCTCATTATTTTTAGTTTGAAAAGGTGGTCGAACATTGAAATCATGTACAGCATTGATTAAGGTTGTTGTGTTGTTGGCGTGTGTTGGAAGAAAAAAGTGAATGAATGAATGAACAATGTATGAATGAATGAATCAGGTTATCTGAACGACAAAGAGGCAACAGAGAGGACGATAGACAAAGAGGGTTGGTTGCACACAGGTGACATCGGTTACATCGACGAAGACGATGAGTTATTCATAGTTGATAGGTTAAAGGAACTCATCAAGTACAAAGGCTTTCAGGTGGCTCCGGCTGAACTTGAAGCCCTTCTTCTCACTCATCCCAACATCTCTGACGCCGCCGTCGTTCCGTAACTCTCTCATCTCATCATCTCATCTCAATCTCTTTCACTGACCATTCTATATATTATCCTCTGATGAACCATAAATTTCAGAATGAAGGATGAAGCCGCGGGAGAAGTACCTGTTGCATTTGTGGTGAGATCAAATGCTTGCATCGACACAACCCAGGATGAAATCAAACACTTTATCTCCAAACAAGTACCTCTTTTCCTCATCAATGCACTTTTATTACACTGCAATAACTATACTGTTTACACACGTAGTTCATCTGAAATGTGCAGGTGGTGTTTTACAAAAGAATAAGCACAGTGTTCTTCGTTGATGCAATTCCCAAGTCACCATCAGGCAAAATCTTGCGTAAGGATCTACGAGCAAAGCTAGCAGCACCTGCAGCTGTATCAAAATGATGTGAAATCAATGAATCCGTTCTCGTTATTATACAATACAATGTCCCCAACGACCTTCAACAACACACAAGCAACACCGATTATTGAGAGCCTTCACCCTCTTGCTTTCGTATTTTCTTCTGTACACCATTTTCACAATCTAATACCAAAATATTTTTTAAGCAACATCATTTCAGTTTTAATTTTGAAATTATGAAATTTAAATAAACTCTTGTTCCTATATTTTATTTATAAATAAGCAATCGTTATCAGTAATTATTTTATCAACAT

```
F) Nucleotide sequence comparison of the 4CL locus Phvul.006G057300 between two common bean genotypes

G19833 Phvul.006G057300       --------------------------------------------------
OAC Rex KF303291              GGAGCATTGTAGTTGGGCTTTGTCAATGACATGATTCAACAAATTACCAA 50

G19833 Phvul.006G057300       ---------------------------------------------TGCAA 5
OAC Rex KF303291              CATAAAGAGCCCCCACGCATGGCAAATTGGCAATGCCATTTCACTTGCAA 100
                                                                           *****

G19833 Phvul.006G057300       CTCATCTAACCTCACTCACCATGAAAAGAGTATGACCACAAATGTGCTTA 55
OAC Rex KF303291              CTCATCTAACCTCACTCACCATGAAAAGAGTATGACCACAAATGTGCTTA 150
                              **************************************************
                                                                     ATG        EXON 1
G19833 Phvul.006G057300       TAATAATCTCACATTCTCAACCCCATTTGAGAGGAGTACATGCAACATCT 105
OAC Rex KF303291              TAATAATCTCACATTCTCAACCCCATTTGAGAGGAGTACATGCAACATCT 200
                              **************************************************

G19833 Phvul.006G057300       TGTGAATTGCTTATCCACACACAACACAAACATGGGAACCTACGTGGAAA 155
OAC Rex KF303291              TGTGAATTGCTTATCCACACACAACACAAACATGGGAACCTACGTGGAAA 250
                              **************************************************

G19833 Phvul.006G057300       GTTTGTCAGGTGTGGAGGAACATGTCTTTCGTAGCCAATACTCACCTGTC 205
OAC Rex KF303291              GTTTGTCAGGTGTGGAGGAACATGTCTTTCGTAGCCAATACTCACCTGTC 300
                              **************************************************

G19833 Phvul.006G057300       CCTATCCCTGACAATGTGACTTTGCCAGAATATGTGCTCCAAAATGCTGA 255
OAC Rex KF303291              CCTATCCCTGACAATGTGACTTTGCCAGAATATGTGCTCCAAAATGCTGA 350
                              **************************************************

G19833 Phvul.006G057300       ATTGTATGGTGAGAAGGTTGCATTTGTGGATGCTGTAACTGGAAAAGGGG 305
OAC Rex KF303291              ATTGTATGGTGAGAAGGTTGCATTTGTGGATGCTGTAACTGGAAAAGGGG 400
                              **************************************************

G19833 Phvul.006G057300       TGAGTTATAGAGAGGTGGTGAGGGATGTAGACAGGTTTTCAAAGGCCTTA 355
OAC Rex KF303291              TGAGTTATAGAGAGGTGGTGAGGGATGTAGACAGGTTTTCAAAGGCCTTA 450
                              **************************************************

G19833 Phvul.006G057300       AGATCTCTTGGTTTGAGGAAAGGGCATGTTGTGATTGTGGTACTTCCAAA 405
OAC Rex KF303291              AGGTCTCTTGGTTTGAGGAAAGGGCATGTTGTGATTGTGGTACTTCCAAA 500
                              ** ***********************************************

G19833 Phvul.006G057300       TGTGGTGGAGTATGCCATTGTTGCTTTGGGGATAATGGCTGCTGGTGGGG 455
OAC Rex KF303291              TGTGGTGGAGTATGCCATTGTTGCTTTGGGGATAATGGCTGCTGGTGGGG 550
                              **************************************************

G19833 Phvul.006G057300       TGTTCTCAGGGGCAAATCCAAGTTCTCATGTCTCAGAAATTAAGAAACAG 505
OAC Rex KF303291              TGTTCTCAGGGGCAAATCCAAGTTCTCATGTCTCAGAAATTAAGAAACAG 600
                              **************************************************

G19833 Phvul.006G057300       GCAGAGTCTGCTGATGCCAAATTAATTGTCACCAATAGCTCAAACTATGA 555
OAC Rex KF303291              GCAGAGTCTGCTGATGCCAAATTAATTGTCACCAATAACTCGAACTATGA 650
                              ************************************* *** ********
                                                                                INTRON 1
G19833 Phvul.006G057300       AAAGgtaactgctttattctttttcatctgtttagtgtttaatactcatg 605
OAC Rex KF303291              AAAGgtaactgctttattctttttcatctgtttagtgtttaatactcatg 700
                              **************************************************

G19833 Phvul.006G057300       acactatgttatatttgattcaatcataattaaaaaatgcatagaaaata 655
OAC Rex KF303291              acactatgttatatttgattcaatcataattaaaaaatgcatagaaaata 750
                              **************************************************

G19833 Phvul.006G057300       aactttaaccaatgagtaaagtgacatttttttcactgtcatctaatcta 705
OAC Rex KF303291              aactttaaccaatgagtaaagtgacatttttttcactgtcatctaatcta 800
```

**************************************************

```
G19833 Phvul.006G057300       aaaattggttcatgtcttatttatattaaacaaaataatgtattatatat 755
OAC Rex KF303291              aaaattggttcatgtcttatttatattaaacaaaataatgtattatatat 850
                              **************************************************

G19833 Phvul.006G057300       atatatatatatatatatatatttcaaaatacaatatgaaatatttgatt 805
OAC Rex KF303291              atatatatatatat--------ttcaaaatacaatatgaaatatttgatt 892
                              **************        ****************************

G19833 Phvul.006G057300       accttattcttgttattgttatttaattttaataaaaactaacatgaata 855
OAC Rex KF303291              accttattcttgttattggtatttaattttaataaaaattaacatgaata 942
                              ****************** ******************* ***********

G19833 Phvul.006G057300       agtataatgtaagggttagataaaaagaattactttataaaaagtcatta 905
OAC Rex KF303291              agtacaatgtaagggttagataaaaagaattactttataaaaagtcatta 992
                              **** *********************************************

G19833 Phvul.006G057300       gtgtgtaattagttgatagagtgaaatttgaaggttatacatagaaaaat 955
OAC Rex KF303291              gtgtgtaattagttgatagagtgaaatttgaaggttatacatagaaaaat 1042
                              **************************************************
                                                                                EXON 2
G19833 Phvul.006G057300       cagagtcttaaagcgtagtttctgagttgatataaacacagGTGAAGGGT 1005
OAC Rex KF303291              cagagtcttaaagcgtagtttttgagttgatataaacacagGTGAAGGGT 1092
                              ********************* ****************************

G19833 Phvul.006G057300       CTAGAGCTACCTGTTATTGTGCTAGGAGATGAAGTTGTTGAAGGTGGCAT 1055
OAC Rex KF303291              CTAGAGCTACCTGTTATTGTGCTAGGAGATGAAGTTGTTGAAGGTGGCAT 1142
                              **************************************************

G19833 Phvul.006G057300       GAATTGGAATAAGTTGCTGGAAGCAGCAGATAGGGCAGGTGATGATTTTG 1105
OAC Rex KF303291              GAATTGGAATAAGTTGCTGGAAGCAGCAGATAGGGCAGGTGATGATTTTG 1192
                              **************************************************

G19833 Phvul.006G057300       CCAAGGAGCCTATTCATCAGAATGATCTTTGTGCCATGCCATTCTCATCA 1155
OAC Rex KF303291              CCAAGGAGCCTATTCATCAGAATGATCTTTGTGCCATGCCATTCTCATCA 1242
                              **************************************************

G19833 Phvul.006G057300       GGCACCACAGGAATGTCAAAGGGTGTGATGCTCACTCATAGAAATCTAGT 1205
OAC Rex KF303291              GGCACCACAGGAATGTCAAAGGGTGTGATGCTCACTCATAGAAATCTAGT 1292
                              **************************************************

G19833 Phvul.006G057300       GGCTAATCTCTGCTCTACACTCTTCACTGTGACTAAGGAAATGGAGGGCC 1255
OAC Rex KF303291              GGCTAATCTCTGCTCCACACTCTTCACTGTGACTAAGGAAATGGAGGGCC 1342
                              *************** **********************************

G19833 Phvul.006G057300       TTGTCACCACACTAGGCTTGATTCCCTTCTTTCACATTTATGGCATCACT 1305
OAC Rex KF303291              TTGTCACCACACTAGGCTTGATTCCCTTCTTTCATATTTATGGCATCACT 1392
                              ********************************** ***************

G19833 Phvul.006G057300       GGAATATGCTGTGCCACTCTTAGGAGCAAAGGGAAGGTTGTGGTTATGGG 1355
OAC Rex KF303291              GGAATATGCTGTGCCACTCTTAGGAGCAAAGGGAAGGTTGTGGTTATGGG 1442
                              **************************************************

G19833 Phvul.006G057300       AAGGTTTGAGTTGAAAAAGTTTCTCAATGCCTTAATCACACATGAGGTCA 1405
OAC Rex KF303291              AAGGTTTGAGTTGAAAAAGTTTCTCAATGCCTTAATCACACATGAGGTCA 1492
                              **************************************************

G19833 Phvul.006G057300       CATTTGCACCCATTGTGCCCCCTATCATTCTCAGTTTGGTGAAAAATCCC 1455
OAC Rex KF303291              CATTTGCACCCATTGTGCCCCCTATCATTCTCAGTTTGGTGAAAAATCCC 1542
                              **************************************************

G19833 Phvul.006G057300       ATAGTAGATGAATTTGACCTCAGCAAGCTAAAACTCCAGGCCATTATGAC 1505
OAC Rex KF303291              ATAGTAGATGAATTTGACCTCAGCAAGCTAAAACTCCAGGCCATTATGAC 1592
                              **************************************************
```

```
G19833 Phvul.006G057300       TGCAGCAGCACCACTTGCACCAGAACTTCTTAATGCTTTTGAACATAAGT 1555
OAC Rex KF303291              TGCAGCAGCACCACTTGCACCAGAACTTCTTAATGCTTTTGAACATAAGT 1642
                              **************************************************
                                                                                INTRON 2
G19833 Phvul.006G057300       TCCCTGGCGTTTCTGTCCAAGAGgttggtacaccattcatcaagcaattc 1605
OAC Rex KF303291              TCCCTGGCGTTTCTGTCCAAGAGgttggtacaccattcatcaagcaattc 1692
                              **************************************************

G19833 Phvul.006G057300       ccttgtacaatattctttgactaaattatttcatgtctatagttctaatt 1655
OAC Rex KF303291              ccttgtacaatattctttgactaaattatttcatgtctatagttctaatt 1742
                              **************************************************

G19833 Phvul.006G057300       atttttcacctgacatatagttgagttccattgactctttctcataaatt 1705
OAC Rex KF303291              atttttcacctgacatatagttgagttccattgactctttctcataaatt 1792
                              **************************************************

G19833 Phvul.006G057300       aagaaaaccaattatctgctgtgttaagattagtgaagactataaactga 1755
OAC Rex KF303291              aagaaaaccaattatctgctgtgttaagattagtgaagactataaactga 1842
                              **************************************************

G19833 Phvul.006G057300       tggagataccaaatcctacaataatttcttcaacatgttattttctgcca 1805
OAC Rex KF303291              tggagataccaaatcctacaataatttcttcaacatgttattttctgcca 1892
                              **************************************************

G19833 Phvul.006G057300       tgaattaatttttacatttgccatctacttcaaatatggtaagtttctca 1855
OAC Rex KF303291              tgaattaatttttacatttgccatctacttcaaatatggtaagtttctca 1942
                              **************************************************

G19833 Phvul.006G057300       taagattttttcatttggtcttttcctctccaaagggattctaaatgaaa 1905
OAC Rex KF303291              taagattttttcatttggtcttttcctctccaaagggattctaaatgaaa 1992
                              **************************************************

G19833 Phvul.006G057300       aacttgtgatgtttatatacgaaccaaacatcatttggtagtaacaaatg 1955
OAC Rex KF303291              aacttgtgatgtttatatacgaaccaaacatcatttggtagtaacaaatg 2042
                              **************************************************

G19833 Phvul.006G057300       ttgtggacaacaactcatttagaaagaaattaaaagagataatgagtgaa 2005
OAC Rex KF303291              ttgtggacaacaactcatttagaaagaaattaaaagagataatgagtgaa 2092
                              **************************************************

G19833 Phvul.006G057300       tagattgtgatatcttttaaggaccaaaacctcattcaagtttgttttct 2055
OAC Rex KF303291              tagattgtgatatcttttaaggaccaaaacctcattcaagtttgttttct 2142
                              **************************************************

G19833 Phvul.006G057300       tttaattatgtaaattgtgttgtgtgacaataatgttatgcatatctagt 2105
OAC Rex KF303291              tttaattatgtaaattgtgttgtgtgacaataatgttatgcatatctagt 2192
                              **************************************************
                                                                                EXON 3
G19833 Phvul.006G057300       atgatgtgtctatcttcctgaagtgctagttttctttggtagGCATATGG 2155
OAC Rex KF303291              atgatgtgtctatcttcctgaagtgctagttttctttggtagGCATATGG 2242
                              **************************************************

G19833 Phvul.006G057300       ACTTACTGAGCACAGCTGCATCACACTCACTCTTGCACAAAAAGGATTGG 2205
OAC Rex KF303291              ACTTACTGAGCACAGCTGCATCACACTCACTCTTGCACAAAAAGGATTGG 2292
                              **************************************************

G19833 Phvul.006G057300       GAAGTCTTCATAAAAATTCAGTGGGATTCGTCCTCCCAAATTTGGAAGTC 2255
OAC Rex KF303291              GAAGTCTTCATAAAAATTCAGTGGGATTCGTCCTCCCAAATTTGGAAGTC 2342
                              **************************************************

G19833 Phvul.006G057300       AAGTTTGTTGATCCTGACACTGGTCGATCCCTTCCTAGGAACACACCTGG 2305
OAC Rex KF303291              AAGTTTGTTGATCCTGACACTGGTCGATCCCTTCCTAGGAACACACCTGG 2392
                              **************************************************
```

```
                                                                                INTRON 3
G19833 Phvul.006G057300       GGAACTTTGTGTGAGAAGCCAATGTGTGATGCAAGgtaagaatggagaaa 2355
OAC Rex KF303291              GGAACTTTGTGTGAGAAGCCAATGTGTGATGCAAGgtaagaatggagaaa 2442
                              **************************************************

G19833 Phvul.006G057300       taatctaccccaaaaaaaccagttataaggttatgatgataaattagact 2405
OAC Rex KF303291              taatctaccccaaaaaaaccagttataaggttatgatgataaattagact 2492
                              **************************************************
                                                                                EXON 4
G19833 Phvul.006G057300       gtgaatgaatataaactatgtgtctttttcattagGTTACTATAAACAGG 2455
OAC Rex KF303291              gtgaatgaatataaactatgtgtctttttcattagGTTACTATAAACAGG 2542
                              **************************************************

G19833 Phvul.006G057300       TGGATGAGACTGCCCAAACCATTGACAAAAATGGATGGCTTCACACAGGT 2505
OAC Rex KF303291              TGGATGAGACTGCCCAAACCATTGACAAAAATGGATGGCTTCACACAGGT 2592
                              **************************************************

G19833 Phvul.006G057300       GACATAGGATTCATAGATGATGAAGAAAATGTCTTTATCATTGATCGTAT 2555
OAC Rex KF303291              GACATAGGATTCATAGATGATGAAGAAAATGTCTTTATCATTGATCGTAT 2642
                              **************************************************
                                                                                INTRON 4
G19833 Phvul.006G057300       CAAGGAGTTGATTAAATACAAAGGCTTCCAAgtaagttcttgatatttca 2605
OAC Rex KF303291              CAAGGAGTTGATTAAATACAAAGGCTTCCAAgtaagttcttgatatttca 2692
                              **************************************************

G19833 Phvul.006G057300       catgcttacaacaggtaaaaaaaaagaatttttaggcactctaaatgaaa 2655
OAC Rex KF303291              catgcttacaacaggtaaaaaaaa-gaatttttaggcactctaaatgaaa 2741
                              ************************ *************************

G19833 Phvul.006G057300       aaaccttacaaaatatgagactttccaactctcattcacgtttgtttttt 2705
OAC Rex KF303291              aaaccttacaaaatatgagactttccaactctcattcacgtttgtttttt 2791
                              **************************************************

G19833 Phvul.006G057300       aaatcagacgagtctatactataagatatcatatttcaaaatgtgtacta 2755
OAC Rex KF303291              aaatcagacgagtctgtactataagatatcatatttcaaaatgtgtacta 2841
                              *************** **********************************

G19833 Phvul.006G057300       caattattttgccatgaaagtatgaatattagagagcacaaattggtgaa 2805
OAC Rex KF303291              caattattttgccaagaaagtatgaatattagaaagcacaaattggtgaa 2891
                              ************** ****************** ****************

G19833 Phvul.006G057300       ggttaaggtatcaaattcccattatagatgccaaaaatagtaaagtaagt 2855
OAC Rex KF303291              ggttaaggtatcaaattcccattatagatgccaaaaatagtaaagtaagt 2941
                              **************************************************
                                                                                EXON 5
G19833 Phvul.006G057300       cattttggatgcatgcagGTTGCTCCTGCAGAGTTAGAGGCCATTTTGCT 2905
OAC Rex KF303291              aattttggatgcatgcagGTTGCTCCTGCAGAGTTAGAGGCCATTTTGCT 2991
                               *************************************************
                                                                                INTRON 5
G19833 Phvul.006G057300       GAGTCATTCATCAGTTGAAGATGCAGCTGTAGTGCCgtaagtaactaaca 2955
OAC Rex KF303291              GAGTCATTCATCAGTTGAAGATGCAGCTGTAGTGCCgtaagtaaccaaca 3041
                              ********************************************* ****

G19833 Phvul.006G057300       tagtctttgttaataaaaaaatgttccttttacgactgcatagtataaat 3005
OAC Rex KF303291              tagtcttcgttaataaaaaaatgttccttttacaactgcatagtataaat 3091
                              ******* ************************* ****************

G19833 Phvul.006G057300       gcaccagcttgtgatttgttctacttttcttcaagtggcatctatgtttt 3055
OAC Rex KF303291              gcaccagcttgtgatttgttctacttttcttcaagtggcatctatgtttt 3141
                              **************************************************

G19833 Phvul.006G057300       agcaaaaaattattttcctcggtgcaagattgtcaatatagttaacattg 3105
OAC Rex KF303291              agcaaaaaattattttccttggtgcaagattgtcaatatagttaacattg 3191
                              ******************* ******************************
```

```
G19833 Phvul.006G057300       ttaatcttacacttctattggaattctagttttcaagaaagtcattgcaa 3155
OAC Rex KF303291              ttaatcttacacttctattggaattctagttttcaagaaagtcattgcaa 3241
                              **************************************************
                                                                                EXON 6
G19833 Phvul.006G057300       tgttatgcttttttctataatggttgtttagATTACCAGATGAAGAGGCA 3205
OAC Rex KF303291              tgttatgcttttttctataatggttgtttagATTACCAGATGAAGAGGCA 3291
                              **************************************************

G19833 Phvul.006G057300       GGGGAAATTCCTGCTGCAAGTGTAGTTTTGAGCCCTGGTGCAAAGGAAAG 3255
OAC Rex KF303291              GGGGAAATCCCTGCTGCAAGTGTAGTTTTGAGCCCTGGTGCAAAGGAAAG 3341
                              ******** *****************************************

G19833 Phvul.006G057300       TGAGGAGGACATCATGAACTATGTTGCCAACAATGCTGCACATTACAAGA 3305
OAC Rex KF303291              TGAGGAGGACATCATGAACTATGTTGCCAACAATGCTGCACATTACAAGA 3391
                              **************************************************

G19833 Phvul.006G057300       AAGTGAGAGTGGTACACTTTGTGGAAACCATACCAAAGTCACCCTCTGGA 3355
OAC Rex KF303291              AAGTGAGAGTGGTACACTTTGTGGAAACCATACCAAAGTCACCCTCTGGA 3441
                              **************************************************

G19833 Phvul.006G057300       AAAATAATGAGGAGGCTCATCAAAGAGAAGATGGTTGAAAAGATAAAGAC 3405
OAC Rex KF303291              AAAATAATGAGGAGGCTCATCAAAGAGAAGATGGTTGAAAAGATAAAGAC 3491
                              **************************************************
                                                          TAA
G19833 Phvul.006G057300       AAGTTCCTTAATAAAATCTGAGAATGTCTAA------------------- 3436
OAC Rex KF303291              AAGTTCCTTAATAAAATCTGAGAATGTCTAAAATCACTCACAAATTATGT 3541
                              *******************************

G19833 Phvul.006G057300       --------------------------------------------------
OAC Rex KF303291              ACTGCTTTGATCATTCATGTCTCCATTATCAGGGTTGCAAACTTGTTAGG 3591

G19833 Phvul.006G057300       --------------------------------------------------
OAC Rex KF303291              TGATGACTCATTTGATGTGAGATTTATTGTTGTGAGAAATATTGGAAAAT 3641

G19833 Phvul.006G057300       --------------------------------------------------
OAC Rex KF303291              AAAATTTGAGACTCTAGTAGATGTAAGCTCTTTGAATTCCCATTTACTTA 3691

G19833 Phvul.006G057300       -----
OAC Rex KF303291              TCTTA 3696


OAC Rex sequence:
>KF303291 OAC Rex gene for 4-coumarate:CoA ligase (4CL-6; 3,696 bp); similar to P. vulgaris 4CL locus Phvul.006G057300 (cultivar G19833)
```

GGAGCATTGTAGTTGGGCTTTGTCAATGACATGATTCAACAAATTACCAACATAAAGAGCCCCCACGCATGGCAAATTGGCAATGCCATTTCACTTGCAACTCATCTAACCTCACTCACCATGAAAAGAGTATGACCACAAATGTGCTTATAATAATCTCACATTCTCAACCCCATTTGAGAGGAGTACATGCAACATCTTGTGAATTGCTTATCCACACACAACACAAACATGGGAACCTACGTGGAAAGTTTGTCAGGTGTGGAGGAACATGTCTTTCGTAGCCAATACTCACCTGTCCCTATCCCTGACAATGTGACTTTGCCAGAATATGTGCTCCAAAATGCTGAATTGTATGGTGAGAAGGTTGCATTTGTGGATGCTGTAACTGGAAAAGGGGTGAGTTATAGAGAGGTGGTGAGGGATGTAGACAGGTTTTCAAAGGCCTTAAGGTCTCTTGGTTTGAGGAAAGGGCATGTTGTGATTGTGGTACTTCCAAATGTGGTGGAGTATGCCATTGTTGCTTTGGGGATAATGGCTGCTGGTGGGGTGTTCTCAGGGGCAAATCCAAGTTCTCATGTCTCAGAAATTAAGAAACAGGCAGAGTCTGCTGATGCCAAATTAATTGTCACCAATAACTCGAACTATGAAAAGGTAACTGCTTTATTCTTTTTCATCTGTTTAGTGTTTAATACTCATGACACTATGTTATATTTGATTCAATCATAATTAAAAAATGCATAGAAAATAAACTTTAACCAATGAGTAAAGTGACATTTTTTTCACTGTCATCTAATCTAAAAATTGGTTCATGTCTTATTTATATTAAACAAAATAATGTATTATATATATATATATATATATTTCAAAATACAATATGAAATATTTGATTACCTTATTCTTGTTATTGGTATTTAATTTTAATAAAAATTAACATGAATAAGTACAATGTAAGGGTTAGATAAAAAGAATTACTTTATAAAAAGTCATTAGTGTGTAATTAGTTGATAGAGTGAAATTTGAAGGTTATACATAGAAAAATCAGAGTCTTAAAGCGTAGTTTTTGAGTTGATATAAACACAGGTGAAGGGTCTAGAGCTACCTGTTATTGTGCTAGGAGATGAAGTTGTTGAAGGTGGCATGAATTGGAATAAGTTGCTGGAAGCAGCAGATAGGGCAGGTGATGATTTTGCCAAGGAGCCTATTCATCAGAATGATCTTTGTGCCATGCCATTCTCATCAGGCACCACAGGAATGTCAAAGGGTGTGATGCTCACTCATAGAAATCTAGTGGCTAATCTCTGCTCCACACTCTTCACTGTGACTAAGGAAATGGAGGGCCTTGTCACCACACTAGGCTTGATTCCCTTCTTTCATATTTATGGCATCACTGGAATATGCTGTGCCACTCTTAGGAGCAAAGGGAAGGTTGTGGTTATGGGAAGGTTTGAGTTGAAAAAGTTTCTCAATGCCTTAATCACACATGAGGTCACATTTGCACCCATTGTGCCCCCTATCATTCTCAGTTTGGTGAAAAATCCCATAGTAGATGAATTTGACCTCAGCAAGCTAAAACTCCAGGCCATTATGACTGCAGCAGCACCACTTGCACCAGAACTTCTTAATGCTTTTGAACATAAGTTCCCTGGCGTTTCTGTCCAAGAGGTTGGTACACCATTCATCAAGCAATTCCCTTGTACAATATTCTTTGACTAAATTATTTCATGTCTATAGTTCTAATTATTTTTCACCTGACATATAGTTGAGTTCCATTGACTCTTTCTCATAAATTAAGAAAACCAATTATCTGCTGTGTTAAGATTAGTGAAGACTATAAACTGATGGAGATACCAAATCCTACAATAATTTCTTCAACATGTTATTTTCTGCCATGAATTAATTTTTACATTTGCCATCTACTTCAAATATGGTAAGTTTCTCATAAGATTTTTTCATTTGGTCTTTTCCTCTCCAAAGGGATTCTAAATGAAAAACTTGTGATGTTTATATACGAACCAAACATCATTTGGTAGTAACAAATGTTGTGGACAACAACTCATTTAGAAAGAAATTAAAAGAGATAATGAGTGAATAGATTGTGATATCTTTTAAGGACCAAAACCTCATTCAAGTTTGTTTTCTTTTAATTATGTAAATTGTGTTGTGTGACAATAATGTTATGCATATCTAGTATGATGTGTCTATCTTCCTGAAGTGCTAGTTTTCTTTGGTAGGCATATGGACTTACTGAGCACAGCTGCATCACACTCACTCTTGCACAAAAAGGATTGGGAAGTCTTCATAAAAATTCAGTGGGATTCGTCCTCCCAAATTTGGAAGTCAAGTTTGTTGATCCTGACACTGGTCGATCCCTTCCTAGGAACACACCTGGGGAACTTTGTGTGAGAAGCCAATGTGTGATGCAAGGTAAGAATGGAGAAATAATCTACCCCAAAAAAACCAGTTATAAGGTTATGATGATAAATTAGACTGTGAATGAATATAAACTATGTGTCTTTTTCATTAGGTTACTATAAACAGGTGGATGAGACTGCCCAAACCATTGACAAAAATGGATGGCTTCACACAGGTGACATAGGATTCATAGATGATGAAGAAAATGTCTTTATCATTGATCGTATCAAGGAGTTGATTAAATACAAAGGCTTCCAAGTAAGTTCTTGATATTTCACATGCTTACAACAGGTAAAAAAAAGAATTTTTAGGCACTCTAAATGAAAAAACCTTACAAAATATGAGACTTTCCAACTCTCATTCACGTTTGTTTTTTAAATCAGACGAGTCTGTACTATAAGATATCATATTTCAAAATGTGTACTACAATTATTTTGCCAAGAAAGTATGAATATTAGAAAGCACAAATTGGTGAAGGTTAAGGTATCAAATTCCCATTATAGATGCCAAAAATAGTAAAGTAAGTAATTTTGGATGCATGCAGGTTGCTCCTGCAGAGTTAGAGGCCATTTTGCTGAGTCATTCATCAGTTGAAGATGCAGCTGTAGTGCCGTAAGTAACCAACATAGTCTTCGTTAATAAAAAAATGTTCCTTTTACAACTGCATAGTATAAATGCACCAGCTTGTGATTTGTTCTACTTTTCTTCAAGTGGCATCTATGTTTTAGCAAAAAATTATTTTCCTTGGTGCAAGATTGTCAATATAGTTAACATTGTTAATCTTACACTTCTATTGGAATTCTAGTTTTCAAGAAAGTCATTGCAATGTTATGCTTTTTTCTATAATGGTTGTTTAGATTACCAGATGAAGAGGCAGGGGAAATCCCTGCTGCAAGTGTAGTTTTGAGCCCTGGTGCAAAGGAAAGTGAGGAGGACATCATGAACTATGTTGCCAACAATGCTGCACATTACAAGAAAGTGAGAGTGGTACACTTTGTGGAAACCATACCAAAGTCACCCTCTGGAAAAATAATGAGGAGGCTCATCAAAGAGAAGATGGTTGAAAAGATAAAGACAAGTTCCTTAATAAAATCTGAGAATGTCTAAAATCACTCACAAATTATGTACTGCTTTGATCATTCATGTCTCCATTATCAGGGTTGCAAACTTGTTAGGTGATGACTCATTTGATGTGAGATTTATTGTTGTGAGAAATATTGGAAAATAAAATTTGAGACTCTAGTAGATGTAAGCTCTTTGAATTCCCATTTACTTATCTTA

```
G) Nucleotide sequence comparison of the 4CL locus Phvul.011G020200 between two common bean genotypes

G19833 Phvul.011G020200      ------------------------------------------------------------
OAC Rex KF303292             CAAGTATCAAGTATGGGTCCATCTTAGTAACCAAGGGTATTAATAGAACATTTTTCATAG 60

                                                             ATG                      EXON 1
G19833 Phvul.011G020200      CCTAAATCCAGACACAACACACAACATAAACAATGTCAGAAAGAGCTGAACCTGAAACAC 60
OAC Rex KF303292             CCTAAATCCAGACACAACACACAACATAAACAATGTCAGAAAGAGCTGAACCTGAAACAC 120
                             ************************************************************

G19833 Phvul.011G020200      CCCAAACACACATTTTCAGATCAAAATTACCAGACATACCCCTCCCCAACAACCTCCTTC 120
OAC Rex KF303292             CCCAAACACACATTTTCAGATCAAAATTACCAGACATACCCCTCCCCAACAACCTCCTTC 180
                             ************************************************************

G19833 Phvul.011G020200      TCCACACATACTGCTTCCTTAAACTTCCCCAAGTTTCCCACCGTACGTGCCTTATCGCCG 180
OAC Rex KF303292             TCCACACATACTGCTTCCTTAAACTTCCCCAAGTTTCCCACCGTACGTGCCTTATCGCCG 240
                             ************************************************************

G19833 Phvul.011G020200      CCGCCAGAACCTATACCTACGCCGAAACTCACAGCGCCTCCCGGAAGGTGGCCGCCGGCA 240
OAC Rex KF303292             CCGCCAGAACCTATACCTACGCCGAAACTCACAGCGCCTCCCGGAAGGTGGCCGCCGGCA 300
                             ************************************************************

G19833 Phvul.011G020200      TGTCGAAGCTGGGCATCCAGAAGGGCGACGCCGTCATGATTCTCCTCCCTAACTCCCCGG 300
OAC Rex KF303292             TGTCGAAGCTGGGCATCCAGAAGGGCGACGCCGTCATGATTCTCCTCCCTAACTCCCCGG 360
                             ************************************************************

G19833 Phvul.011G020200      AGTTCGTGTTCTCCTTCATGGCAGCCTCCATGCTCGGCGCCGTGGCCACAACCGCCAACC 360
OAC Rex KF303292             AGTTCGTGTTCTCCTTCATGGCAGCCTCCATGCTCGGCGCCGTGGCCACAACCGCCAACC 420
                             ************************************************************

G19833 Phvul.011G020200      CCTCCTACACCGCCGCCGAAATCGCCAAGCAACTCGCCGCCTCCAACGCCAAGCTGGTCG 420
OAC Rex KF303292             CCTCCTACACCGCCGCCGAAATCGCCAAGCAACTCGCCGCCTCCAACGCCAAGCTGGTCG 480
                             ************************************************************

G19833 Phvul.011G020200      TCACCCTCTCCGAGAACGTCCCCAAGCTCCACCAGCAAGAGCGCCAACACTTCTTCAAGG 480
OAC Rex KF303292             TCACCCTCTCCGAGAACGTCCCCAAGCTCCACCAGCAAGAGCGCCAACACTTCTTCAAGG 540
                             ************************************************************

G19833 Phvul.011G020200      TGGTCACCGTCGACGACCCGCCGGAGAATTGCACGAGGTTCCCGGAGGGCGACGAGAGCG 540
OAC Rex KF303292             TGGTCACCGTCGACGACCCGCCGGAGAATTGCACGAGGTTCCCGGAGGGCGACGAGAGCG 600
                             ************************************************************

G19833 Phvul.011G020200      GGGTTCCGGAGGTCGAGATATCGGCGGAGGACGCGGTGGCGCTGCCGTTTTCGTCGGGGA 600
OAC Rex KF303292             GGGTTCCGGAGGTCGAGATATCGGCGGAGGACGCGGTGGCGCTGCCGTTTTCGTCGGGGA 660
                             ************************************************************

G19833 Phvul.011G020200      CGACGGGGCTAGCGAAGGGGGTGATTCTGACGCACAAGAGTTTGGTAACGAGCGTGGCGC 660
OAC Rex KF303292             CGACGGGGCTAGCGAAGGGGGTGATTCTGACGCACAAGAGTTTGGTAACGAGCGTGGCGC 720
                             ************************************************************

G19833 Phvul.011G020200      AACAGATGGAGGGAGAGAACCCGAACATGTACCTGAAAGAAGAGGACGTTGTGTTGTGCG 720
OAC Rex KF303292             AACAGATGGAGGGAGAGAACCCGAACATGTACCTGAAAGAAGAGGACGTTGTGTTGTGCG 780
                             ************************************************************

G19833 Phvul.011G020200      TGCTTCCTCTTTTTCACATATTCTCCATGCACATCGCCATGATGTGCGCGCTGAGAGCGG 780
OAC Rex KF303292             TGCTTCCTCTTTTTCACATATTCTCCATGCACATCGCCATGATGTGCGCGCTGAGAGCGG 840
                             ************************************************************

G19833 Phvul.011G020200      GGAGTGCCATTTTGTTGATCGAGAAGTTTGAGATGAGGAAGCTGCTGGAGGCGATTGAGC 840
OAC Rex KF303292             GGAGTGCCATTTTGTTGATCGAGAAGTTTGAGATGAGGAAGCTGCTGGAGGCGATTGAGC 900
                             ************************************************************
```

```
G19833 Phvul.011G020200      GGCAAAGGGTCACGGTGGCGATAGTGGTGCCGCCGCTGGTGGCCGCACTTACGAAGAACA 900
OAC Rex KF303292             GGCAAAGGGTCACGGTGGCGATAGTGGTGCCGCCGCTGGTGGCCGCACTTACGAAGAACA 960
                             ************************************************************

G19833 Phvul.011G020200      GTACGGTGGAGGAGTATGATTTGAGGTCGATACGATTGGTGATATCGGGGGCTGCGCCGC 960
OAC Rex KF303292             GTACGGTGGAGGAGTATGATTTGAGGTCGATACGATTGGTGATATCGGGGGCTGCGCCGC 1020
                             ************************************************************
                                                                                      INTRON 1
G19833 Phvul.011G020200      TGGGGCAACAGGCGGAGGAAGCTCTCCGGAAAAGGTTGCCTAACGCCATTCTGGGACAGg 1020
OAC Rex KF303292             TGGGGCAACAGGCGGAGGAAGCTCTCCGGAAAAGGTTGCCTAACGCCATTCTGGGACAGg 1080
                             ************************************************************

G19833 Phvul.011G020200      tatactcttctttacattgttataaaaagtgaattttaagtctaactgaatcccatacaa 1080
OAC Rex KF303292             tatactcttctttacattgttataaaaagtgaattttaagtctaactgaatcccatacaa 1140
                             ************************************************************

G19833 Phvul.011G020200      tcccataaaatcggttgaatgagatgaagtttgtatctacatataaaatgttataatctc 1140
OAC Rex KF303292             tcccataaaatcggttgaatgagatgaagtttgtatctacatataaaatgttataatctc 1200
                             ************************************************************

G19833 Phvul.011G020200      tatctacaccatgcattgattcattgttcttccatcttatctctctctttctctgaatga 1200
OAC Rex KF303292             tatctacaccatgcattgattcattgttcttccatcttatctctctctttctctgaatga 1260
                             ************************************************************
                                                                                      EXON 2
G19833 Phvul.011G020200      agGGCTATGGAATGACAGAGGGTGGACCAGTTTTGACTATGTCTCTGGGCTTTGCAAAGT 1260
OAC Rex KF303292             agGGCTATGGAATGACAGAGGGTGGACCAGTTTTGACTATGTCTCTGGGCTTTGCAAAGT 1320
                             ************************************************************

G19833 Phvul.011G020200      ATCCATTCCCAACGAAATCTGGTTCATGCGGAACTGTGATCAGAAATGGTGAACTGAAGA 1320
OAC Rex KF303292             ATCCATTCCCAACGAAATCTGGTTCATGCGGAACTGTGATCAGAAATGGTGAACTGAAGA 1380
                             ************************************************************

G19833 Phvul.011G020200      TCATTGACCCTCTAACATCCTTTTCTCTTCCCCGTAACCACTCTGGGGAGATTTGCGTCC 1380
OAC Rex KF303292             TCATTGACCCTCTAACATCCTTTTCTCTTCCCCGTAACCACTCTGGGGAGATTTGCGTCC 1440
                             ************************************************************

G19833 Phvul.011G020200      GTGGGCCACAGATCATGAAAGGGTACCTGAATGACGAGGAGGCCACTGCAGCCGCCATTG 1440
OAC Rex KF303292             GTGGGCCACAGATCATGAAAGGGTACCTGAATGACGAGGAGGCCACTGCAGCCGCCATTG 1500
                             ************************************************************

G19833 Phvul.011G020200      ATGGAGATGGTTGGCTTCACACAGGTGATATTGGGTACGTGGATGATGATGATGAGGTTT 1500
OAC Rex KF303292             ATGGAGATGGTTGGCTTCACACAGGTGATATTGGGTACGTGGATGATGATGATGAGGTTT 1560
                             ************************************************************
                                                                                      INTRON 2
G19833 Phvul.011G020200      TCCTCGTTGACAGGGCCAAGGAACTCATCAAATTCAAAGGCTTTCAGgtacatgcatata 1560
OAC Rex KF303292             TCCTCGTTGACAGGGCCAAGGAACTCATCAAATTCAAAGGCTTTCAGgtacatgcatata 1620
                             ************************************************************

G19833 Phvul.011G020200      tactataacatctcttcttcttcttccttttgtattcactcacctgctattacttttaca 1620
OAC Rex KF303292             tactataacatctcttcttcttcttccttttgtattcactcacctgctattacttttaca 1680
                             ************************************************************
                                                                                      EXON 3
G19833 Phvul.011G020200      gGTGCCGCCGGCAGAACTTGAAGACCTTCTCATGCGCCACCCTTCCATTGCAGATGCAGC 1680
OAC Rex KF303292             gGTGCCGCCGGCAGAACTTGAAGACCTTCTCATGCGCCACCCTTCCATTGCAGATGCAGC 1740
                             ************************************************************
                                                                                      INTRON 3
G19833 Phvul.011G020200      TGTCGTCCCgtaagtaaccatctatcacaatttctatgcaatgatttgaattatttacat 1740
OAC Rex KF303292             TGTCGTCCCgtaagtaaccatctatcacaatttctatgcaatgatttgaattatttacat 1800
                             ************************************************************

G19833 Phvul.011G020200      attaaataataaaaatatctcttacaatattttagatttttaactaaaactaatcttata 1800
OAC Rex KF303292             attaaataataaaaatatctcttacaatattttagatttttaactaaaactaatcttata 1860
                             ************************************************************
```

```
G19833 Phvul.011G020200      ctttaattaccttttttatcagttgttattgatgtataattataaaagttttgaagtcga 1860
OAC Rex KF303292             ctttaattaccttttttatcagttgttattgatgtataattataaaagttttgaagtcga 1920
                             ************************************************************

G19833 Phvul.011G020200      acattcttcttaaatattcttaaatgaggaatccatacgttaacaaagtgttcgatttaa 1920
OAC Rex KF303292             acattcttcttaaatattcttaaatgaggaatccatacgttaacaaagtgttcgatttaa 1980
                             ************************************************************

G19833 Phvul.011G020200      aaaatattaattaaatttttaataattttaatacggatctaataccatttttaaaaaaat 1980
OAC Rex KF303292             aaaatattaattaaatttttaataattttaatacggatctaataccatttttaaaaaaat 2040
                             ************************************************************

G19833 Phvul.011G020200      atatatacattaattttttttaaaaattaaattgatcatataaatttttattataatttt 2040
OAC Rex KF303292             atatatacattaattttttttaaaaattaaattgatcatataaatttttattataatttt 2100
                             ************************************************************

G19833 Phvul.011G020200      aaaaataaagaacaaactttttaaatgataaacatttttattataagtgtttattttata 2100
OAC Rex KF303292             aaaaataaagaacaaactttttaaatgataaacatttttattataagtgtttattttata 2160
                             ************************************************************

G19833 Phvul.011G020200      agttataattactttgtccattataactactatatcataatagtatgagttattttttat 2160
OAC Rex KF303292             agttataattactttgtccattataactactatatcataatagtatgagttattttttat 2220
                             ************************************************************

G19833 Phvul.011G020200      ttttttatctgttatataaatatattactcaataatttaagtataattaataaaatatat 2220
OAC Rex KF303292             ttttttatctgttatataaatatattactcaataatttaagtataattaataaaatatat 2280
                             ************************************************************

G19833 Phvul.011G020200      tattttattattattattataaatttttattctttttataaattagatatataaactaat 2280
OAC Rex KF303292             tattttattattattattataaatttttattctttttataaattagatatataaactaat 2340
                             ************************************************************
                                                                                      EXON 4
G19833 Phvul.011G020200      aatgcaatgttgttattggaacaatccctccaaaataacgttacagGCAGAATGACGATG 2340
OAC Rex KF303292             aatgcaatgttgttattggaacaatccctccaaaataacgttacagGCAGAATGACGATG 2400
                             ************************************************************

G19833 Phvul.011G020200      CCGCCGGTGAAGTTCCGGTCGCTTTTGTCGTGAGCTTTGATCTTACAGAAGATGCTGTAA 2400
OAC Rex KF303292             CCGCCGGTGAAGTTCCGGTCGCTTTTGTCGTGAGCTTTGATCTTACAGAAGATGCTGTAA 2460
                             ************************************************************
                                                                                      INTRON 4
G19833 Phvul.011G020200      AGGATTTTATAGCTAAACAGgtagaattagtaagattaattataaaatatttagaattaa 2460
OAC Rex KF303292             AGGATTTTATAGCTAAACAGgtagaattagtaagattaattataaaatatttagaattaa 2520
                             ************************************************************
                                                                                      EXON 5
G19833 Phvul.011G020200      gagaatgagacgatgtgtttgatgtgatcatagGTGGTGTTTTACAAGAGACTGCACAAA 2520
OAC Rex KF303292             gagaatgagacgatgtgtttgatgtgatcatagGTGGTGTTTTACAAGAGACTGCACAAA 2580
                             ************************************************************

G19833 Phvul.011G020200      GTGTATTTTGTTGACGAGATTCCAAAGTCTTCCACTGGAAAGATATTAAGAAAAGAACTG 2580
OAC Rex KF303292             GTGTATTTTGTTGACGAGATTCCAAAGTCTTCCACTGGAAAGATATTAAGAAAAGAACTG 2640
                             ************************************************************
	       TAG
G19833 Phvul.011G020200      AGAGCAAAGGTGGGGAGCATTATTCAGAGGACCTAGCTACCACTCTTTTCCCATTCTTTT 2640
OAC Rex KF303292             AGAGCAAAGGTGGGGAGCATTATTCAGAGGACCTAGCTACCACTCTTTTCCCATTCTTTT 2700
                             ************************************************************

G19833 Phvul.011G020200      CCTTAATTTCTTCTTCCTTTGCTTTGCTGTTCTTCCTCAATGTGTAATCAAAGGATGCAT 2700
OAC Rex KF303292             CCTTAATTTCTTCTTCCTTTGCTTTGCTGTTCTTCCTCAATGTGTAATCAAAGGATGCAT 2760
                             ************************************************************

G19833 Phvul.011G020200      ATTCTCTTCAGAGATTGAAACTGCATCCAAAAGTATGTTTTCATGGTTCTACAAAGTTGT 2760
OAC Rex KF303292             ATTCTCTTCAGAGATTGAAACTGCATCCAAAAGTATGTTTTCATGGTTCTACAAAGTTGT 2820
                             ************************************************************
```

```
G19833 Phvul.011G020200      GGTCCCATACTTTCTAATTCAGAATTTGTGTTCCCATGGAATTCAGTAGTTGGTAGTGAT 2820
OAC Rex KF303292             GGTCCCATACTTTCTAATTCAGAATTTGTGTTCCCATGGAATTCAGTAGTTGGTAGTGAT 2880
                             ************************************************************

G19833 Phvul.011G020200      AAATAAATGGATGCTGTTATTTATTGATTTCCCTAATTGATGGT---------------- 2864
OAC Rex KF303292             AAATAAATGGATGCTGTTATTTATTGATTTCCCTAATTGATGGTAAGAATTTGGAATTTC 2940
                             ********************************************

G19833 Phvul.011G020200      --------------------
OAC Rex KF303292             AATTTTGCAGTGTTTCATCA 2960


OAC Rex sequence:
>KF303292 OAC Rex gene for 4-coumarate:CoA ligase (4CL-7; 2,960 bp); similar to P. vulgaris acyl-CoA synthetase locus Phvul.011G020200 (cultivar G19833)
```

CAAGTATCAAGTATGGGTCCATCTTAGTAACCAAGGGTATTAATAGAACATTTTTCATAGCCTAAATCCAGACACAACACACAACATAAACAATGTCAGAAAGAGCTGAACCTGAAACACCCCAAACACACATTTTCAGATCAAAATTACCAGACATACCCCTCCCCAACAACCTCCTTCTCCACACATACTGCTTCCTTAAACTTCCCCAAGTTTCCCACCGTACGTGCCTTATCGCCGCCGCCAGAACCTATACCTACGCCGAAACTCACAGCGCCTCCCGGAAGGTGGCCGCCGGCATGTCGAAGCTGGGCATCCAGAAGGGCGACGCCGTCATGATTCTCCTCCCTAACTCCCCGGAGTTCGTGTTCTCCTTCATGGCAGCCTCCATGCTCGGCGCCGTGGCCACAACCGCCAACCCCTCCTACACCGCCGCCGAAATCGCCAAGCAACTCGCCGCCTCCAACGCCAAGCTGGTCGTCACCCTCTCCGAGAACGTCCCCAAGCTCCACCAGCAAGAGCGCCAACACTTCTTCAAGGTGGTCACCGTCGACGACCCGCCGGAGAATTGCACGAGGTTCCCGGAGGGCGACGAGAGCGGGGTTCCGGAGGTCGAGATATCGGCGGAGGACGCGGTGGCGCTGCCGTTTTCGTCGGGGACGACGGGGCTAGCGAAGGGGGTGATTCTGACGCACAAGAGTTTGGTAACGAGCGTGGCGCAACAGATGGAGGGAGAGAACCCGAACATGTACCTGAAAGAAGAGGACGTTGTGTTGTGCGTGCTTCCTCTTTTTCACATATTCTCCATGCACATCGCCATGATGTGCGCGCTGAGAGCGGGGAGTGCCATTTTGTTGATCGAGAAGTTTGAGATGAGGAAGCTGCTGGAGGCGATTGAGCGGCAAAGGGTCACGGTGGCGATAGTGGTGCCGCCGCTGGTGGCCGCACTTACGAAGAACAGTACGGTGGAGGAGTATGATTTGAGGTCGATACGATTGGTGATATCGGGGGCTGCGCCGCTGGGGCAACAGGCGGAGGAAGCTCTCCGGAAAAGGTTGCCTAACGCCATTCTGGGACAGGTATACTCTTCTTTACATTGTTATAAAAAGTGAATTTTAAGTCTAACTGAATCCCATACAATCCCATAAAATCGGTTGAATGAGATGAAGTTTGTATCTACATATAAAATGTTATAATCTCTATCTACACCATGCATTGATTCATTGTTCTTCCATCTTATCTCTCTCTTTCTCTGAATGAAGGGCTATGGAATGACAGAGGGTGGACCAGTTTTGACTATGTCTCTGGGCTTTGCAAAGTATCCATTCCCAACGAAATCTGGTTCATGCGGAACTGTGATCAGAAATGGTGAACTGAAGATCATTGACCCTCTAACATCCTTTTCTCTTCCCCGTAACCACTCTGGGGAGATTTGCGTCCGTGGGCCACAGATCATGAAAGGGTACCTGAATGACGAGGAGGCCACTGCAGCCGCCATTGATGGAGATGGTTGGCTTCACACAGGTGATATTGGGTACGTGGATGATGATGATGAGGTTTTCCTCGTTGACAGGGCCAAGGAACTCATCAAATTCAAAGGCTTTCAGGTACATGCATATATACTATAACATCTCTTCTTCTTCTTCCTTTTGTATTCACTCACCTGCTATTACTTTTACAGGTGCCGCCGGCAGAACTTGAAGACCTTCTCATGCGCCACCCTTCCATTGCAGATGCAGCTGTCGTCCCGTAAGTAACCATCTATCACAATTTCTATGCAATGATTTGAATTATTTACATATTAAATAATAAAAATATCTCTTACAATATTTTAGATTTTTAACTAAAACTAATCTTATACTTTAATTACCTTTTTTATCAGTTGTTATTGATGTATAATTATAAAAGTTTTGAAGTCGAACATTCTTCTTAAATATTCTTAAATGAGGAATCCATACGTTAACAAAGTGTTCGATTTAAAAAATATTAATTAAATTTTTAATAATTTTAATACGGATCTAATACCATTTTTAAAAAAATATATATACATTAATTTTTTTTAAAAATTAAATTGATCATATAAATTTTTATTATAATTTTAAAAATAAAGAACAAACTTTTTAAATGATAAACATTTTTATTATAAGTGTTTATTTTATAAGTTATAATTACTTTGTCCATTATAACTACTATATCATAATAGTATGAGTTATTTTTTATTTTTTTATCTGTTATATAAATATATTACTCAATAATTTAAGTATAATTAATAAAATATATTATTTTATTATTATTATTATAAATTTTTATTCTTTTTATAAATTAGATATATAAACTAATAATGCAATGTTGTTATTGGAACAATCCCTCCAAAATAACGTTACAGGCAGAATGACGATGCCGCCGGTGAAGTTCCGGTCGCTTTTGTCGTGAGCTTTGATCTTACAGAAGATGCTGTAAAGGATTTTATAGCTAAACAGGTAGAATTAGTAAGATTAATTATAAAATATTTAGAATTAAGAGAATGAGACGATGTGTTTGATGTGATCATAGGTGGTGTTTTACAAGAGACTGCACAAAGTGTATTTTGTTGACGAGATTCCAAAGTCTTCCACTGGAAAGATATTAAGAAAAGAACTGAGAGCAAAGGTGGGGAGCATTATTCAGAGGACCTAGCTACCACTCTTTTCCCATTCTTTTCCTTAATTTCTTCTTCCTTTGCTTTGCTGTTCTTCCTCAATGTGTAATCAAAGGATGCATATTCTCTTCAGAGATTGAAACTGCATCCAAAAGTATGTTTTCATGGTTCTACAAAGTTGTGGTCCCATACTTTCTAATTCAGAATTTGTGTTCCCATGGAATTCAGTAGTTGGTAGTGATAAATAAATGGATGCTGTTATTTATTGATTTCCCTAATTGATGGTAAGAATTTGGAATTTCAATTTTGCAGTGTTTCATCA

```
H) Nucleotide sequence comparison of the 4CL locus Phvul.011G084300 between two common bean genotypes


G19833 Phvul.011G084300          --------------------------------------------------
OAC Rex KF303293                 TGTGTGAGTAGACAAAGTTACATTGCACGACCTCTGACTTTGTCTGGTTT 50


G19833Phvul.011G084300           -----------------------------------TGTCATCTACTCTCA 15
OAC Rex KF303293                 AGGAAAAAAAACTTCATTCTACATCAGTATATCACTGTCATCTACTCTCA 100
                                                                    ***************

G19833 Phvul.011G084300          AGGATCTACTAAAACAGTAGCAAAACAACAATTAATGTCTCGTTATCATC 65
OAC Rex KF303293                 AGGATCTACTAAAACAGTAGCAAAACAACAATTAATGTCTCGTTATCATC 150
                                 **************************************************

G19833 Phvul.011G084300          TCCCACCTAATCCACGAATTCCGTGGAAGGAACTACTTGCTGTTCTTCTT 115
OAC Rex KF303293                 TCCCACCTAATCCACGAATTCCGTGGAAGGAACTACTTGCTGTTCTTCTT 200
                                 **************************************************

G19833 Phvul.011G084300          CTTCTTCTTCCCTTGTTCATCTGTGTAAAAATCTGCTCTATAAAATCCCC 165
Rex KF303293                     CTTCTTCTTCCCTTGTTCATCTGTGTAAAAATCTGCTCTATAAAATCCCC 250
                                 **************************************************
                                                               ATG                 EXON 1
G19833 Phvul.011G084300          TTCAATTCCTTCCCTCATCACATTAGAATTATGAATTCTGCAATCCATAG 215
OAC Rex KF303293                 TTCAATTCCTTCCCTCATCACATTAGAATTATGAATTCTGCAATGCATAG 300
                                 ******************************************** *****

G19833 Phvul.011G084300          ATCCGGGTACTGTAGCGATGGAACATACCGGTCTCAAAGGCCTTCCTTGG 265
OAC Rex KF303293                 ATCCGGGTACTGTAGCGATGGAACATACCGGTCTCAAAGGCCTTCGTTGG 350
                                 ********************************************* ****

G19833 Phvul.011G084300          TTCTCCCAGACAACCCCAACCTCTCACTCGTTTCGTTTCTCTTCCAAAGT 315
OAC Rex KF303293                 TTCTCCCAAAGAACCCCAACCTCTCACTCGTTTCGTTTCTCTTCCAAAGT 400
                                 ******** * ***************************************

G19833 Phvul.011G084300          GTTTCTTCTTTTCCATCCAAAACCGCTCTCGTAGACGCCGACTCTTCCCA 365
OAC Rex KF303293                 GTTTCTTCTTTTCCATCCAAAACCGCTCTCGTAGACGCCGACTCTTCCCA 450
                                 **************************************************

G19833 Phvul.011G084300          AACCCTCACCTTCGCGCAGCTAAAAGCCCGAGTCGCAACCCTCGCCCATG 415
OAC Rex KF303293                 AACCCTCACCTTCGCGCAGCTAAAAGCCCGAGTCGCAACCCTCGCCCATG 500
                                 **************************************************

G19833 Phvul.011G084300          GTTTCCTCAAACTAGGCATAAAGAAAAACGACGTCGTCCTCCTCCTCGCT 465
OAC Rex KF303293                 GTTTCCTCAAACTAGGCATAAAGAAAAACGACGTCGTCCTCCTCCTCGCT 550
                                 **************************************************

G19833 Phvul.011G084300          CCCAACACCATCCACTTCCCCGTCTGCTTCCTCGCCGCCACTGCCATCGG 515
OAC Rex KF303293                 CCCAACACCATCCACTTCCCCGTCTGCTTCCTCGCCGTCACTGCCATCGG 600
                                 ************************************* ************

G19833 Phvul.011G084300          AGCCATCGTCTCCACCGCCAACCCCATTTACACCGTCCACGAACTCTCCA 565
OAC Rex KF303293                 AGCCATCGTCTCCACCGCCAACCCCATTTACACCGTCCACGAACTCTCCA 650
                                 **************************************************

G19833 Phvul.011G084300          ACCAAGTCAAAGATTCCAACCCCAAGCTCGTCATCACTGTCCCTGAACTA 615
OAC Rex KF303293                 ACCAAGTCAAAGATTCCAACCCCAAGCTCGTCATCACTGTCCCTGAACTA 700
                                 **************************************************

G19833 Phvul.011G084300          TGCGACAAAGCAAAGAACCTCAACCTCCCCGCCGTCATTCTCAGCACCAA 665
OAC Rex KF303293                 TGGGACAAAGCAAAGAACCTCAACCTCCCCGCCGTCATTCTCAGCACCAA 750
```

** ***********************************************

```
                                                                                   INTRON 1
OAC Rex KF303293                 CAACCTTCAAAACCCAACTAAAAgtaagacttccaagacaccattgttta 800
G19833 Phvul.011G084300          CAACCTTCAAAACCCAACTAAAAgtaagacttccaagacaccattgttta 715
                                 **************************************************

G19833 Phvul.011G084300          tttattattattattagcaaaaataaataaaatcttctctgttggttcaa 765
OAC Rex KF303293                 tttattattattattagcaaaaataaataaaatcttctctgttggttcaa 850
                                 **************************************************

G19833 Phvul.011G084300          tttttttaaaatatgtacttttttaaattttttattgaa-ttaatgatta 814
OAC Rex KF303293                 ttttttaaaaatatgtacttttttaaattttttattgaaattaatgatta 900
                                 ****** ******************************** **********

G19833 Phvul.011G084300          aattaacttttttaagtatctaaatctggaaatatgacagtatgcaattt 864
OAC Rex KF303293                 aattaacttttttaagtatctaaatctggaaatatgacagtatgcaattt 950
                                 **************************************************

G19833 Phvul.011G084300          catttcaatatattttgctctgttttttgaaggaaaagaaatagatatca 914
OAC Rex KF303293                 catttcaatatattttgctctgtttttttaaggaaaagaaatagatatca 1000
                                 **************************** *********************

G19833 Phvul.011G084300          aagttgtataagaatttgtttctgaattcacacactattgaatccaccat 964
OAC Rex KF303293                 aagttgtataagaatttgtttctgaattcacacactattgaatccaccat 1050
                                 **************************************************
                                                                                   EXON 2
G19833 Phvul.011G084300          gcagACGTGTCAGGGAAAATCACGAGTCTGGAAGCGGTGATGGAGATGGC 1014
OAC Rex KF303293                 gcagACGTGTCAGGGAAAATCACGAGTCTGGAAGCGGTGATGGAGATGGC 1100
                                 **************************************************

G19833 Phvul.011G084300          GGGGCCGGTGACGGAGCTTCCGGAGAGCGGCGTTAAGCAGGGGGACACGG 1064
OAC Rex KF303293                 GGGGCCGGTGACGGAGCTTCCGGAGAGCGGCGTTAAGCAGGGGGACACGG 1150
                                 **************************************************

G19833 Phvul.011G084300          CGGCGCTGCTGTACTCCTCGGGGACCACGGGGCTGAGCAAAGGCGTGGTC 1114
OAC Rex KF303293                 CGGCGCTGCTGTACTCCTCTGGGACCACGGGGCTGAGCAAAGGCGTGGTC 1200
                                 ******************* ******************************

G19833 Phvul.011G084300          CTGACGCATGGGAACTTCATCGCGGCGTCGGTGATGATCAGCATGGACGA 1164
OAC Rex KF303293                 CTGACGCATGGGAACTTCATCGCGGCGTCGGTGATGATCAGCATGGACGA 1250
                                 **************************************************

G19833 Phvul.011G084300          CGACCTGGCCGGGGAGGTAAACGACGTGTTCCTCTGTGTTTTGCCGATGT 1214
OAC Rex KF303293                 CGACCTGGCCGGGGAGGTAAACGACGTGTTCCTCTGTGTTTTGCCGATGT 1300
                                 **************************************************

G19833 Phvul.011G084300          TCCACGTGTTCGGGCTGGCGGTTGTGACGTACACGGCGCTGCGGCGAGGG 1264
OAC Rex KF303293                 TCCACGTGTTCGGGCTGGCGGTTGTGACGTACACGGCGCTGCGGCGAGGG 1350
                                 **************************************************

G19833 Phvul.011G084300          AGCGCGGTGGTGTCGATGGGGAAGTTCGAGCTGGAGGCGTTTCTAAAAGC 1314
OAC Rex KF303293                 AGCGCGATGGTGTCGATGGGGAAGTTCGAGCTGGAGGCGTTTCTAAAAGC 1400
                                 ****** *******************************************

G19833 Phvul.011G084300          GATTGAGAAGCATAGAGTGACGAAGTTGTGGTTGGTGCCTCCGATCCTGC 1364
OAC Rex KF303293                 GATTGAGAAGCATAGAGTGACGAAGTTGTGGTTGGTGCCTCCGATCCTGC 1450
                                 **************************************************

G19833 Phvul.011G084300          TTGCTATGGCGAAACAGAGTGTGGTCCAGAGCTATGATCTGTCTTCGTTG 1414
OAC Rex KF303293                 TTGCTATGGCGAAACAGAGTGTGGTCCAGAGCTATGATCTGTCTTCGTTG 1500
                                 **************************************************

G19833 Phvul.011G084300          AAGCGGATAGGTTCCGGTGCCGCTCCTTTAGGGAAAGAGTTGATGCAAGA 1464
OAC Rex KF303293                 AAGCGGATAGGTTCCGGTGCTGCTCCTTTAGGGAAAGAGTTGATGCAAGA 1550
                                 ******************** *****************************
```

```
G19833 Phvul.011G084300          ATGTGGCAGGCGTTTTCCTCATGTAGCCATTTGTCAGGGCTATGGAATGA 1514
OAC Rex KF303293                 ATGTGGCAGGCGTTTTCCTCATGTAGCCATTTGTCAGGGCTATGGAATGA 1600
                                 **************************************************

G19833 Phvul.011G084300          CAGAGACTTGCGGCATTGTTTCTGTGGAGAATCCAAGAGCGGGGATTCGT 1564
OAC Rex KF303293                 CAGAAACTTGCGGCATTGTTTCTGTGGAGAATCCAAGAGCGGGGATTCGT 1650
                                 **** *********************************************

G19833 Phvul.011G084300          CATACTGGGTCGACGGGGATGCTGGCTTCGGGAGTAGAAGCTCAAATAGT 1614
OAC Rex KF303293                 CATACTGGGTCGACGGGGATGCTGGCTTCGGGAGTGGAAGCTCAAATAGT 1700
                                 *********************************** **************

G19833 Phvul.011G084300          GACTGTGGATACACAAAAGCCTCTCCCTCCTACACAGTCCGGGGAAATAT 1664
OAC Rex KF303293                 GACTGTGGATACACAAAAGCCTCTCCCTCCTACACAGTCCGGGGAAATAT 1750
                                 **************************************************
                                                                                   INTRON 2
G19833 Phvul.011G084300          GGGTGCGAGGTCCTAACATGATGAAAGgtaatgaaatttcatgtaccatt 1714
OAC Rex KF303293                 GGGTGCGAGGTCCTAACATGATGAAAGgtaatgaaatttcatgtaccatt 1800
                                 **************************************************

G19833 Phvul.011G084300          catgcttatccatcaaattcttaatctctaatatctagtttaaataattt 1764
OAC Rex KF303293                 catacttatccctcaaattcttaatctctaatatctagttt--------- 1841
                                 *** ******* *****************************

G19833 Phvul.011G084300          ttcatagtgtttctccttcatgcttttaaataattttgtaatcatatctc 1814
OAC Rex KF303293                 --------------------------------------------------


G19833 Phvul.011G084300          atgactagtttgtaaagggcaatataaactgtggtagcttgggttttatt 1864
OAC Rex KF303293                 -----------gtaaagggcaatataaactgtggtggcttgggttttatt 1880
                                            ************************ **************

G19833 Phvul.011G084300          ctggtattgaaccttttactctggctttctaattgaagactcagattcct 1914
OAC Rex KF303293                 ctggtattgaaccttttactctggctttctaattgaagactcagattcct 1930
                                 **************************************************

G19833 Phvul.011G084300          ctttctgtcaatgttttctgctgggatagatgtggaggattaacattttt 1964
OAC Rex KF303293                 ctttctgtcaatgttttctgctgggatagatgtggaggattaacatattt 1980
                                 ********************************************** ***

G19833 Phvul.011G084300          tttactttatatggagttttgacattaatatgttgtgtagtcttttccta 2014
OAC Rex KF303293                 tt-actttatatggagttt-gacatcaatttgttgtgtagtcttttccta 2028
                                 ** **************** ***** *** ********************
                                                                                   EXON 3
G19833 Phvul.011G084300          tataaaacttagtgcaaccattaaaaaactttcagGTTATCACAACAATC 2064
OAC Rex KF303293                 tataaaacttagtgcaaccattaaaaaa-tttcagGTTATCACAACAATC 2077
                                 **************************** *********************

G19833 Phvul.011G084300          CACAGGCCACGAGATTGACAATAGATGAAAAGGGGTGGGTGCATACAGGA 2114
OAC Rex KF303293                 CACAGGCCACGAGATTGACAATAGATGAAAAGGGGTGGGTGCATACAGGA 2127
                                 **************************************************

G19833 Phvul.011G084300          GATATTGGATATTTTGATGAAGATGGACAACTTTATGTGGTAGACCGTAT 2164
OAC Rex KF303293                 GATATTGGATATTTTGATGAAGATGGACAACTTTATGTGGTTGACCGTAT 2177
                                 ***************************************** ********
                                                                                   INTRON 3
G19833 Phvul.011G084300          CAAAGAACTAATCAAGTACAAAGGTTTTCAGgtatgttatgtagtgcaaa 2214
OAC Rex KF303293                 CAAAGAACTAATCAAGTACAAAGGTTTTCAGgtatgttatgtagtgcaaa 2227
                                 **************************************************

G19833 Phvul.011G084300          gaaagtgaatttttctggtatgaagtgaattgcatgattatttatctgaa 2264
OAC Rex KF303293                 gaaagtgaatttttctggtatgaagtgaattgcatgattatatatctgaa 2277
                                 ***************************************** ********
```

```
G19833 Phvul.011G084300          atcttaaccaaatccttgtgggtctagtggggtgttgaagtacattaaac 2314
OAC Rex KF303293                 atcttaaccaaatccttgtgggtctagtggggtgttgaaggacattaaac 2327
                                 **************************************** *********

G19833 Phvul.011G084300          agatgttccatgtccaaagtggttaatgtgtgaattgtagtaatttctgt 2364
OAC Rex KF303293                 agatgttccatgtccaaagtggttaatgtgtgaattgtagtaatttctgt 2377
                                 **************************************************

G19833 Phvul.011G084300          ttgggtaattatttgtccatatcatgacccttctgctcattggagacaat 2414
OAC Rex KF303293                 ttgggtaattattcgtccatatcataacccttctgctcattggagacaat 2427
                                 ************* *********** ************************
                                                                                   EXON 4
G10933 Phvul.011G084300          atcttactgaactatgagctgtttaacaagGTTGCACCAGCTGAACTTGA 2464
OAC Rex KF303293                 atcttactgaactatgagctgtttaacaagGTTGCACCAGCTGAACTTGA 2477
                                 **************************************************
                                                                                   INTRON 4
G19833 Phvul.011G084300          AGGCCTTCTCATTTCTCACCCTGAAATACTAGACGCTGTTGTTGTTCCgt 2514
OAC Rex KF303293                 AGGCCTTCTCATTTCTCACCCTGAAATACTAGACGCTGTTGTTGTTCCgt 2527
                                 **************************************************

G19833 Phvul.011G084300          aagtgtcatataagtttgattttagtcatcatatgttatcgattattagg 2564
OAC Rex KF303293                 aagtgtcatataagtttgattttagtcatcatatgttatcgattattagg 2577
                                 **************************************************
                                                                                   EXON 5
G19833 Phvul.011G084300          aattcacacttgagtaactttctttcaatagATATCCCGATGATGAGGCT 2614
OAC Rex KF303293                 aattcacacttgagtaactttctttcaatagATATCCCGATGATGAGGCT 2627
                                 **************************************************

G19833 Phvul.011G084300          GGTGAGGTTCCAATTGCCTATGTTGTTCGCTCACCCAATAGTTCGCTGAC 2664
OAC Rex KF303293                 GGTGAGGTTCCAATTGCCTATGTTGTTCGCTCACCCAATAGTTCGCTGAC 2677
                                 **************************************************
                                                                                   INTRON 5
G19833 Phvul.011G084300          TGAAGAAGAAATTAAGAAGTTTATTGCTAATCAGgtaagagaaagataca 2714
OAC Rex KF303293                 TGAAGAAGAAATTAAGAAGTTTATTGCTAATCAGgtaagagaaagataca 2727
                                 **************************************************

G19833 Phvul.011G084300          aaaactagtttgattttttactgtcactcataacttaaatagattgtctt 2764
OAC Rex KF303293                 aaaactagtttgattttttactgtcactcataacttaaatagattgtctt 2777
                                 **************************************************

G19833 Phvul.011G084300          gccaaagtcaaaatacagtgaatttttgtcaagaaaaagtaaaaggaagt 2814
OAC Rex KF303293                 gccaaagtcaaaatacagtgaatttttgtcaagaaaaagtaaaaggaagt 2827
                                 **************************************************

G19833 Phvul.011G084300          gcatgctttcaagaaaattgcttctttcatcctttagttctgtgtttgaa 2864
OAC Rex KF303293                 gcatgctttcaagaaaattgcttctttcatcctttagttctgtgtttgaa 2877
                                 **************************************************

G19833 Phvul.011G084300          ctttgtcttgtaataaaatttgcaataattatagtatgaatccaacttat 2914
OAC Rex KF303293                 ctttgtcttgtaataaaatttgcaataattatagtatgaatccaacttat 2927
                                 **************************************************

G19833 Phvul.011G084300          ctctagtggatgattctgaatcaaagtttaacccataaatgaagcacttg 2964
OAC Rex KF303293                 ctctagtggatgattctgaatcaaagtttaacccataaatgaagcacttg 2977
                                 **************************************************

G19833 Phvul.011G084300          ccattgttttcacagattggtcaactaatcacatctgaatgtttgctctc 3014
OAC Rex KF303293                 ccattgttttcacagattggtcaactaatcacatctgaatgtttgctctc 3027
                                 **************************************************
                                                                                   EXON 6
G19833 Phvul.011G084300          tgcatattttgtagttactcatttttatactatttacagGTTGCACCGTT 3064
OAC Rex KF303293                 tgcatattttgtagttactcatttttatactatttacagGTTGCACCGTT 3077
                                 **************************************************
```

```
G19833 Phvul.011G084300          CAAAAAATTGAGAAGGGTGACATTCATAAGTAGTGTTCCAAGGACATCTT 3114
OAC Rex KF303293                 CAAAAAATTGAGAAGGGTGACATTCATAAGTAGTGTTCCAAGGACATCTT 3127
                                 **************************************************

G19833 Phvul.011G084300          CAGGAAAAATTCTTAGAAGAGAGATCACTGCGAAAGCACGATCCAAAATA 3164
OAC Rex KF303293                 CAGGAAAAATTCTTAGAAGAGAGATCACTGCGAAAGCACGATCCAAAATA 3177
                                 **************************************************
                                 TAA
G19833 Phvul.011G084300          TAATATATTACAATTCTTTTGGAGGGTCAATTCAATTTCTGAGAAAGAGT 3214
OAC Rex KF303293                 TAATATATTACAATTCTTTTGGAGGGTCAATTCAATTTCTGAGAAAGAGT 3227
                                 **************************************************

G19833 Phvul.011G084300          GAAAATTCTCAGTGCATCTTGAAGCATTAGTAGATGTGCCCAATCAAGTT 3264
OAC Rex KF303293                 GAAAATTCTCAGTGCATCTTGAAGCATTAGTAGATGTGCCCAATCAAGTT 3277
                                 **************************************************

G19833 Phvul.011G084300          TAAAATTAAAACCACACAACAGTTCCTACATAGTAGCATTTCTTCTTCGC 3314
OAC Rex KF303293                 TAAAATTAAAACCACACAACAGTTACTACATAGTAGCATTTCTTCTTCGC 3327
                                 ************************ *************************

G19833 Phvul.011G084300          ACATAAATCTTGTGAATTTTATTACAGTAAATATTTACTCCATTTAACCC 3364
OAC Rex KF303293                 ACATAAATCTTGTGAATTTTATTACAGTAAATATTTACTCCATTTAACCC 3377
                                 **************************************************

G19833 Phvul.011G084300          AAGGAAAGCAAGAAAGTGAATTTGGTGTTTGTGTACTAAATTTAACTCTT 3414
OAC Rex KF303293                 AAGGAAAGCAAGAAAGTGAATTTGGTGTTTGTGTACTAAATTTAACTCTT 3427
                                 **************************************************

G19833 Phvul.011G084300          CAAGAGTTAGAAAAATTAATGAGTACATACTTTCTAAGT----------- 3453
OAC Rex KF303293                 CAAGAGTTAGAAAAATTAATGAGTACATACTTTCTAAGTATTCTAAGAGT 3477
                                 ***************************************


OAC Rex sequence:
>KF303293 OAC Rex gene for 4-coumarate:CoA ligase (4CL-8; 3,477 bp); similar to P. vulgaris 4CL locus Phvul.011G084300 (cultivar G19833)
TGTGTGAGTAGACAAAGTTACATTGCACGACCTCTGACTTTGTCTGGTTTAGGAAAAAAAACTTCATTCTACATCAGTATATCACTGTCATCTACTCTCAAGGATCTACTAAAACAGTAGCAAAACAACAATTAATGTCTCGTTATCATCTCCCACCTAATCCACGAATTCCGTGGAAGGAACTACTTGCTGTTCTTCTTCTTCTTCTTCCCTTGTTCATCTGTGTAAAAATCTGCTCTATAAAATCCCCTTCAATTCCTTCCCTCATCACATTAGAATTATGAATTCTGCAATGCATAGATCCGGGTACTGTAGCGATGGAACATACCGGTCTCAAAGGCCTTCGTTGGTTCTCCCAAAGAACCCCAACCTCTCACTCGTTTCGTTTCTCTTCCAAAGTGTTTCTTCTTTTCCATCCAAAACCGCTCTCGTAGACGCCGACTCTTCCCAAACCCTCACCTTCGCGCAGCTAAAAGCCCGAGTCGCAACCCTCGCCCATGGTTTCCTCAAACTAGGCATAAAGAAAAACGACGTCGTCCTCCTCCTCGCTCCCAACACCATCCACTTCCCCGTCTGCTTCCTCGCCGTCACTGCCATCGGAGCCATCGTCTCCACCGCCAACCCCATTTACACCGTCCACGAACTCTCCAACCAAGTCAAAGATTCCAACCCCAAGCTCGTCATCACTGTCCCTGAACTATGGGACAAAGCAAAGAACCTCAACCTCCCCGCCGTCATTCTCAGCACCAACAACCTTCAAAACCCAACTAAAAGTAAGACTTCCAAGACACCATTGTTTATTTATTATTATTATTAGCAAAAATAAATAAAATCTTCTCTGTTGGTTCAATTTTTTAAAAATATGTACTTTTTTAAATTTTTTATTGAAATTAATGATTAAATTAACTTTTTTAAGTATCTAAATCTGGAAATATGACAGTATGCAATTTCATTTCAATATATTTTGCTCTGTTTTTTTAAGGAAAAGAAATAGATATCAAAGTTGTATAAGAATTTGTTTCTGAATTCACACACTATTGAATCCACCATGCAGACGTGTCAGGGAAAATCACGAGTCTGGAAGCGGTGATGGAGATGGCGGGGCCGGTGACGGAGCTTCCGGAGAGCGGCGTTAAGCAGGGGGACACGGCGGCGCTGCTGTACTCCTCTGGGACCACGGGGCTGAGCAAAGGCGTGGTCCTGACGCATGGGAACTTCATCGCGGCGTCGGTGATGATCAGCATGGACGACGACCTGGCCGGGGAGGTAAACGACGTGTTCCTCTGTGTTTTGCCGATGTTCCACGTGTTCGGGCTGGCGGTTGTGACGTACACGGCGCTGCGGCGAGGGAGCGCGATGGTGTCGATGGGGAAGTTCGAGCTGGAGGCGTTTCTAAAAGCGATTGAGAAGCATAGAGTGACGAAGTTGTGGTTGGTGCCTCCGATCCTGCTTGCTATGGCGAAACAGAGTGTGGTCCAGAGCTATGATCTGTCTTCGTTGAAGCGGATAGGTTCCGGTGCTGCTCCTTTAGGGAAAGAGTTGATGCAAGAATGTGGCAGGCGTTTTCCTCATGTAGCCATTTGTCAGGGCTATGGAATGACAGAAACTTGCGGCATTGTTTCTGTGGAGAATCCAAGAGCGGGGATTCGTCATACTGGGTCGACGGGGATGCTGGCTTCGGGAGTGGAAGCTCAAATAGTGACTGTGGATACACAAAAGCCTCTCCCTCCTACACAGTCCGGGGAAATATGGGTGCGAGGTCCTAACATGATGAAAGGTAATGAAATTTCATGTACCATTCATACTTATCCCTCAAATTCTTAATCTCTAATATCTAGTTTGTAAAGGGCAATATAAACTGTGGTGGCTTGGGTTTTATTCTGGTATTGAACCTTTTACTCTGGCTTTCTAATTGAAGACTCAGATTCCTCTTTCTGTCAATGTTTTCTGCTGGGATAGATGTGGAGGATTAACATATTTTTACTTTATATGGAGTTTGACATCAATTTGTTGTGTAGTCTTTTCCTATATAAAACTTAGTGCAACCATTAAAAAATTTCAGGTTATCACAACAATCCACAGGCCACGAGATTGACAATAGATGAAAAGGGGTGGGTGCATACAGGAGATATTGGATATTTTGATGAAGATGGACAACTTTATGTGGTTGACCGTATCAAAGAACTAATCAAGTACAAAGGTTTTCAGGTATGTTATGTAGTGCAAAGAAAGTGAATTTTTCTGGTATGAAGTGAATTGCATGATTATATATCTGAAATCTTAACCAAATCCTTGTGGGTCTAGTGGGGTGTTGAAGGACATTAAACAGATGTTCCATGTCCAAAGTGGTTAATGTGTGAATTGTAGTAATTTCTGTTTGGGTAATTATTCGTCCATATCATAACCCTTCTGCTCATTGGAGACAATATCTTACTGAACTATGAGCTGTTTAACAAGGTTGCACCAGCTGAACTTGAAGGCCTTCTCATTTCTCACCCTGAAATACTAGACGCTGTTGTTGTTCCGTAAGTGTCATATAAGTTTGATTTTAGTCATCATATGTTATCGATTATTAGGAATTCACACTTGAGTAACTTTCTTTCAATAGATATCCCGATGATGAGGCTGGTGAGGTTCCAATTGCCTATGTTGTTCGCTCACCCAATAGTTCGCTGACTGAAGAAGAAATTAAGAAGTTTATTGCTAATCAGGTAAGAGAAAGATACAAAAACTAGTTTGATTTTTTACTGTCACTCATAACTTAAATAGATTGTCTTGCCAAAGTCAAAATACAGTGAATTTTTGTCAAGAAAAAGTAAAAGGAAGTGCATGCTTTCAAGAAAATTGCTTCTTTCATCCTTTAGTTCTGTGTTTGAACTTTGTCTTGTAATAAAATTTGCAATAATTATAGTATGAATCCAACTTATCTCTAGTGGATGATTCTGAATCAAAGTTTAACCCATAAATGAAGCACTTGCCATTGTTTTCACAGATTGGTCAACTAATCACATCTGAATGTTTGCTCTCTGCATATTTTGTAGTTACTCATTTTTATACTATTTACAGGTTGCACCGTTCAAAAAATTGAGAAGGGTGACATTCATAAGTAGTGTTCCAAGGACATCTTCAGGAAAAATTCTTAGAAGAGAGATCACTGCGAAAGCACGATCCAAAATATAATATATTACAATTCTTTTGGAGGGTCAATTCAATTTCTGAGAAAGAGTGAAAATTCTCAGTGCATCTTGAAGCATTAGTAGATGTGCCCAATCAAGTTTAAAATTAAAACCACACAACAGTTACTACATAGTAGCATTTCTTCTTCGCACATAAATCTTGTGAATTTTATTACAGTAAATATTTACTCCATTTAACCCAAGGAAAGCAAGAAAGTGAATTTGGTGTTTGTGTACTAAATTTAACTCTTCAAGAGTTAGAAAAATTAATGAGTACATACTTTCTAAGTATTCTAAGAGT
```
