# Supplementary material for: In silico comparison of genomic regions containing genes coding for enzymes and transcription factors for the phenylpropanoid pathway in Phaseolus vulgaris L. and Glycine max L. Merr
Source: Front Plant Sci. 2013 Sep 5;4:317. doi: 10.3389/fpls.2013.00317 (PMC3763686; doi:10.3389/fpls.2013.00317)
Supplement: Figure S1 — Phylogenetic relationships among common bean and soybean 4CL homologous proteins. Trees were generated from 4CL protein sequence analyses in CLC Genomics Workbench 3 using Neigbor Joining (NJ) algorithm and 1000 bootstrap replications. Scale represents genetic distance and node numbers indicate bootstrap support values. (A) Primary transcripts (98 protein sequences); (B) Twenty-one 4CL proteins selected based on similarity to soybean 4CL1 (red), 4CL2 (blue) and 4CL3/4CL4 (green). [file DataSheet1.ZIP › Supplementary Material/Supplemental Figure S3 - 4CL protein sequence comparison.doc]

**Supplementary Figure S3 | Amino acid sequence comparison of 4CL proteins between Andean common bean landrace G19833 (reference) and Mesoamerican cultivar OAC Rex**

```
G19833 Phvul.005G131400         M------------------QRSGYGSDGIFRSLRPSIALPKQPNLTLVSH 32
OAC Rex KF303289                M------------------QRSGYGSDGIFRSLRPSIVLPKQPNLTLVSH 32
G19833 Phvul.011G084300         M--------------NSAIHRSGYCSDGTYRSQRPSLVLPDNPNLSLVSF 36
OAC Rex KF303293                M--------------NSAMHRSGYCSDGTYRSQRPSLVLPKNPNLSLVSF 36
G19833 Phvul.002G040100         M-LSVAPSLDTN---TKTDQTQIPQTTHVFKSKLPDIPISN--HLSLHDY 44
OAC Rex KF303286                M-LSVAPSLDTNTKDTKTDQTQIPQTTHVFKSKLPDIPISN--HLSLHDY 47
G19833 Phvul.011G020200         M--------------SERAEPETPQT-HIFRSKLPDIPLPN--NLLLHTY 33
OAC Rex KF303292                M--------------SERAEPETPQT-HIFRSKLPDIPLPN--NLLLHTY 33
G19833 Phvul.005G184500         M-----------------AVETQQGKDFIFRSKLPDIYIPK--HLPLHAY 31
OAC Rex KF303290                M-----------------AVETQQGKDFIFRSKLPDIYIPK--HLPLHAY 31
G19833 Phvul.003G146900         M--------------EQQQSTQLHNDDFIFRSKLPDIYIPT--HLPLHTY 34
OAC Rex KF303287                M--------------EQQQSTQLHNDDFIFRSKLPDIYIPT--HLPLHTY 34
G19833 Phvul.003G147000         M---------PPTTPPPPPTMAPSPQEIIFRSPLPDIPIPT--HLPLHSY 39
OAC Rex KF303288                M---------PPTTPPPPPTMAPSPQEIIFRSPLPDIPIPT--HLPLHSY 39
G19833 Phvul.006G057300         MQHLVNCLSTHNTNMGTYVESLSGVEEHVFRSQYSPVPIPD--NVTLPEY 48
OAC Rex KF303291                MQHLVNCLSTHNTNMGTYVESLSGVEEHVFRSQYSPVPIPD--NVTLPEY 48
                                                             ::*  . : :.   :: *  .


G19833 Phvul.005G131400         LFNRVAPFPSKHALIDADSSQTLSFAQLKSLTVKVSHGLLRLGVAKNDVV 82
OAC Rex KF303289                LFNRVAPFPSKHALIDADSSQTLSFAQLKSLTVKVSHGLLRLGVAKNDVV 82
G19833 Phvul.011G084300         LFQSVSSFPSKTALVDADSSQTLTFAQLKARVATLAHGFLKLGIKKNDVV 86
OAC Rex KF303293                LFQSVSSFPSKTALVDADSSQTLTFAQLKARVATLAHGFLKLGIKKNDVV 86
G19833 Phvul.002G040100         CFQNLSQFADRPCLIVGSASKTYTYAQTYLISRKIAAGFSNLGIRKGDVV 94
OAC Rex KF303286                CFQNLSQFADCPCLIVGSASKTYTYAQTYLISRKIAAGFSNLGIRKGDVV 97
G19833 Phvul.011G020200         CFLKLPQVSHRTCLIA--AARTYTYAETHSASRKVAAGMSKLGIQKGDAV 81
OAC Rex KF303292                CFLKLPQVSHRTCLIA--AARTYTYAETHSASRKVAAGMSKLGIQKGDAV 81
G19833 Phvul.005G184500         CFQNLPDHASRPCLINAPTGEVYTYSDVDATARKVAAGLHKLGVRQGEVI 81
OAC Rex KF303290                CFQNLPDHASRPCLINAPTGEVFTYSDVDATARKVAAGLHKLGVRQGEVI 81
G19833 Phvul.003G146900         IFQNISQFSHRPCLINAATGDCFTYAEVQLTSRRVAAGLNKLGIEKGDVI 84
OAC Rex KF303287                IFQNISQFSHRPCLINAATGDCFTYAEVQLTSRRVAAGLNKLDIQKGDVI 84
G19833 Phvul.003G147000         CFQNLSQFQHRTCLIDGETGETLTYAEVDLAAHRIAAGLHNIGIRQGDVI 89
OAC Rex KF303288                CFQNLSQFQHRTCLIDGETGETLTYAEVDLAARRIAAGLHNIGIRQGDVI 89
G19833 Phvul.006G057300         VLQNAELYGEKVAFVDAVTGKGVSYREVVRDVDRFSKALRSLGLRKGHVV 98
OAC Rex KF303291                VLQNAELYGEKVAFVDAVTGKGVSYREVVRDVDRFSKALRSLGLRKGHVV 98
                                 :          .::   :.   :: :       .: .:  :.: :...:


G19833 Phvul.005G131400         LFLAPNDIRYIVCFLAVTSLGAIVTTVNPAYTAAEVAKQAKDSNPKFVVT 132
OAC Rex KF303289                LFLAPNDIRYIVCFLAVTSLGAIVTTVNPAYTAAEVAKQAKDSNPKFVVT 132
G19833 Phvul.011G084300         LLLAPNTIHFPVCFLAATAIGAIVSTANPIYTVHELSNQVKDSNPKLVIT 136
OAC Rex KF303293                LLLAPNTIHFPVCFLAVTAIGAIVSTANPIYTVHELSNQVKDSNPKLVIT 136
G19833 Phvul.002G040100         MILLQNSAEFVLSFLAASMVGAVATTANPFYTAAEIFKQFTASKAKLIIT 144
OAC Rex KF303286                MILLQNSAEFVLSFLAASMVGAVATTANPFYTAAEIFKQFTASKAKLIIT 147
G19833 Phvul.011G020200         MILLPNSPEFVFSFMAASMLGAVATTANPSYTAAEIAKQLAASNAKLVVT 131
OAC Rex KF303292                MILLPNSPEFVFSFMAASMLGAVATTANPSYTAAEIAKQLAASNAKLVVT 131
G19833 Phvul.005G184500         MILLPNCPEFVFAFLGASHRGAMATAANPFFTAAEIAKQAKASNAKVLIT 131
OAC Rex KF303290                MILLPNCPEFVFAFLGASHRGAMATAANPFFTAAEIAKQAKASNAKVLIT 131
G19833 Phvul.003G146900         LLLLQNCPQFVFAFLGSSFCGATVTTANPFYTPAEVAKQAAASNAKLIIT 134
OAC Rex KF303287                LLLLQNCPQFVFAFLGSSFCGATVTTANPFYTPAEVAKQAAASNAKLIIT 134
G19833 Phvul.003G147000         MLVLRNCPQFALAFLGASHRGAVITTANPFYTPLELAKQAAATKTRLVIT 139
OAC Rex KF303288                MLVLRNCPQFALAFLGASHRGAVITTANPFYTPLELAKQAAATKTRLVIT 139
G19833 Phvul.006G057300         IVVLPNVVEYAIVALGIMAAGGVFSGANPSSHVSEIKKQAESADAKLIVT 148
OAC Rex KF303291                IVVLPNVVEYAIVALGIMAAGGVFSGANPSSHVSEIKKQAESADAKLIVT 148
```

:.: * .: . :. *. : .**	*: :*	:..:.::*

```
G19833 Phvul.005G131400         VPELWDKLQHLNLP---AVFLHSRAHSN--------ATSFDALVHLAGSP 171
OAC Rex KF303289                VPELWDKLQHLNLP---AVFLHSRAHSN--------ATSFDALVHLAGSP 171
G19833 Phvul.011G084300         VPELCDKAKNLNLP---AVILSTNNLQNPTKNVSGKITSLEAVMEMAGPV 183
OAC Rex KF303293                VPELWDKAKNLNLP---AVILSTNNLQNPTKNVSGKITSLEAVMEMAGPV 183
G19833 Phvul.002G040100         QAMYVEKLRSHDAGWSLGEDFKVVTVDDPPE------NCLHFSVLSEANE 188
OAC Rex KF303286                QAMYVEKLRSHDAGWSLGEDFKVVTVDDPPE------NCLHFSVLSEANE 191
G19833 Phvul.011G020200         LSENVPKLHQQER----QHFFKVVTVDDPPE------NCTRFP---EGDE 168
OAC Rex KF303292                LSENVPKLHQQER----QHFFKVVTVDDPPE------NCTRFP---EGDE 168
G19833 Phvul.005G184500         QASYYEKVKELEE----VKLVFVDSCPAAEAD---GGGHIHFSELCEGGG 174
OAC Rex KF303290                QASYYEKVKELEE----VKLVFVDSCPAAEAY---GGGHIHFSELCEGGG 174
G19833 Phvul.003G146900         QASYVDKVKDYAR----EKDVRVMCVDAAPE------GYLAFSDLTEANE 174
OAC Rex KF303287                QASYVDKVKDYAR----EKDVRVMCVDTAPE------GCKK--------- 165
G19833 Phvul.003G147000         QSAYVDKIKSFAD----NNDVMVMCIDSEDD------GVLHFSTLTNADE 179
OAC Rex KF303288                QSAYVDKIKSFAD----NNDVMVMCIDSEDD------GVLHFSTLTNADE 179
G19833 Phvul.006G057300         NSSNYEKVKGLELP---VIVLGDEVVEGGMN-------WNKLLEAADRAG 188
OAC Rex KF303291                NNSNYEKVKGLELP---VIVLGDEVVEGGMN-------WNKLLEAADRAG 188
                                      * :           .

        Acyl-activating enzyme consensus motif #  ###### ##
                                                  SSGTTGLPKGV                      BOX I
G19833 Phvul.005G131400         TEFPKVDVKQSDTAVLLYSSGTTGLSKGVVLTHGNFVA--ASLMIGLDDD 219
OAC Rex KF303289                TEFPKVDVKQSDTAVLLYSSGTTGLSKGVVLTHGNFVA--ASLMIGLDDD 219
G19833 Phvul.011G084300         TELPESGVKQGDTAALLYSSGTTGLSKGVVLTHGNFIA--ASVMISMDDD 231
OAC Rex KF303293                TELPESGVKQGDTAALLYSSGTTGLSKGVVLTHGNFIA--ASVMISMDDD 231
G19833 Phvul.002G040100         SDAPEVEIHPDDAVALPFSSGTTGLPKGVILTHKSLTTSVAQQVDGENPN 238
OAC Rex KF303286                SDAPEVEIHPDDAVALPFSSGTTGLPKGVILTHKSLTTSVAQQVDGENPN 241
G19833 Phvul.011G020200         SGVPEVEISAEDAVALPFSSGTTGLAKGVILTHKSLVTSVAQQMEGENPN 218
OAC Rex KF303292                SGVPEVEISAEDAVALPFSSGTTGLAKGVILTHKSLVTSVAQQMEGENPN 218
G19833 Phvul.005G184500         -EMDEVDIRADDVVALPYSSGTTGLPKGVMLTHKGLVTSIAQQVDGENPN 223
OAC Rex KF303290                -EMDEVDIRADDVVALPYSSGTTGLPKGVMLTHKGLVTSIAQQVDGENPN 223
G19833 Phvul.003G146900         RDIPGVKISPDDVVALPYSSGTTGLPKGVMLTHKGMVTSVAQQVDGENPN 224
OAC Rex KF303287                -----------------------------------MVTSVAQQVDGENPN 15
G19833 Phvul.003G147000         TEAPAVKISPDDVVALPFSSGTSGLPKGVMLSHKNLVTTIAQLVDGENAH 229
OAC Rex KF303288                TEAPAVKISPDDVVALPFSSGTSGLPKGVMLSHKNLVTTIAQLVDGENAH 229
G19833 Phvul.006G057300         DDFAKEPIHQNDLCAMPFSSGTTGMSKGVMLTHRNLVANLCSTLFTVTKE 238
OAC Rex KF303291                DDFAKEPIHQNDLCAMPFSSGTTGMSKGVMLTHRNLVANLCSTLFTVTKE 238

                                Active site *    **   *
                               AMP binding site &
                  Putative CoA binding site |
G19833 Phvul.005G131400         LAGEMHSVFLCVLPMFHVFGLMVISYSQLQRGSAVVSLKKFEFELVLKTI 269
OAC Rex KF303289                LAGEMHSVFLCVLPMFHVFGLMVISYSQLQRGSAVVSLKKFEFELVLKTI 269
G19833 Phvul.011G084300         LAGEVNDVFLCVLPMFHVFGLAVVTYTALRRGSAVVSMGKFELEAFLKAI 281
OAC Rex KF303293                LAGEVNDVFLCVLPMFHVFGLAVVTYTALRRGSAMVSMGKFELEAFLKAI 281
G19833 Phvul.002G040100         LYLTTEDVLLCVLPLFHIFSLNSVLLCALRAGSGVLLMQKFEIGTLLELI 288
OAC Rex KF303286                LYLTTEDVLLCVLPLFHIFSLNSVLLCALRAGSGVLLMQKFEIGTLLELI 291
G19833 Phvul.011G020200         MYLKEEDVVLCVLPLFHIFSMHIAMMCALRAGSAILLIEKFEMRKLLEAI 268
OAC Rex KF303292                MYLKEEDVVLCVLPLFHIFSMHIAMMCALRAGSAILLIEKFEMRKLLEAI 268
G19833 Phvul.005G184500         LYYHSDDTILCVLPLFHIYSLNSVLLCGLRSKAAILLMPKFEIKALLALI 273
OAC Rex KF303290                LYYHSDDTILCVLPLFHIYSLNSVLLCGLRSKAAILLMPKFEIKALLALI 273
G19833 Phvul.003G146900         LYLRSEDVVLCVLPLFHIYSLNTVLLCSLRVGAAVLVMPKFEIASLLELV 274
OAC Rex KF303287                LYLRSEDVVLCVLPLFHIYSLNTVLLCSLRVGAAVLVMPKFEIASLLELV 65
G19833 Phvul.003G147000         QYTHSEDVLLCVLPMFHIYALNSILLSGIRSGAAVLIVQKFEMTSLLELI 279
OAC Rex KF303288                QYTHSEDVLLCVLPMFHIYALNSILLSGIRSGAAVLIVQKFEMTSLLELI 279
G19833 Phvul.006G057300         MEGLVT--TLGLIPFFHIYGITGICCATLRSKGKVVVMGRFELKKFLNAL 286
```

OAC Rex KF303291 MEGLVT--TLGL**I**PFF**HIY**GIT**G**ICCATLRSKGKVVVMGRFELKKFLNAL 286

```
                                            **  *                    * ****
                                                                        &&&
                                            ||  |                      |
G19833 Phvul.005G131400         EKFSVTHLWVVPPIILALAKHGLVDKYDLS--SLKHIGSGAAPLGKELME 317
OAC Rex KF303289                EKFSVTHLWVVPPIILALAKHGLVDKYDLS--SLKHIGSGAAPLGKELME 317
G19833 Phvul.011G084300         EKHRVTKLWLVPPILLAMAKQSVVQSYDLS--SLKRIGSGAAPLGKELMQ 329
OAC Rex KF303293                EKHRVTKLWLVPPILLAMAKQSVVQSYDLS--SLKRIGSGAAPLGKELMQ 329
G19833 Phvul.002G040100         QRYRVSVAMVVPPLVLALAKNPMVANFDLS--SIRLVLSGAAPLGKELEE 336
OAC Rex KF303286                QRYRVSVAMVVPPLVLALAKNPMVANFDLS--SIRLVLSGAAPLGKELEE 339
G19833 Phvul.011G020200         ERQRVTVAIVVPPLVAALTKNSTVEEYDLR--SIRLVISGAAPLGQQAEE 316
OAC Rex KF303292                ERQRVTVAIVVPPLVAALTKNSTVEEYDLR--SIRLVISGAAPLGQQAEE 316
G19833 Phvul.005G184500         HKHRVTVAAVVPPIVLAIARSPDLASYDLS--SIRLFKSGGAPLGKDLED 321
OAC Rex KF303290                HKHRVTVAAVVPPIVLAIARSPDLASYDLS--SIRLFKSGGAPLGKDLED 321
G19833 Phvul.003G146900         EKYKVSVAPFVPPIILAIAKTPDLERYDLS--SIRMILSGAAPVGKELED 322
OAC Rex KF303287                EKHKVSVAPFVPPIILAIAKTPDLERYDLS--SIRMILSGAAPVGKELED 113
G19833 Phvul.003G147000         QKYRVTVASFVPPIVLALVKSGEAHRYDLS--SVRAVVTGAAPLGRELQE 327
OAC Rex KF303288                QKYRVTVASFVPPIVLALVKSGEAHRYDLS--SVRAVVTGAAPLGKELQE 327
G19833 Phvul.006G057300         ITHEVTFAPIVPPIILSLVKNPIVDEFDLSKLKLQAIMTAAAPLAPELLN 336
OAC Rex KF303291                ITHEVTFAPIVPPIILSLVKNPIVDEFDLSKLKLQAIMTAAAPLAPELLN 336


                                            ******   **
                                             &&&&&
G19833 Phvul.005G131400         ECAKRFPRATVAQGYGMTETCGIVSVENTRLG---IRHSGSAGMLVAGVE 364
OAC Rex KF303289                ECAKRFPRATVAQGYGMTETCGIVSVENTRMG---IRHSGSAGMLVAGVE 364
G19833 Phvul.011G084300         ECGRRFPHVAICQGYGMTETCGIVSVENPRAG---IRHTGSTGMLASGVE 376
OAC Rex KF303293                ECGRRFPHVAICQGYGMTETCGIVSVENPRAG---IRHTGSTGMLASGVE 376
G19833 Phvul.002G040100         ALHSRVPHAVLGQGYGMTEAGPVLSMCLGFAKQPFPTKSGSCGTVVRNAE 386
OAC Rex KF303286                ALHSRVPHAVLGQGYGMTEAGPVLSMCLGFAKQPFPTKSGSCGTVVRNAE 389
G19833 Phvul.011G020200         ALRKRLPNAILGQGYGMTEGGPVLTMSLGFAKYPFPTKSGSCGTVIRNGE 366
OAC Rex KF303292                ALRKRLPNAILGQGYGMTEGGPVLTMSLGFAKYPFPTKSGSCGTVIRNGE 366
G19833 Phvul.005G184500         SVRSKFPTARLGQGYGMTEAGPVLTMSLAFAKEAMHVKAGACGTVVRNAE 371
OAC Rex KF303290                SVRSKFPTARLGQGYGMTEAGPVLTMSLAFAKEAMHVKAGACGTVVRNAE 371
G19833 Phvul.003G146900         SVRAKLPHATLGQGYGMTEAGPVLSMSLAFAKEAVHVKSGSCGTVVRNAE 372
OAC Rex KF303287                SVRAKLPHATLGQGYGMTEAGPVLSMSLAFAKEAVHVKSGSCGTVVRNAE 163
G19833 Phvul.003G147000         AVESRLPHATFGQGYGMTEAGP-LAISMAFAKEPSNIKPGACGTVVRNAE 376
OAC Rex KF303288                AVESRLPHATFGQGYGMTEAGP-LAISMAFAKEPSNIKPGACGTVVRNAE 376
G19833 Phvul.006G057300         AFEHKFPGVSVQEAYGLTEHSC-ITLTLAQKGLG-SLHKNSVGFVLPNLE 384
OAC Rex KF303291                AFEHKFPGVSVQEAYGLTEHSC-ITLTLAQKGLG-SLHKNSVGFVLPNLE 384


                                                 GEICIRG                           BOX II
G19833 Phvul.005G131400         AQIVSVDTLKALPPGQLGEIWVRGPNMMQGYHNNPQATRLTIDKKGWVHT 414
OAC Rex KF303289                AQIVSVDTLKALPPGQLGEIWVRGPNMMQGYHNNPQATRLTIDKKGWVHT 414
G19833 Phvul.011G084300         AQIVTVDTQKPLPPTQSGEIWVRGPNMMKGYHNNPQATRLTIDEKGWVHT 426
OAC Rex KF303293                AQIVTVDTQKPLPPTQSGEIWVRGPNMMKGYHNNPQATRLTIDEKGWVHT 426
G19833 Phvul.002G040100         LKVVDPETGRSLGYNQPGEICIRGLQIMKGYLNDDQATASTIDAEGWLHT 436
OAC Rex KF303286                LKVVDPETGRSLGYNQPGEICIRGLQIMKGYLNDDQATASTIDAEGWLHT 439
G19833 Phvul.011G020200         LKIIDPLTSFSLPRNHSGEICVRGPQIMKGYLNDEEATAAAIDGDGWLHT 416
OAC Rex KF303292                LKIIDPLTSFSLPRNHSGEICVRGPQIMKGYLNDEEATAAAIDGDGWLHT 416
G19833 Phvul.005G184500         MKIVDPETAQSLPRNHSGEICIRGDQIMKGYLNDKEATERTIDKEGWLHT 421
OAC Rex KF303290                MKIVDPETAQSLPRNHSGEICIRGDQIMKGYLNDKEATERTIDKEGWLHT 421
G19833 Phvul.003G146900         MKIIDPHTAASLHRNQAGEICIRGNQIMKGYLNDEEATKITIDNDGWLHT 422
OAC Rex KF303287                MKIIDPHTAASLHRNQAGEICIRGNQIMKGYLNDEEATKITIDKDGWLHT 213
G19833 Phvul.003G147000         MKIVDTETGVSLPKNETGEICIRGTKVMKGYLNDPEATERTIDKDGWLHT 426
OAC Rex KF303288                MKIVDTETGVSLPKNETGEICIRGTKVMKGYLNDPEATERTIDKDGWLHT 426
G19833 Phvul.006G057300         VKFVDPDTGRSLPRNTPGELCVRSQCVMQGYYKQVDETAQTIDKNGWLHT 434
OAC Rex KF303291                VKFVDPDTGRSLPRNTPGELCVRSQCVMQGYYKQVDETAQTIDKNGWLHT 434
```

```
                                 *           *  * *   * ****
                                 &           &  & & & &    &
                                                        |||
G19833 Phvul.005G131400         GDLGYFDEDGQLFVVDRIKELIKYKGFQVAPAELEGLLVSHPEILDAVVI 464
OAC Rex KF303289                GDLGYFDEDGQLFVVDRIKELIKYKGFQVAPAELEGLLVSHPEILDAVVI 464
G19833 Phvul.011G084300         GDIGYFDEDGQLYVVDRIKELIKYKGFQVAPAELEGLLISHPEILDAVVV 476
OAC Rex KF303293                GDIGYFDEDGQLYVVDRIKELIKYKGFQVAPAELEGLLISHPEILDAVVV 476
G19833 Phvul.002G040100         GDVGYVDDDDEIFIVDRVKELIKFKGFQVPPAELEGLLVSHPSIADAAVV 486
OAC Rex KF303286                GDVGYVDDDDEIFIVDRVKELIKFKGFQVPPAELEGLLVSHPSIADAAVV 489
G19833 Phvul.011G020200         GDIGYVDDDDEVFLVDRAKELIKFKGFQVPPAELEDLLMRHPSIADAAVV 466
OAC Rex KF303292                GDIGYVDDDDEVFLVDRAKELIKFKGFQVPPAELEDLLMRHPSIADAAVV 466
G19833 Phvul.005G184500         GDIGYIDEDDELFIVDRLKELIKYKGFQVAPAELEALLLTHPNISDAAVV 471
OAC Rex KF303290                GDIGYIDEDDELFIVDRLKELIKYKGFQVAPAELEALLLTHPNISDAAVV 471
G19833 Phvul.003G146900         GDIGYMDDDDQIFIVDRLKELIKYKGFQVAPAELEAMLIVHPNISEAVVV 472
OAC Rex KF303287                GDIGYMDDDDQIFIVDRLKELIKYKGFQVAPAELEAMLIVHPNISEAVVV 263
G19833 Phvul.003G147000         GDIGYIDDDDELFIVDRLKELIKYKGFQVAPAELEALLIAHPNISEAAVV 476
OAC Rex KF303288                GDIGYIDDDDELFIVDRLKELIKYKGFQVAPAELEALLIAHPNISEAAVV 476
G19833 Phvul.006G057300         GDIGFIDDEENVFIIDRIKELIKYKGFQVAPAELEAILLSHSSVEDAAVV 484
OAC Rex KF303291                GDIGFIDDEENVFIIDRIKELIKYKGFQVAPAELEAILLSHSSVEDAAVV 484


                                                                 *     *
                                                                 |     |
G19833 Phvul.005G131400         PYPDAEAGEVPVAYVVRSPNS-SLTEEDVQKFIAKQVAPFKRLRRVTFIN 513
OAC Rex KF303289                PYPDAEAGEVPVAYVVRSPNS-SLTEEDVQKFIAKQVAPFKRLRRVTFIN 513
G19833 Phvul.011G084300         PYPDDEAGEVPIAYVVRSPNS-SLTEEEIKKFIANQVAPFKKLRRVTFIS 525
OAC Rex KF303293                PYPDDEAGEVPIAYVVRSPNS-SLTEEEIKKFIANQVAPFKKLRRVTFIS 525
G19833 Phvul.002G040100         PQKDVVAGELPVAFVVRS-NGFDLTEEAVKEFIAKQVVFYKRLHKVYFVH 535
OAC Rex KF303286                PQKDVVAGELPVAFVVRS-NGFDLTEEAVKEFIAKQVVFYKRLHKVYFVH 538
G19833 Phvul.011G020200         PQNDDAAGEVPVAFVVS----FDLTEDAVKDFIAKQVVFYKRLHKVYFVD 512
OAC Rex KF303292                PQNDDAAGEVPVAFVVS----FDLTEDAVKDFIAKQVVFYKRLHKVYFVD 512
G19833 Phvul.005G184500         PMKDEAAGEVPVAFVVRSNACIDTTQDEIKHFISKQVVFYKRISTVFFVD 521
OAC Rex KF303290                PMKDEAAGEVPVAFVVRSNACIDTTQDEIKHFISKQVVFYKRISTVFFVD 521
G19833 Phvul.003G146900         SMKDEVAGEVPVAFVVLSNGS-KISEEEIKQYISKQVVFYKRISRVFFVG 521
OAC Rex KF303287                SMKDEVAGEVPVAFVVLSNGS-KISEEEIKQYISKQVVFYKRISRVFFVG 312
G19833 Phvul.003G147000         PMKDEAAGEIPVAFVVRSKGS-EITEDEIKQYISQQVVFYKRIGRVFFTD 525
OAC Rex KF303288                PMKDEAAGEIPVAFVVRSKGS-EITEDEIKQYISQQVVFYKRIGRVFFTD 525
G19833 Phvul.006G057300         PLPDEEAGEIPAASVVLSPGA-KESEEDIMNYVANNAAHYKKVRVVHFVE 533
OAC Rex KF303291                PLPDEEAGEIPAASVVLSPGA-KESEEDIMNYVANNAAHYKKVRVVHFVE 533


G19833 Phvul.005G131400         AVPKTASGKILRRELIEKVRSKI----------- 536
OAC Rex KF303289                AVPKTASGKILRRELIEKVRSKI----------- 536
G19833 Phvul.011G084300         SVPRTSSGKILRREITAKARSKI----------- 548
OAC Rex KF303293                SVPRTSSGKILRREITAKARSKI----------- 548
G19833 Phvul.002G040100         AIPKSPSGKILRKDLRAKLEAAAAAAA-TPTP-- 566
OAC Rex KF303286                AIPKSPSGKILRKDLRAKLEAAAAAAAATPTP-- 570
G19833 Phvul.011G020200         EIPKSSTGKILRKELRAKVGSIIQRT-------- 538
OAC Rex KF303292                EIPKSSTGKILRKELRAKVGSIIQRT-------- 538
G19833 Phvul.005G184500         AIPKSPSGKILRKDLRAKLAAPAAVSK------- 548
OAC Rex KF303290                AIPKSPSGKILRKDLRAKLAAPAAVSK------- 548
G19833 Phvul.003G146900         SIPKAASGKILRRELRARLANGLPSS-------- 547
OAC Rex KF303287                SIPKAASGKILRRELRARLANGLPSS-------- 338
G19833 Phvul.003G147000         SIPKAASGKILRKVLTARLNEGLVVAN------- 552
OAC Rex KF303288                SIPKAASGKILRKVLTARLNEGLVVAN------- 552
G19833 Phvul.006G057300         TIPKSPSGKIMRRLIKEKMVEKIKTSSLIKSENV 567
OAC Rex KF303291                TIPKSPSGKIMRRLIKEKMVEKIKTSSLIKSENV 567


Conserved features:

* Active site
& AMP binding site
| Putative CoA binding site
# Acyl-activating enzyme consensus motif
```
