# Supplementary material for: In silico comparison of genomic regions containing genes coding for enzymes and transcription factors for the phenylpropanoid pathway in Phaseolus vulgaris L. and Glycine max L. Merr
Source: Front Plant Sci. 2013 Sep 5;4:317. doi: 10.3389/fpls.2013.00317 (PMC3763686; doi:10.3389/fpls.2013.00317)
Supplement: Figure S1 — Phylogenetic relationships among common bean and soybean 4CL homologous proteins. Trees were generated from 4CL protein sequence analyses in CLC Genomics Workbench 3 using Neigbor Joining (NJ) algorithm and 1000 bootstrap replications. Scale represents genetic distance and node numbers indicate bootstrap support values. (A) Primary transcripts (98 protein sequences); (B) Twenty-one 4CL proteins selected based on similarity to soybean 4CL1 (red), 4CL2 (blue) and 4CL3/4CL4 (green). [file DataSheet1.ZIP › Supplementary Material/Supplemental Table S1 - Pv in silico map.doc]

**Table S1 | Common bean phenylpropanoid pathway gene sequence-based *in silico* map.**

| **Chromosome** | **Feature** | **Genome position (nt)** |
| --- | --- | --- |
| Pv1 | g1795 | 1071371 |
|  | DFR1-AF167556-1 | 1086866 |
|  | DFR2-EF187612-1 | 1086866 |
|  | DFR1-NM_001251683-1a | 1086866 |
|  | DFR2-DQ026299-1b | 1086866 |
|  | DFR2-M | 1086866 |
|  | DFR1-AF167556-2 | 1093579 |
|  | DFR2-EF187612-2 | 1093579 |
|  | DFR1-NM_001251683-1b | 1093579 |
|  | DFR2-DQ026299-1a | 1093579 |
|  | DFR3-M | 1093579 |
|  | g1645 | 1466733 |
|  | g2145 | 2070145 |
|  | g2266 | 2450251 |
|  | g1410 | 5493230 |
|  | g822 | 6893444 |
|  | g794 | 7868270 |
|  | g934 | 8000345 |
|  | Myb5-NM_112200 | 9405726 |
|  | CHS-X06411-6 | 14547185 |
|  | CHS-EF623853-6 | 14547185 |
|  | CHS-A-Z | 14547185 |
|  | g2647 | 31807268 |
|  | C3H-NM_180006 | 33062004 |
|  | C3H-AY065995 | 33062004 |
|  | C3H-CV670754 | 33062004 |
|  | g743 | 34341152 |
|  | g1737 | 36683028 |
|  | g2490 | 37401036 |
|  | g2580 | 39142072 |
|  | g1959 | 40167176 |
|  | AN1(bHLH)-AF260919-2 | 40413941 |
|  | g724 | 40842120 |
|  | TTG2-AF516172 | 43670815 |
|  | g1771 | 43892176 |
|  | PAL1-M11939-2 | 44141798 |
|  | PAL1-M11939-1 | 44150743 |
|  | PAL1-CV670758 | 44150743 |
|  | g2162 | 46226312 |
|  | g1886 | 47632304 |
|  | g2504 | 47640888 |
|  | g1404 | 48091260 |
|  | g34 | 49631826 |
|  | AAT-CA900148 | 50073685 |
|  | AAT-CV670746 | 50073685 |
|  | g499 | 50360892 |
|  | LAC-AF527604 | 50563497 |
|  | LAC-CV670743 | 50563497 |
|  | g1367 | 51028792 |
|  | g2562 | 51462160 |
|  | g683 | 51619488 |
|  | UF5GT-CV670749 | 51855780 |
|  | UF5GT-AB013596 | 51855782 |
|  | g2132 | 51952588 |
|  | g510 | 52015768 |
|  | g523 | 52150964 |
| Pv2 | g1686 | 238724 |
|  | g2101 | 680862 |
|  | g1307 | 829652 |
|  | g2020 | 952941 |
|  | g797 | 1263772 |
|  | IGlyT-FD796931 | 1800527 |
|  | g511 | 1998847 |
|  | IFR-AF202183 | 3361078 |
|  | CHS-X06411-4 | 3726784 |
|  | CHS-EF623853-2 | 3726784 |
|  | CHS-CV670744 | 3726784 |
|  | CHS-X06411-5 | 3733338 |
|  | CHS-EF623853-4 | 3733338 |
|  | g1515 | 3733987 |
|  | CHS-X06411-1 | 3765494 |
|  | CHS-EF623853-3 | 3765494 |
|  | CHS-X06411-3 | 3775079 |
|  | CHS-EF623853-5 | 3775079 |
|  | CHS-B-Z | 3775079 |
|  | 4CL3-AF002258 | 3830262 |
|  | 4CL4-X69955 | 3830262 |
|  | HIDH-AB154415 | 4420238 |
|  | F5H-U38416 | 22774390 |
|  | F5H-CV670742 | 22774390 |
|  | g316 | 26398780 |
|  | g2581 | 26399228 |
|  | g1467 | 28356532 |
|  | g321 | 28872406 |
|  | g2040 | 29059738 |
|  | ANS-AF325853 | 29354509 |
|  | ANS2-AY382829 | 29354509 |
|  | ANS3-AY382830 | 29354509 |
|  | g1801 | 29462192 |
|  | g1556 | 30219124 |
|  | g2097 | 32429354 |
|  | g457 | 32958166 |
|  | g1148 | 33358788 |
|  | CHS-X06411-2 | 33749563 |
|  | CHS-EF623853-1 | 33749563 |
|  | g2321 | 34249208 |
|  | I2`H-AB001379-1 | 34287348 |
|  | I2`H-AB001379-2 | 34293234 |
|  | g849 | 34502600 |
|  | g1784 | 34626412 |
|  | A454.p2--I(CHS)** | 34709252 |
|  | KAP2-AF293344 | 36583975 |
|  | KAP2-CV670752 | 36583975 |
|  | UF3GT-GU434274-1a | 37644973 |
|  | UF3GT-HM591298-1a | 37644973 |
|  | UF3GT-GU434274-1b | 37659893 |
|  | UF3GT-HM591298-1b | 37659893 |
|  | UF3GT-GU434274-1c | 37664881 |
|  | UF3GT-HM591298-1c | 37664881 |
|  | Satt493--O(ANR)** | 37772333 |
|  | ANR1-JF433915-1b | 38122856 |
|  | ANR2-JN098512-1a | 38122856 |
|  | ANR-EV264997 | 38130928 |
|  | ANR-CW652104 | 38130928 |
|  | ANR1-JF433915-1a | 38130928 |
|  | ANR2-JN098512-1b | 38130928 |
|  | g2113 | 38306710 |
|  | PPO-ABL6798-1 | 39065473 |
|  | g893 | 39763296 |
|  | GMENOD2B--I(CHS)** | 42551237 |
|  | g1247 | 42851508 |
|  | g1132 | 43306736 |
|  | CAD-L46856 | 43368131 |
|  | CAD-CV670740 | 43368131 |
|  | g1063 | 44127847 |
|  | g2348 | 44547552 |
|  | GL3-NC_003076.8 | 45309544 |
|  | MycA-EW678711 | 45309544 |
|  | g2540 | 45338260 |
|  | g1194 | 45385680 |
|  | DP-BE806864 | 45829124 |
|  | g680 | 46087360 |
|  | Leg120* | 46291487 |
|  | Leg301* | 46493470 |
|  | g774 | 46557436 |
|  | g693 | 46792484 |
|  | NAD(REF1)-NM_113359-2 | 47476474 |
|  | g364 | 47921216 |
|  | g656 | 48041224 |
| Pv3 | g2068 | 950004 |
|  | g2274 | 1812356 |
|  | g1830 | 4298993 |
|  | CCOMT-U20736 | 5702889 |
|  | SM304-F--I(CHS)** | 5724803 |
|  | g1805 | 5794105 |
|  | Bng12-F* | 5992897 |
|  | g2493 | 6262459 |
|  | HI4'OMT-AB091684 | 6331367 |
|  | IFS-AF195818 | 6352471 |
|  | IFS-AY195819 | 6352471 |
|  | IFS-CV670762 | 6352471 |
|  | g964 | 7760594 |
|  | g1520 | 9965230 |
|  | PER-AF145348 | 12314817 |
|  | g755 | 19492644 |
|  | BMd1-F* | 27653945 |
|  | g2108 | 28179608 |
|  | g586 | 28196980 |
|  | g2548 | 29881028 |
|  | g1656 | 29901396 |
|  | Bng75-F* | 30176546 |
|  | g417 | 30310232 |
|  | g2654 | 31019086 |
|  | g2476 | 32094312 |
|  | g125 | 34456752 |
|  | 4CL1-AF279267-2 | 34628465 |
|  | 4CL1-AF279267-1 | 34642251 |
|  | 4CL1-CV670737 | 34642251 |
|  | FOGT-Glyma13g01220 | 34817441 |
|  | g1925 | 36004276 |
|  | g2371 | 36267388 |
|  | g117 | 38478648 |
|  | Satt207--O(ANR)** | 40700569 |
|  | Myb176-FJ550209 | 40821500 |
|  | g2409 | 42036224 |
|  | g686 | 42258912 |
|  | Bng165-F* | 43004538 |
|  | CHI-AY595414 | 43280816 |
|  | g1808 | 43951012 |
|  | g2341 | 46106822 |
|  | g665 | 46955824 |
|  | g2213 | 47921080 |
|  | F3H-AY669325 | 48978646 |
|  | F3H-AY669326 | 48978646 |
|  | F3H-CW652103 | 48978646 |
|  | g2218 | 48979312 |
|  | g664 | 48997300 |
|  | Satt216--Wp(F3H1)** | 49040890 |
|  | g2558 | 49075876 |
|  | g1624 | 49607756 |
|  | IFR-CV670750 | 49874597 |
|  | g762 | 50010228 |
|  | 3RT-CV670748 | 51409536 |
|  | g1296 | 51791104 |
| Pv4 | g2467 | 1428273 |
|  | g1375 | 2918475 |
|  | g2303 | 3356081 |
|  | IF7MaT-AB291058 | 4518144 |
|  | IF7MaT-AB291059 | 4518144 |
|  | Bng71-F* | 4761456 |
|  | FLS-NM_001254319 | 6691007 |
|  | g2685 | 9110218 |
|  | G-U14S | 23820496 |
|  | BM68* | 25840516 |
|  | G-AP7S | 28378808 |
|  | UFGlyT-AF198453 | 32932991 |
|  | A3OGT-BE190894 | 33042774 |
|  | g128 | 33324172 |
|  | BM140* | 39837520 |
|  | BMd26* | 41482361 |
|  | g2595 | 42468888 |
|  | CHR-CW652099 | 42558699 |
|  | g1564 | 42893400 |
|  | BMd15* | 44019884 |
|  | Bng224-R* | 44329948 |
|  | NAD(REF1)-NM_113359-1a | 44427945 |
|  | NAD(REF1)-NM_113359-2b | 44444006 |
|  | g968 | 45287608 |
|  | g118 | 45736396 |
| Pv5 | g564 | 2347029 |
|  | DP-DQ395252 | 3016733 |
|  | g136 | 3058017 |
|  | g1188 | 3615107 |
|  | g1676 | 5584160 |
|  | g2596 | 6708071 |
|  | CHI-AY595416 | 9760705 |
|  | g1226 | 9928089 |
|  | g1395 | 11803387 |
|  | g2507 | 20216608 |
|  | g292 | 24354292 |
|  | g985 | 33741464 |
|  | g2410 | 34743168 |
|  | g1968 | 36351588 |
|  | g1773 | 36864696 |
|  | g174 | 37094700 |
|  | CV533374 | 37702936 |
|  | g1333 | 37716008 |
|  | g1689 | 38017356 |
|  | g2308 | 38647688 |
|  | g1664 | 39818728 |
|  | g1883 | 40021548 |
|  | CCR-NM_101463 | 40358576 |
|  | CCR-CV670739 | 40358576 |
|  | 4CL2-AF002259 | 40478925 |
|  | 4CL2-CV670738 | 40478925 |
| Pv6 | F3`5`H-AY117551 | 9243136 |
|  | g2538 | 10189094 |
|  | V-OD12800-R* | 10480551 |
|  | g2553 | 12388878 |
|  | g1639 | 15727170 |
|  | g1436 | 16378574 |
|  | F6H-XM_003551443 | 16959683 |
|  | g139 | 17667572 |
|  | BMd12* | 17992245 |
|  | g471 | 18476192 |
|  | g1192 | 19502500 |
|  | g2329 | 21329416 |
|  | g1361 | 22459962 |
|  | Myb4-CA902489 | 23032617 |
|  | Myb4-CW652106 | 23032617 |
|  | PERFBP1-AF149277-2 | 24384794 |
|  | PERFBP1-AF149277-9 | 24394199 |
|  | PERFBP1-AF149277-4 | 24400830 |
|  | PERFBP1-AF149277-5 | 24407240 |
|  | PERFBP1-AF149277-1 | 24415433 |
|  | PER-CW652094 | 24415433 |
|  | PERFBP1-AF149277-8 | 24427640 |
|  | PERFBP1-AF149277-7 | 24444737 |
|  | PERFBP1-AF149277-3 | 24448722 |
|  | PERFBP1-AF149277-6 | 24462655 |
|  | PERFBP1-AF149277-10 | 24475020 |
|  | g1748 | 25439492 |
|  | Bng27-F* | 25589898 |
|  | g1249 | 25826556 |
|  | g1159 | 26195090 |
|  | g861 | 27967476 |
|  | g1488 | 28199112 |
|  | g2192 | 28863988 |
|  | 7IOMT-CV670751 | 29034073 |
|  | g1998 | 29502320 |
|  | g2208 | 29612498 |
|  | PPO-ABL67928 | 29658118 |
|  | g1818 | 29854156 |
|  | g1280 | 30261220 |
|  | FNGalT-DB977791 | 30546387 |
|  | g1757 | 30743952 |
|  | PER-CW652095 | 30963525 |
|  | PERFBP4-AF149279 | 30963881 |
|  | 3RT-BE211343-1b | 31054461 |
|  | 3RT-BE211343-1a | 31057924 |
|  | g1190 | 31283580 |
|  | g634 | 31613658 |
|  | g2383 | 31664860 |
|  | g2480 | 31664976 |
|  | g1174 | 31674056 |
|  | g1957 | 31761604 |
| Pv7 | CHI-CV670745 | 599228 |
|  | g2298 | 1562320 |
|  | g1719 | 1599427 |
|  | C4H-Y09447 | 1889412 |
|  | C4H-CV670736 | 1889412 |
|  | MybTT2-1-L | 2210695 |
|  | g1615 | 2858517 |
|  | CAD-X62344 | 3217682 |
|  | Bng42-F* | 3577315 |
|  | g501 | 3767388 |
|  | g2129 | 4252992 |
|  | g1233 | 6845683 |
|  | SM304-R--I(CHS)** | 7750412 |
|  | g2531 | 8035832 |
|  | g366 | 8036017 |
|  | g1378 | 9010955 |
|  | g487 | 9356108 |
|  | PAL2-P19142-3 | 10062183 |
|  | PAL2-P19142-2 | 10124135 |
|  | LAR-XM_003556213 | 11417842 |
|  | LAR-XM_003536262 | 11417842 |
|  | LAR1-JF433916 | 11417842 |
|  | Myb15-CA902486 | 13461806 |
|  | Myb15-CW652102 | 13461806 |
|  | Myb15-Z | 13461806 |
|  | BM210* | 31123662 |
|  | Bng47-F* | 31976851 |
|  | g415 | 36797748 |
|  | PAL2-P19142-1 | 37020027 |
|  | PAL2-CV670734 | 37020027 |
|  | PAL2-Z | 37020027 |
|  | Bng204-R* | 37401195 |
|  | PVBR269* | 38741363 |
|  | Leg146* | 40306818 |
|  | AN1(bHLH)-GD853858 | 40383553 |
|  | AN1(bHLH)-AF260919-1 | 40383553 |
|  | Myb111-NM_124310 | 45475635 |
|  | g1065 | 46444520 |
|  | G4DT-AB434690 | 46735189 |
|  | g1357 | 46951108 |
|  | g1853 | 47125820 |
|  | g1380 | 48138496 |
|  | DFR1-AF167556-3 | 48573352 |
|  | DFR2-EF187612-3 | 48573352 |
|  | DFR1-NM_001251683-2 | 48573352 |
|  | DFR2-DQ026299-2 | 48573352 |
|  | DFR-Z | 48573352 |
|  | DFR1-M | 48573352 |
|  | g1947 | 48785376 |
|  | g290 | 48956248 |
|  | Bng47-R* | 50145181 |
| Pv8 | g1084 | 155567 |
|  | g1588 | 418104 |
|  | g1786 | 478500 |
|  | BMd25* | 931678 |
|  | g2311 | 1161284 |
|  | g89 | 1317280 |
|  | g2393 | 1539083 |
|  | g1879 | 2026312 |
|  | 7IOMT-U97125-1 | 2695482 |
|  | 7IOMT-U97125-2 | 2701370 |
|  | g1119 | 3156032 |
|  | MybAN2-FG986768 | 3225601 |
|  | MybA-Glyma09g36990-1d | 3225601 |
|  | MybA-Glyma09g36990-1a | 3242793 |
|  | MybA-Glyma09g36990-1c | 3254754 |
|  | MybA-Glyma09g36990-1b | 3273361 |
|  | Gy-W17S | 3282867 |
|  | Gy-OW17600-R* | 3283473 |
|  | Sat_293--R(?)** | 3491559 |
|  | BMd44* | 3735968 |
|  | g2567 | 3778302 |
|  | g2462 | 4796974 |
|  | g696 | 4847854 |
|  | g2561 | 4866851 |
|  | g131 | 5125991 |
|  | g549 | 5377477 |
|  | g1031 | 6545931 |
|  | HD-CA902455 | 6744457 |
|  | HD-CV670753 | 6744457 |
|  | PPO-ABL67928 | 6844049 |
|  | VR-EU925557-1 | 7319897 |
|  | VR-EU925557-2 | 7325513 |
|  | g1990 | 7487417 |
|  | BM165* | 7854943 |
|  | C-OAP2700-F* | 9799367 |
|  | g776 | 11935053 |
|  | BM153* | 13325926 |
|  | g140 | 13644236 |
|  | GST1-AY220095 | 13646227 |
|  | GST1-CW652097 | 13646227 |
|  | g2514 | 18366728 |
|  | IFGT-EV280635 | 20063721 |
|  | g440 | 25433944 |
|  | g1758 | 30160900 |
|  | FLS-NM_001250490 | 42855237 |
|  | Satt160--W1(F3'5'H)** | 43572866 |
|  | WIP-CA902512 | 45920629 |
|  | WIP-CW652101 | 45920629 |
|  | GST2-AY099257 | 50329170 |
|  | GST2-CV670756 | 50329170 |
|  | VT-CA907034 | 50487048 |
|  | VT-CV670747 | 50487048 |
|  | g580 | 51211756 |
|  | g1181 | 53880420 |
|  | g2264 | 53912760 |
|  | g1224 | 54518859 |
|  | g1713 | 55417148 |
|  | g1858 | 56222944 |
|  | g2413 | 56433440 |
|  | g2358 | 56886268 |
|  | g2151 | 57280432 |
|  | g2316 | 57771000 |
|  | g796 | 58081616 |
|  | Bng73-F* | 58454291 |
|  | g2061 | 58813088 |
|  | CHR-EU921437 | 59169950 |
|  | PAL3-Z | 59310387 |
|  | PAL3-P19143 | 59310665 |
|  | PAL3-CV670735 | 59310665 |
| Pv9 | BMd52* | 5750860 |
|  | g2584 | 6429831 |
|  | FOMT-Glyma04g40580 | 8600878 |
|  | COMT-M63853 | 8600878 |
|  | COMT-M73235 | 8600878 |
|  | COMT-CV670741 | 8600878 |
|  | TTG1-AJ133743 | 8685737 |
|  | PTR-TC433083 | 10600501 |
|  | g544 | 10629212 |
|  | T-OM19400-F* | 11404925 |
|  | g2178 | 11853213 |
|  | LIM-BU764417 | 12021375 |
|  | LIM-CW652092 | 12021375 |
|  | Bng129-F* | 13009966 |
|  | g791 | 13266937 |
|  | g1286 | 14315669 |
|  | g1884 | 14499886 |
|  | g2498 | 15832316 |
|  | g993 | 16238963 |
|  | g1107 | 16713947 |
|  | g732 | 17624544 |
|  | Myb42-AM156908 | 17794990 |
|  | Bng5-F* | 18933854 |
|  | g2272 | 19713104 |
|  | g792 | 20515140 |
|  | CHI-AY595417 | 20929174 |
|  | g1671 | 23557877 |
|  | g708 | 24164370 |
|  | g2613 | 24582148 |
|  | g2516 | 24594392 |
|  | g1206 | 25374364 |
|  | g1126 | 26616516 |
|  | F3`H-AB061212 | 28477571 |
|  | F3`H-CV670760 | 28477571 |
|  | F3H-EV190438(T) | 28477571 |
|  | SN317-F--T(F3'H)** | 28478155 |
|  | g1379 | 33187724 |
|  | g2335 | 35927040 |
|  | g195 | 35927220 |
|  | g385 | 35990012 |
|  | g860 | 36052048 |
|  | g2209 | 36534776 |
| Pv10 | g2221 | 2032367 |
|  | Bng200-R* | 6019047 |
|  | g2521 | 6884394 |
|  | BM157* | 8999124 |
|  | Ana-OM9200-R* | 11577315 |
|  | Bip-OJ17700-R* | 21532271 |
|  | g1029 | 23528972 |
|  | Myb11-NM_116126 | 29502170 |
|  | g1320 | 32127556 |
|  | CPRF2-X58577 | 36976049 |
|  | Myb12-NM_130314 | 38186999 |
|  | g1994 | 38598968 |
|  | Bng68-F* | 38622087 |
|  | g1643 | 39278744 |
|  | Myb123-AJ299452 | 40062049 |
|  | L-OL4525-F* | 40286884 |
|  | g2600 | 40697760 |
|  | g2560 | 41571608 |
|  | g1724 | 41896708 |
|  | BMd42* | 41936831 |
|  | g2268 | 41953296 |
|  | g1341 | 42092640 |
|  | g1661 | 42569060 |
|  | CCR-AF320623 | 42889543 |
|  | HCT-AJ582651 | 43002237 |
| Pv11 | g909 | 169275 |
|  | g1329 | 328771 |
|  | MybL2-NM_105772-1 | 3061388 |
|  | g785 | 3709117 |
|  | IFR-AF202184 | 3852808 |
|  | g2273 | 3958859 |
|  | g1731 | 4828365 |
|  | g1415 | 5218185 |
|  | g1932 | 5294374 |
|  | g2307 | 5761039 |
|  | FNS-GU575290 | 5897231 |
|  | FNS-FJ767773 | 5897231 |
|  | g2285 | 6387568 |
|  | g1438 | 6442565 |
|  | g835 | 7396845 |
|  | g1489 | 8863810 |
|  | PER-AF019116 | 12340871 |
|  | g2527 | 12854591 |
|  | MybTT2-2-L | 13946276 |
|  | g1510 | 15079622 |
|  | g1168 | 26735040 |
|  | g156 | 38890528 |
|  | g2135 | 46008884 |
|  | g1983 | 48167288 |
|  | g1215 | 49713356 |
