# Supplementary material for: In silico comparison of genomic regions containing genes coding for enzymes and transcription factors for the phenylpropanoid pathway in Phaseolus vulgaris L. and Glycine max L. Merr
Source: Front Plant Sci. 2013 Sep 5;4:317. doi: 10.3389/fpls.2013.00317 (PMC3763686; doi:10.3389/fpls.2013.00317)
Supplement: Figure S1 — Phylogenetic relationships among common bean and soybean 4CL homologous proteins. Trees were generated from 4CL protein sequence analyses in CLC Genomics Workbench 3 using Neigbor Joining (NJ) algorithm and 1000 bootstrap replications. Scale represents genetic distance and node numbers indicate bootstrap support values. (A) Primary transcripts (98 protein sequences); (B) Twenty-one 4CL proteins selected based on similarity to soybean 4CL1 (red), 4CL2 (blue) and 4CL3/4CL4 (green). [file DataSheet1.ZIP › Supplementary Material/Supplemental Table S2 - Gm in silico map.doc]

**Table S2 | Soybean phenylpropanoid pathway gene sequence-based *in silico* map.**

| **Chromosome** | **Feature** | **Chromosome position (nt)** |
| --- | --- | --- |
| Gm01 | g316 | 992725 |
|  | g2581 | 992725 |
|  | CAD-L46856-3 | 2102227 |
|  | g2040 | 2727694 |
|  | g1830 | 8874870 |
|  | Ana-OM9200-R-1* | 25902501 |
|  | g2108 | 27963671 |
|  | CHS-X06411-2b | 29149920 |
|  | GST1-AY220095-5b | 34484153 |
|  | GST1-AY220095-5a | 34508538 |
|  | g968 | 38308111 |
|  | TTG2-AF516172-2 | 43469371 |
|  | F3H-AY669326-3 | 49513580 |
|  | F5H-U38416-1 | 49781379 |
|  | IFR1-AF202183-2a | 50095780 |
|  | IFR1-AF202183-2b | 50121925 |
|  | IFR1-AF202183-2c | 50131015 |
|  | IFR1-AF202183-2d | 50135212 |
|  | g511 | 51321328 |
|  | g797 | 51939526 |
|  | g1307 | 52339317 |
|  | g2101 | 52462429 |
|  | g1686 | 52795980 |
|  | ANS-AF325853-1 | 53622048 |
|  | ANS2-AY382829-1 | 53622048 |
|  | ANS3-AY382830-1 | 53622048 |
|  | g1801 | 53693495 |
|  | CHS-X06411-2a(CHS7) | 54742696 |
|  | CHS-CV670744-1 | 54742696 |
|  | CHS-B-Z-2 | 54742696 |
|  | g1515 | 54742696 |
|  | 4CL4-X69955-1 | 54974200 |
|  | HIDH-AB154415-1 | 55451075 |
| Gm02 | BMd25-1* | 19669 |
|  | g786 | 936546 |
|  | Myb12-NM_130314-1 | 1261281 |
|  | g2040 | 3564468 |
|  | Satt216--Wp(F3H1)** | 4052712 |
|  | g2558 | 4324525 |
|  | F3H-AY669326-1a | 4388762 |
|  | F3H-CW652103-1 | 4388762 |
|  | g2218 | 4388762 |
|  | SN318-F--Wp(F3H1)** | 4389914 |
|  | F3H-AY669326-1b | 4397313 |
|  | g664 | 4411071 |
|  | g2213 | 5235481 |
|  | BM68-1* | 6976306 |
|  | g89 | 8427139 |
|  | g2108 | 9219066 |
|  | g1805 | 9624724 |
|  | CCOMT-U20736-2 | 9737811 |
|  | g1830 | 11601786 |
|  | CHS-X06411-5 | 13039403 |
|  | g1296 | 13436734 |
|  | g964 | 14324201 |
|  | AN1(bHLH)-AF260919 | 15047873 |
|  | BMd15-1* | 16502679 |
|  | g290 | 16871344 |
|  | DFR-EF187612 | 17169065 |
|  | DFR1-NM_001251683-3 | 17169065 |
|  | DFR2-DQ026299-3 | 17169065 |
|  | DFR-Z-1 | 17169065 |
|  | DFR1-M-2 | 17169065 |
|  | g1731 | 27007817 |
|  | DP-DQ395252 | 27493907 |
|  | Bng47-F-1* | 27906411 |
|  | PER-FBP1-AF149277-4 | 30138764 |
|  | CHR-EU921437-1b | 34348288 |
|  | g2371 | 41825978 |
|  | g1925 | 42101743 |
|  | g2151 | 44573693 |
|  | Bng73-F-1* | 44682634 |
|  | LAC-AF527604-4b | 44958918 |
|  | g2413 | 45218644 |
|  | C4H-J09447-4 | 45490798 |
|  | g1858 | 45490798 |
|  | g1713 | 46037511 |
|  | AAT-CA900148-4 | 47265637 |
|  | g2264 | 47295297 |
|  | g1181 | 47319664 |
|  | g2274 | 47328286 |
|  | LAC-AF527604-4a | 47846407 |
|  | LAC-CV670743-1 | 47846408 |
|  | g580 | 49195434 |
|  | GST2-CV670756-1 | 49624876 |
|  | GST2-AY099257-1 | 49624877 |
|  | g2061 | 49709174 |
|  | g2316 | 50155094 |
|  | g796 | 50317605 |
|  | CHR-EU921437-1a | 51242652 |
|  | PAL3-P19142-1 | 51366326 |
|  | PAL3-Z-1 | 51366328 |
| Gm03 | HD-GL2l1/ANL2-CA902455-4 | 1606119 |
|  | g1320 | 2115730 |
|  | g1029 | 8115861 |
|  | g968 | 10376440 |
|  | Leg146-2* | 14392187 |
|  | GST1-AY220095-4a | 21076741 |
|  | GST1-AY220095-4b | 21083396 |
|  | Bng200-R-1* | 31426484 |
|  | BM157-1* | 34718863 |
|  | C3H-CV670754-2 | 35505244 |
|  | C3H-AY065995-2 | 35505245 |
|  | g743 | 36054786 |
|  | g1737 | 36907805 |
|  | g2490 | 37317236 |
|  | g440 | 37827275 |
|  | g2580 | 38040593 |
|  | g1959 | 38588864 |
|  | g724 | 39052478 |
|  | TTG2-AF516172 | 40963198 |
|  | g1771 | 41145845 |
|  | PAL1-M11939-2b | 41364183 |
|  | PAL1-CV670758-2 | 41364184 |
|  | PAL1-M11939-2a | 41374989 |
|  | g1952 | 42126110 |
|  | g1954 | 42126110 |
|  | TTG1-AJ133743-3 | 42749356 |
|  | g2162 | 43110265 |
|  | Myb111-NM_124319-4 | 44182072 |
|  | g1886 | 44289084 |
|  | g2504 | 44295867 |
|  | g1404 | 44683193 |
|  | CPRF2-X58577-4 | 46394192 |
|  | g1361 | 46881239 |
| Gm04 | g732 | 189069 |
|  | Myb42-AM156908 | 309418 |
|  | IFR2-AF202184 | 871805 |
|  | BMd52-2* | 982371 |
|  | g544 | 2431933 |
|  | PTR-TC433083-2 | 2446812 |
|  | g2178 | 3531798 |
|  | LIM-BU764417-2a | 3635587 |
|  | LIM-CW652092-2 | 3635587 |
|  | g791 | 4603818 |
|  | g1286 | 5466276 |
|  | g1884 | 5620083 |
|  | g2498 | 6579279 |
|  | g993 | 6862755 |
|  | g1107 | 7255767 |
|  | g2584 | 9828691 |
|  | PPO-ABL67928-4 | 14306524 |
|  | g564 | 19074951 |
|  | g2135 | 29747125 |
|  | g136 | 32802988 |
|  | LIM-BU764417-2b | 41329682 |
|  | g1126 | 41449809 |
|  | g1206 | 42684729 |
|  | g2516 | 43359290 |
|  | g2613 | 43366123 |
|  | g708 | 43710219 |
|  | g792 | 45899076 |
|  | CHI4B-AY595417-2 | 46175881 |
|  | COMT-CV670741-2 | 46576629 |
|  | FOMT-Glyma04g40458-1 | 46576629 |
|  | COMT-M63853-2a | 46576629 |
|  | COMT-M63853-2b | 46580329 |
|  | TTG1-AJ133743-2 | 46614102 |
|  | g2272 | 47863756 |
|  | Bng5-F-2* | 48284346 |
| Gm05 | F3'H-CV670760-2 | 242329 |
|  | F3'H-AB061212-2 | 242562 |
|  | Myb176-FJ550209-1 | 1127206 |
|  | g2409 | 2052973 |
|  | g686 | 2262584 |
|  | g128 | 2490762 |
|  | g1808 | 4024413 |
|  | Myb5-NM_112200-5 | 4075972 |
|  | g2467 | 8432965 |
|  | FLS-NM_001254319-1 | 12981662 |
|  | g1436 | 14476143 |
|  | g2341 | 26710023 |
|  | KAP2-CV670752-1 | 31618404 |
|  | KAP2-AF293344-1 | 31618405 |
|  | g1784 | 32299965 |
|  | g849 | 32375062 |
|  | g2321 | 32469820 |
|  | g1148 | 32787312 |
|  | g457 | 33482127 |
|  | g2097 | 33793874 |
|  | CCOMT-TC428575-1 | 33816674 |
|  | g683 | 33841617 |
|  | UF5GT-AB013596-2a | 34171449 |
|  | UF5GT-AB013596-2b | 34177627 |
|  | UF5GT-CV670749-2 | 34177628 |
|  | g2493 | 34292597 |
|  | CHS-X06411-3(CHS2) | 34420945 |
|  | Bng165-F-2* | 34962484 |
|  | g1247 | 36611301 |
|  | g1132 | 37026365 |
|  | CAD-L46856-2 | 37097574 |
|  | CAD-CV670740-1 | 37097576 |
|  | g1063 | 37812808 |
|  | g364 | 39084764 |
|  | NAD(REF1)-NM_113359-4 | 39408189 |
|  | g693 | 40044981 |
|  | g774 | 40244394 |
|  | Leg301-2* | 40311685 |
|  | Leg120-2* | 40492118 |
|  | g680 | 40639245 |
|  | DP-BE806864-1 | 40844769 |
|  | g1194 | 41238011 |
|  | g2540 | 41267148 |
|  | GL3-NM_148067.3-2 | 41301147 |
|  | MycA-EW678711-1 | 41301147 |
|  | g2348 | 41919826 |
| Gm06 | g732 | 229125 |
|  | Myb42-AM156908 | 342193 |
|  | BMd52-1* | 986678 |
|  | PTR-TC433083-1 | 2398134 |
|  | g2178 | 3426133 |
|  | LIM-BU764417-1a | 3517994 |
|  | LIM-CW652092-1 | 3517994 |
|  | Bng129-F-1* | 4152590 |
|  | g1286 | 5171163 |
|  | g1884 | 5332403 |
|  | g2498 | 6214144 |
|  | g993 | 6451805 |
|  | g1107 | 6770807 |
|  | g2584 | 8448323 |
|  | FLS1-NM_001250490-2 | 8935983 |
|  | Bng5-F-1* | 9425527 |
|  | g2272 | 9880418 |
|  | TTG1-AJ133743-1 | 11175571 |
|  | FOMT-Glyma04g40580-2a | 11198586 |
|  | COMT-M63853-1a | 11198586 |
|  | COMT-CV670741-1 | 11198586 |
|  | FOMT-Glyma04g40580-2b | 11205832 |
|  | COMT-M63853-1b | 11205832 |
|  | FOMT-Glyma04g40580-2c | 11210470 |
|  | COMT-M63853-1c | 11210470 |
|  | CHI4A-AY595417-1 | 11615532 |
|  | g792 | 11917322 |
|  | g708 | 14078497 |
|  | g2516 | 14423580 |
|  | g2613 | 14430632 |
|  | g1206 | 15052843 |
|  | g1126 | 15929171 |
|  | LIM-BU764417-1b | 16023639 |
|  | Satt286--T(F3'H)** | 16171760 |
|  | F3'H-CV670760-1 | 18534604 |
|  | F3'H-AB061212-1 | 18534651 |
|  | SL305-F--T(F3'H)** | 18539871 |
|  | SN317-F--T(F3'H)** | 18540829 |
|  | Satt365--T(F3'H)** | 19653669 |
|  | Bng12-F-1* | 27018342 |
|  | PER-FBP4-AF149279-3 | 27375519 |
|  | KAP2-AF293344-2 | 39936985 |
|  | g2507 | 40988932 |
|  | g156 | 40988932 |
|  | PPO-ABL67928-5 | 45393709 |
|  | 7IOMT-U97125-5 | 46918121 |
|  | g1168 | 47347185 |
|  | g1510 | 47901457 |
|  | MybTT2-2-L1 | 48295324 |
|  | g2527 | 48434231 |
|  | g290 | 49171217 |
|  | A3OGT-BE190894-3 | 50276299 |
| Gm07 | g2358 | 305682 |
|  | g34 | 378528 |
|  | g2560 | 1150398 |
|  | g1724 | 1509310 |
|  | BMd42-2* | 1545898 |
|  | g2268 | 1559801 |
|  | HCT-AJ582561-2 | 1671721 |
|  | CCR2-AF320623-2 | 1823459 |
|  | g1661 | 2295185 |
|  | g1883 | 2441522 |
|  | g1341 | 2727086 |
|  | g2600 | 2743982 |
|  | L-OL4525F-1* | 2997560 |
|  | g1643 | 3851230 |
|  | Bng68-F-2* | 4355520 |
|  | g1994 | 4377615 |
|  | Myb111-NM_124319-2a | 4673477 |
|  | MybAN2-EF423868 | 4673477 |
|  | HD-GL2l1/ANL2-CA902455-3 | 6941456 |
|  | g1320 | 7552617 |
|  | NAD(REF1)-NM_113359-2 | 8054389 |
|  | Bng224-R-1* | 8208425 |
|  | g755 | 8636103 |
|  | g1564 | 10041409 |
|  | Bng200-R-2* | 11210648 |
|  | BM157-2* | 14239631 |
|  | GST1-AY220095-2b | 16548611 |
|  | GST1-AY220095-2c | 16556854 |
|  | GST1-CW652097-2 | 16556854 |
|  | GST1-AY220095-2a | 16569606 |
|  | g776 | 17870814 |
|  | g1029 | 18300064 |
|  | UF3GT1-GU434274-1a | 35234524 |
|  | UF3GT1-GU434274-1b | 35247806 |
|  | UF3GT1-GU434274-1c | 35251110 |
|  | g2113 | 35704510 |
|  | PPO-ABL67928-1c | 36224753 |
|  | PPO-ABL67928-1f | 36264785 |
|  | PPO-ABL67928-1d | 36277825 |
|  | PPO-ABL67928-1b | 36292993 |
|  | PPO-ABL67928-1e | 36308160 |
|  | PPO-ABL67928-1a | 36320692 |
|  | g893 | 37054314 |
|  | IFS2-AF195819-2 | 37261063 |
|  | IFS-CV670762-1 | 37261064 |
|  | g964 | 37531783 |
|  | g1296 | 38521679 |
|  | CCOMT-U20736-1 | 38732608 |
|  | MybL2-NM_105772-1 | 38888508 |
|  | KAP2-CV670752-2 | 39937824 |
|  | g2548 | 41537078 |
|  | g1656 | 41537078 |
|  | g586 | 42236397 |
|  | Myb111-NM_124319-2b | 42294770 |
| Gm08 | g1063 | 329749 |
|  | g2348 | 607637 |
|  | GL3-NC_003076-1 | 1168003 |
|  | MycA-EW678711-2 | 1168003 |
|  | g2540 | 1202356 |
|  | g1194 | 1237214 |
|  | DP-BE806864-2 | 1629677 |
|  | g680 | 1807827 |
|  | Leg120-1* | 1965702 |
|  | Leg301-1* | 2174248 |
|  | g774 | 2234830 |
|  | g693 | 2449528 |
|  | NAD(REF1)-NM_113359-3a | 3063446 |
|  | NAD(REF1)-NM_113359-3b | 3069385 |
|  | g364 | 3422767 |
|  | g656 | 3590976 |
|  | Satt207--O(ANR)** | 4000296 |
|  | g2113 | 4712518 |
|  | ANR1-JF433915-1a | 4777633 |
|  | ANR2-JN098512-1b | 4777633 |
|  | ANR-EV264997(O)-1a | 4777633 |
|  | ANR-CW652104-1 | 4777633 |
|  | SN013-F--O(ANR)** | 4779786 |
|  | ANR-CW652104-2 | 4782640 |
|  | ANR1-JF433915-1b | 4782640 |
|  | ANR2-JN098512-1a | 4782640 |
|  | ANR-EV264997-1b | 4782640 |
|  | Satt493--O(ANR)** | 5027239 |
|  | UF3GT2-HM591298 | 5114568 |
|  | A454.p2--I(CHS)** | 6533713 |
|  | g1784 | 6592175 |
|  | g849 | 6659058 |
|  | I2'H-AB001379-1 | 6754465 |
|  | g2321 | 6787031 |
|  | g1148 | 7052589 |
|  | g457 | 7661330 |
|  | g2097 | 7954932 |
|  | UF5GT-AB013596-1b | 8262562 |
|  | UF5GT-AB013596-1a | 8272450 |
|  | g2493 | 8356346 |
|  | CHS-X06411-4e(CHS5) | 8380418 |
|  | CHS-X06411-4c | 8384462 |
|  | CHS-X06411-4a(CHS9) | 8462526 |
|  | CHS-X06411-4b(CHS1) | 8468361 |
|  | CHS-X06411-4d | 8472117 |
|  | CHS-X06411-4f | 8485475 |
|  | SM305-F--I(CHS)** | 8510069 |
|  | SM303-F--I(CHS)** | 8562659 |
|  | GMENOD2B--I(CHS** | 10190778 |
|  | g1247 | 10717342 |
|  | g1132 | 11123568 |
|  | CAD-L46856-1 | 11181796 |
|  | CAD-CV670740-2 | 11181796 |
|  | g1188 | 11786721 |
|  | Bng165-F-1* | 13511596 |
|  | g2596 | 14106344 |
|  | PER-FBP4-AF149279-2c | 14466236 |
|  | PER-FBP4-AF149279-2a | 14476991 |
|  | PER-FBP4-CW652095-2 | 14476991 |
|  | PER-FBP4-AF149279-2b | 14481579 |
|  | 3RT-BE211343-1 | 14593186 |
|  | g1190 | 14827181 |
|  | g634 | 15129555 |
|  | g2480 | 15189698 |
|  | g1174 | 15189698 |
|  | g2383 | 15189698 |
|  | g2600 | 15241496 |
|  | g2560 | 16235382 |
|  | g1724 | 16623260 |
|  | BMd42-1* | 16661947 |
|  | g2268 | 16675046 |
|  | g34 | 16704065 |
|  | g1341 | 16811151 |
|  | g1661 | 17333755 |
|  | CCR2-AF320623-3 | 17787776 |
|  | HCT-AJ582651-1 | 17958099 |
|  | g2358 | 18124492 |
|  | g909 | 20505227 |
|  | 7IOMT-U97125-1 | 21407859 |
|  | HI4'OMT-AB091684-3 | 21598597 |
|  | g1879 | 22547236 |
|  | g2393 | 23486724 |
|  | g762 | 29135488 |
|  | 3RT-CA914524-2a | 36530855 |
|  | 3RT-CA914524-2b | 36585019 |
|  | 3RT-CV670748-2 | 36864868 |
|  | g1520 | 40841441 |
|  | g2553 | 41432249 |
|  | GST2-AY099257-4b | 41842056 |
|  | GST2-AY099257-4a | 41850404 |
|  | g1639 | 42823807 |
|  | F6H-XM_003551443 | 43676827 |
|  | BMd12-2* | 44496462 |
|  | g139 | 44701243 |
|  | IFGT-EV280635-1a | 45440345 |
|  | IFGT-EV280635-1a | 45451402 |
|  | g2514 | 45827926 |
|  | g1084 | 46349895 |
|  | g1588 | 46591084 |
|  | g1786 | 46669031 |
| Gm09 | g1159 | 349423 |
|  | g1249 | 676453 |
|  | Bng27-F-2* | 899868 |
|  | g1748 | 1073206 |
|  | PER-FBP1-AF149277-2e | 1770834 |
|  | PER-FBP1-AF149277-2f | 1779883 |
|  | PER-FBP1-AF149277-2a | 1789829 |
|  | PER-FBP1-CW652094-1 | 1789829 |
|  | PER-FBP1-AF149277-2b | 1808653 |
|  | PER-FBP1-AF149277-2c | 1815538 |
|  | PER-FBP1-AF149277-2d | 1822866 |
|  | Myb12-NM_130314-4 | 3221871 |
|  | g2209 | 3363906 |
|  | g860 | 3876212 |
|  | g385 | 3958489 |
|  | g195 | 4031848 |
|  | g2335 | 4031848 |
|  | I2'H-AB001379-2f | 4168252 |
|  | I2'H-AB001379-2e | 4172117 |
|  | I2'H-AB001379-2c | 4181197 |
|  | I2'H-AB001379-2d | 4222344 |
|  | I2'H-AB001379-2a | 4241100 |
|  | I2'H-AB001379-2b | 4246821 |
|  | g1379 | 7653942 |
|  | CHS-X06411-6(CHS6) | 8193570 |
|  | CHS-A-Z-2 | 8193750 |
|  | Bng12-F-2* | 10662166 |
|  | 7IOMT-U97125-4a | 13240432 |
|  | 7IOMT-U97125-4b | 13291314 |
|  | A3OGT-BE190894-2c | 28796853 |
|  | A3OGT-BE190894-2b | 28836585 |
|  | UFGlyT-AF198563-1b | 28836585 |
|  | UFGlyT-AF198563-1a | 29114111 |
|  | A3OGT-BE190894-2a | 29114111 |
|  | BM140-1* | 34450054 |
|  | BMd26-1* | 35895042 |
|  | g2595 | 36785736 |
|  | CHR-CW652099-2 | 36863388 |
|  | g1564 | 37197001 |
|  | g755 | 38241249 |
|  | Bng224-R-2* | 38618040 |
|  | NAD(REF1)-NM_113359-1 | 38717888 |
|  | g2581 | 40791755 |
|  | g316 | 40791755 |
|  | IFR-CV670750-2 | 40843648 |
|  | g1329 | 42384660 |
|  | MybA-Glyma09g36990-1c | 42550341 |
|  | MybA-Glyma09g36990-1a | 42562649 |
|  | MybA-Glyma09g36990-1b | 42584534 |
|  | g1119 | 42645024 |
|  | g2303 | 42668409 |
|  | Sat_293--R(?)** | 42848788 |
|  | g2567 | 43096777 |
|  | g2462 | 43883788 |
|  | g696 | 43923593 |
|  | g2561 | 43942279 |
|  | g131 | 44151482 |
|  | g549 | 44353512 |
|  | g1031 | 45023215 |
|  | HD-GL2l1/ANL2-CA902455-1 | 45086534 |
|  | VR-EV925557-2c | 45471281 |
|  | VR-EV925557-2b | 45474436 |
|  | VR-EV925557-2a | 45480189 |
|  | g1990 | 45547606 |
|  | HD-CV670753-1 | 45986536 |
| Gm10 | Myb15-CA902486-1b | 648359 |
|  | Myb111-NM_124310-1 | 1311954 |
|  | Leg146-1* | 2197707 |
|  | AN1(bHLH)-GD853858 | 2254639 |
|  | g415 | 5181160 |
|  | PAL2-P19142-2a | 5306846 |
|  | PAL2-CV670734-1 | 5306846 |
|  | PAL3-CV670735-1 | 5306846 |
|  | PAL2-Z-1 | 5306846 |
|  | g1773 | 5837270 |
|  | g1065 | 6185830 |
|  | g1357 | 7192834 |
|  | g1947 | 12806043 |
|  | BMd15-2* | 15139117 |
|  | 3RT-BE211343-2 | 19953720 |
|  | g1380 | 29589123 |
|  | g1853 | 35422165 |
|  | CPRF2-X58577-2 | 39083462 |
|  | 7IOMT-U97125-3a | 40474528 |
|  | 7IOMT-U97125-3b | 40479615 |
|  | 7IOMT-U97125-3c | 40494081 |
|  | Myb15-CA902486-1a | 40893607 |
|  | Myb15-CW652102-1 | 40893607 |
|  | Myb15-Z-2 | 40893697 |
|  | BM210-2* | 41699471 |
|  | LAR1-JF433916-2 | 42885284 |
|  | LAR-XM_003536262-2 | 42885284 |
|  | Myb5-NM_112200-6 | 43232651 |
|  | PAL2-P19142-2b | 43593265 |
|  | g487 | 44044352 |
|  | g1378 | 44289678 |
|  | g366 | 44862402 |
|  | g2531 | 44862402 |
|  | g1233 | 45475464 |
|  | C4H-CV670736 | 46771937 |
|  | Bng42-F-2* | 46772843 |
|  | HIDH-AB154415-3 | 47241868 |
|  | g2129 | 47374155 |
|  | g501 | 47688343 |
|  | CAD-X62344-2 | 48109861 |
|  | g1615 | 48383045 |
|  | MybTT2-1-L1 | 48945586 |
|  | C4H-J09447-2 | 49233735 |
|  | g1719 | 49425766 |
|  | g2298 | 49458709 |
|  | CHI1B2-AY595414-2 | 50409724 |
|  | G4DT-AB434690 | 50652977 |
| Gm11 | HIDH-AB154415-2 | 305133 |
|  | 4CL3-AF002258-1a | 728019 |
|  | CHS-CV670744-2 | 793271 |
|  | CHS-X06411-1(CHS8) | 793491 |
|  | CHS-B-Z-1 | 793491 |
|  | g1515 | 793491 |
|  | ANS-AF325853-2 | 1983769 |
|  | ANS2-AY382829-2 | 1983769 |
|  | ANS3-AY382830-2 | 1983769 |
|  | g1686 | 2777697 |
|  | g2101 | 3082562 |
|  | g1307 | 3187422 |
|  | g797 | 3581983 |
|  | IglyT-FD796931 | 3999570 |
|  | g511 | 4153540 |
|  | g2213 | 4820792 |
|  | IFR1-AF202183-1a | 5251186 |
|  | IFR1-AF202183-1b | 5255032 |
|  | IFR1-AF202183-1c | 5264338 |
|  | F5H-U38416-3 | 5526663 |
|  | F5H-CV670742-2 | 5526663 |
|  | 4CL3-AF002258-1b | 6919458 |
|  | MybL2-NM_105772-2 | 8161261 |
|  | g785 | 8748576 |
|  | g2273 | 9004342 |
|  | g1731 | 9894814 |
|  | g1415 | 10198533 |
|  | g1932 | 10275277 |
|  | g2307 | 10784153 |
|  | Myb111-NM_124319-7 | 10884176 |
|  | FNS2-GU575290-2 | 10958112 |
|  | g2285 | 11369889 |
|  | g1438 | 11423942 |
|  | g2135 | 11931775 |
|  | g1489 | 16202763 |
|  | 4CL4-X69955-2 | 16819001 |
|  | g835 | 17353964 |
|  | g136 | 17800944 |
|  | g564 | 24346293 |
|  | LAC-AF527604-2b | 30365037 |
|  | g1192 | 30915872 |
|  | g1224 | 36332989 |
|  | AAT-CA900148-1 | 36720504 |
|  | AAT-CV670746-1 | 36720504 |
|  | g499 | 37118304 |
|  | CV542014 | 37274042 |
|  | LAC-AF527604-2a | 37321739 |
|  | g510 | 37936719 |
|  | g2132 | 38012422 |
|  | g683 | 38334238 |
|  | g2562 | 38498605 |
|  | g1367 | 39002116 |
| Gm12 | g909 | 136883 |
|  | g1329 | 297835 |
|  | MybL2-NM_105772-4 | 2412543 |
|  | g785 | 2936060 |
|  | g2273 | 3195175 |
|  | g1415 | 4219309 |
|  | g1932 | 4266364 |
|  | g2307 | 4800665 |
|  | FNS2-GU575290-1b | 4908632 |
|  | FNS2-GU575290-1a | 4917640 |
|  | g1438 | 5323535 |
|  | g2285 | 5380069 |
|  | g1671 | 5635091 |
|  | g835 | 5783425 |
|  | g1489 | 6788947 |
|  | g2527 | 9136123 |
|  | MybTT2-2-L2 | 9469066 |
|  | g1510 | 10244817 |
|  | g1168 | 11676847 |
|  | g156 | 25404759 |
|  | g1957 | 32288817 |
|  | g2383 | 32482889 |
|  | g1174 | 32482889 |
|  | g2480 | 32482889 |
|  | g634 | 32552683 |
|  | g1968 | 32985108 |
|  | g985 | 35321070 |
|  | g292 | 37733269 |
|  | g2507 | 38200286 |
|  | g1395 | 38339552 |
|  | g1226 | 39008805 |
|  | VR-EV925557-4c | 39739707 |
|  | VR-EV925557-4a | 39744244 |
|  | VR-EV925557-4b | 39748567 |
| Gm13 | 4CL1-CV670737-2 | 798836 |
|  | 4CL2-AF002259-1b | 798836 |
|  | FOGT-Glyma13g01220-1 | 914991 |
|  | TT1-WIP-CA902512-1 | 1391254 |
|  | WIP-CW652101-2 | 1391254 |
|  | FLS1-NM_001250490-1 | 2707784 |
|  | Satt160--W1(F3'5'H)** | 3993204 |
|  | g2538 | 4101977 |
|  | F3'5'H-AY117551-1 | 4552813 |
|  | SL019-F--W1(F3'5'H)** | 4552901 |
|  | SN019-F--W1(F3'5'H)** | 4556157 |
|  | SL018-F--W1(F3'5;H** | 4557025 |
|  | Satt348--W1(F3'5'H)** | 5491146 |
|  | g1375 | 5566164 |
|  | IF7MaT-AB291058-1a | 6501616 |
|  | IF7MaT-AB291058-1b | 6762198 |
|  | Bng71-F-1* | 6931703 |
|  | g2685 | 7807024 |
|  | g934 | 9872718 |
|  | CHS-X06411-8 | 11153569 |
|  | Myb5-NM_112200-1 | 11547033 |
|  | GST1-AY220095-3 | 19373899 |
|  | Myb12-NM_130314-3 | 20751545 |
|  | g2476 | 20815872 |
|  | g2654 | 21211880 |
|  | g417 | 21526734 |
|  | Bng75-F-1* | 21598107 |
|  | g415 | 24149862 |
|  | PAL2-CV670734-2 | 24273024 |
|  | PAL3-CV670735-2 | 24273024 |
|  | PAL2-Z-2 | 24273024 |
|  | PAL2-P19142-1 | 24273025 |
|  | g1065 | 24843338 |
|  | g1357 | 25615991 |
|  | g117 | 26803671 |
|  | PER-FBP4-AF149279-4 | 26988435 |
|  | IFS2-AF195819-1 | 27567230 |
|  | IFS-CV670762-2 | 27567230 |
|  | 7IOMT-CV670751-1 | 27576175 |
|  | HI4'OMT-AB091684-1 | 27576175 |
|  | g893 | 27803332 |
|  | PPO-ABL67928-2a | 28423043 |
|  | PPO-ABL67928-2c | 28448239 |
|  | PPO-ABL67928-2b | 28493023 |
|  | g1983 | 29482058 |
|  | VR-EV925557-5 | 30581801 |
|  | g1215 | 30601821 |
|  | g861 | 32311115 |
|  | g1488 | 32580775 |
|  | g2192 | 33144685 |
|  | g1998 | 33804895 |
|  | g2208 | 33918567 |
|  | PPO-ABL67928-2d | 33991444 |
|  | g1818 | 34044845 |
|  | g1280 | 34664990 |
|  | FNGalT-DB977791-1 | 34928015 |
|  | CHI3-AY595416-1 | 35462234 |
|  | g1226 | 36051576 |
|  | g1395 | 36761910 |
|  | g292 | 37323929 |
|  | g1968 | 40681809 |
|  | Myb111-NM_124319-8 | 41273374 |
|  | g174 | 41338044 |
|  | g2311 | 41371201 |
|  | g1333 | 41843024 |
|  | CV533374 | 41926545 |
|  | g1689 | 42114208 |
|  | g2308 | 42687814 |
|  | g1664 | 43551639 |
|  | g1883 | 43685634 |
|  | CCR2-AF320623-4 | 44081597 |
|  | CCR-CV670739-1 | 44081597 |
|  | 4CL2-AF002259-1a | 44256628 |
|  | 4CL2-CV670738-1 | 44256628 |
| Gm14 | COMT-M63853-3 | 402041 |
|  | CHR-EU921437-2 | 456385 |
|  | g2061 | 825705 |
|  | BMd44-2* | 840018 |
|  | Bng73-F-1* | 1139762 |
|  | g796 | 1417595 |
|  | g2316 | 1667934 |
|  | GST2-CV670756-2 | 2247489 |
|  | GST2-AY099257-2 | 2247490 |
|  | VT-CA907023-1 | 2353106 |
|  | VT-CV670747-1 | 2353106 |
|  | g580 | 2677311 |
|  | C-OAP2700-F-2* | 2801234 |
|  | LAC-AF527604-3a | 4367564 |
|  | g2274 | 4860979 |
|  | g1181 | 4870205 |
|  | g2264 | 4894197 |
|  | AAT-CA900148-3 | 4919153 |
|  | g1795 | 5977529 |
|  | DFR1-AF167556-1 | 6000541 |
|  | DFR1-NM_001251683-1 | 6000541 |
|  | DFR2-DQ026299-2b | 6000541 |
|  | DFR-Z-2 | 6000541 |
|  | DFR1-M-1 | 6000541 |
|  | DFR2-M-1 | 6000541 |
|  | DFR3-M-2 | 6000541 |
|  | DFR2-DQ026299-2a | 6010108 |
|  | g544 | 6027565 |
|  | g1645 | 6508170 |
|  | g2145 | 7301879 |
|  | g2266 | 7817138 |
|  | LIM-BU764417-4 | 7942630 |
|  | g1410 | 13572069 |
|  | g822 | 18970980 |
|  | g794 | 24869254 |
|  | Myb5-NM_112200-2 | 29396832 |
|  | g934 | 34322879 |
|  | VR-EV925557-3 | 41350815 |
|  | TT1-WIP-CA902512-2 | 43919359 |
|  | WIP-CW652101-1 | 43919560 |
|  | g471 | 46288452 |
|  | g1773 | 46288452 |
|  | g2151 | 46713761 |
|  | LAC-AF527604-3b | 47109835 |
|  | g2413 | 47391305 |
|  | C4H-J09447-3 | 47721318 |
|  | g1858 | 47721318 |
|  | g1713 | 48273861 |
| Gm15 | 4CL2-CV670738-2 | 190218 |
|  | 4CL2-AB002259-2 | 190549 |
|  | CCR2-AF320623-1 | 331629 |
|  | CCR-CV670739-2 | 331629 |
|  | g1664 | 832888 |
|  | g2308 | 1770744 |
|  | g1689 | 2387975 |
|  | CV533374 | 2596631 |
|  | g1333 | 2665120 |
|  | g174 | 3168864 |
|  | Myb111-NM_124319-6 | 3219634 |
|  | g1190 | 3825150 |
|  | 3RT-BE211343-3 | 4023433 |
|  | PER-FBP4-AF149279-1a | 4124462 |
|  | PER-FBP4-CW652095-1 | 4124462 |
|  | PER-FBP4-AF149279-1b | 4128648 |
|  | PER-FBP4-AF149279-1c | 4137377 |
|  | FNGalT-DB977791-2 | 4522893 |
|  | g1280 | 4786889 |
|  | g1818 | 5199130 |
|  | PPO-ABL67928-3a | 5423442 |
|  | PPO-ABL67928-3b | 5435993 |
|  | g2208 | 5498261 |
|  | g1998 | 5594149 |
|  | g2192 | 6136725 |
|  | g1488 | 6660618 |
|  | g861 | 6901796 |
|  | g1159 | 8506526 |
|  | g1249 | 8857946 |
|  | Bng27-F-1* | 9101770 |
|  | g1748 | 9300006 |
|  | PER-FBP1-AF149277-1f | 10109836 |
|  | PER-FBP1-CW652094-2 | 10122158 |
|  | PER-FBP1-AF149277-1a | 10122187 |
|  | PER-FBP1-AF149277-1b | 10131627 |
|  | PER-FBP1-AF149277-1c | 10138726 |
|  | PER-FBP1-AF149277-1d | 10146240 |
|  | PER-FBP1-AF149277-1e | 10154126 |
|  | Myb12-NM_130314-2 | 11823043 |
|  | g2209 | 12056042 |
|  | g860 | 12659002 |
|  | g385 | 12766860 |
|  | g2335 | 12864223 |
|  | g195 | 12864223 |
|  | I2'H-AB001379-3a | 13058034 |
|  | I2'H-AB001379-3b | 13079515 |
|  | Myb123(TT2)-AJ299452-2 | 16631713 |
|  | g1379 | 17689334 |
|  | CHR-EU921437-4 | 20019179 |
|  | g2580 | 22244290 |
|  | g1983 | 42869468 |
|  | CHI-AY595416-2 | 45616919 |
|  | g2596 | 47102151 |
|  | CHR-EU157917 | 47884091 |
|  | g1188 | 50089754 |
| Gm16 | L-OL4525-F-2* | 493954 |
|  | g1643 | 1255073 |
|  | Bng68-F-1* | 1814667 |
|  | g1994 | 1837265 |
|  | Myb111-NM_124310-3 | 2157868 |
|  | Myb4-CW652106-2 | 2157868 |
|  | g2647 | 3592555 |
|  | BMd44-1* | 5390400 |
|  | g1758 | 6179439 |
|  | I2'H-AB001379-4 | 12197040 |
|  | g2514 | 14673087 |
|  | IFR-CV670750-1 | 19294324 |
|  | g2558 | 27560220 |
|  | F3H-AY669326-3 | 27725414 |
|  | F3H-CW652103-2 | 27725414 |
|  | g2218 | 27725414 |
|  | g664 | 27763520 |
|  | F5H-U38416-4 | 28198101 |
|  | g2221 | 30624881 |
|  | BM68-2* | 32142659 |
|  | A3OGT-BE190894-1c | 33163914 |
|  | A3OGT-BE190894-1b | 33177016 |
|  | A3OGT-BE190894-1a | 33191272 |
|  | A3OGT-BE190894-1f | 33209634 |
|  | A3OGT-BE190894-1e | 33218242 |
|  | A3OGT-BE190894-1d | 33235219 |
|  | g1233 | 33409804 |
|  | BM140-2* | 35766953 |
|  | BMd26-2* | 36406092 |
|  | g2595 | 37104093 |
|  | CHR-CW652099-1 | 37180576 |
| Gm17 | KAP2-AF293344-3 | 542091 |
|  | Myb111-NM_124319-5 | 2316582 |
|  | g586 | 2358685 |
|  | g1656 | 2899644 |
|  | g2548 | 2903194 |
|  | Bng75-F-2* | 3066205 |
|  | g417 | 3131270 |
|  | g2476 | 4052511 |
|  | Myb123(TT2)-AJ299452-1 | 4106284 |
|  | g125 | 5141524 |
|  | 4CL1-AF279267-1b | 5222706 |
|  | 4CL1-AF279267-1c | 5244221 |
|  | 4CL1-AF279267-1a | 5257986 |
|  | 4CL1-CV670737-1 | 5257986 |
|  | FOGT-Glyma13g01220-2 | 5356106 |
|  | g2371 | 6172757 |
|  | F3'H-AB061212-3 | 6333712 |
|  | LIM-BU764417-3b | 7489954 |
|  | Myb176-FJ550209-2 | 7663874 |
|  | g117 | 8575815 |
|  | g2409 | 10243560 |
|  | g686 | 10426265 |
|  | g128 | 10635968 |
|  | g1801 | 10940703 |
|  | g1808 | 11914351 |
|  | Myb5-NM_112200-3 | 11955213 |
|  | g2341 | 15078684 |
|  | g1805 | 18046298 |
|  | g665 | 18700495 |
|  | g794 | 27473031 |
|  | g822 | 32511358 |
|  | UFGlyT-AF198453-2 | 33191272 |
|  | g1410 | 36116513 |
|  | g1671 | 38404853 |
|  | LIM-BU764417-3a | 39269900 |
|  | g2266 | 39398628 |
|  | g2145 | 39873382 |
|  | g1645 | 40544056 |
|  | DFR1-AF167556-2 | 40920379 |
|  | DFR1-NM_001251683-2 | 40920379 |
|  | DFR2-DQ026299-1 | 40920379 |
|  | DFR2-M-2 | 40920379 |
|  | DFR3-M-1 | 40920379 |
|  | g1795 | 40950826 |
| Gm18 | g523 | 31200 |
|  | g510 | 111191 |
|  | g2132 | 180621 |
|  | UF5GT-AB013596-3 | 262086 |
|  | UF5GT-CV670749-1 | 262086 |
|  | g2562 | 692279 |
|  | g1367 | 1156069 |
|  | LAC-AF527604-1a | 1723142 |
|  | LAC-CV670743-2 | 1723142 |
|  | CV542014 | 1784034 |
|  | g1436 | 1830185 |
|  | g499 | 1945752 |
|  | AAT-CA900148-2 | 2269841 |
|  | AAT-CV670746-2 | 2269841 |
|  | g1224 | 2644821 |
|  | C-OAP2700-F-1* | 4573999 |
|  | g1192 | 4774488 |
|  | LAC-AF527604-1b | 5028074 |
|  | g471 | 5844745 |
|  | LAC-AF527604-1c | 5888501 |
|  | BMd12-1* | 6604652 |
|  | g139 | 6886755 |
|  | F6H-XM_003551443-1 | 7686955 |
|  | g1639 | 9843467 |
|  | GST2-AY099257-3c | 13118613 |
|  | GST2-AY099257-3b | 13149832 |
|  | GST2-AY099257-3a | 13164051 |
|  | VT-CV670747-2 | 14483543 |
|  | VT-CA907034-2 | 14483877 |
|  | g2553 | 14619412 |
|  | g1520 | 16300302 |
|  | Bng47-F-2* | 22012716 |
|  | 3RT-CA914524-1 | 33368085 |
|  | 3RT-CV670748-1 | 33368084 |
|  | BM165-1* | 46995357 |
|  | GST1-AY220095-1b | 50177763 |
|  | GST1-AY220095-1a | 50296558 |
|  | GST1-CW652097-1 | 50296560 |
|  | g140 | 50304042 |
|  | BMd25-2* | 51665056 |
|  | g776 | 52256979 |
|  | g1990 | 54863721 |
|  | VR-EV925557-11 | 55008785 |
|  | VR-EV925557-1b | 55014596 |
|  | PPO-ABL67928-6 | 55592071 |
|  | HD-GL2l1/ANL2-CA902455-2 | 55667266 |
|  | HD-CV670753-2 | 55667264 |
|  | g1031 | 55912056 |
|  | g549 | 56723615 |
|  | g131 | 57053145 |
|  | g2561 | 57288823 |
|  | g696 | 57305442 |
|  | g2462 | 57349763 |
|  | g2567 | 58458501 |
|  | g1119 | 58973387 |
|  | MybA-Glyma09g36990-2a | 59036003 |
|  | MybA-Glyma09g36990-2b | 59060487 |
|  | Gy-W17S | 59072960 |
|  | HI4'OMT-AB091684-2c | 59451551 |
|  | HI4'OMT-AB091684-2a | 59464284 |
|  | HI4'OMT-AB091684-2d | 59471368 |
|  | 7IOMT-CV670751-2 | 59471368 |
|  | HI4'OMT-AB091684-2b | 59601194 |
|  | g1879 | 60199171 |
|  | g2393 | 60720284 |
|  | CHR-EU921437-3 | 60909653 |
|  | g89 | 60969639 |
|  | g2311 | 61162047 |
|  | g1786 | 61889084 |
|  | g1588 | 61964545 |
|  | g1084 | 62243638 |
| Gm19 | g2467 | 740074 |
|  | F5H-U38416-2 | 1864175 |
|  | F5H-CV670742-1 | 1864175 |
|  | g1375 | 2306077 |
|  | g2303 | 2743480 |
|  | MybAN2-FG986768 | 3087401 |
|  | IF7MaT-AB291058-2a | 3748793 |
|  | IF7MaT-AB291058-2b | 3763949 |
|  | IF7MaT-AB291058-2c | 3769271 |
|  | Bng71-F-2* | 4482985 |
|  | g2685 | 7216940 |
|  | g1758 | 29584550 |
|  | CHS-A-Z-1 | 35280136 |
|  | CHS-X06411-7 | 35280425 |
|  | g2647 | 36747960 |
|  | C3H-AY065995-1 | 38217224 |
|  | C3H-CV670754-1 | 38217224 |
|  | g743 | 38658381 |
|  | g1737 | 39512630 |
|  | g2490 | 39843036 |
|  | g440 | 40400559 |
|  | PER-FBP1-AF149277-3 | 40721621 |
|  | g1959 | 41169291 |
|  | g724 | 41607755 |
|  | g1771 | 43656990 |
|  | PAL1-M11939-1 | 43914640 |
|  | PAL1-CV670758-1 | 43914640 |
|  | PAL3-Z-2 | 43914640 |
|  | g1952 | 44626053 |
|  | g1954 | 44626053 |
|  | g2162 | 45517825 |
|  | Myb11-NM_116126 | 46673096 |
|  | g1886 | 46811144 |
|  | g2504 | 46817050 |
|  | g786 | 47038077 |
|  | g1404 | 47202729 |
|  | CPRF2-X58577-3 | 49077921 |
|  | g1361 | 49548595 |
|  | Myb4-CA902489-1 | 50015376 |
|  | Myb4-CW652106-1 | 50015376 |
|  | FOMT-Glyma04g40480-3 | 50265557 |
| Gm20 | MybL2-NM_105772-3 | 1123688 |
|  | F3'5'H-AY117551-2 | 11345405 |
|  | g2538 | 15039921 |
|  | g1380 | 23746063 |
|  | g1853 | 30090281 |
|  | g125 | 30345305 |
|  | g2298 | 34161799 |
|  | g1719 | 34186091 |
|  | C4H-J09447-1 | 34452404 |
|  | C4H-CV670736-1 | 34452632 |
|  | MybTT2-1-L2 | 34824216 |
|  | g1615 | 35585334 |
|  | CAD-X62344-1 | 35920481 |
|  | Bng42-F-1* | 36282651 |
|  | g2129 | 36959159 |
|  | HIDH-AB154415-4 | 37104686 |
|  | g2531 | 39491213 |
|  | g366 | 39491213 |
|  | g1378 | 40110770 |
|  | PAL3-P19143-2 | 40779183 |
|  | Myb5-NM_112200-4a | 41105162 |
|  | Myb5-NM_112200-4b | 41116470 |
|  | LAR1-JF433916-1 | 41312511 |
|  | LAR-XM_003536262-1 | 41312511 |
|  | BM210-1* | 42724762 |
|  | Myb15-CW652102-2 | 43456265 |
|  | Myb15-Z-1 | 43456265 |
|  | Myb15-CA902486-2 | 43456265 |
|  | 7IOMT-U97125-2c | 43867834 |
|  | 7IOMT-U97125-2d | 43871700 |
|  | 7IOMT-U97125-2a | 43882581 |
|  | 7IOMT-U97125-2b | 43884371 |
|  | CPRF2-X58577-1 | 44787193 |
|  | CHI-CV670745-1 | 46127096 |
|  | CHI1A-AY595414-1a | 46127173 |
|  | CHI2-AY595414-1b | 46131958 |
|  | CHI1B1-AY595414-1c | 46134762 |
