# Supplementary material for: In silico comparison of genomic regions containing genes coding for enzymes and transcription factors for the phenylpropanoid pathway in Phaseolus vulgaris L. and Glycine max L. Merr
Source: Front Plant Sci. 2013 Sep 5;4:317. doi: 10.3389/fpls.2013.00317 (PMC3763686; doi:10.3389/fpls.2013.00317)
Supplement: Figure S1 — Phylogenetic relationships among common bean and soybean 4CL homologous proteins. Trees were generated from 4CL protein sequence analyses in CLC Genomics Workbench 3 using Neigbor Joining (NJ) algorithm and 1000 bootstrap replications. Scale represents genetic distance and node numbers indicate bootstrap support values. (A) Primary transcripts (98 protein sequences); (B) Twenty-one 4CL proteins selected based on similarity to soybean 4CL1 (red), 4CL2 (blue) and 4CL3/4CL4 (green). [file DataSheet1.ZIP › Supplementary Material/Supplemental Table S3 - Exon intron structure of selected 4CL genes in Gm and PV.doc]

**Table S3 | Exon/intron structure of selected 4CL genes in common bean and soybean.**

| Enzyme | Locus* | Gene | Gene structure** | | | | | | | | | | | | | Total E | Total I | Total  E+I |
| --- | --- | --- | --- | --- | --- | --- | --- | --- | --- | --- | --- | --- | --- | --- | --- | --- | --- | --- |
|  |  |  | 5’UTR | E1 | I1 | E2 | I2 | E3 | I3 | E4 | I4 | E5 | I5 | E6 | 3’UTR |  |  |  |
| 4CL1 | Glyma17g07190.2 | Gm4CL1-1 | 878 | 1,002 | 780 | 196 | 2,196 | 214 | 474 | 103 | 372 | 126 | - | - | 975 | 1,641 | 3,822 | 5,463 |
|  | Glyma13g01080.1 | Gm4CL1-2 | NA | 999 | 1,765 | 196 | 2,234 | 214 | 759 | 280 | - | - | - | - | NA | 1,689 | 4,758 | 6,447 |
|  | Phvul.003G147000.1 | Pv4CL1 | NA | 1,020 | 417 | 196 | 2,053 | 214 | 175 | 103 | 100 | 112 | - | - | NA | 1,645 | 2,745 | 4,390 |
| 4CL2 | Glyma13g44950.1 | Gm4Cl2-1 | 521 | 1,005 | 179 | 199 | 590 | 214 | 78 | 106 | 314 | 120 | - | - | 612 | 1,644 | 1,161 | 2,805 |
|  | Glyma15g00390.1 | Gm4CL2-2 | NA | 978 | 343 | 199 | 719 | 214 | 78 | 106 | 244 | 120 | - | - | NA | 1,617 | 1,384 | 3,001 |
|  | Phvul.005G184500.1 | Pv4CL2 | NA | 1,201 | 153 | 214 | 84 | 94 | 95 | 126 | - | - | - | - | NA | 1,635 | 332 | 1,967 |
| 4CL3/4 | Glyma11g01240.1 | Gm4CL3 | 649 | 1,071 | 1,936 | 199 | 117 | 146 | 102 | 68 | 93 | 103 | 170 | 126 | 841 | 1,713 | 2,418 | 4,131 |
|  | Glyma01g44270.1 | Gm4CL4 | 594 | 1,048 | 1,767 | 199 | 130 | 146 | 103 | 68 | 97 | 103 | 173 | 126 | 604 | 1,690 | 2,270 | 3,960 |
|  | Phvul.002G040100.1 | Pv4CL3/4 | NA | 1,047 | 985 | 345 | 109 | 68 | 112 | 103 | 118 | 138 | - | - | NA | 1,701 | 1,324 | 3,025 |

*Phytozome/ GenBank

**E, exons; I, introns

NA, not available
